# Design and Synthesis of AMPK Activators and GDF15 Inducers

**DOI:** 10.3390/molecules28145468

**Published:** 2023-07-17

**Authors:** Meijian Zhang, Andrea Bagán, Donna Martínez, Emma Barroso, Xavier Palomer, Santiago Vázquez, Carmen Escolano, Manuel Vázquez-Carrera

**Affiliations:** 1 Department of Pharmacology, Toxicology and Therapeutic Chemistry, Institute of Biomedicine (IBUB), Faculty of Pharmacy and Food Sciences, University of Barcelona, Av. Joan XXIII, 27-31, 08028 Barcelona, Spain; zmeijian@gmail.com (M.Z.); ebarroso@ub.edu (E.B.); xpalomer@ub.edu (X.P.); 2 Spanish Biomedical Research Center in Diabetes and Associated Metabolic Diseases (CIBERDEM)-Instituto de Salud Carlos III, 28029 Madrid, Spain; 3 Pediatric Research Institute-Hospital Sant Joan de Déu, 08950 Esplugues de Llobregat, Spain; 4 Laboratory of Medicinal Chemistry (Associated Unit to CSIC), Department of Pharmacology, Toxicology and Medicinal Chemistry, Faculty of Pharmacy and Food Sciences, Institute of Biomedicine (IBUB), University of Barcelona, Av. Joan XXIII, 27-31, 08028 Barcelona, Spain; andreabaganp@gmail.com (A.B.); luzy779@gmail.com (D.M.); svazquez@ub.edu (S.V.)

**Keywords:** GDF15, AMPK, BC1618, (1-dibenzylamino-3-phenoxy)propan-2-ol

## Abstract

Targeting growth differentiation factor 15 (GDF15) is a recent strategy for the treatment of obesity and type 2 diabetes mellitus (T2DM). Here, we designed, synthesized, and pharmacologically evaluated in vitro a novel series of AMPK activators to upregulate GDF15 levels. These compounds were structurally based on the (1-dibenzylamino-3-phenoxy)propan-2-ol structure of the orphan ubiquitin E3 ligase subunit protein Fbxo48 inhibitor, **BC1618**. This molecule showed a better potency than metformin, increasing *GDF15* mRNA levels in human Huh-7 hepatic cells. Based on **BC1618**, structural modifications have been performed to create a collection of diversely substituted new molecules. Of the thirty-five new compounds evaluated, compound **21** showed a higher increase in *GDF15* mRNA levels compared with **BC1618**. Metformin, **BC1618**, and compound **21** increased phosphorylated AMPK, but only **21** increased GDF15 protein levels. Overall, these findings indicate that **21** has a unique capacity to increase GDF15 protein levels in human hepatic cells compared with metformin and **BC1618**.

## 1. Introduction

Growth differentiation factor 15 (GDF15) (also known as nonsteroidal anti-inflammatory drug-activated gene, placental bone morphogenetic protein, placental transforming growth factor-ß, and prostate-derived factor) is a stress-induced cytokine that is involved in appetite regulation [1]. In fact, the increase in GDF15 levels promotes an anorectic effect that leads to a reduction in body weight through its binding to its central receptor glial cell-line-derived neurotrophic factor (GDNF)-like alpha-1 (glial cell-derived neurotrophic factor family receptor alpha-like (GFRAL)) [1]. Consistent with this role of GDF15, transgenic mice overexpressing *Gdf15* display a lean phenotype and a reduction in food intake and are more resistant to obesity, metabolic inflammation, and glucose intolerance [2,3]. Likewise, administration of recombinant GDF15 (rGDF15) has been reported to reduce food intake and body weight in mice, but these effects are absent in *Gfral*-knockout mice [2,4,5]. Overall, these findings suggest that pharmacological modulation of GDF15 shows promise for the treatment of obesity and its complications, such as type 2 diabetes mellitus (T2DM). Indeed, GDF15 analogs, which try to overcome the pharmacokinetic (e.g., a short half-life of ~3 h in mice and non-human primates) and physicochemical (e.g., high aggregation propensity) limitations of native GDF15 [1,6,7], have been developed for the treatment of obesity. Another potential strategy to target the GDF15–GFRAL pathway involves the pharmacological regulation of endogenous GDF15 levels. Interestingly, metformin, the most prescribed drug for the treatment of T2DM, has been reported to increase serum GDF15 in patients [8]. Subsequently, two studies reported that the anorectic response of mice to metformin requires an intact GDF15–GFRAL pathway [9,10]. In addition, the increase in GDF15 levels caused by metformin was associated with the reduction in body weight in patients with [10] or without [9] T2DM. Although a recent study reported that the effects of metformin on body weight were independent of the GDF15–GFRAL signaling in some circumstances [11], a new study confirmed the involvement of this pathway on the effects of metformin on body weight [12]. These findings validate that targeting endogenous GDF15 may serve as a pharmacological approach to treat obesity and T2DM. Interestingly, many of the antidiabetic effects of metformin are mediated by the activation of the adenosine monophosphate-activated protein kinase (AMPK) [13], and we reported that the increase in GDF15 caused by metformin involves the activation of this kinase [14]. Moreover, we observed that the antidiabetic effect of a low dose of metformin is not observed in *Gdf15*-knockout mice [14]. These findings suggest that targeting AMPK may provide new possibilities to increase endogenous levels of GDF15 to treat obesity and T2DM.

In this study, we designed, synthesized, and pharmacologically evaluated in vitro a novel series of AMPK activators, aiming at increasing *GDF15* expression. These compounds are structurally based on the (1-dibenzylamino-3-phenoxy)propan-2-ol skeleton of the orphan ubiquitin E3 ligase subunit protein Fbxo48 inhibitor, **BC1618**, which exceeds metformin potency for stimulating AMPK [15]. In this context, we carried out conservative structural modifications on the substituents in the phenoxy ring and in the amine moiety. Our findings show that one of the new compounds synthesized, **21**, exhibits substantially better potency compared to metformin and **BC1618** towards increased GDF15 mRNA and protein levels in human hepatic cells.

## 2. Results 

### 2.1. Synthesis of GDF15 Inducers

**Scheme 1:**
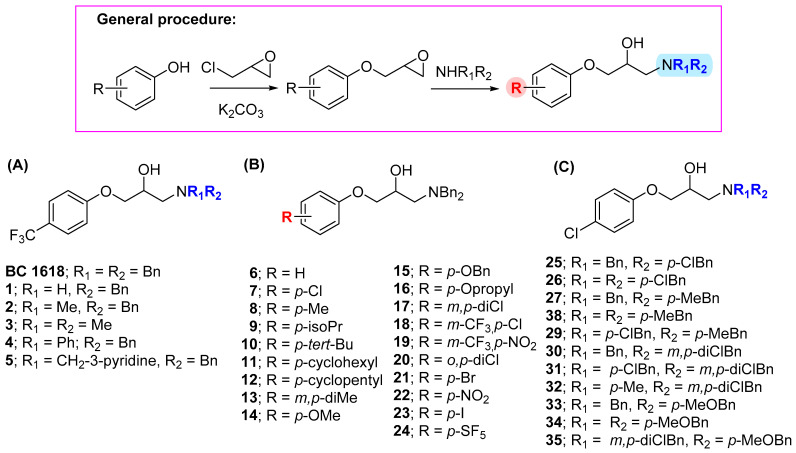
General procedure for the synthesis of compounds (**1**–**35**) with modifications in the amino substituents (in blue) and in the substituents of the phenoxy group (in red). General structures, (**A**) variations in the substituents in the amino group of the *p*-trichlorophenyl general structure, (**B**) variations in the substituents in the phenoxy group, and (**C**) variations in the substituents in the amino group of the *p*-chlorophenyl general structure.

The synthesis of **BC1618** was performed as described [16]. The other products were synthetized following the same synthetic strategy (Scheme 1). In this manner, a methanol solution of the required phenol (1 equiv.) with epichlorohydrin (10 equiv.) in the presence of potassium carbonate (1.2 equiv.) was heated at reflux for 4 h, leading to the corresponding phenoxymethyloxirane intermediates. In a second synthetic step, an equimolecular mixture of the phenoxymethyloxirane derivatives and the required primary or secondary amine were heated at 70 °C for 24 h to afford the final compounds (**1**–**35**) in good yields (see Section 4 for further details).

All the compounds synthesized for in vitro evaluation were fully characterized through their spectroscopic data (^1^H and ^13^C), infrared (IR), and high-resolution mass spectrometry (HMRS). The purity was determined by high-performance liquid chromatography (HPLC)/mass spectrometry, and only compounds with a purity >95% were considered for biological studies (see the Section 4 and the Supplementary Materials for further details).

### 2.2. Assessment of the Activity of the New Compounds Increasing GDF15 mRNA Levels in Huh-7 Human Hepatic Cells and Structural–Activity Relationship of the New Compounds

To evaluate the activity of the synthesized compounds (Scheme 1A–C) in increasing *GDF15* expression, we incubated Huh-7 human hepatic cells with the new compounds (30 µM) for 24 h, using metformin (5 mM) and **BC1618** (30 µM) as standards for comparison purposes. Hepatic cells were used since circulating GDF15 is primarily derived from the liver [17]. As shown in Table 1, metformin caused a 2.7-fold increase in *GDF15* mRNA levels compared with control cells. By contrast, a much lower concentration of **BC1618** caused a higher increase in *GDF15* expression (10.4-fold induction), thereby indicating that this compound shows a higher potency, which is consistent with the reported higher activation of AMPK [15]. 

To determine the impact that modifications in the different substituents could have in the biological activities (Table 1), we considered two main approximations starting from **BC1618**: (a) an exploration of the substituents in the amino moiety (right-hand side, RHS; highlighted in blue in the general structure in the Scheme 1) and (b) a study of the substituents at the phenoxy group (left-hand side, LHS; highlighted in red in the general structure in the Scheme 1). 

In this context, the simplification of the dibenzylamino substituent of **BC1618** to a benzylamino was considered, leading to compound **1**, which exhibited important increases in GDF15 expression compared with BC1618 but also increased cell mortality, suggesting some toxicity that rendered it unsuitable for further work. Compared with **BC1618**, an *N*-benzyl,*N*-methylamine substituent in **2** or *N*,*N*-dimethylamine in **3** resulted in substantial reductions in the expression of GDF15. An *N*-benzyl-*N*-phenylamino moiety in **4** caused no significant changes in GDF15 levels compared with **BC1618**, whereas the presence of an electron-deficient aromatic ring such as a 3-pyridine in **5** caused a dramatic reduction in GDF15 levels. 

At this point, we maintained unaltered the RHS part of the initial scaffold (*N*-dibenzylamino) and focused on the modifications of the substituents at the phenoxy moiety. Removal of the trifluoromethyl group, as in **6**, had no effect on GDF15 levels compared with **BC1618**. Comprehensive exploration of the RHS involved the replacement of the trifluoromethyl group by a chlorine atom in **7** or a methyl group in **8**, leading to small increases in the *GDF15* mRNA levels. Other replacements, featuring bulkier *p*-alkyl substituents, such as those conducted in **9** (isopropyl), **10** (*tert*-butyl), **11** (cyclohexyl), or **12** (cyclopentyl), did not improve the levels of the biomarker. Compound **13**, featuring a 3,4-dimethylphenoxy group, exhibited a remarkable increase in the GDF15 levels, although it was somehow toxic, showing an increase in the cell mortality. Compound **14**, embodying a *p*-methoxy group, depicted similar activity as **BC1618**. With the aim of exploring alternative *p*-alkyloxy substituents, **15** with a *p*-benzyloxy group and **16** with a *p*-propyloxy unit were synthesized, leading to a significant reduction in *GDF15* expression compared with **BC1618**. Next, we undertook the synthesis of disubstituted compounds **17–19**, featuring *m*,*p*-dichloro-, *p*-chloro,*m*-trifluoromethyl-, and *p*-nitro,*m*-trifluoromethyl-phenoxy units, respectively. The three new compounds did not improve the activity of **BC1618**. The presence of a *o*,*p*-dichloro atoms at the phenoxy group in **20** led to an increase in *GDF15* expression.

Considering the abovementioned results, in a final round, we selected compound **7**, with a *p*-chlorophenoxy group, and we introduced substituents in the dibenzylamino moiety. Therefore, we synthesized eleven new analogs, including **25** and **26**, with a *p*-chloro in one or two of the benzyl groups, respectively; **27** and **28**, with a *p*-methyl in one or two of the benzyl groups, respectively; and **29**, with a *p*-chloro and a *p*-methyl group in each benzyl group. All these compounds depicted a very important decrease in the *GDF15* levels compared with **BC1618**. Particularly, **25** was toxic for the cells, and the number of cells collected was too low to analyze *GDF15* expression. Increasing the number of chloro atoms in the benzyl substituents, as in **30** (*m*,*p*-dichloro), **31** (*m*,*p*-dichloro and *p*-chloro), and **32** (*m*,*p*-dichloro and *p*-methyl), resulted in a dramatical decrease in *GDF15* levels. Finally, moving to electron-donating groups, such as a *p*-methoxy group (**33** in one benzyl or **34** in both benzyl groups), or to a combination of *m*,*p*-dichloro on one ring and *p*-methoxy in the other ring (**35**) was deleterious for the biological activity.

Considering that compound **7**, featuring a dibenzylamino unit at the RHS of the molecule and a *p*-chlorophenoxy group at the LHS, provided the highest *GDF15* levels without toxicity, we finally explored the effect of replacing the *p*-chlorine atom by another electron-withdrawing group. Thus, we synthesized compound **21**, with a *p*-bromophenoxy group. Interestingly, this compound exhibited a marked increase in *GDF15* expression without toxicity issues. When a nitro group or an iodine atom occupied the *para* position of the phenoxy group in **22** and **23**, respectively, a decrease in *GDF15* levels was observed. Our experience in the study of the rather scarcely explored pentafluorosulfanyl substituent [18] prompted us to prepare compound **24**, which did not represent a biological improvement. Considering all the above-mentioned results, compound **21** was selected for further experiments. 

### 2.3. Evaluation of the Activity of 21 on AMPK Activation and GDF15 Protein Levels in Huh-7 Human Hepatic Cells

**Figure 1:**
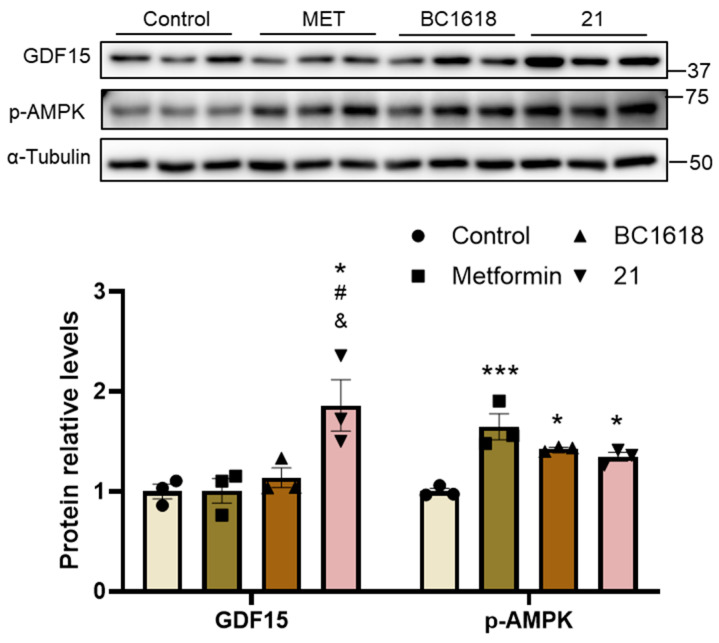
Compound **21** increases GDF15 protein abundance. GDF15 and phosphorylated AMPK protein levels in Huh-7 human hepatic cells exposed to metformin (MET) (5 mM), **BC1618** (10 µM), or **21** (10 µM) for 72 h. Data are presented as the mean ± SEM (*n* = 3). * *p* < 0.05, and *** *p* < 0.001 vs. control. # *p* < 0.05 vs. metformin-treated cells. & *p* < 0.05 vs. **BC1618**-treated cells. *p*-values determined by one-way ANOVA with Tukey’s post hoc test.

The effect of compound **21** on GDF15 protein levels compared with metformin and **BC1618** was evaluated in Huh-7 cells. The three compounds activated AMPK, determined by the increase in phosphorylated AMPK. However, of the three compounds, only **21** increased the protein abundance of GDF15 (Figure 1). 

## 3. Discussion

There is conclusive evidence that GDF15 is an attractive target for the treatment of obesity and T2DM [1]. Indeed, recombinant GDF15 and its analogs reduce food intake and body weight and improve glucose intolerance in animal models of obesity [1,6,7]. In addition, metformin increases circulating GDF15 levels [8]. The metformin-mediated increase in GDF15 levels may mediate part of its effects on body weight and glucose metabolism [9,10,14]. Therefore, pharmacological modulation of endogenous GDF15 levels by small molecules offers promise for the treatment of obesity and T2DM. In this line, it has been reported that metformin increases GDF15 levels via AMPK [14], converting this kinase in a target to increase this cytokine. In this study, we synthesized new compounds based on the (1-dibenzylamino-3-propoxy)propan-2-ol group of the orphan ubiquitin E3 ligase subunit protein Fbxo48 inhibitor, **BC1618** [15], to upregulate GDF15 levels. It is known that the potency of **BC1618** activating AMPK exceeds that of metformin [15], but little was known about the capacity of this compound to increase GDF15.

Here, we show that **BC1618** (10 µM) causes a strong increase in *GDF15* mRNA levels in human Huh-7 cells compared with metformin (5 mM). In addition, we synthesized and fully characterized thirty-five new analogues of **BC1618**, and we evaluated their effects on *GDF15* expression. All the new compounds increased GDF15 mRNA levels over the control, and a few of them were more potent than **BC1618**.

Compound **21**, which features a bromine atom instead of the trifluoromethyl group of **BC1618**, caused a higher increase in GDF15 levels compared with **BC1618** and was selected to evaluate its effects on GDF15 protein abundance compared with metformin and **BC1618**. The three compounds increased phosphorylated levels of AMPK. However, only **21** increased GDF15 protein levels. Although previous studies have reported that metformin administration increases serum GDF15 levels, the source tissue leading to the increase in this cytokine remains controversial [19]. Thus, two studies reported that metformin can induce GDF15 release from mouse primary hepatocytes [9,10], but one of these studies showed that metformin increased GDF15 levels in kidneys and intestines but not in the liver [9]. More recently, a new study demonstrated that acute metformin treatment elevated GDF15 levels in different tissues but not in the liver [11]. Therefore, it is likely that due to the slight increase in *GDF15* expression caused by metformin, longer exposures are needed to detect an increase in GDF15 protein abundance in hepatic cells. However, **BC1618** caused a higher increase in GDF15 mRNA levels compared to metformin, but this increase did not result in an elevation of the protein abundance of this cytokine. By contrast, **21** treatment increased GDF15 protein levels. These findings suggest that the substitution of the trifluoromethyl group by a bromine atom may contribute to increase GDF15 protein abundance. Although the reasons for this effect are unknown, several factors may contribute. For instance, a bigger atom such as bromine may affect the ability to interact with target proteins and receptors. In fact, a study investigating the effects of 3,3′-diindolylmethane and its synthetic halogenated derivatives found that a bromide derivative caused a higher activation of AMPK [20]. These findings suggest that the presence of bromide atoms in **21** could potentiate or sustain AMPK activation, leading to an increase in GDF15 protein levels. Although we did not observe a greater increase in phosphorylated AMPK following **21** treatment compared with **BC1618**, we only examined a single time point. Therefore, transient increases might have contributed to achieve a higher potency in AMPK activation. Further studies are needed to elucidate the mechanisms that contribute to increasing GDF15 protein levels. 

Overall, the findings of this study show that there is no correlation between GDF15 mRNA and protein levels and that the presence of a bromine atom in the structure of molecules activating AMPK and increasing *GDF15* expression allows to increase the protein abundance of this stress cytokine in hepatic cells. Moreover, we describe that new compound 21 has a unique capacity to increase GDF15 protein levels in human hepatic cells compared with metformin and BC1618.

## 4. Materials and Methods

### 4.1. Chemical Synthesis

#### 4.1.1. General Methods

Reagents, solvents, and starting products were acquired from commercial sources. When indicated, the reaction products were purified by “flash” chromatography on silica gel (35–70 μm) with the indicated solvent system. The melting points were measured in a MFB 59510M Gallenkamp instruments. IR spectra were performed in a spectrophotometer Nicolet Avantar 320 FTR-IR or in a Spectrum Two FT-IR Spectrometer, and only noteworthy IR absorptions (cm^−1^) are listed. NMR spectra were recorded in CDCl_3_ at 400 MHz (^1^H) and 101 MHz (^13^C), and chemical shifts are reported in δ values downfield from TMS or relative to residual chloroform (7.26 ppm, 77.0 ppm) as an internal standard. Data are reported in the following manner: chemical shift, multiplicity, coupling constant (*J*) in hertz (Hz), and integrated intensity and assignment (when possible). Multiplicities are reported using the following abbreviations: s, singlet; d, doublet; dd, doublet of doublets; t, triplet; q, quadruplet; m, multiplet; br s, broad signal; app, apparent. Assignments and stereochemical determinations are given only when they are derived from definitive dimensional NMR experiments (g-HSQC). The accurate mass analyses were carried out using a LC/MSD-TOF spectrophotometer. HPLC-MS (Agilent 1260 Infinity II) analysis was conducted on a Poroshell 120 EC-C15 (4.6 mm × 50 mm, 2.7 μm) at 40 °C with mobile phase A (H_2_O + 0.05% formic acid) and B (ACN + 0.05% formic acid) using a gradient elution and flow rate 0.6 mL/min. The DAD detector was set at 254 nm, the injection volume was 5 μL, and the oven temperature was 40 °C. 

General procedure for the preparation of final aryloxypropanolamines from aryloxyepoxydes and amines. The corresponding amines (1 equivalent) and the corresponding oxiranes (1 equivalent) were stirred under N_2_ atmosphere at 70 °C for 24 h. The reaction mixture was concentrated, and the resulting residue was purified by column chromatography to afford pure products.

#### 4.1.2. 1-(Dibenzylamino)-3-[4-(trifluoromethyl)phenoxy]propan-2-ol (BC1618)

Following the general procedure, a mixture of dibenzylamine (CAS 103-49-1) (0.09 mL, 0.47 mmol) and 2-{[4-(trifluoromethyl)phenoxy]methyl}oxirane (100 mg, 0.46 mmol) afforded BC1618 (149 mg, 78%) as a white solid after column chromatography (hexane/DCM 9:1 to 8:2). IR (ATR) 3412, 3029, 2924, 1613, 1451, 1261, 1034, 840, 745, 698 cm^−1^. ^1^H NMR (500 MHz, CDCl_3_) δ 2.69 (d, *J* = 7.0 Hz, 2H, CH_2_N), 3.56 (d, *J* = 13.5 Hz, 2H, CH_2_Ph), 3.82 (d, *J* = 13.5 Hz, 2H, CH_2_Ph), 3.85–3.94 (m, 2H, CH_2_O), 4.06–4.14 (m, 1H, CH), 6.88 (d, *J* = 8.5 Hz, 2H, ArH), 7.24–7.30 (m, 2H, ArH), 7.31–7.35 (m, 8H, ArH), 7.51 (d, *J* = 8.5 Hz, 2H, ArH). ^13^C NMR (126 MHz, CDCl_3_) δ 56.0 (CH_2_N), 59.0 (2CH_2_Ph), 66.4 (CH), 70.6 (CH_2_O), 114.6 (2CHAr), 121.3 (CAr), 123.2 (q, *J* = 32.5 Hz, CF_3_), 125.6 (CAr), 127.0 (d, *J* = 4.0 Hz, 2CHAr), 127.6 (2CHAr), 128.7 (4CHAr), 129.1 (CHAr), 129.3 (2CHAr), 130.0 (CHAr), 138.4 (CAr), 161.2 (OCAr) [15]. Purity 96.90% (t_R_ = 3.27 min).

#### 4.1.3. 1-(Benzylamino)-3-[4-(trifluoromethyl)phenoxy]propan-2-ol (**1**)

Following the general procedure, benzylamine (CAS 100-46-9) (0.05 mL, 0.46 mmol) and 2-{[4-(trifluoromethyl)phenoxy]methyl}oxirane (100 mg, 0.46 mmol) afforded **1** (82 mg, 55%) as a white solid after column chromatography (hexane/EtOAc 9:1 to 8:2). Mp 102–105 °C (EtOAc). IR (ATR) 3266, 1614, 1520, 1335, 1256, 1153, 1109, 1035, 836, 698 cm^−1^. ^1^H NMR (400 MHz, CDCl_3_, HETCOR) δ 2.47 (s, 2H, OH, NH), 2.79 (dd, *J* = 12.0, 8.0 Hz, 1H, CH_2_N), 2.91 (dd, *J* = 12.0, 4.0, Hz, 1H, CH_2_N), 3.79–3.91 (m, 2H, CH_2_Ph), 4.02 (d, *J* = 5.5 Hz, 2H, CH_2_O), 4.05–4.13 (m, 1H, CH), 6.96 (d, *J* = 8.5 Hz, 2H, ArH), 7.25–7.38 (m, 5H, ArH), 7.54 (d, *J* = 8.5 Hz, 2H, ArH). ^13^C NMR (101 MHz, CDCl_3_) δ 51.1 (CH_2_N), 53.9 (CH_2_Ph), 68.3 (CH), 70.7 (CH_2_O), 114.7 (2CHAr), 121.4–125.4 (m, CF_3_), 127.0 (2CHAr), 127.4 (2CHAr), 128.3 (3CHAr), 128.7 (CAr), 139.8 (CAr), 161.2 (OCAr). HRMS C_17_H_19_F_3_NO_2_ [M + H]^+^ 326.1362; found 326.1366. Purity 95.23% (t_R_ = 2.80 min). 

#### 4.1.4. 1-[Methyl(phenyl)amino]-3-[4-(trifluoromethyl)phenoxy]propan-2-ol (**2**)

Following the general procedure, *N*-benzylmethylamine (CAS 103-67-3) (0.06 mL, 0.46 mmol) and 2-{[4-(trifluoromethyl)phenoxy]methyl}oxirane (100 mg, 0.46 mmol) afforded **2** (73 mg, 47%) as a white solid after a reversed-phase column chromatography (H_2_O:ACN 95:5). Mp 53–56 °C (EtOAc). IR (ATR) 3432, 2843, 1615, 1519, 1327, 1258, 1153, 1107, 1068, 836, 699 cm^−1^. ^1^H NMR (400 MHz, CDCl_3_, HETCOR) δ 2.36 (s, 3H, CH_3_), 2.61 (dd, *J* = 12.5, 4.0, Hz, 1H, CH_2_), 2.71 (dd, *J* = 12.5, 10.0 Hz, 1H, CH_2_), 3.6 (br s, 1H, OH), 3.61 (d, *J* = 13.0 Hz, 1H, CH_2_Ph), 3.76 (d, *J* = 13.0 Hz, 1H, CH_2_Ph), 4.00 (dd, *J* = 5.0, 1.0 Hz, 2H, CH_2_), 4.12–4.21 (m, 1H, CH), 6.96 (d, *J* = 8.5 Hz, 2H, ArH), 7.34 (dd, *J* = 8.0, 2.0 Hz, 5H, ArH), 7.54 (d, *J* = 8.5 Hz, 2H, ArH). ^13^C NMR (101 MHz, CDCl_3_) δ 42.3 (CH_3_), 59.5 (CH_2_N), 62.6 (CH_2_Ph), 66.0 (CH), 70.5 (CH_2_O), 114.7 (2CHAr), 123.3 (q, *J* = 33.0 Hz, CF_3_), 127.0 (q, *J* = 4.0 Hz, 3CHAr), 127.8 (2CHAr), 128.7 (2CHAr), 129.4 (CAr), 137.3 (CAr), 161.2 (OCAr). HRMS C_18_H_21_F_3_NO_2_ [M + H]^+^ 340.1519; found 340.1523. Purity 100% (t_R_ = 2.74 min). 

#### 4.1.5. 1-(Dimethylamino)-3-[4-(trifluoromethyl)phenoxy]propan-2-ol (**3**)

Following the general procedure, dimethylamine (CAS 124-40-3) (0.04 mL, 0.55 mmol) and 2-{[4-(trifluoromethyl)phenoxy]methyl}oxirane (120 mg, 0.55 mmol) afforded **3** (48 mg, 33%) as an oil after a reversed-phase column chromatography (H_2_O:ACN 95:5). IR (ATR) 3348, 2926, 1615, 1520, 1461, 1257, 1033, 835, 775, 691 cm^−1^. ^1^H NMR (400 MHz, CDCl_3_, HETCOR) δ 2.66 (s, 6H, 2CH_3_), 2.84 (m, 1H, CH_2_N), 2.98 (m, 1H, CH_2_N), 4.00 (dd, *J* = 9.5, 5.5 Hz, 1H, CH_2_O), 4.10 (dd, *J* = 9.5, 5.0 Hz, 1H, CH_2_O), 4.34 (m, 1H, CH), 6.97 (d, *J* = 8.5 Hz, 2H, ArH), 7.53 (d, *J* = 9.0 Hz, 2H, ArH). ^13^C NMR (101 MHz, CDCl_3_) δ 45.1 (2CH_3_), 62.0 (CH_2_N), 65.2 (CH), 70.1 (CH_2_O), 114.6 (2CHAr), 121.6–124.3 (m, CF_3_), 125.8 (CAr), 127.1 (d, *J* = 4.0 Hz, 2CAr), 160.9 (OCAr). HRMS C_12_H_17_F_3_NO_2_ [M + H]^+^ 264.1206; found 264.1210. Purity 98.36% (t_R_ = 2.47 min).

#### 4.1.6. 1-[Benzyl(phenyl)amino]-3-[4-(trifluoromethyl)phenoxy]propan-2-ol (**4**)

Following the general procedure, *N*-benzylaniline (CAS 103-32-2) (0.08 mL, 0.46 mmol) and 2-{[4-(trifluoromethyl)phenoxy]methyl}oxirane (100 mg, 0.46 mmol) afforded **4** (95 mg, 52%) as a white solid after column chromatography (hexane/EtOAc 9:1 to 8:2). Mp 82–86 °C (EtOAc). IR (ATR) 3445, 1616, 1521, 1336, 1259, 1153, 1108, 1025, 839, 692 cm^−1^. ^1^H NMR (400 MHz, CDCl_3_, HETCOR) δ 2.36 (br s, 1H, OH), 3.63 (dd, *J* = 12.0, 4.5 Hz, 2H, CH_2_N), 4.00 (dd, *J* = 12.0, 4.5 Hz, 2H, CH_2_Ph), 4.33 (br s, 1H, CH), 4.61 (d, *J* = 5.5 Hz, 2H, CH_2_O), 6.73 (t, *J* = 7.0 Hz, 1H, ArH), 6.81 (d, *J* = 8.0 Hz, 2H, ArH), 6.91 (d, *J* = 8.5 Hz, 2H, ArH), 7.17–7.24 (m, 5H, ArH), 7.26–7.28 (m, 2H, ArH), 7.52 (d, *J* = 9.0 Hz, 2H, ArH). ^13^C NMR (101 MHz, CDCl_3_) δ 54.2 (CH_2_N), 55.8 (CH_2_Ph), 68.2 (CH), 70.1 (CH_2_O), 113.4 (2CHAr), 114.7 (2CHAr), 117.8 (2CHAr), 122.0–125.2 (m, CF_3_), 126.9 (2CHAr), 127.2 (CAr), 128.8 (3CAr), 129.5 (3CAr), 138.4 (CAr), 148.8 (CAr), 161.0 (OCAr). HRMS C_23_H_23_F_3_NO_2_ [M + H]^+^ 402.1675; found 402.1673. Purity 98.66% (t_R_ = 4.71 min).

#### 4.1.7. 1-[Benzyl(pyridin-3-ylmethyl)amino]-3-[4-(trifluoromethyl)phenoxy]propan-2-ol (**5**)

Following the general procedure, *N*-benzyl-1-(pyridin-3-yl)methanamine (CAS 63361-56-8) (73 mg, 0.37 mmol) and 2-{[4-(trifluoromethyl)phenoxy]methyl}oxirane (80 mg, 0.37 mmol) afforded **5** (40 mg, 26%) as a white solid after column chromatography (hexane/EtOAc 9:1 to 8:2). Mp 95–97 °C (EtOAc). IR (ATR) 2920, 1453, 1325, 1257, 1158, 1109, 1068, 1028, 837, 701 cm^−1^. ^1^H NMR (400 MHz, CDCl_3_, HETCOR) δ 2.66–2.77 (m, 2H, CH_2_N), 3.62 (dd, *J* = 10.5, 13.5 Hz, 2H, CH_2_Ph, CH_2_Pyr), 3.82 (dd, *J* = 13.5, 5.0 Hz, 2H, CH_2_Ph, CH_2_Pyr), 3.91 (d, *J* = 5.0 Hz, 2H, CH_2_O), 4.08–4.16 (m, 1H, CH), 6.88 (d, *J* = 8.5 Hz, 2H, ArH), 7.26–7.37 (m, 6H, ArH), 7.52 (d, *J* = 8.5 Hz, 2H, ArH), 7.67 (d, *J* = 8.0 Hz, 1H, ArH), 8.53 (d *J* = 4.0 Hz, 1H, ArH), 8.57 (s, 1H, ArH). ^13^C NMR (101 MHz, CDCl_3_) δ 56.1 (CH_2_N), 56.3 (CH_2_Pyr), 59.0 (CH_2_Ph), 66.7 (CH), 70.4 (CH_2_O), 114.6 (2CHAr), 123.0–123.6 (m, CF_3_), 123.7 (CHAr), 125.6 (CAr) 127.0 (2CHAr), 127.9 (CHAr), 128.8 (2CHAr), 129.3 (2CHAr), 133.8 (CAr), 137.0 (CHAr), 137.7 (CAr), 149.0 (CHAr), 150.4 (CHAr), 161.1 (CAr). HRMS C_23_H_24_F_3_N_2_O_2_ [M + H]^+^ 417.1784; found, 417.1794. Purity 100% (t_R_ = 2.43 min).

#### 4.1.8. 1-(Dibenzylamino)-3-phenoxypropan-2-ol (**6**)

Following the general procedure, dibenzylamine (CAS 103-49-1) (0.57 mL, 2.96 mmol) and 2-(phenoxymethyl)oxirane (442 mg, 2.94 mmol) afforded **6** (698 mg, 68%) as an oil after column chromatography (hexane/DCM 9:1 to 8:2). IR (ATR) 3434, 3028, 1598, 1494, 1452, 1242, 1927, 814, 747, 691 cm^−1^. ^1^H NMR (400 MHz, CDCl_3_, HETCOR) δ 2.61–2.73 (m, 2H, CH_2_N), 3.54 (d, *J* = 13.5 Hz, 2H, CH_2_Ph), 3.80 (d, *J* = 13.5 Hz, 2H, CH_2_Ph), 3.87 (d, *J* = 5.5 Hz, 2H, CH_2_O), 4.05–4.16 (m, 1H, CH), 6.84 (d, *J* = 8.0 Hz, 2H, ArH), 6.94 (t, *J* = 7.5 Hz, 1H, ArH), 7.21–7.38 (m, 12H, ArH). ^13^C NMR (101 MHz, CDCl_3_) δ 56.3 (CH_2_N), 58.9 (2CH_2_Ph), 66.6 (CH), 70.4 (CH_2_O), 114.6 (2CHAr), 121.0 (CHAr), 127.5 (2CHAr), 128.6 (4CHAr), 129.2 (4CHAr), 129.5 (2CHAr), 138.6 (2CAr), 158.8 (OCAr). HRMS C_23_H_26_NO_2_ [M + H]^+^ 348.1958; found 348.1961. Purity 98.28% (t_R_ = 3.16 min).

#### 4.1.9. 1-(4-Chlorophenoxy)-3-(dibenzylamino)propan-2-ol (**7**)

Following the general procedure, dibenzylamine (CAS 103-49-1) (0.33 mL, 1.71 mmol) and 2-[(4-chlorophenoxy)methyl]oxirane (316 mg, 1.71 mmol) afforded **7** (624 mg, 96%) as an oil after column chromatography (hexane/DCM 9:1 to 8:2). IR (ATR) 3426, 3027, 1589, 1485, 1446, 1248, 1027, 827, 743, 697 cm^−1^. ^1^H NMR (400 MHz, CDCl_3_, HETCOR) δ 2.75 (d, *J* = 6.5 Hz, 2H, CH_2_N), 3.57 (d, *J* = 13.5 Hz, 2H, CH_2_), 3.81 (d, *J* = 13.5 Hz, 2H, CH_2_Ph), 3.89–3.99 (m, 2H, CH_2_O), 4.13 (m, 1H, CH), 6.81–6.93 (m, 2H, ArH), 7.18 (m, 1H, ArH), 7.22–7.30 (m, 2H, ArH), 7.30–7.36 (m, 9H, ArH). ^13^C NMR (101 MHz, CDCl_3_) δ 56.1 (CH_2_N), 59.0 (2CH_2_Ph), 66.6 (CH), 71.3 (CH_2_O), 113.7 (2CHAr), 121.7 (2CHAr), 123.2 (CAr), 127.4 (2CHAr), 127.8 (2CHAr), 128.6 (2CHAr), 129.2 (2CHAr), 130.3 (2CHAr), 138.6 (2CAr), 154.3 (OCAr). HRMS C_23_H_25_ClNO_2_ [M + H]^+^ 382.1568; found 382.1570. Purity 96.90% (t_R_ = 3.27 min). 

#### 4.1.10. 1-(Dibenzylamino)-3-(p-tolyloxy)propan-2-ol (**8**)

Following the general procedure, dibenzylamine (CAS 103-49-1) (0.20 mL, 1.04 mmol) and 2-[(*p*-tolyloxy)methyl]oxirane (170 mg, 1.04 mmol) afforded **8** (300 mg, 80%) as a white solid after column chromatography (hexane/DCM 9:1 to 8:2). Mp 55–57 °C (EtOAc). IR (ATR) 3419, 3028, 2938, 2805, 1615, 1511, 1238, 1044, 803, 744, 697 cm^−1^. ^1^H NMR (400 MHz, CDCl_3_, HETCOR) δ 2.27 (s, 3H, CH_3_), 2.66 (d, *J* = 7.5 Hz, 2H, CH_2_N), 3.18 (s, 1H, OH), 3.53 (d, *J* = 13.5 Hz, 2H, CH_2_Ph), 3.79 (d, *J* = 13.5 Hz, 2H, CH_2_Ph), 3.84 (d, *J* = 5.5 Hz, 2H, CH_2_O), 4.04–4.14 (m, 1H, CH), 6.74 (d, *J* = 8.5 Hz, 2H, ArH), 7.04 (d, 2H, ArH), 7.32 (m, *J* = 3.0 Hz, 10H, ArH). ^13^C NMR (101 MHz, CDCl_3_) δ 20.6 (CH_3_), 56.3 (CH_2_N), 58.8 (2CH_2_Ph), 66.7 (CH), 70.6 (CH_2_O), 114.5 (2CHAr), 127.4 (2CHAr), 128.6 (4CHAr), 129.2 (4CHAr), 130.0 (CAr), 130.2 (2CHAr), 138.7 (2CAr), 156.7 (OCAr). HRMS C_24_H_28_NO_2_ [M + H]^+^ 362.2115; found 362.2115. Purity 97.23% (t_R_ = 3.35 min).

#### 4.1.11. 1-(Dibenzylamino)-3-(4-isopropylphenoxy)propan-2-ol (**9**)

Following the general procedure, dibenzylamine (CAS 103-49-1) (0.25 mL, 1.30 mmol) and 2-[(4-isopropylphenoxy)methyl]oxirane (253 mg, 1.32 mmol) afforded **9** (320 mg, 62%) as a white solid after column chromatography (hexane/DCM 9:1 to 8:2). Mp 70–72 °C (EtOAc). IR (ATR) 3449, 3027, 1609, 1513, 1455, 1245, 1039, 838, 745, 696 cm^−1^. ^1^H NMR (400 MHz, CDCl_3_, HETCOR) δ 1.21 (d, *J* = 7.0 Hz, 6H, CH_3_), 2.63–2.69 (m, 2H, CH_2_N), 2.85 (m, *J* = 7.0 Hz, 1H, CH), 3.17 (s, 1H, OH), 3.53 (d, *J* = 13.5 Hz, 2H, CH_2_Ph), 3.79 (d, *J* = 13.5 Hz, 2H, CH_2_Ph), 3.85 (d, *J* = 5.5 Hz, 2H, CH_2_O), 4.04–4.14 (m, 1H, CH), 6.77 (d, *J* = 9.0 Hz, 2H, ArH), 7.11 (d, *J* = 8.5 Hz, 2H, ArH), 7.25–7.36 (m, 10H, ArH). ^13^C NMR (101 MHz, CDCl_3_) δ 24.3 (2CH_3_), 33.4 (CH), 56.4 (CH_2_N), 58.9 (2CH_2_Ph), 66.7 (CH), 70.5 (CH_2_O), 114.4 (2CHAr), 127.3 (2CHAr), 127.4 (2CHAr), 128.6 (4CHAr), 129.2 (4CHAr), 138.7 (2CAr), 141.5 (CAr), 156.9 (OCAr). HRMS C_26_H_32_NO_2_ [M + H]^+^ 390.2428; found 390.2427. Purity 98.32% (t_R_ = 3.63 min).

#### 4.1.12. 1-[4-(Tert-butyl)phenoxy]-3-(dibenzylamino)propan-2-ol (**10**)

Following the general procedure, dibenzylamine (CAS 103-49-1) (0.24 mL, 1.25 mmol) and 2-{[4-(*tert*-butyl)phenoxy]methyl}oxirane (261 mg, 1.27 mmol) afforded **10** (375 mg, 74%) as a white solid after column chromatography (hexane/DCM 9:1 to 8:2). Mp 80–83 °C (EtOAc). IR (ATR) 3432, 3028, 2963, 1605, 1513, 1247, 1042, 828, 746, 698 cm^−1^. ^1^H NMR (400 MHz, CDCl_3_, HETCOR) δ 1.29 (s, 9H, 3CH_3_), 2.61–2.70 (m, 2H, CH_2_N), 3.16 (s, 1H, OH), 3.53 (d, *J* = 13.5 Hz, 2H, CH_2_Ph), 3.79 (d, *J* = 13.5 Hz, 2H, CH_2_Ph), 3.85 (d, *J* = 5.5 Hz, 2H, CH_2_O), 4.05–4.12 (m, 1H, CH), 6.78 (d, *J* = 9.0 Hz, 2H, ArH), 7.24 (m, 2H, ArH), 7.26–7.35 (m, 10H, ArH). ^13^C NMR (101 MHz, CDCl_3_) δ 31.7 (3CH_3_), 34.2 (C), 56.4 (CH_2_N), 58.9 (2CH_2_Ph), 66.7 (CH), 70.5 (CH_2_O), 114.1 (2CHAr), 126.3 (2CHAr), 127.4 (2CHAr), 128.6 (4CHAr), 129.2 (4CHAr), 138.7 (2CAr), 143.7 (CAr), 156.5 (OCAr). HRMS C_27_H_34_NO_2_ [M + H]^+^ 404.2584; found 404.2590. Purity 100% (t_R_ = 3.62 min).

#### 4.1.13. 1-(4-Cyclohexylphenoxy)-3-(dibenzylamino)propan-2-ol (**11**)

Following the general procedure, dibenzylamine (CAS 103-49-1) (0.21 mL, 1.09 mmol) and 2-[(4-cyclohexylphenoxy)methyl]oxirane (253 mg, 1.09 mmol) afforded **11** (340 mg, 73%) as a white solid after column chromatography (hexane/DCM 9:1 to 8:2). Mp 113–115 °C (EtOAc). IR (ATR) 3427, 3028, 2928, 1512, 1515, 1248, 1047, 809, 744, 696 cm^−1^. ^1^H NMR (400 MHz, CDCl_3_, HETCOR) δ 1.18–1.31 (m, 1H, CH_2_cycl), 1.31–1.46 (m, 4H, 2CH_2_cycl), 1.75 (m, 1H, CH_2_cycl), 1.78–1.91 (m, 4H, 2CH_2_cycl), 2.39–2.51 (m, 1H, CHcycl), 2.63–2.75 (m, 2H, CH_2_N), 3.18 (s, 1H, OH), 3.55 (d, *J* = 13.5 Hz, 2H, CH_2_Ph), 3.80 (d, *J* = 13.5 Hz, 2H, CH_2_ Ph), 3.86 (d, *J* = 5.5 Hz, 2H, CH_2_O), 4.05–4.15 (m, 1H, CH), 6.78 (d, *J* = 9.8 Hz, 2H, ArH), 7.11 (d, *J* = 6.5 Hz, 2H, ArH), 7.24–7.31 (m, 2H, ArH), 7.30–7.39 (m, 8H, ArH). ^13^C NMR (101 MHz, CDCl_3_) 26.3 (CH_2_cycl), 27.1 (2CH_2_cycl), 34.8 (2CH_2_cycl), 43.8 (CHcycl), 56.4 (CH_2_N), 58.9 (2CH_2_Ph), 66.7 (CH), 70.5 (CH_2_O), 114.4 (2CHAr), 127.4 (2CHAr), 127.7 (2CHAr), 128.6 (4CHAr), 129.2 (4CHAr), 138.7 (2CAr), 140.8 (CAr), 156.9 (OCAr). HRMS C_29_H_36_NO_2_ [M + H]^+^ 430.2741; found 430.2736. Purity 99.24% (t_R_ = 3.88 min).

#### 4.1.14. 1-(4-Cyclopentylphenoxy)-3-(dibenzylamino)propan-2-ol (**12**)

Following the general procedure, dibenzylamine (CAS 103-49-1) (0.23 mL, 1.19 mmol) and 2-[(4-cyclopentylphenoxy)methyl]oxirane (257 mg, 1.18 mmol) afforded **12** (295 mg, 60%) as a white solid after column chromatography (hexane/DCM 9:1 to 8:2). Mp 88–90 °C (EtOAc). IR (ATR) 3413, 2938, 1610, 1511, 1451, 1242, 1044, 824, 747, 698 cm^−1^. ^1^H NMR (400 MHz, CDCl_3_, HETCOR) δ1.48–1.60 (m, 2H, CH_2_cycl), 1.62–1.71 (m, 2H, CH_2_cycl), 1.73–1.83 (m, 2H, CH_2_cycl), 1.96–2.09 (m, 2H, CH_2_cycl), 2.63–2.69 (m, 2H, CH_2_N), 2.86–2.99 (m, 1H, CHcycl), 3.53 (d, *J* = 13.5 Hz, 2H, CH_2_Ph), 3.79 (d, *J* = 13.5 Hz, 2H, CH_2_Ph), 3.84 (d, *J* = 5.5 Hz, 2H, CH_2_O), 4.05–4.11 (m, 1H, CH), 6.73–6.80 (d, 2H, ArH), 7.09–7.16 (d, 2H, ArH), 7.22–7.37 (m, 10H, ArH). ^13^C NMR (101 MHz, CDCl_3_) δ 25.5 (2CH_2_cycl), 34.8 (2CH_2_cycl), 45.3 (CHcycl), 56.3 (CH_2_), 58.9 (2CH_2_Ph), 66.7 (CH), 70.6 (CH_2_), 114.4 (2CHAr), 127.4 (2CHAr), 128.0 (2CHAr), 128.6 (4CHAr), 129.2 (4CHAr), 138.7 (CAr), 139.0 (2CAr), 156.9 (OCAr). HRMS C_28_H_34_NO_2_ [M + H]^+^ 416.2584; found 416.2591. Purity 100% (t_R_ = 3.66 min).

#### 4.1.15. 1-(Dibenzylamino)-3-(3,4-dimethylphenoxy)propan-2-ol (**13**)

Following the general procedure, dibenzylamine (CAS 103-49-1) (0.37 mL, 1.92 mmol) and 2-[(3,4-dimethylphenoxy)methyl]oxirane (347 mg, 1.95 mmol) afforded **13** (555 mg, 76%) as an oil after column chromatography (hexane/DCM 9:1 to 8:2). IR (ATR) 3435, 3026, 2921, 1607, 1501, 1452, 1252, 1046, 735, 697 cm^−1^. ^1^H NMR (400 MHz, CDCl_3_, HETCOR) δ 2.18 (s, 3H, CH_3_), 2.21 (s, 3H, CH_3_), 2.62–2.73 (m, 2H, CH_2_N), 3.54 (d, *J* = 13.5 Hz, 2H, CH_2_Ph), 3.79 (d, *J* = 13.5 Hz, 2H, CH_2_Ph), 3.83 (d, *J* = 5.0 Hz, 2H, CH_2_O), 4.02–4.14 (m, 1H, CH), 6.58 (dd, *J* = 8.5, 3.0 Hz, 1H, ArH), 6.65 (d, *J* = 3.0 Hz, 1H, ArH), 7.00 (d, *J* = 8.3 Hz, 1H, ArH), 7.21–7.42 (m, 10H, ArH). ^13^C NMR (101 MHz, CDCl_3_) δ 18.9 (CH_3_), 20.1 (CH_3_), 56.3 (CH_2_N), 58.8 (2CH_2_Ph), 66.7 (CH), 70.6 (CH_2_O), 111.6 (CHAr), 116.2 (CHAr), 127.4 (2CHAr), 128.6 (4CHAr), 129.0 (CHAr), 129.2 (4CHAr), 130.4 (CAr), 137.8 (CAr), 138.7 (2CAr), 157.0 (OCAr). HRMS C_25_H_30_NO_2_ [M + H]^+^ 376.2271; found 376.2270. Purity 97.06% (t_R_ = 3.35 min).

#### 4.1.16. 1-(Dibenzylamino)-3-(4-methoxyphenoxy)propan-2-ol (**14**)

Following the general procedure, dibenzylamine (CAS 103-49-1) (0.13 mL, 0.70 mmol) and 2-[(4-methoxyphenoxy)methyl]oxirane (126 mg, 0.70 mmol) afforded **14** (170 mg, 64%) as a white solid after column chromatography (hexane/DCM 9:1 to 8:2). Mp 61–63 °C (EtOAc). IR (ATR) 3417, 2925, 1505, 1451, 1231, 1044, 1028, 822, 744, 697 cm^−1^. ^1^H NMR (400 MHz, CDCl_3_, HETCOR) δ 2.67 (d, *J* = 7.5 Hz, 2H, CH_2_N), 3.54 (d, *J* = 13.5 Hz, 2H, CH_2_Ph), 3.76 (s, 3H, CH_3_), 3.77 (d, *J* = 13.5 Hz, 2H, CH_2_Ph), 3.78–3.85 (m, 2H, CH_2_O), 4.08 (m, 1H, CH), 6.75–6.83 (m, 4H, ArH), 7.23–7.37 (m, 10H, ArH). ^13^C NMR (101 MHz, CDCl_3_) δ 55.9 (CH_3_), 56.3 (CH_2_N), 58.9 (2CH_2_Ph), 66.7 (CH), 71.2 (CH_2_O), 114.7 (2CHAr), 115.6 (2CHAr), 127.4 (2CHAr), 128.6 (4CHAr), 129.2 (4CHAr), 138.7 (2CAr), 153.0 (CAr), 154.1 (OCAr). HRMS C_24_H_28_NO_3_ [M + H]^+^ 378.2064; found 378.2062. Purity 98.43% (t_R_ = 3.02 min).

#### 4.1.17. 1-[4-(Benzyloxy)phenoxy]-3-(dibenzylamino)propan-2-ol (**15**)

Following the general procedure, dibenzylamine (CAS 103-49-1) (0.19 mL, 0.99 mmol) and 2-{[4-(benzyloxy)phenoxy]methyl}oxirane (247 mg, 0.96 mmol) afforded **15** (325 mg, 74%) as a white solid after column chromatography (hexane/DCM 9:1 to 8:2). Mp 87–88 °C (EtOAc). IR (ATR) 3454, 3027, 2795, 1602, 1509, 1235, 1041, 825, 731, 696 cm^−1^. ^1^H NMR (400 MHz, CDCl_3_, HETCOR) δ 2.63–2.68 (m, 2H, CH_2_N), 3.17 (s, 1H, OH), 3.53 (d, *J* = 13.5 Hz, 2H, CH_2_Ph), 3.75–3.84 (m, 4H, 2CH_2_Ph), 4.07 (m, *J* = 5.8 Hz, 1H, CH), 5.00 (s, 2H, PhCH_2_O), 6.73–6.80 (m, 2H, ArH), 6.83–6.90 (m, 2H, ArH), 7.22–7.44 (m, 15H, ArH). ^13^C NMR (101 MHz, CDCl_3_) δ 56.3 (CH_2_N), 58.9 (2CH_2_Ph), 66.7 (CH), 70.8 (PhCH_2_O), 71.1 (CH_2_O), 115.5 (2CHAr), 115.9 (2CHAr), 127.4 (2CHAr), 127.6 (2CHAr), 128.0 (CHAr), 128.6 (4CHAr), 128.7 (2CHAr), 129.2 (4CHAr), 137.4 (2CAr), 138.7 (CAr), 153.1 (CAr), 153.2 (OCAr). HRMS C_30_H_32_NO_3_ [M + H]^+^ 454.2377; found 454.2385. Purity 96.19% (t_R_ = 3.43 min).

#### 4.1.18. 1-(Dibenzylamino)-3-(4-propoxyphenoxy)propan-2-ol (**16**)

Following the general procedure, dibenzylamine (CAS 103-49-1) (0.46 mL, 2.38 mmol) and 2-[(4-propoxyphenoxy)methyl]oxirane (490 mg, 2.35 mmol) afforded **16** (620 mg, 65%) as a white solid after column chromatography (hexane/DCM 9:1 to 8:2). Mp 51–53 °C (EtOAc). IR (ATR) 3380, 3027, 2921, 1589, 1508, 1228, 1048, 827, 747, 697 cm^−1^. ^1^H NMR (400 MHz, CDCl_3_, HETCOR) δ 1.02 (t, *J* = 7.5 Hz, 3H, CH_3_), 1.71–1.84 (m, 2H, OCH_2_C*H*_2_), 2.63–2.69 (m, 2H, CH_2_N), 3.54 (d, *J* = 13.5 Hz, 2H, CH_2_Ph), 3.80 (d, *J* = 13.5 Hz, 2H, CH_2_Ph), 3.86 (t, *J* = 6.4 Hz, 2H, OC*H*_2_), 4.08 (m, 1H, CH), 6.73–6.84 (m, 4H, ArH), 7.26–7.37 (m, 10H, ArH).^13^C NMR (101 MHz, CDCl_3_) δ 10.7 (CH_3_), 22.8 (OCH_2_*C*H_2_), 56.3 (CH_2_N), 58.9 (2CH_2_Ph), 66.7 (CH), 70.3 (CH_2_O), 71.2 (OCH_2_), 115.5 (2CHAr), 115.5 (2CHAr), 127.4 (2CHAr), 128.6 (4CHAr), 129.2 (4CHAr), 138.7 (2CAr), 152.9 (CAr), 153.6 (OCAr). HRMS C_26_H_32_NO_3_ [M + H]^+^ 406.2377; found 406.2379. Purity 98.33% (t_R_ = 3.33 min).

#### 4.1.19. 1-(Dibenzylamino)-3-(3,4-dichlorophenoxy)propan-2-ol (**17**)

Following the general procedure, dibenzylamine (CAS 103-49-1) (0.45 mL, 2.35 mmol) and 2-[(3,4-dichlorophenoxy)methyl]oxirane (510 mg, 2.33 mmol) afforded **17** (882 mg, 91%) as an oil after column chromatography (hexane/DCM 9:1 to 8:2). IR (ATR) 3425, 3027, 2931, 1592, 1451, 1230, 1026, 839, 735, 698 cm^−1^. ^1^H NMR (400 MHz, CDCl_3_, HETCOR) δ 2.61–2.67 (m, 2H, CH_2_N), 3.17 (s, 1H, OH), 3.53 (d, *J* = 13.0 Hz, 2H, CH_2_Ph), 3.73–3.78 (m, 4H, CH_2_Ph_,_ CH_2_O), 4.03 (m, 1H, CH), 6.67 (dd, *J* = 9.0, 3.0 Hz, 1H, ArH), 6.89 (d, *J* = 3.0 Hz, 1H, ArH), 7.22–7.28 (m, 1H, ArH), 7.28–7.36 (m, 10H, ArH). ^13^C NMR (101 MHz, CDCl_3_) δ 55.9 (CH_2_N), 59.0 (2CH_2_Ph), 66.4 (CH), 71.0 (CH_2_O), 114.7 (CHAr), 116.5 (CHAr), 124.3 (CAr), 127.6 (2CHAr), 128.6 (4CHAr), 129.2 (4CHAr), 130.7 (CHAr), 132.9 (CAr), 138.5 (2CAr), 157.9 (OCAr). HRMS C_23_H_24_Cl_2_NO_2_ [M + H]^+^ 416.1179; found 416.1176. Purity 96.24% (t_R_ = 3.59 min).

#### 4.1.20. 1-[4-Chloro-3-(trifluoromethyl)phenoxy]-3-(dibenzylamino)propan-2-ol (**18**)

Following the general procedure, dibenzylamine (CAS 103-49-1) (0.40 mL, 2.10 mmol) and 2-{[4-chloro-3-(trifluoromethyl)phenoxy]methyl}oxirane (529 mg, 2.10 mmol) afforded **18** (878 mg, 93%) as an oil after column chromatography (hexane/DCM 9:1 to 8:2). IR (ATR) 3433, 3028, 1606, 1482, 1423, 1239, 1027, 816, 747, 698 cm^−1^. ^1^H NMR (400 MHz, CDCl_3_, HETCOR) δ 2.63–2.69 (m, 2H, CH_2_N), 3.20 (s, 1H, OH), 3.54 (d, *J* = 13.0 Hz, 2H, CH_2_Ph), 3.85 (m, 2H_,_ CH_2_O), 4.04 (m, 1H, CH), 6.90 (dd, *J* = 9.0, 3.0 Hz, 1H, ArH), 7.11 (d, *J* = 3.0 Hz, 1H, ArH), 7.22–7.38 (m, 11H, ArH). ^13^C NMR (101 MHz, CDCl_3_) δ 55.8 (CH_2_N), 59.0 (2CH_2_Ph), 66.4 (CH), 70.9 (CH_2_O), 114.2 (q, *J* = 5.5 Hz, CHAr), 118.7 (CHAr), 121.4 (CAr), 123.5 (CF_3_), 124.1 (CAr), 127.6 (2CHAr), 128.6 (4CHAr), 129.2 (4CHAr), 132.4 (CHAr), 138.5 (2CAr), 157.2 (OCAr). HRMS C_24_H_24_ClF_3_NO_2_ [M + H]^+^ 450.1442; found 450.1448. Purity 99.79% (t_R_ = 3.72 min).

#### 4.1.21. 1-(Dibenzylamino)-3-[4-nitro-3-(trifluoromethyl)phenoxy]propan-2-ol (**19**)

Following the general procedure, dibenzylamine (CAS 103-49-1) (0.45 mL, 2.34 mmol) and 2-{[4-nitro-3-(trifluoromethyl)phenoxy]methyl}oxirane (609 mg, 2.31 mmol) afforded **19** (619 mg, 58%) as an oil after column chromatography (hexane/DCM 9:1 to 8:2). IR (ATR) 3424, 2931, 1453, 1310, 1242, 1038, 1027, 834, 748, 698 cm^−1^. ^1^H NMR (400 MHz, CDCl_3_, HETCOR) δ 1H NMR (400 MHz, CDCl3) δ 2.56–2.75 (m, 2H, CH_2_N), 3.55 (d, *J* = 13.0 Hz, 2H, CH_2_Ph), 3.80 (d, *J* = 13.0 Hz, 2H, CH_2_Ph), 3.88–4.01 (m, 2H, CH_2_O), 4.01–4.10 (m, 1H, CH), 7.01 (dd, *J* = 9.0, 3.0 Hz, 1H, ArH), 7.18 (d, *J* = 2.5 Hz, 1H, ArH), 7.23–7.43 (m, 10H, ArH), 7.95 (d, *J* = 9.5 Hz, 1H, ArH). ^13^C NMR (101 MHz, CDCl_3_) δ 55.5 (CH_2_N), 59.1 (2CH_2_Ph), 66.3 (CH), 71.3 (CH_2_O), 115.0 (q, *J* = 6.0 Hz, CF_3_), 116.8 (CHAr), 120.6 (CHAr), 123.3 (CAr), 126.2 (q, *J* = 34.0 Hz, CF_3_), 127.6 (2CAr), 128.1 (CAr), 128.7 (4CAr), 129.2 (4CAr), 138.4 (2CAr), 141.1 (CAr), 161.9 (OCAr). HRMS C_24_H_24_F_3_N_2_O_4_ [M + H]^+^ 461.1683; found 461.1681. Purity 98.68% (t_R_ = 3.50 min).

#### 4.1.22. 1-(Dibenzylamino)-3-(2,4-dichlorophenoxy)propan-2-ol (**20**)

Following the general procedure, dibenzylamine (CAS 103-49-1) (0.48 mL, 2.49 mmol) and 2-[(2,4-dichlorophenoxy)methyl]oxirane (551 mg, 2.52 mmol) afforded **20** (642 mg, 61%) as an oil after column chromatography (hexane/DCM 9:1 to 8:2). IR (ATR) 3426, 3027, 2803, 1585, 1482, 1290, 1062, 802, 744, 697 cm^−1^. ^1^H NMR (400 MHz, CDCl_3_, HETCOR) δ 2.64–2.70 (m, 2H, CH_2_N), 3.49 (d, *J* = 13.5 Hz, 2H, CH_2_Ph), 3.74 (d, *J* = 13.5 Hz, 2H, CH_2_Ph), 3.79–3.90 (m, 2H, CH_2_O), 3.99–4.08 (m, 1H, CH), 6.69 (d, *J* = 9.0 Hz, 1H, ArH), 7.08 (dd, *J* = 9.0, 2.5 Hz, 1H, ArH), 7.16–7.31 (m, 11H, ArH). ^13^C NMR (101 MHz, CDCl_3_) δ 55.9 (CH_2_N), 59.0 (2CH_2_Ph), 66.5 (CH), 71.6 (CH_2_O), 114.4 (CHAr), 124.0 (CHAr), 126.1 (2CHAr), 127.5 (4CHAr), 127.6 (CAr), 128.6 (4CHAr), 129.2 (2CHAr), 130.0 (CAr), 138.6 (2CAr), 153.2 (OCAr). HRMS C_23_H_24_Cl_2_NO_2_ [M + H]^+^ 416.1179; found 416.1179. Purity 95.03% (t_R_ = 3.58 min).

#### 4.1.23. 1-(4-Bromophenoxy)-3-(dibenzylamino)propan-2-ol (**21**)

Following the general procedure, dibenzylamine (CAS 103-49-1) (0.42 mL, 2.21 mmol) and 2-[(4-bromophenoxy)methyl]oxirane (500 mg, 2.18 mmol) afforded **21** (800 mg, 86%) as a white solid after column chromatography (hexane/EtOAc 9:1 to 8:2). Mp 63–65 °C (EtOAc). IR (ATR) 3568, 3413, 3031, 2936, 1587, 1488, 1243, 1036, 830, 804, 744, 733, 698 cm^−1^. ^1^H NMR (500 MHz, CDCl_3_, HETCOR) δ 2.66 (d, *J* = 7.0 Hz, 2H, CH_2_N), 3.19 (s, 1H, OH), 3.54 (d, *J* = 13.5 Hz, 2H, CH_2_Ph), 3.80 (d, *J* = 13.5 Hz, 2H, CH_2_Ph), 3.83 (m, 2H, CH_2_O), 4.03–4.14 (m, 1H, CH), 6.68–6.74 (m, 2H, ArH), 7.23–7.30 (m, 2H, ArH), 7.30–7.42 (m, 10H, ArH). ^13^C NMR (101 MHz, CDCl_3_) δ 56.1 (CH_2_N), 58.9 (2CH_2_Ph), 66.5 (CH), 70.7 (CH_2_O), 113.2 (CAr), 116.5 (2CHAr), 127.5 (2CHAr), 128.6 (4CHAr), 129.2 (4CHAr), 132.3 (2CHAr), 138.6 (2CAr), 157.9 (OCAr). HRMS C_23_H_25_BrNO_2_ [M + H]^+^ 426.1063; found 426.1064. Purity 97.85% (t_R_ = 4.17 min). 

#### 4.1.24. 1-(Dibenzylamino)-3-(4-nitrophenoxy)propan-2-ol (**22**)

Following the general procedure, dibenzylamine (CAS 103-49-1) (0.45 mL, 2.33 mmol) and 2-[(4-nitrophenoxy)methyl]oxirane (450 mg, 2.31 mmol) afforded **22** (610 mg, 67.4%) as an oil after column chromatography (hexane/EtOAc 9:1 to 8:2). IR (ATR) 3427, 3028, 2931, 1592, 1509, 1496, 1331, 1259, 1109, 1026, 842, 750, 697 cm^−1^. ^1^H NMR (500 MHz, CDCl_3_, HETCOR) δ 2.60–2.74 (m, 2H, CH_2_N), 3.25 (s, 1H, OH), 3.55 (d, *J* = 13.5 Hz, 2H, CH_2_Ph), 3.82 (d, *J* = 13.5 Hz, 2H, CH_2_Ph), 3.91 (dd, *J* = 10, 4.5 Hz, 1H, CH_2_O), 3.95 (dd, *J* = 10, 4.5 Hz, 1H, CH_2_O), 4.08(m, 1H, CH), 6.87 (m, 2H, ArH), 7.23 -7.41 (m, 10H, ArH), 8.11–8.22 (m, 2H, ArH). ^13^C NMR (126 MHz, CDCl_3_) δ 55.8 (CH_2_N), 59.0 (2CH_2_Ph), 66.3 (CH), 71.1 (CH_2_O), 114.7 (CHAr), 126.0 (2CHAr), 127.6 (2CHAr), 128.7 (4CHAr), 129.2 (4CHAr), 138.5 (2CAr), 141.8 (CAr), 163.8 (OCAr). HRMS C_23_H_24_N_2_O_4_ [M + H]^+^ 393.1809; found 393.1814. Purity 96.42% (t_R_ = 4 min).

#### 4.1.25. 1-(Dibenzylamino)-3-(4-iodophenoxy)propan-2-ol (**23**)

Following the general procedure, dibenzylamine (CAS 103-49-1) (0.33 mL, 1.7 mmol) and 2-[(4-iodophenoxy)methyl]oxirane (465 mg, 1.68 mmol) afforded **23** (525 mg, 65.8%) as a white solid after column chromatography (hexane/EtOAc 9:1 to 8:2). Mp 103–105 °C (EtOAc). IR (ATR) 3406, 3030, 2936, 1580, 1484, 1282, 1246, 1035, 828, 803, 744, 697 cm^−1^. ^1^H NMR (500 MHz, CDCl_3_, HETCOR) *δ* 2.66 (d, *J* = 7.0 Hz, 2H, CH_2_N), 3.19 (s, 1H, OH), 3.54 (d, *J* = 13.5 Hz, 2H, CH_2_Ph), 3.79 (d, *J* = 13.5 Hz, 2H, CH_2_Ph), 3.74–3.87 (m, 2H, CH_2_O), 4.07 (m, 1H, CH), 6.58–6.64 (m, 2H, ArH), 7.19–7.38 (m, 10H, ArH), 7.49–7.56 (m, 2H, ArH). ^13^C NMR (126 MHz, CDCl_3_) δ 56.1 (CH_2_N), 58.9 (2CH_2_Ph), 66.5 (CH), 70.5 (CH_2_O), 83.1 (CAr), 117.1 (2CHAr), 127.5 (2CHAr), 128.6 (4CHAr), 129.2 (4CHAr), 138.3 (2CAr), 138.6 (2CHAr), 158.7 (CAr). HRMS C_23_H_24_INO_2_ [M + H]^+^ 474.0924; found 474.0927. Purity 96.40% (t_R_ = 4.26 min). 

#### 4.1.26. 1-(Dibenzylamino)-3-[4-(pentafluoro-λ6-sulfaneyl)phenoxy]propan-2-ol (**24**)

Following the general procedure, dibenzylamine (CAS 103-49-1) (0.45 mL, 2.33 mmol) and 2-[(4-(pentafluoro-λ6-sulfaneyl)phenoxy)methyl]oxirane (643 mg, 2.33 mmol) afforded **24** (657 mg, 59.6%) as a white solid after column chromatography (hexane/EtOAc 9:1 to 8:2). Mp 67–69⁰C (EtOAc). IR (ATR) 3414, 3028, 2938, 1597, 1504, 1451, 1309, 1264, 1101, 1037, 844, 826, 806, 745, 698, 595, 579 cm^−1^. ^1^H NMR (500 MHz, CDCl_3_, HETCOR) δ 2.67 (d, *J* = 6.0 Hz, 2H, CH_2_N), 3.21 (s, 1H, OH), 3.54 (d, *J* = 13.5 Hz, 2H, CH_2_Ph), 3.81 (d, *J* = 13.5 Hz, 2H, CH_2_Ph), 3.85–3.94 (m, 2H, CH_2_O), 4.03–4.10 (m, 1H, CH), 6.83 (d, *J* = 9.5 Hz, 2ArH), 7.19–7.38 (m, 10H, ArH), 7.61–7.68 (m, 2H, ArH).^13^C NMR (126 MHz, CDCl_3_) δ 56.0 (CH_2_N), 59.0 (2CH_2_Ph), 66.4 (CH), 70.8 (CH_2_O), 114.2 (2CHAr), 127.6 (4CHAr), 127.7 (m, C-SF_5_), 128.7 (4CHAr), 129.2 (4CHAr), 138.5 (2CAr), 160.6 (OCAr). HRMS C_23_H_24_F_5_NO_2_S [M + H]^+^ 474.1521; found 474.1524. Purity 95.25% (t_R_ = 4.36 min). 

#### 4.1.27. 1-[Benzyl(4-chlorobenzyl)amino]-3-(4-chlorophenoxy)propan-2-ol (**25**)

Following the general procedure, *N*-benzyl-1-(4-chlorophenyl)methanamine (CAS 13541-00-9) (0.14 mL, 0.70 mmol) and 2-[(4-chlorophenoxy)methyl]oxirane (130 mg, 0.70 mmol) afforded **25** (182.5 mg, 62.3%) as an oil after column chromatography (hexane/EtOAc 9:1 to 8:2). IR (ATR) 3426, 3026, 2932, 1590, 1485, 1445, 1276, 1248, 1087, 1063, 1015, 801, 743, 697 cm^−1^. ^1^H NMR (400 MHz, CDCl_3_, HETCOR) *δ* 2.72 (dd, *J* = 6.5, 2.0 Hz, 2H, CH_2_N), 3.54 (dd, *J* = 13.5, 11 Hz, 2H, CH_2_Ph), 3.74 (t, *J* = 13.5 Hz, 2H, CH_2_Ph), 3.90–3.95 (m, 2H, CH_2_O), 4.06–4.13 (m, 1H, CH), 6.83 (dd, *J* = 8.5, 1.5 Hz, 1H, ArH), 6.88 (td, *J* = 7.5, 1.5 Hz, 1H, ArH), 7.18 (ddd, *J* = 8.5, 7.5, 1.5 Hz, 1H, ArH), 7.22–7.36 (m, 10H, ArH). ^13^C NMR (101 MHz, CDCl_3_) δ 56.1 (CH_2_N), 58.3 (CH_2_Ph), 59.0 (CH_2_Ph), 66.8 (CH), 71.2 (CH_2_O), 113.6 (CHAr), 121.8 (CHAr), 123.2 (CAr), 127.5 (CHAr), 127.8 (CHAr), 128.6 (2CHAr), 128.7 (2CHAr), 129.2 (2CHAr), 130.4 (CHAr), 130.5 (2CHAr), 133.2 (CAr), 137.3 (CAr), 138.5 (CAr), 154.2 (CAr). HRMS C_23_H_23_Cl_2_NO_2_ [M + H]^+^ 416.1179; found 4161191. Purity 95.59% (t_R_ = 4.45 min). 

#### 4.1.28. 1-[Bis(4-chlorobenzyl)amino]-3-(4-chlorophenoxy)propan-2-ol (**26**)

Following the general procedure, bis(4-chlorobenzyl)amine (CAS 21913-13-3)(150 mg, 0.56 mmol) and 2-[(4-chlorophenoxy)methyl]oxirane (104 mg, 0.56 mmol) afforded **26** (194.2 mg, 76.4%) as an oil after column chromatography (hexane/EtOAc 9:1 to 8:2). IR (ATR) 3438, 3058, 2921, 1740, 1589, 1486, 1447, 1248, 1087, 1062, 1014, 807, 746 cm^−1^. ^1^H NMR (400 MHz, CDCl_3_) δ 2.63–2.77 (m, 2H, CH_2_N), 2.96 (s, 1H, OH), 3.52 (d, *J* = 13.5 Hz, 2H, CH_2_Ph), 3.69 (d, *J* = 13.5 Hz, 2H, CH_2_Ph), 3.90–3.94 (m, 2H, CH_2_O), 4.06–4.14 (m, 1H, CH), 6.82 (dd, *J* = 8.5, 1.5 Hz, 1H, ArH), 6.89 (td, *J* = 7.5, 1.5 Hz, 1H, ArH), 7.15–7.29 (m, 9H, ArH), 7.33 (dd, *J* = 8.0, 1.5 Hz, 1H, ArH). ^13^C NMR (101 MHz, CDCl_3_) δ 56.0 (CH_2_N), 58.3 (2CH_2_Ph), 66.9 (CH), 71.0 (CH_2_O), 113.6 (CHAr), 121.9 (CHAr), 123.1 (CAr), 127.9 (CHAr), 128.8 (4CHAr), 130.4 (CHAr), 130.4 (4CHAr), 133.3 (2CAr), 137.1 (2CAr), 154.1 (CAr). HRMS C_23_H_22_Cl_3_NO_2_ [M + H]^+^ 450.0789; found 450.9787. Purity 99.45% (t_R_ = 5.24 min).

#### 4.1.29. 1-[Benzyl(4-methylbenzyl)amino]-3-(4-chlorophenoxy)propan-2-ol (**27**)

Following the general procedure, *N*-benzyl-1-(*p*-tolyl)methanamine (CAS 55096-86-1) (148.8 mg, 0.70 mmol) and 2-[(4-chlorophenoxy)methyl]oxirane (130 mg, 0.70 mmol) afforded **27** (119,5 mg, 42.9%) as an oil after column chromatography (hexane/EtOAc 9:1 to 8:2). IR (ATR) 3436, 3028, 2924, 1589, 1485, 1446, 1277, 1249, 1061, 1028, 801, 744, 697 cm^−1^. ^1^H NMR (400 MHz, CDCl_3_, HETCOR) *δ* 2.33 (s, 3H, CH_3_), 2.73 (d, *J* = 6.5 Hz, 2H, CH_2_N), 3.53 (t, *J* = 13.0 Hz, 2H, CH_2_Ph), 3.80 (t, *J* = 14.0 Hz, 2H, CH_2_Ph), 3.91–4.00 (m, 2H, CH_2_O), 4.11 (m, 1H, CH), 6.84 (dd, *J* = 8.0, 1.5 Hz, 1H, ArH), 6.89 (td, *J* = 7.5, 1.5 Hz, 1H, ArH), 7.11–7.38 (m, 11H, ArH). ^13^C NMR (101 MHz, CDCl_3_) δ 21.3 (CH_3_), 56.0 (CH_2_N), 58.6 (CH_2_Ph), 58.9 (CH_2_Ph), 66.6 (CH), 71.3 (CH_2_O), 113.6 (CHAr), 121.7 (CHAr), 123.2 (CAr), 127.4 (CHAr), 127.8 (CHAr), 128.6 (4CHAr), 129.1–129.3 (4CHAr), 130.3 (CHAr), 135.5 (CAr), 137.0 (CAr), 138.8 (CAr), 154.3 (CAr). HRMS C_24_H_26_ClNO_2_ [M + H]^+^ 396.1729; found 396.1725. Purity 95.30% (t_R_ = 4.12 min).

#### 4.1.30. 1-[Bis(4-methylbenzyl)amino]-3-(4-chlorophenoxy)propan-2-ol (**28**)

Following the general procedure, bis(4-methylbenzyl)amine (CAS 98180-43-9) (100.0 mg, 0.43 mmol) and 2-[(4-chlorophenoxy)methyl]oxirane (81.9 mg, 0.48 mmol) afforded **28** (219.8 mg, 87.3%) as an oil after column chromatography (hexane/EtOAc 9:1 to 8:2). IR (ATR) 2920, 2843, 1590, 1510, 1485, 1445, 1278, 1253, 1061, 1036, 807, 741 cm^−1^. ^1^H NMR (400 MHz, CDCl_3_) δ 2.33 (s, 6H, 2CH_3_), 2.72 (d, *J* = 6.5 Hz, 2H, CH_2_N), 3.51 (d, *J* = 13.0 Hz, 2H, CH_2_Ph), 3.76 (d, *J* = 13.0 Hz, 2H, CH_2_Ph), 3.93 (d, *J* = 5.0 Hz, 2H, CH_2_O), 4.10 (m, 1H, CH), 6.84 (dd, *J* = 8.0, 1.5 Hz, 1H, ArH), 6.88 (td, *J* = 7.5, 1.5 Hz, 1H, ArH), 7.10–7.15 (m, 4H, ArH), 7.16–7.18 (m, 1H, ArH), 7.18–7.23 (m, 4H, ArH), 7.33 (dd, *J* = 8.0, 1.5 Hz, 1H, ArH). ^13^C NMR (101 MHz, CDCl_3_) δ 21.3 (2CH_3_), 55.9 (CH_2_N), 58.57 (2CH_2_Ph), 66.5 (CH), 71.25 (CH_2_O), 113.61 (2CHAr), 121.67 (2CHAr), 123.17 (CAr), 127.7 (2CHAr), 129.2 (2CHAr), 130.3 (4CHAr), 135.6 (2CAr), 137.0 (2CAr), 154.3 (CAr). HRMS C_25_H_29_ClNO_2_ [M + H]^+^ 464.0945; found 464.0947. 

#### 4.1.31. 1-[(4-Chlorobenzyl)(4-methylbenzyl)amino]-3-(4-chlorophenoxy)propan-2-ol (**29**)

Following the general procedure, *N*-(4-chlorobenzyl)-1-(*p*-tolyl)methanamine (150 mg, 0.61 mmol) and 2-[(4-chlorophenoxy)methyl]oxirane (112.7 mg, 0.61 mmol) afforded **29** (240 mg, 91.4%) as an oil after column chromatography (hexane/EtOAc 9:1 to 8:2). IR (ATR) 3445, 3024, 2924, 1736, 1588, 1485, 1446, 1277, 1247, 1062, 1015, 805, 746 cm^−1^. ^1^H NMR (400 MHz, CDCl_3_) δ 2.32 (s, 3H, CH_3_), 2.69–2.73 (m, 2H, CH_2_N), 3.42–3.55 (dd, *J* = 13.5, 2 Hz, 2H, CH_2_Ph), 3.72 (d, *J* = 13.5 Hz, 2H, CH_2_Ph), 3.92 (d, *J* = 5.0 Hz, 2H, CH_2_O), 4.06–4.13 (m, 1H, CH), 6.83 (dd, *J* = 8.5 Hz, 1.5 Hz, 1H, ArH), 6.88 (td, *J* = 7.5 Hz, 1.5 Hz, 1H, ArH), 7.10–7.29 (m, 10H, ArH), 7.33 (dd, *J* = 8.0, 1.5 Hz, 1H, ArH). ^13^C NMR (101 MHz, CDCl_3_) δ 21.3 (CH_3_), 56.0 (CH_2_N), 58.2 (CH_2_Ph), 58.6 (CH_2_Ph), 66.7 (CH), 71.1 (CH_2_O), 113.6 (CHAr), 121.8 (CHAr), 123.2 (CAr), 127.8 (CHAr), 128.7 (2CHAr), 129.2 (2CHAr), 129.3 (2CHAr), 130.4 (CHAr), 130.5 (2CHAr), 133.1 (CAr), 135.3 (CAr), 137.2 (CAr), 137.4 (CAr), 154.2 (CAr). HRMS C_24_H_25_Cl_2_NO_2_ [M + H]^+^ 430.1335; found 430.1341. Purity 94.16% (t_R_ = 4.51 min).

#### 4.1.32. 1-[Benzyl(3,4-dichlorobenzyl)amino]-3-(4-chlorophenoxy)propan-2-ol (**30**)

Following the general procedure, *N*-benzyl-1-(3,4-dichlorophenyl)methanamine (CAS 14502-37-5) (271.6 mg, 1.02 mmol) and 2-[(4-chlorophenoxy)methyl]oxirane (188 mg, 1.02 mmol) afforded **30** (402.5 mg, 87.5%) as an oil after column chromatography (hexane/EtOAc 9:1 to 8:2). IR (ATR) 3432, 3025, 2932, 1588, 1485, 1445, 1278, 1247, 1062, 1028, 812, 743, 698 cm^−1^.^1^H NMR (400 MHz, CDCl_3_) δ 2.68–2.79 (m, 2H, CH_2_N), 2.96 (s, 1H, OH), 3.51 (d, *J* = 13.5 Hz, 1H, CH_2_Ph), 3.57 (d, *J* = 13.5 Hz, 1H, CH_2_Ph), 3.70 (d, *J* = 13.5 Hz, 1H, CH_2_Ph), 3.74 (d, *J* = 13.5 Hz, 1H, CH_2_Ph), 3.98 (m, 2H, CH_2_O), 4.15 (m, 1H, CH), 6.82–6.95 (m, 2H, ArH), 7.13–7.23 (m, 2H, ArH), 7.25–7.43 (m, 8H, ArH).^13^C NMR (101 MHz, CDCl_3_) δ 56.1 (CH_2_N), 58.0 (CH_2_Ph), 59.1 (CH_2_Ph), 67.0 (CH), 71.1 (CH_2_O), 113.6 (CHAr), 121.9 (CHAr), 123.2 (CAr), 127.6 (CHAr), 127.9 (CHAr), 128.4 (CHAr), 128.7 (2CHAr), 129.2 (2CHAr), 130.4 (CHAr), 130.5 (CHAr), 131.0 (CHAr), 131.3 (CHAr), 132.6 (CAr), 138.2 (CAr), 139.3 (CAr), 154.2 (CAr). HRMS C_23_H_22_Cl_3_NO_2_ [M + H]^+^ 450.0789; found 450.0789. Purity 93.97% (t_R_ = 5.36 min).

#### 4.1.33. 1-[(4-Chlorobenzyl)(3,4-dichlorobenzyl)amino]-3-(4-chlorophenoxy)propan-2-ol (**31**)

Following the general procedure, *N*-(4-chlorobenzyl)-1-(3,4-dichlorophenyl)methan amine (150 mg, 0.5 mmol) and 2-[(4-chlorophenoxy)methyl]oxirane (92.1 mg, 0.5 mmol) afforded **31** (192.7 mg, 79.6%) as an oil after column chromatography (hexane/EtOAc 9:1 to 8:2). IR (ATR) 3438, 3058, 2934, 1740, 1589, 1485, 1277, 1248, 1062, 1029, 815, 746 cm^−1^. ^1^H NMR (400 MHz, CDCl_3_) δ 2.60–2.80 (m, 2H, CH_2_N), 2.88 (s, 1H, OH), 3.54 (dd, *J* = 13.5, 11.5 Hz, 2H, CH_2_), 3.69 (dd, *J* = 13.5, 11.5 Hz, 2H, CH_2_), 3.89–3.99 (m, 2H, CH_2_O), 4.12 (m, 1H, CH), 6.84 (dd, *J* = 8.0, 1.5 Hz, 1H, ArH), 6.91 (td, *J* = 7.5, 1.5 Hz, 1H, ArH), 7.12–7.25 (m, 4H, ArH), 7.27–7.40 (m, 5H, ArH). ^13^C NMR (101 MHz, CDCl_3_) δ 56.0 (CH_2_N), 58.0 (CH_2_Ph), 58.4 (CH_2_Ph), 67.2 (CH), 71.0 (CH_2_O), 113.6 (CHAr), 122.0 (CHAr), 123.1 (CAr), 127.9 (CHAr), 128.3 (CHAr), 128.9 (2CHAr), 130.4 (3CHAr), 130.6 (CHAr), 130.9 (CHAr), 131.5 (CAr), 132.7 (CAr), 133.4 (CAr), 136.8 (CAr), 139.1 (CAr), 154.1 (OCAr). HRMS C_23_H_21_Cl_4_NO_2_ [M + H]^+^ 484.0399; found 484.04. Purity 92.14% (t_R_ = 5.93 min).

#### 4.1.34. 1-(4-Chlorophenoxy)-3-[(3,4-dichlorobenzyl)(4-methylbenzyl)amino]propan-2-ol (**32**)

Following the general procedure, *N*-(3,4-dichlorobenzyl)-1-(*p*-tolyl)methanamine (151.8 mg, 0.54 mmol) and 2-[(4-chlorophenoxy)methyl]oxirane (100 mg, 0.54 mmol) afforded **32** (219.8 mg, 87.3%) as an oil after column chromatography (hexane/EtOAc 9:1 to 8:2). IR (ATR) 3414, 3019, 2928, 1737, 1588, 1485, 1445, 1247, 1061, 1029, 814, 802, 746 cm^−1^. ^1^H NMR (400 MHz, CDCl_3_) δ 2.34 (s, 3H, CH_3_), 2.67–2.78 (m, 2H, CH_2_N), 3.50 (d, *J* = 13.5 Hz, 1H, CH_2_Ph), 3.53 (d, *J* = 13.5 Hz, 1H, CH_2_Ph), 3.68 (d, *J* = 13.5 Hz, 1H, CH_2_Ph), 3.71 (d, *J* = 13.5 Hz, 1H, CH_2_Ph), 3.90–3.98 (m, 2H, CH_2_O), 4.11 (m, 1H, CH), 6.85 (dd, *J* = 8.5, 1.5 Hz, 1H, ArH), 6.90 (td, *J* = 7.5, 1.5 Hz, 1H, ArH), 7.10–7.22 (m, 6H, HAr), 7.31–7.41 (m, 3H, 3HAr). ^13^C NMR (101 MHz, CDCl_3_) δ 21.3 (CH_3_), 56.0 (CH_2_N), 57.9 (CH_2_Ph), 58.8 (CH_2_Ph), 66.9 (CH), 71.1 (CH_2_O), 113.6 (CHAr), 121.9 (CHAr), 123.1 (CAr), 127.8 (CHAr), 128.4 (CHAr), 129.1 (2CHAr), 129.4 (2CHAr), 130.4 (CHAr), 130.5 (CHAr), 130.9 (CHAr), 131.3 (CAr), 132.6 (CAr), 135.1 (CAr), 137.3 (CAr), 139.4 (CAr), 154.2 (OCAr). HRMS C_24_H_24_Cl_3_NO_2_ [M + H]^+^ 464.0945; found 464.0947. Purity 90.51% (t_R_ = 5.34 min).

#### 4.1.35. 1-[Benzyl(4-methoxybenzyl)amino]-3-(4-chlorophenoxy)propan-2-ol (**33**)

Following the general procedure, *N*-benzyl-1-(4-methoxyphenyl)methanamine (CAS 14429-02-8) (184.6 mg, 0.45 mmol) and 2-[(4-chlorophenoxy)methyl]oxirane (104 mg, 0.81 mmol) afforded **33** (283.1 mg, 84.6%) as an oil after column chromatography (hexane/EtOAc 9:1 to 8:2). IR (ATR) 2932, 2828, 1612, 1587, 1509, 1485, 1447, 1244, 1063, 1029, 810, 742, 697 cm^−1^. ^1^H NMR (400 MHz, CDCl_3_) δ 2.72 (d, *J* = 6.5 Hz, 2H, CH_2_N), 3.50 (d, *J* = 13.5 Hz, 1H, CH_2_Ph), 3.51 (d, *J* = 13.5 Hz, 1H, CH_2_Ph), 3.53 (d, *J* = 13.5 Hz, 1H, CH_2_Ph), 3.71 (d, *J* = 13.5 Hz, 1H, CH_2_Ph), 3.79 (d, *J* = 13.5 Hz, 1H, CH_2_Ph), 3.79 (s, 3H, CH_3_), 3.92 (dd, *J* = 5.0, 1.5 Hz, 2H, CH_2_), 4.10 (m, 1H, CH), 6.81–6.86 (m, 2H, ArH), 6.88 (dd, *J* = 7.5, 1.5 Hz, 2H, ArH), 7.17 (ddd, *J* = 8.0, 7.5, 1.5 Hz, 1H, ArH), 7.20–7.26 (m, 3H), 7.29–7.32 (m, 3H, ArH), 7.33 (d, *J* = 1.5 Hz, 2H, ArH). ^13^C NMR (101 MHz, CDCl_3_) δ 55.2 (CH_3_), 55.8 (CH_2_N), 58.1 (CH_2_Ph), 58.7 (CH_2_Ph), 66.4 (CH), 71.15 (CH_2_O), 113.5 (CHAr), 113.8 (CHAr), 121.6 (2CHAr), 123.0 (2CHAr), 127.3 (2CHAr), 127.6 (CHAr), 128.4 (CAr), 129.1 (CAr), 130.2 (CAr), 130.3 (CAr), 130.5 (2CAr), 138.6 (CAr), 154.2 (CAr), 158.8 (OCAr). HRMS C_24_H_27_ClNO_3_ [M + H]^+^ 484.0399; found 484.04. Purity 92.14% (t_R_ = 5.93 min).

#### 4.1.36. 1-[Bis(4-methoxybenzyl)amino]-3-(4-chlorophenoxy)propan-2-ol (**34**)

Following the general procedure, bis(4-methoxybenzyl)amine (CAS 17061-62-0) (278.8 mg, 1.08 mmol) and 2-[(4-chlorophenoxy)methyl]oxirane (200 mg, 1.08 mmol) afforded **34** (405.6 mg, 84.7%) as a white solid after column chromatography (hexane/EtOAc 9:1 to 8:2). Mp 112–114 °C (EtOAc). IR (ATR) 3409, 3005, 2932, 2834, 1586, 1510, 1486, 1446, 1249, 1232, 1060, 1026, 808, 746, 696 cm^−1^. ^1^H NMR (400 MHz, CDCl_3_) δ 2.71 (d, *J* = 6.5 Hz, 2H, CH_2_N), 3.48 (d, *J* = 13.5 Hz, 2H, CH_2_Ph), 3.72 (d, *J* = 13.5 Hz, 2H, CH_2_Ph), 3.80 (s, 4H, 2CH_3_), 3.92 (d, *J* = 5.0 Hz, 2H, CH_2_O), 4.05–4.12 (m, 1H, CH), 6.82–6.91 (m, 6H, ArH), 7.14–7.24 (m, 5H, ArH), 7.33 (dd, *J* = 8.0, 1.5 Hz, 1H, ArH). ^13^C NMR (101 MHz, CDCl_3_) δ 55.4 (2CH_3_), 55.7 (CH_2_N), 58.1 (2CH_2_Ph), 66.5 (CH), 71.3 (CH_2_O), 113.6 (C-*ipso*), 113.9 (4CHAr), 121.7 (CHAr), 123.2 (CAr), 127.8 (CHAr), 130.3 (CHAr), 130.4 (5CHAr), 130.7 (CAr), 154.3 (CAr), 158.9 (2CAr). HRMS C_25_H_28_ClNO_4_ [M + H]^+^ 442.178; found 442.1777. Purity 95.65% (t_R_ = 4.02 min).

#### 4.1.37. 1-(4-Chlorophenoxy)-3-[(3,4-dichlorobenzyl)(4-methoxybenzyl)amino]propan-2-ol (**35**)

Following the general procedure, *N*-(3,4-dichlorobenzyl)-1-(4-methoxyphenyl)methanamine (160.4 mg, 0.54 mmol) and 2-[(4-chlorophenoxy)methyl]oxirane (100 mg, 0.54 mmol) afforded **35** (251 mg, 96.4%) as an oil after column chromatography (hexane/EtOAc 9:1 to 8:2). IR (ATR) 3451, 3006, 2934, 1588, 1511, 1486, 1245, 1061, 1028, 819, 746 cm^−1^. ^1^H NMR (400 MHz, CDCl_3_) δ 2.65–2.79 (m, 2H, CH_2_N), 2.99 (s, 1H, OH), 3.51 (dd, *J* = 13.5, 3.0 Hz, 2H, CH_2_), 3.69 (d, *J* = 13.5 Hz, 2H, CH_2_), 3.80 (s, 3H, CH_3_), 3.87–4.03 (m, 2H, CH_2_O), 4.06–4.15 (m, 1H, CH), 6.83–6.93 (m, 4H, ArH), 7.12–7.23 (m, 4H, ArH), 7.31–7.40 (m, 3H, ArH). ^13^C NMR (101 MHz, CDCl_3_) δ 55.4 (CH_3_), 55.9 (CH_2_N), 57.8 (CH_2_Ph), 58.4 (CH_2_Ph), 66.9 (CH), 71.1 (CH_2_O), 113.6 (CHAr), 114.1 (2CHAr), 121.9 (CHAr), 123.1 (CAr), 127.8 (CHAr), 128.4 (CHAr), 130.1 (CAr), 130.4 (3CHAr), 130.5 (CHAr), 130.9 (CHAr), 131.3 (CAr), 132.6 (CAr), 139.4 (CAr), 154.2 (CAr), 159.1 (OCAr). HRMS C_24_H_24_Cl_3_NO_3_ [M + H]^+^ 480.0895; found 480.0894. Purity 96.56% (t_R_ = 4.71 min).

### 4.2. Cell Culture

Human Huh-7 hepatoma cells (kindly donated by Dr. Mayka Sanchez from the Josep Carreras Leukemia Research Institute, Barcelona) were cultured in DMEM supplemented with 10% fetal bovine serum and 1% penicillin-streptomycin, at 37 °C under 5% CO_2_. 

### 4.3. Reverse Transcription-Polymerase Chain Reaction and Quantitative Polymerase Chain Reaction

Isolated RNA was reverse transcribed to obtain 1 μg of complementary DNA (cDNA) using Random Hexamers (Thermo Fisher Scientific, Waltham, MA, USA), 10 mM deoxynucleotide (dNTP) mix, and the reverse transcriptase enzyme derived from the Moloney murine leukemia virus (MMLV, Thermo Fisher Scientific). The experiment was run in a thermocycler (BioRad, Hercules, CA, USA) and consisted of a program with different steps and temperatures: 65 °C for 5 min, 4 °C for 5 min, 37 °C for 2 min, 25 °C for 10 min, 37 °C for 50 min, and 70 °C for 15 min. The relative levels of specific mRNAs were assessed by real-time RT-PCR in a Mini 48-Well T100™ thermal cycler (Bio-Rad), using the SYBR Green Master Mix (Applied Biosystems, Waltham, MA, USA), as previously described [13]. Briefly, samples had a final volume of 20 μL, with 20 ng of total cDNA, 0.9 μM of the primer mix, and 10 μL of 2× SYBR Green Master Mix. The thermal cycler protocol for real-time PCR included a first step of denaturation at 95 °C for 10 min, followed by 40 repeated cycles of 95 °C for 15 s, 60 °C for 30 s, and 72 °C for 30 s for denaturation, primer annealing, and amplification, respectively. Primer sequences were designed using the Primer-BLAST tool (NCBI), based on the full mRNA sequences to find the optimal primers for amplification, and evaluated with the Oligo-Analyzer Tool (Integrated DNA Technologies) to ensure an optimal melting temperature (Tm) and avoid the formation of homo/heterodimers or non-specific structures that can interfere with the interpretation of the results. The primer sequences were designed specifically to span the junction between the exons. Values were normalized to the glyceraldehyde 3-phosphate dehydrogenase (Gapdh) expression levels, and measurements were performed in triplicate. All changes in expression were normalized to the untreated control.

### 4.4. Immunoblotting

The isolation of total protein extracts was performed as described elsewhere [13]. Immunoblotting was performed with antibodies against α-tubulin (T6074 Sigma-Aldrich, St. Louis, MO, USA), phosphorylated AMPK^T172^ (2531, Cell Signaling Technology), and GDF15 (sc-515675, Santa Cruz Biotechnology Inc, Dallas, TX, USA). Signal acquisition was conducted using the Bio-Rad ChemiDoc apparatus, and quantification of the immunoblot signal was performed with the Bio-Rad Image Lab software. The results for protein quantification were normalized to the levels of a control protein (α-tubulin) to avoid unwanted sources of variation.

### 4.5. Statistical analysis

Results are expressed as the mean ± SEM. Significant differences were assessed by two-way ANOVA using the GraphPad Prism program (version 9.0.2) (GraphPad Software Inc., San Diego, CA, USA). When significant variations were found by ANOVA, Tukey’s post hoc test for multiple comparisons was performed only if F achieved a *p*-value < 0.05. Differences were considered significant at *p* < 0.05.

## Figures and Tables

**Table 1:** Assessment of the effects of the compounds **1**–**35** on human *GDF15* expression. *GDF15* mRNA levels in the human liver cell line Huh-7 exposed to 5 mM metformin, 30 µM BC1618, or 30 µM of new compound for 24 h. Data are presented as the mean ± SEM (*n* = 3). * *p* < 0.05, ** *p* < 0.01, and *** *p* < 0.001 vs. control. *p*-values determined by one-way ANOVA with Tukey’s post hoc test.

Entry	Compound	Structure	GDF15 mRNA Levels (%)	GDF15 mRNA Levels Regarding BC1618 (%)	Activity Change Regarding BC1618 (%)
1	**Control**	-	100.00 ± 16.00	9.60	−90.40
2	**Metformin**	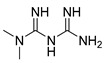	273.49 ± 3.79	26.26	−73.74
		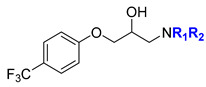			
3	**BC1618**	R_1_ = R_2_ = Bn	1041.41 ± 83.58 *	100.00	0.00
4	**1**	R_1_ = H, R_2_ = Bn	1692.98 ± 120.22 ***	162.57	+62.57
5	**2**	R_1_ = Me, R_2_ = Bn	377.99 ± 71.33	36.30	−63.70
6	**3**	R_1_ = R_2_ = Me	296.39 ± 7.77	28.46	−71.54
7	**4**	R_1_ = Ph, R_2_ = Bn	986.69 ± 24.56 *	94.75	−5.25
8	**5**	R_1_ = CH_2_-3-pyridine, R_2_ = Bn	394.10 ± 75.18	37.84	−62.16
		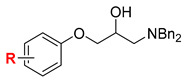			
9	**6**	R = H	1021.35 ± 167.60 ***	98.07	−1.93
10	**7**	R = *p*-Cl	1249.04 ± 181.39 **	119.94	+19.94
11	**8**	R = *p*-Me	1107.44 ± 152.04 *	106.34	+6.34
12	**9**	R = *p*-isopropyl	871.04 ± 91.59 **	83.64	−16.36
13	**10**	R = *p*-*tert*-butyl	640.00 ± 126.21	61.46	−38.54
14	**11**	R = *p*-cyclohexyl	630.49 ± 160.87 *	60.54	39.46
15	**12**	R = *p*-cyclopentyl	942.87 ± 291.40 ***	90.54	−9.46
16	**13**	R = *m*,*p*-diMe	1807.01 ± 190.07 ***	173.52	+73.52
17	**14**	R = *p*-OMe	988.06 ± 137.45 ***	94.88	−5.12
18	**15**	R = *p*-OBn	275.85 ± 74.89	26.49	−73.51
19	**16**	R = *p*-Opropyl	592.33 ± 220.72	56.88	−43.12
20	**17**	R = *m*,*p*-diCl	664.89 ± 201.62	63.85	−36.15
21	**18**	R = *m*-CF_3_,*p*-Cl	446.25 ± 34.9	42.85	−57.15
22	**19**	R = *m*-CF_3_,*p*-NO_2_	520.63 ± 37.79	49.99	−50.01
23	**20**	R = o,*p*-diCl	1172.43 ± 168.93 **	112.58	+12.58
24	**21**	R = *p*-Br	1464.51 ± 16.27 ***	140.63	+40.63
25	**22**	R = *p*-NO_2_	1050.44 ± 69.45 ***	100.87	+0.87
26	**23**	R = *p*-I	517.48 ± 196.49	49.69	−50.31
27	**24**	R = *p*-SF_5_	720.70 ± 107.34 *	69.20	−30.80
		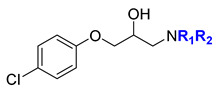			
28	**25**	R_1_ = Bn, R_2_ = *p*-ClBn	-	-	-
29	**26**	R_1_ = R_2_ = *p*-ClBn	150.28 ± 18.68	14.43	−85.57
30	**27**	R_1_ = Bn, R_2_ = *p*-MeBn	177.92 ± 58.52	17.08	−82.92
31	**28**	R_1_ = R_2_ = *p*-MeBn	205.79 ± 22.24 *	19.76	−80.24
32	**29**	R_1_ = *p*-ClBn, R_2_ = *p*-MeBn	219.48 ± 21.63 *	21.08	−78.92
33	**30**	R_1_ = Bn, R_2_ = *m*,*p*-diClBn	176.61 ± 40.52	16.96	−83.04
34	**31**	R_1_ = *p*-ClBn, R_2_ = *m*,*p*-diClBn	128.74 ± 11.19	12.36	−87.64
35	**32**	R_1_ = *p*-Me, R_2_ = *m*,*p*-diClBn	143.90 ± 34.31	13.82	−86.18
36	**33**	R_1_ = Bn, R_2_ = *p*-MeOBn	98.35 ± 65.89	9.44	−90.56
37	**34**	R_1_ = R_2_ = *p*-MeOBn	161.63 ± 12.29	15.52	−84.48
38	**35**	R_1_ = *m*,*p*-diClBn, R_2_ = *p*-MeOBn	186.69 ± 27.81	17.93	−82.07

## Data Availability

The study data are available upon request.

## Supplementary Materials

The following supporting information can be downloaded at: https://www.mdpi.com/article/10.3390/molecules28145468/s1, General procedure for the preparation of epoxyde intermediates; ^1^H-NMR and ^13^C-NMR spectra; HPLC/MS analysis, Smiles, HETCOR, HMRS.

## References

[1] Aguilar-Recarte D., Barroso E., Palomer X., Wahli W., Vázquez-Carrera M. (2022). Knocking on GDF15′s door for the treatment of type 2 diabetes mellitus. Trends Endocrinol. Metab..

[2] Hsu J.-Y., Crawley S., Chen M., Ayupova D.A., Lindhout D.A., Higbee J., Kutach A., Joo W., Gao Z., Fu D. (2017). Non-homeostatic body weight regulation through a brainstem-restricted receptor for GDF15. Nature.

[3] Tran T., Yang J., Gardner J., Xiong Y. (2018). GDF15 deficiency promotes high fat diet-induced obesity in mice. PLoS ONE.

[4] Emmerson P.J., Wang F., Du Y., Liu Q., Pickard R.T., Gonciarz M.D., Coskun T., Hamang M.J., Sindelar D.K., Ballman K.K. (2017). The metabolic effects of GDF15 are mediated by the orphan receptor GFRAL. Nat. Med..

[5] Yang L., Chang C.-C., Sun Z., Madsen D., Zhu H., Padkjær S.B., Wu X., Huang T., Hultman K., Paulsen S.J. (2017). GFRAL is the receptor for GDF15 and is required for the anti-obesity effects of the ligand. Nat. Med..

[6] Benichou O., Coskun T., Gonciarz M.D., Garhyan P., Adams A.C., Du Y., Dunbar J.D., Martin J.A., Mather K.J., Pickard R.T. (2023). Discovery, development, and clinical proof of mechanism of LY3463251, a long-acting GDF15 receptor agonist. Cell Metab..

[7] Zhang Y., Zhao X., Dong X., Zhang Y., Zou H., Jin Y., Guo W., Zhai P., Chen X., Kharitonenkov A. (2023). Activity-balanced GLP-1/GDF15 dual agonist reduces body weight and metabolic disorder in mice and non-human primates. Cell Metab..

[8] Gerstein H.C., Pare G., Hess S., Ford R.J., Sjaarda J., Raman K., McQueen M., Lee S., Haenel H., Steinberg G.R. (2017). Growth Differentiation Factor 15 as a Novel Biomarker for Metformin. Diabetes Care.

[9] Coll A.P., Chen M., Taskar P., Rimmington D., Patel S., Tadross J.A., Cimino I., Yang M., Welsh P., Virtue S. (2020). GDF15 mediates the effects of metformin on body weight and energy balance. Nature.

[10] EDay A., Ford R.J., Smith B.K., Mohammadi-Shemirani P., Morrow M.R., Gutgesell R.M., Lu R., Raphenya A.R., Kabiri M., McArthur A.G. (2019). Metformin-induced increases in GDF15 are important for suppressing appetite and promoting weight loss. Nat. Metab..

[11] Klein A.B., Nicolaisen T.S., Johann K., Fritzen A.M., Mathiesen C.V., Gil C., Pilmark N.S., Karstoft K., Blond M.B., Quist J.S. (2022). The GDF15-GFRAL pathway is dispensable for the effects of metformin on energy balance. Cell Rep..

[12] Zhang S.-Y., Bruce K., Danaei Z., Li R.J., Barros D.R., Kuah R., Lim Y.-M., Mariani L.H., Cherney D.Z., Chiu J.F. (2023). Metformin triggers a kidney GDF15-dependent area postrema axis to regulate food intake and body weight. Cell Metab..

[13] He L. (2020). Metformin and Systemic Metabolism. Trends Pharmacol. Sci..

[14] Aguilar-Recarte D., Barroso E., Zhang M., Rada P., Pizarro-Delgado J., Peña L., Palomer X., Valverde M., Wahli W., Vázquez-Carrera M. (2022). A positive feedback loop between AMPK and GDF15 promotes metformin antidiabetic effects. Pharmacol. Res..

[15] Liu Y., Jurczak M.J., Lear T.B., Lin B., Larsen M.B., Kennerdell J.R., Chen Y., Huckestein B.R., Nguyen M.K., Tuncer F. (2021). A Fbxo48 inhibitor prevents pAMPKalpha degradation and ameliorates insulin resistance. Nat. Chem. Biol..

[16] Chen B., Mallampalli R.K., Liu Y. (2018). WO2018067685A1.

[17] Xie B., Murali A., Vandevender A.M., Chen J., Silva A.G., Bello F.M., Chuan B., Bahudhanapati H., Sipula I., Dedousis N. (2022). Hepatocyte-derived GDF15 suppresses feeding and improves insulin sensitivity in obese mice. iScience.

[18] Zarei M., Pujol E., Quesada-López T., Villarroya F., Barroso E., Vázquez S., Pizarro-Delgado J., Palomer X., Vázquez-Carrera M. (2019). Oral administration of a new HRI activator as a new strategy to improve high-fat-diet-induced flucose intolerance, hepatic steatosis, and hypertriglyceridaemia through FGF21. Br. J. Pharmacol..

[19] Kincaid J.W.R., Coll A.P. (2023). Metformin and GDF15: Where are we now?. Nat. Rev. Endocrinol..

[20] Draz H., Goldberg A.A., Titorenko V.I., Guns E.S.T., Safe S.H., Sanderson J.T. (2017). Diindolylmethane and its halogenated derivatives induce protective autophagy in human prostate cancer cells via induction of the oncogenic protein AEG-1 and activation of AMP-activated protein kinase (AMPK). Cell. Signal..

