# Supplementary material for: Design and Synthesis of AMPK Activators and GDF15 Inducers
Source: Molecules. 2023 Jul 17;28(14):5468. doi: 10.3390/molecules28145468 (PMC10386161; doi:10.3390/molecules28145468)

Article

Design and Synthesis of AMPK Activators and GDF15 Inducers

Meijian Zhang [1,2,3,†], Andrea Bagán [4,†], Donna Martínez [4], Emma Barroso [1,2,3], Xavier Palomer [1,2,3], Santiago Vázquez [4], Carmen Escolano [4,*] and Manuel Vázquez-Carrera [1,2,3,*]

1 Department of Pharmacology, Toxicology and Therapeutic Chemistry, Institute of Biomedicine (IBUB), Faculty of Pharmacy and Food Sciences, University of Barcelona, Av. Joan XXIII, 27-31, 08028 Barcelona, Spain; zmeijian@gmail.com (M.Z.); ebarroso@ub.edu (E.B.); xpalomer@ub.edu (X.P.)
2 Spanish Biomedical Research Center in Diabetes and Associated Metabolic Diseases (CIBERDEM)-Instituto de Salud Carlos III, 28029 Madrid, Spain
3 Pediatric Research Institute-Hospital Sant Joan de Déu, 08950 Esplugues de Llobregat, Spain
4 Laboratory of Medicinal Chemistry (Associated Unit to CSIC), Department of Pharmacology, Toxicology and Medicinal Chemistry, Faculty of Pharmacy and Food Sciences, Institute of Biomedicine (IBUB), University of Barcelona, Av. Joan XXIII, 27-31, 08028 Barcelona, Spain; andreabaganp@gmail.com (A.B.); luzy779@gmail.com (D.M.); svazquez@ub.edu (S.V.)
* Correspondence: cescolano@ub.edu (C.E.); mvazquezcarrera@ub.edu (M.V.-C.)
† These authors contributed equally to this work.

Contents:

General procedure for the preparation of epoxyde intermediates 2

^{1}H-NMR and ^{13}C-NMR spectra of compounds **BC1615**, **1-15** 3

HPLC/MS analysis of **BC1618**, **1-35** 39

Smiles 58

HETCOR 59

1.1.1. General Procedure for the synthesis of phenoxymethyloxirane derivatives.

A mixture of the corresponding phenol derivative (1 equiv) [4-(trifluoromethyl)phenol, phenol, 4-chlorophenol, 4-methylphenol, 4-isopropylphenol, 4-*tert*-butylphenol, 4-cyclohexylphenol, 4-cyclopentylphenol, 4-benzyloxyphenol, 3,4-dimethylphenol, 4-methoxyphenol, 4-propoxyphenol, 3,4-dichlorophenol, 4-chloro-3-(trifluoromethyl)phenol, 4-nitro-3-(trifluoromethyl)phenol, 2,4-dichlorophenol], (±)-epichlorohydrin (10 equiv) and K_2CO_3 (1.2 equiv) was stirred in methanol at 120 ºC for 4 h. The mixture was evaporated to yield a residue, which was purified by column chromatography (hexane/AcOEt) to afford pure products; 2-{[4-(trifluoromethyl)phenoxy]methyl}oxirane; 2-(phenoxymethyl)oxirane; 2-[(4-chlorophenoxy)methyl]oxirane; 2-[(*p*-tolyloxy)methyl]oxirane; 2-[(4-isopropylphenoxy)methyl]oxirane, 2-{[(4-(*tert*-butyl)phenoxy]methyl}oxirane, 2-[(4-cyclohexylphenoxy)methyl]oxirane, 2-[(4-cyclopentylphenoxy)methyl]oxirane, 2-{[4-(benzyloxy)phenoxy]methyl}oxirane, 2-[(3,4-dimethylphenoxy)methyl]oxirane, 2-[(4-methoxyphenoxy)methyl]oxirane, 2-[(4-propoxyphenoxy)methyl]oxirane, 2-[(3,4-dichlorophenoxy)methyl]oxirane, 2-{[4-chloro-3-(trifluoromethyl)phenoxy]methyl}oxirane, 2-{[4-nitro-3-(trifluoromethyl)phenoxy]methyl}oxirane, 2-[(2,4-dichlorophenoxy)methyl]oxirane.

^{1}H-NMR and ^{13}C-NMR spectra of new compounds BC1618, 1-35

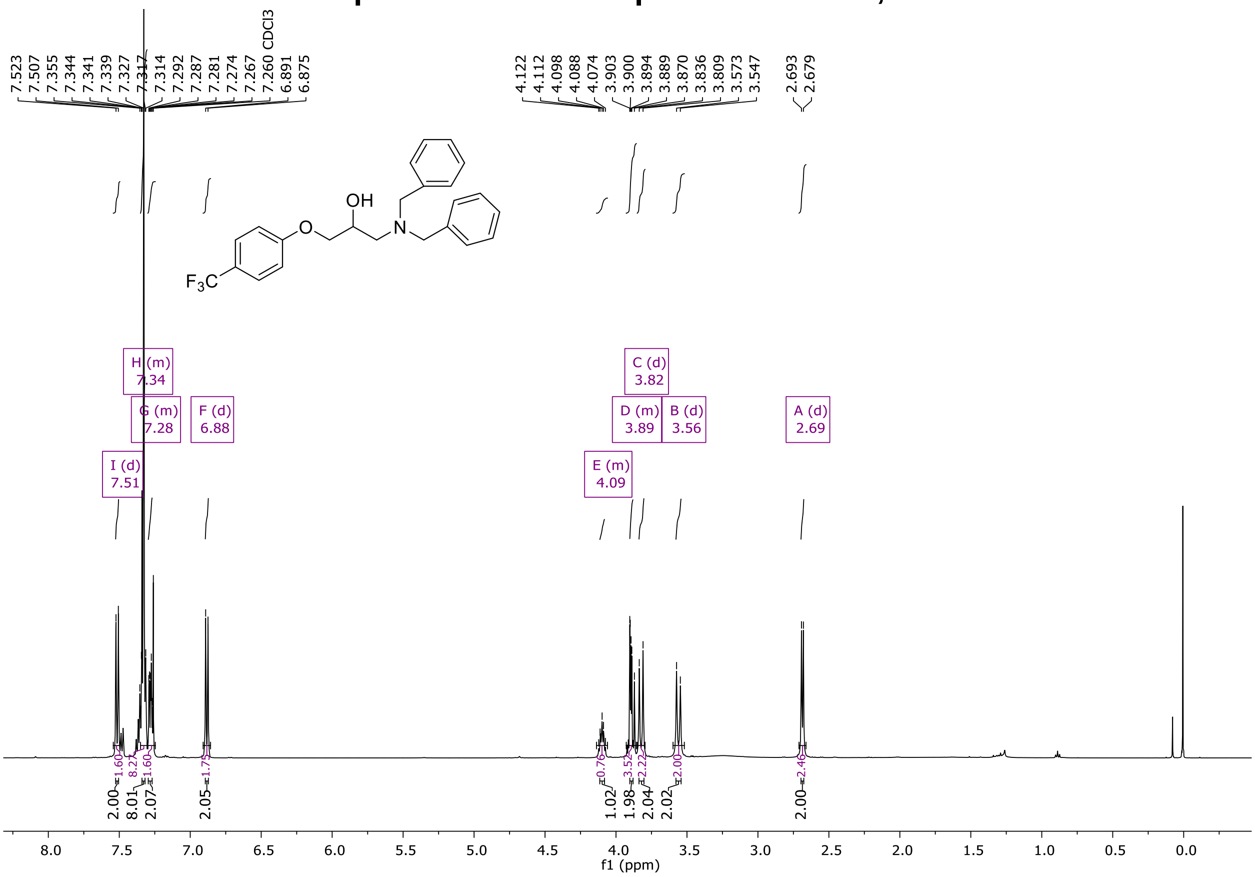

1-(Dibenzylamino)-3-[4-(trifluoromethyl)phenoxy]propan-2-ol (BC1618)

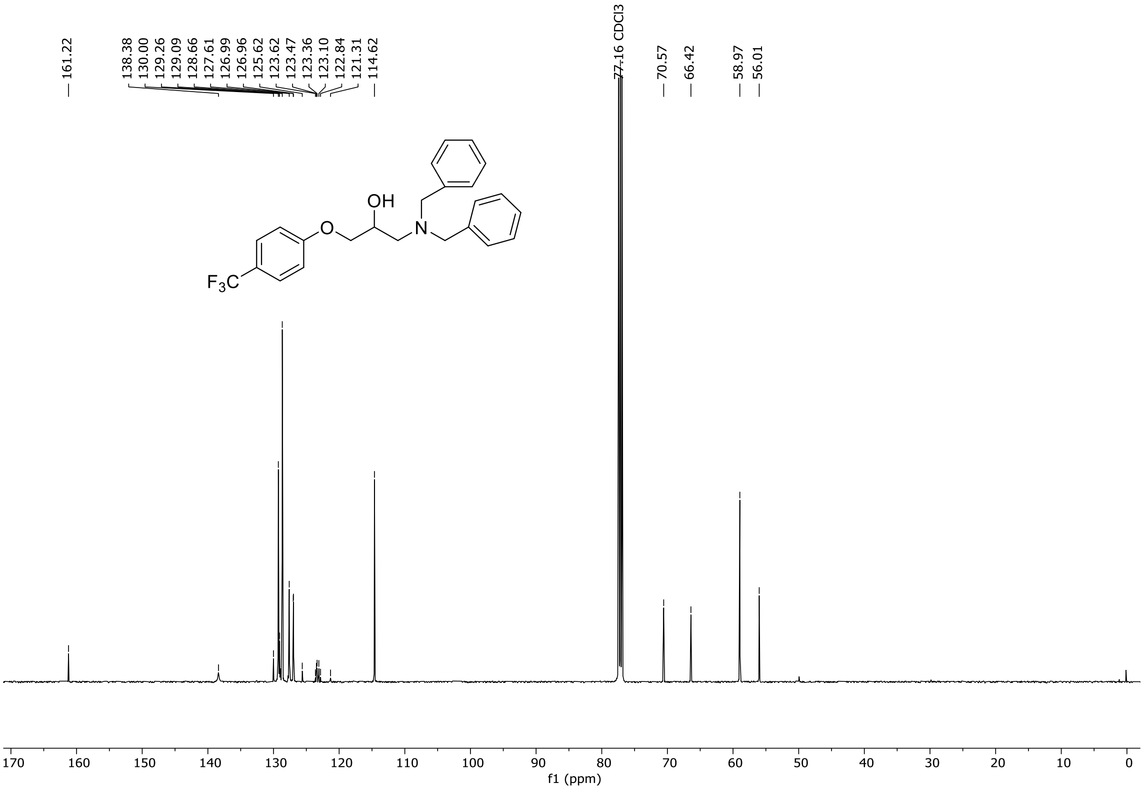

1-(Dibenzylamino)-3-[4-(trifluoromethyl)phenoxy]propan-2-ol (BC1618)

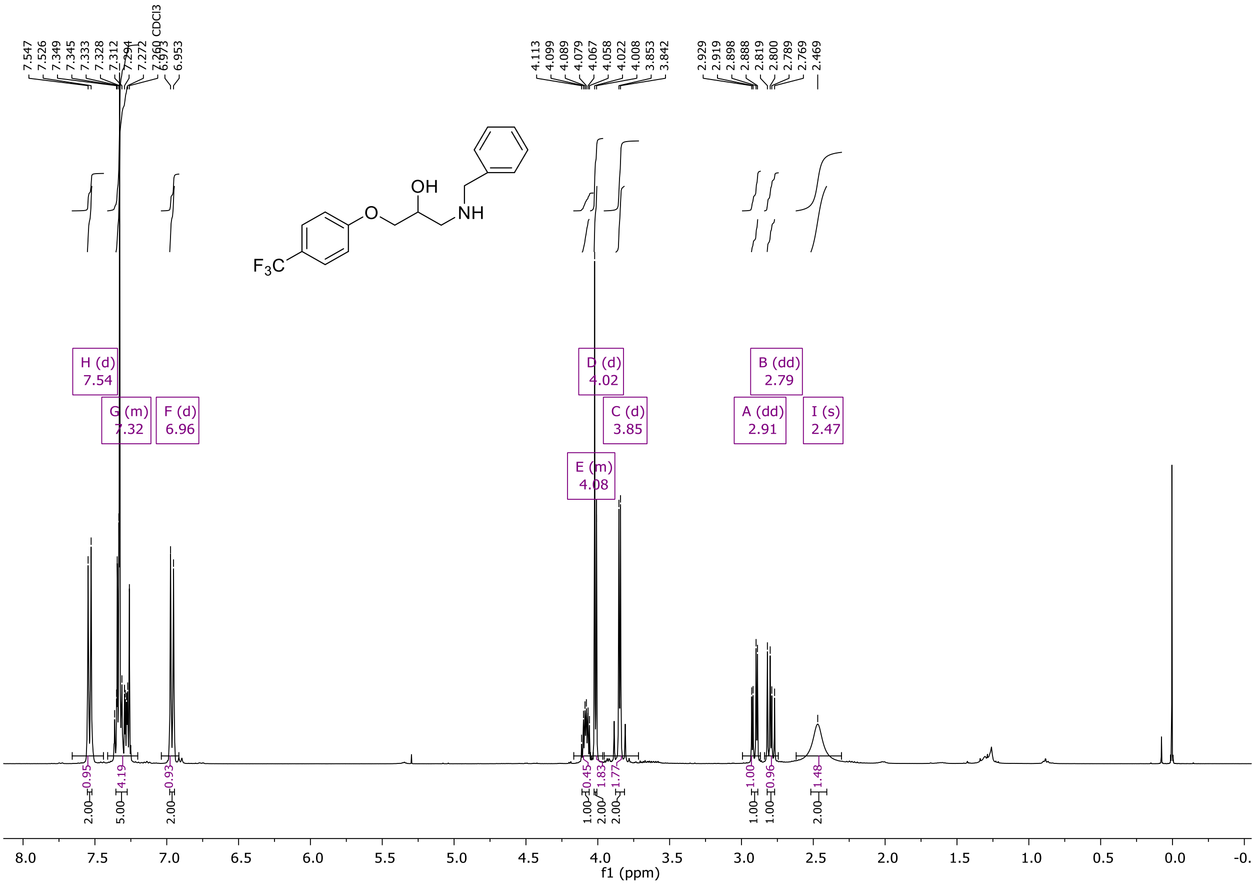

1-(Benzylamino)-3-[4-(trifluoromethyl)phenoxy]propan-2-ol (1)

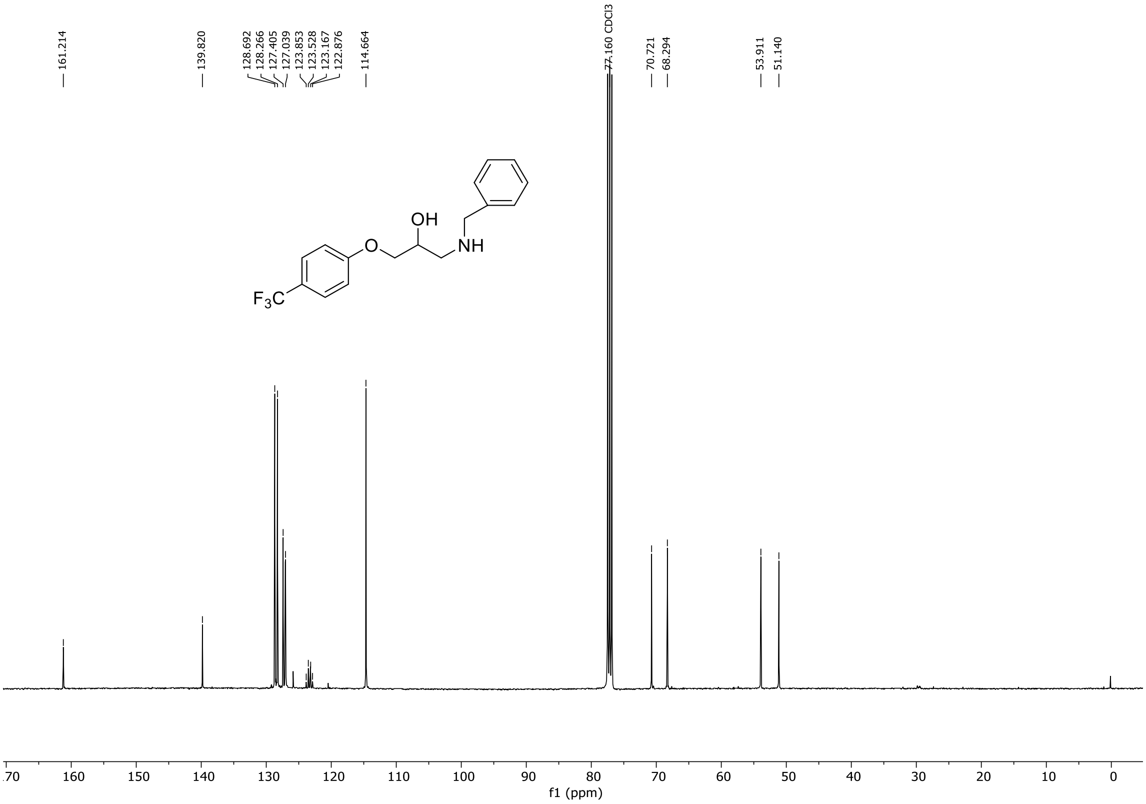

1-(Benzylamino)-3-[4-(trifluoromethyl)phenoxy]propan-2-ol (1)

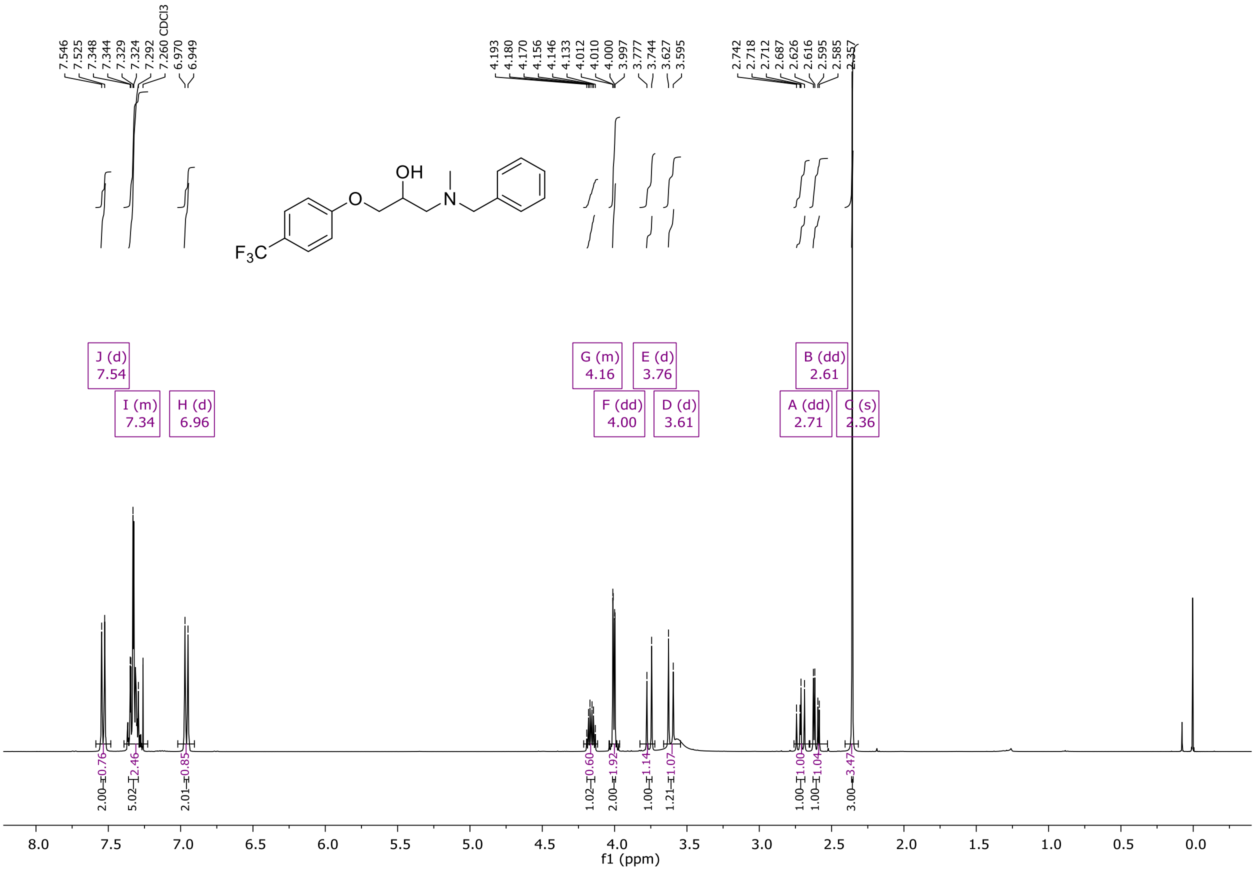

1-[Methyl(phenyl)amino]-3-[4-(trifluoromethyl)phenoxy]propan-2-ol (2)

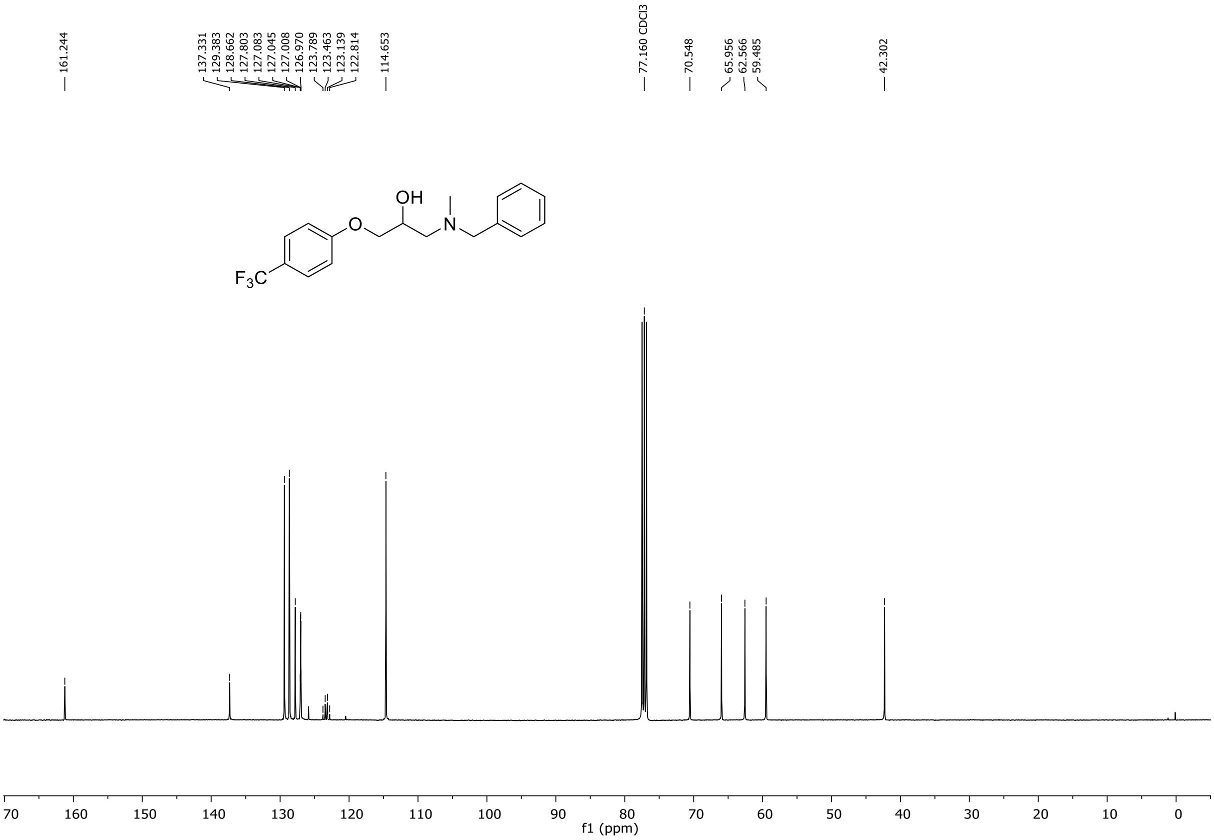

1-[Methyl(phenyl)amino]-3-[4-(trifluoromethyl)phenoxy]propan-2-ol (2)

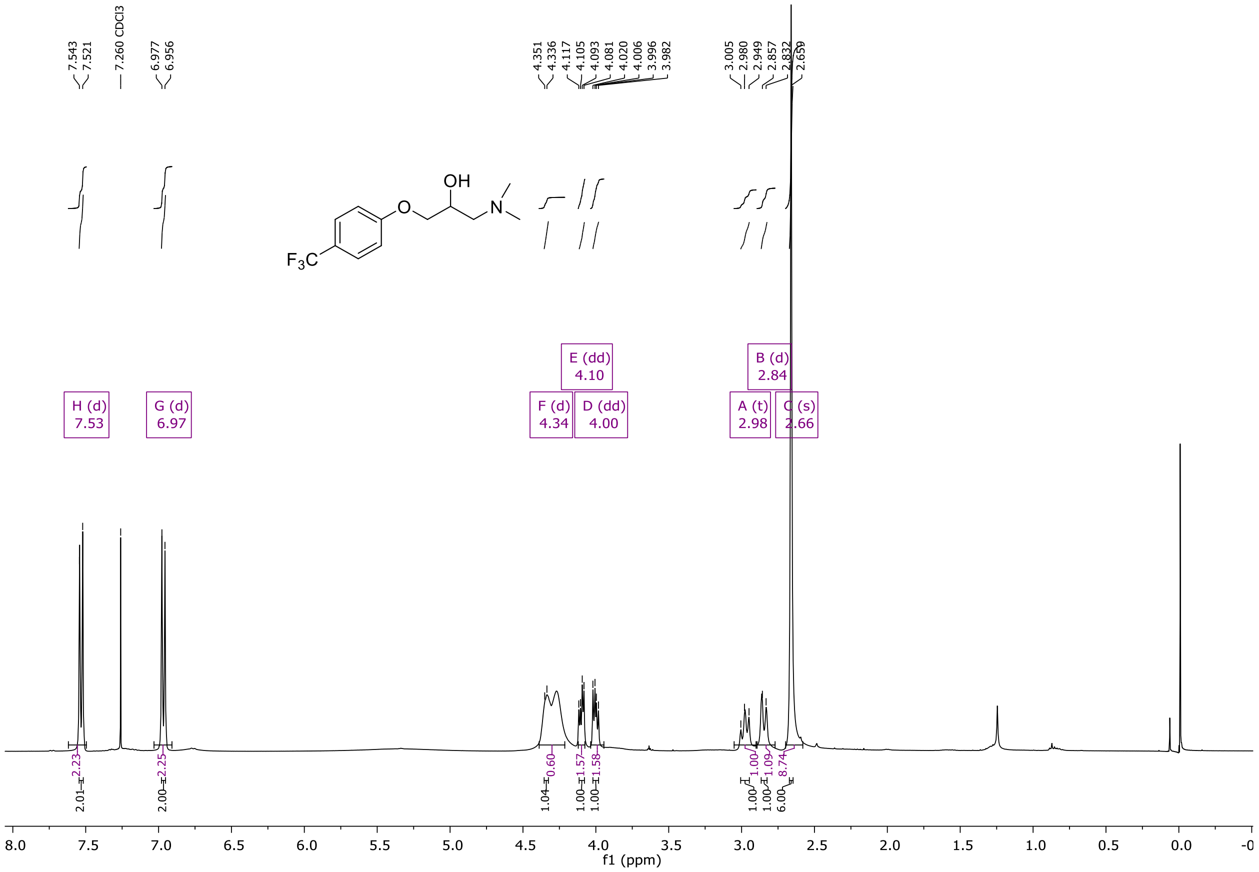

1-(Dimethylamino)-3-[4-(trifluoromethyl)phenoxy]propan-2-ol (3)

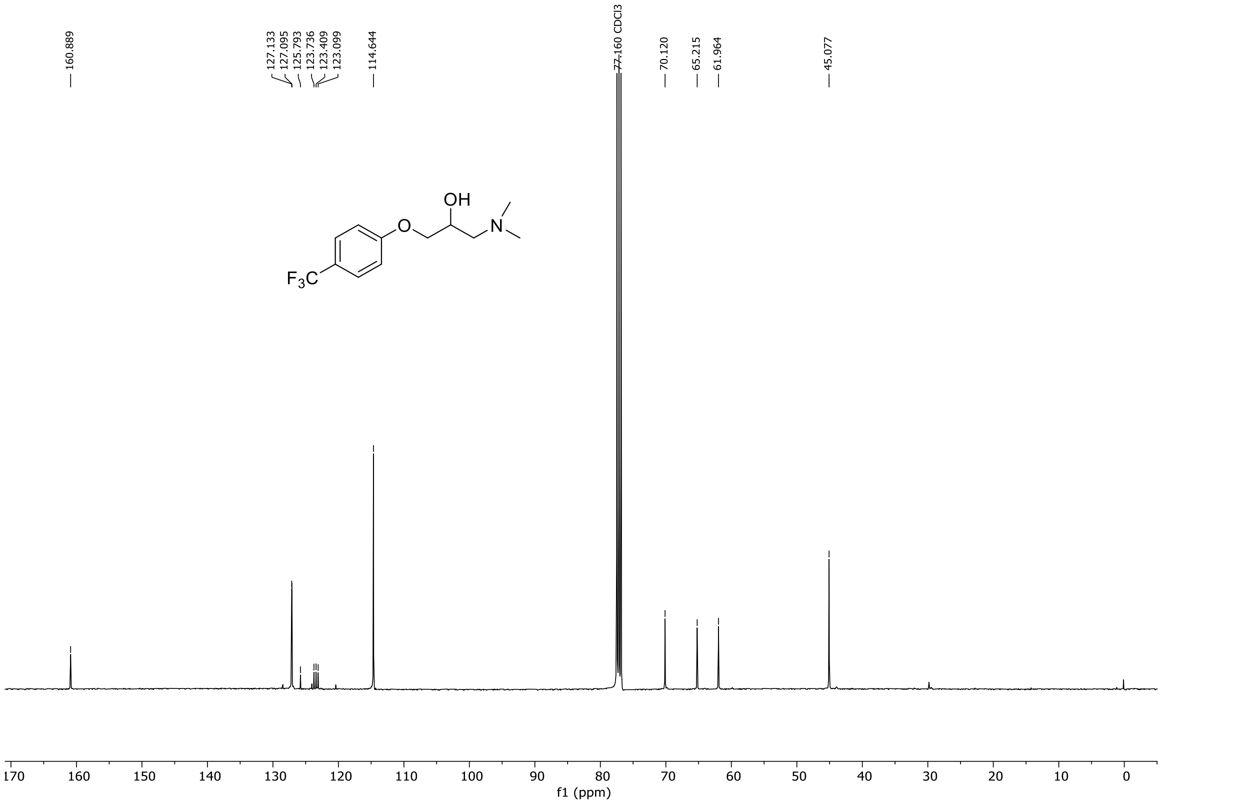

1-(Dimethylamino)-3-[4-(trifluoromethyl)phenoxy]propan-2-ol (3)

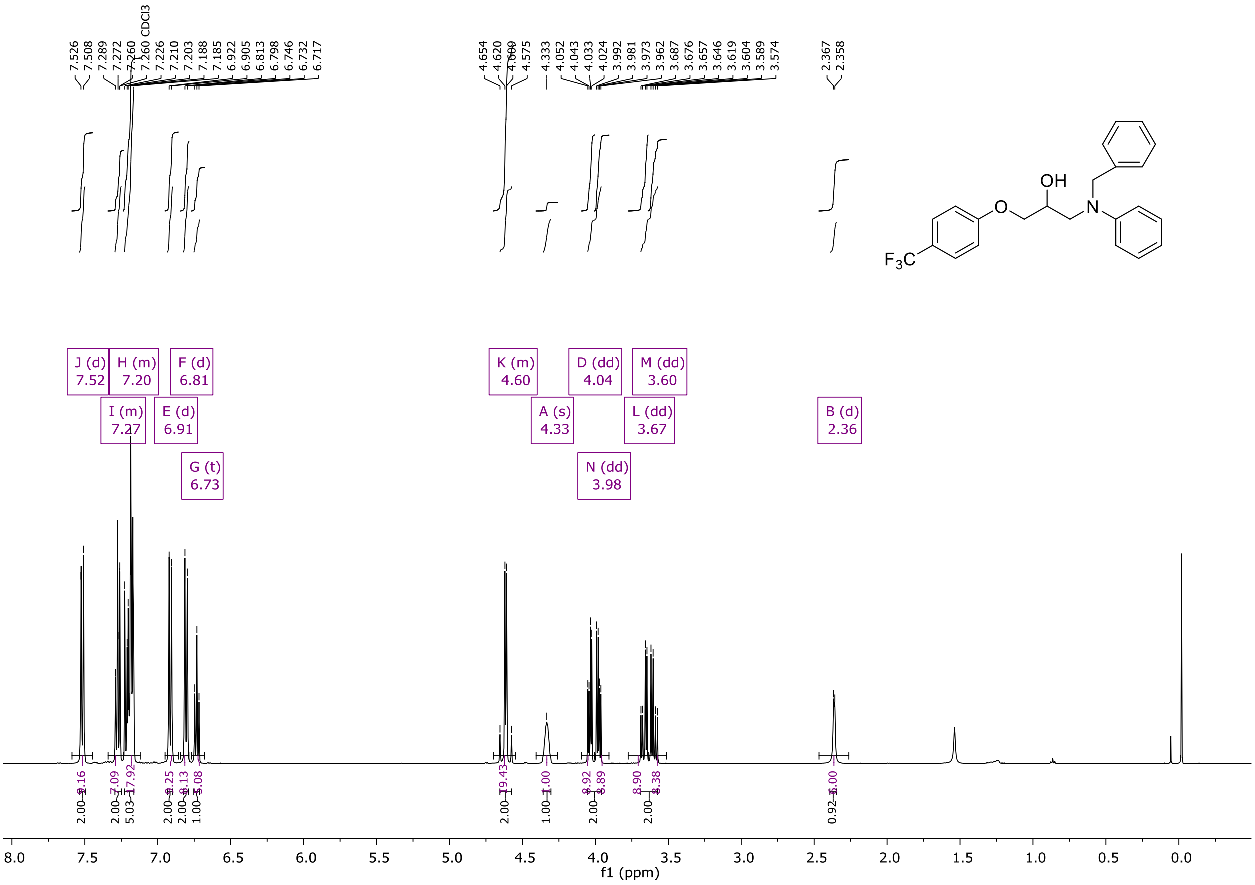

1-[Benzyl(phenyl)amino]-3-[4-(trifluoromethyl)phenoxy]propan-2-ol (4)

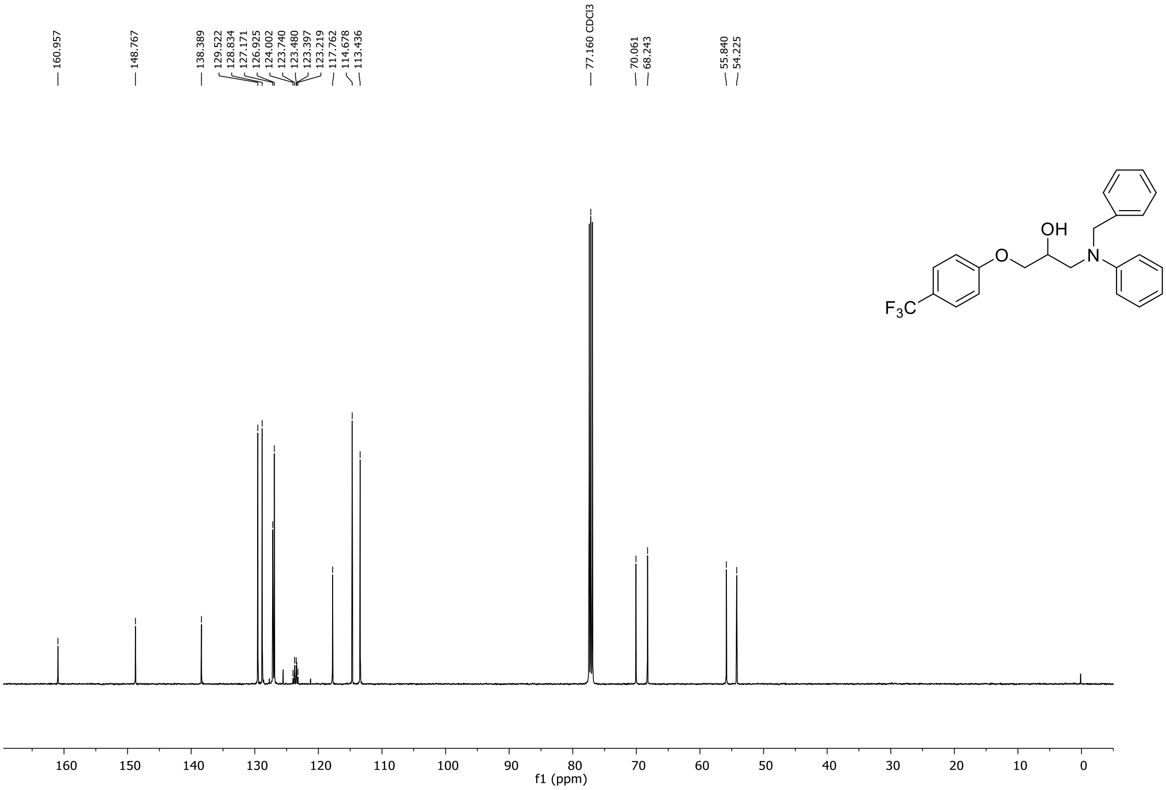

1-[Benzyl(phenyl)amino]-3-[4-(trifluoromethyl)phenoxy]propan-2-ol (4)

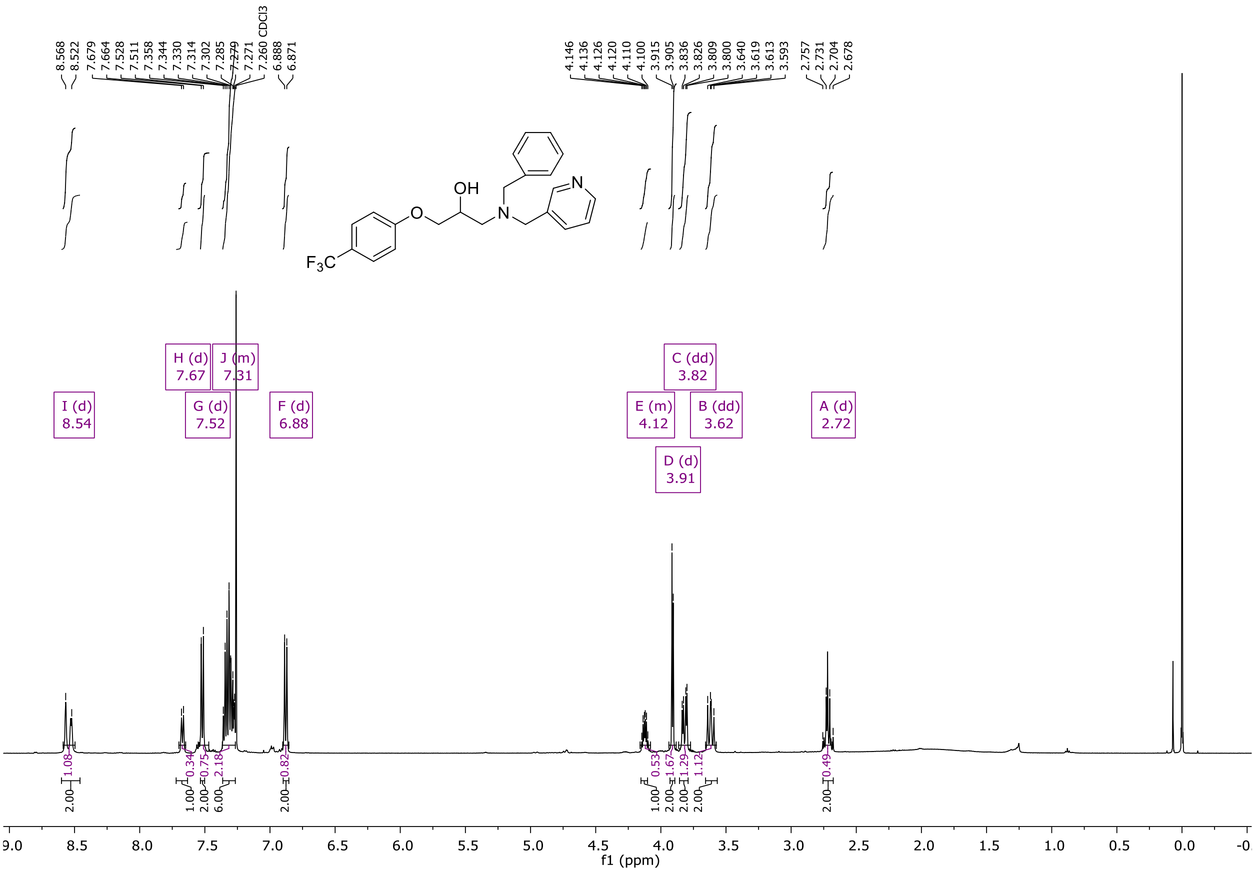

1-[Benzyl(pyridin-3-ylmethyl)amino]-3-[4-(trifluoromethyl)phenoxy]propan-2-ol (5)

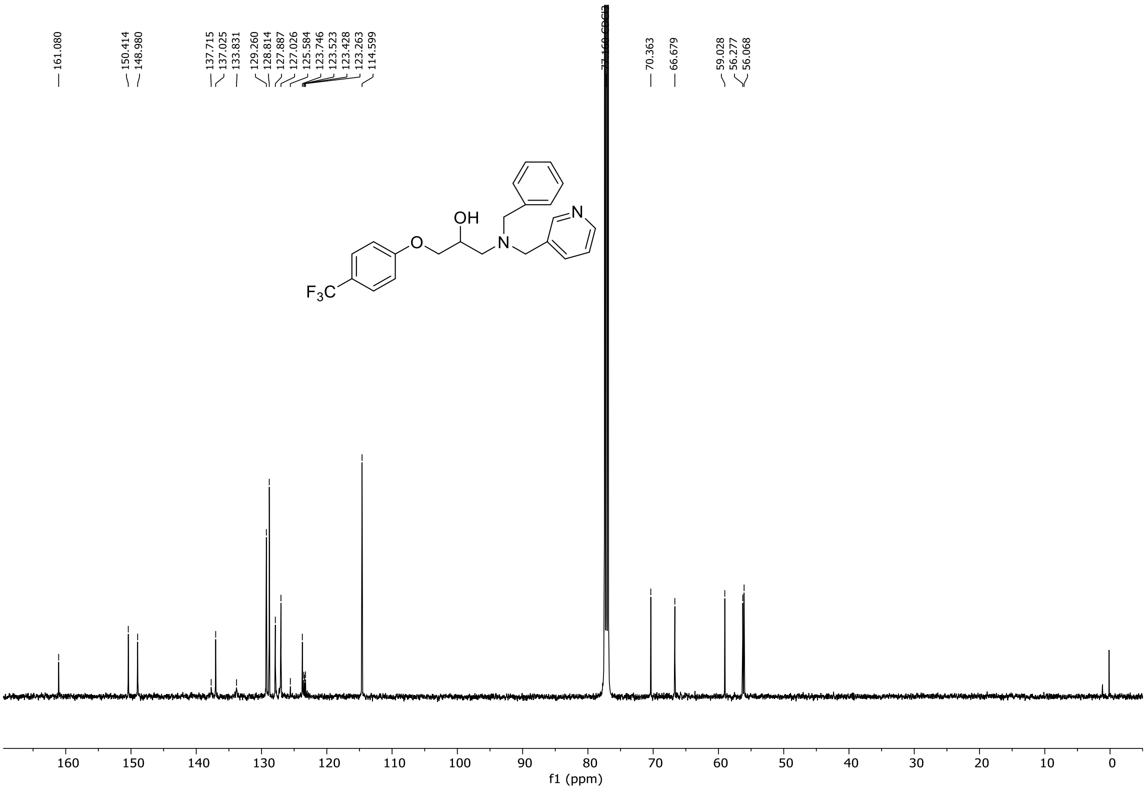

1-[Benzyl(pyridin-3-ylmethyl)amino]-3-[4-(trifluoromethyl)phenoxy]propan-2-ol (5)

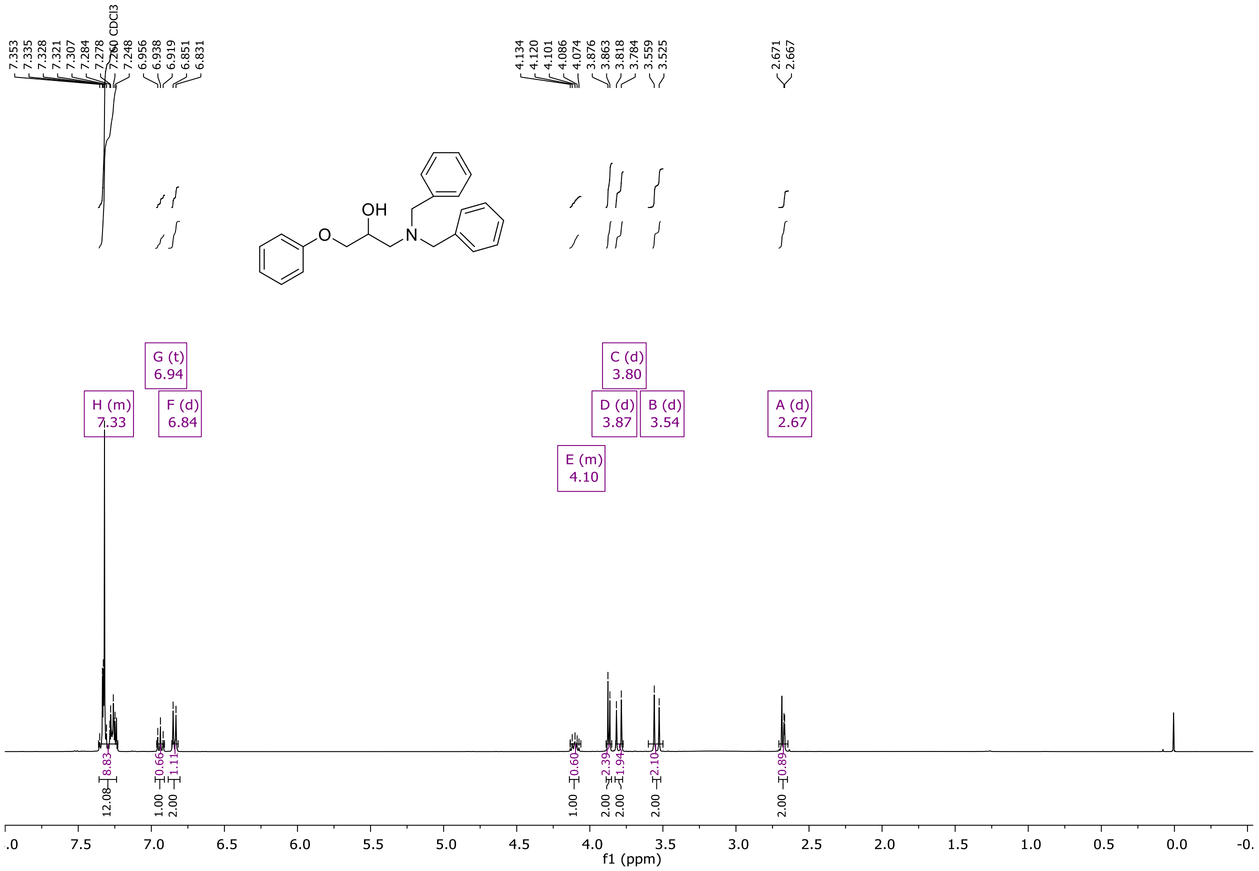

1-(Dibenzylamino)-3-phenoxypropan-2-ol (6)

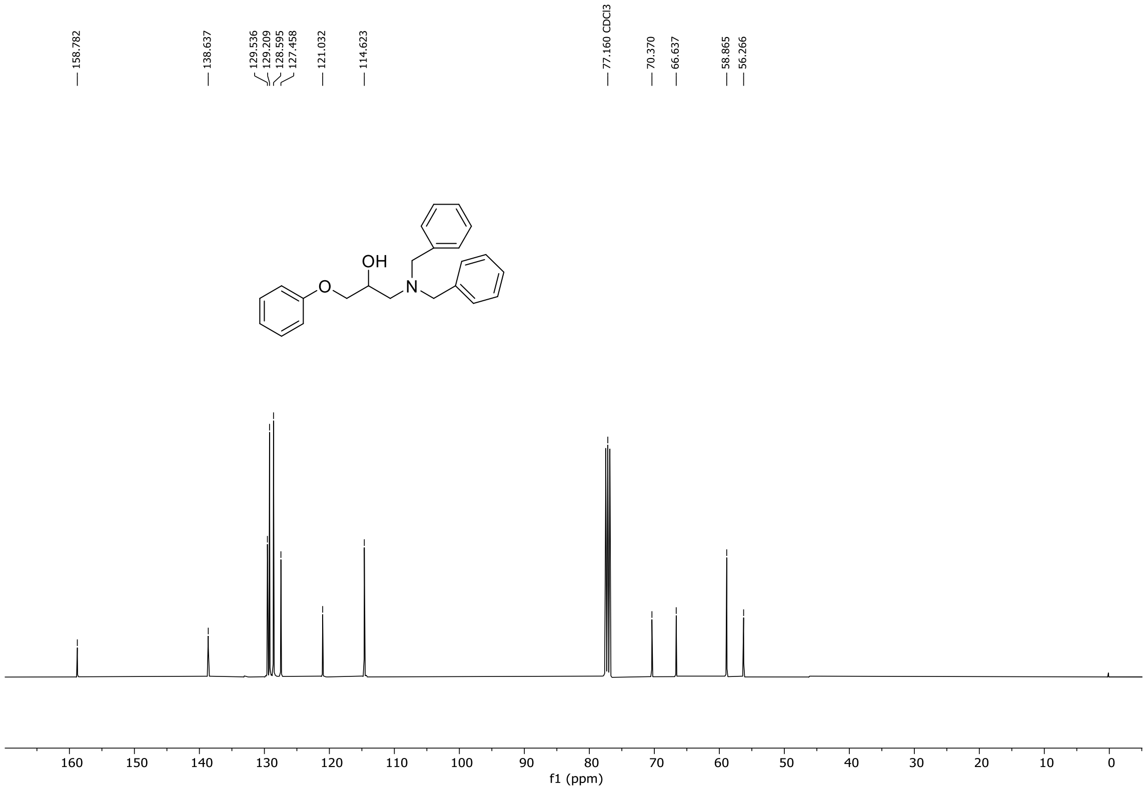

1-(Dibenzylamino)-3-phenoxypropan-2-ol (6)

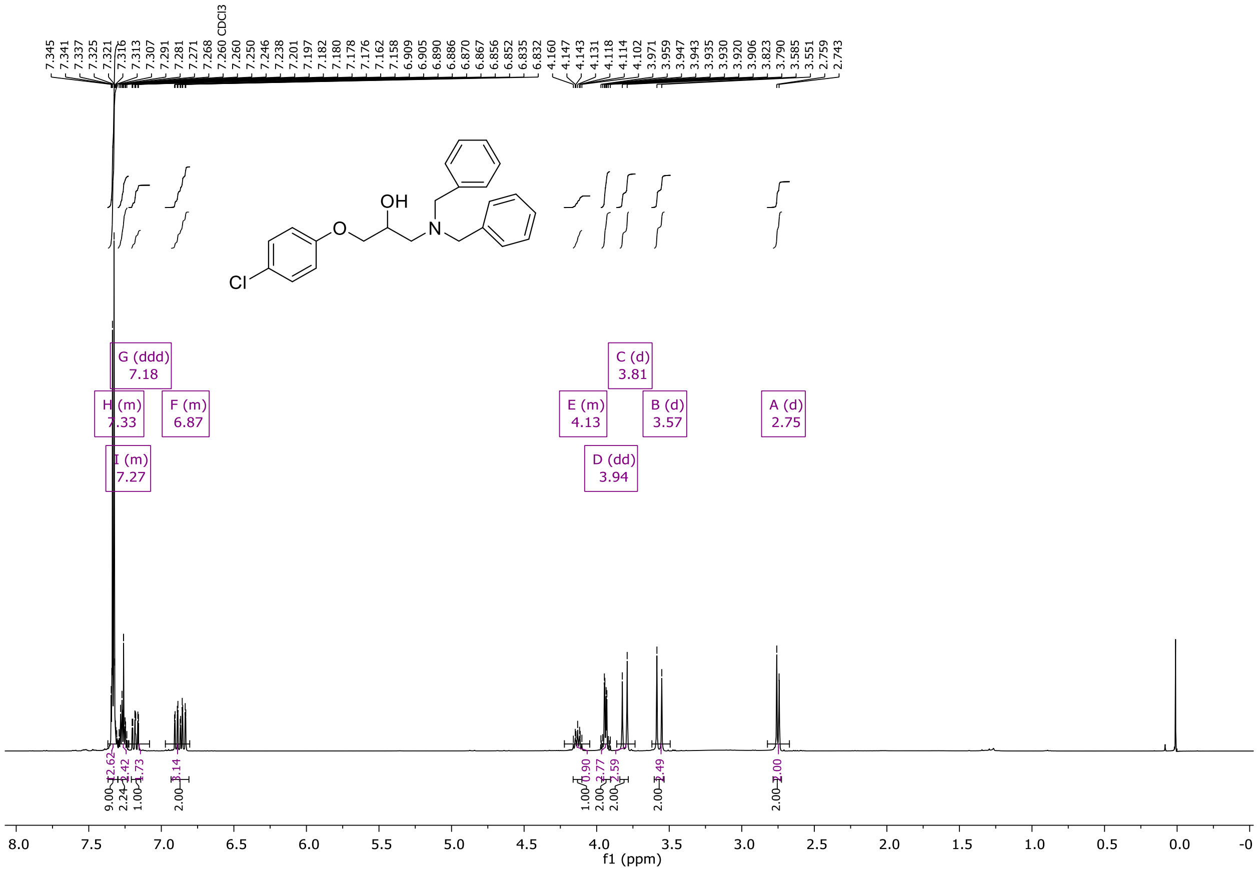

1-(4-Chlorophenoxy)-3-(dibenzylamino)propan-2-ol (7)

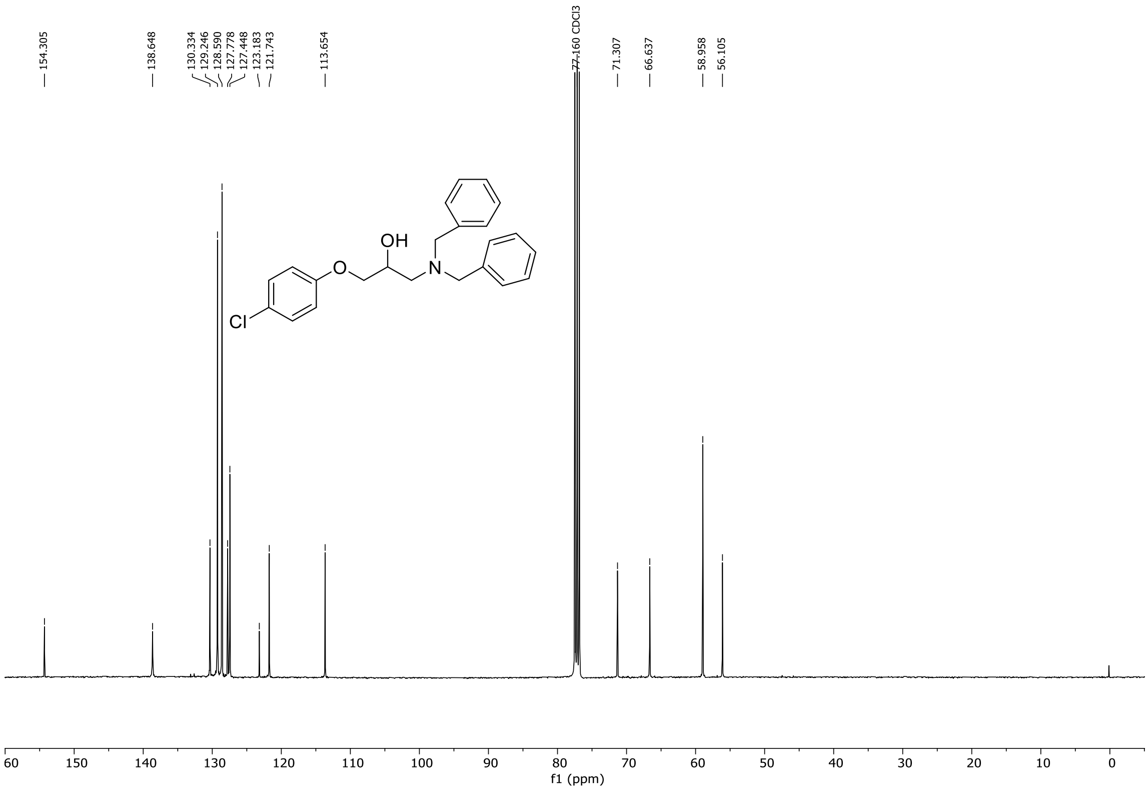

1-(4-Chlorophenoxy)-3-(dibenzylamino)propan-2-ol (7)

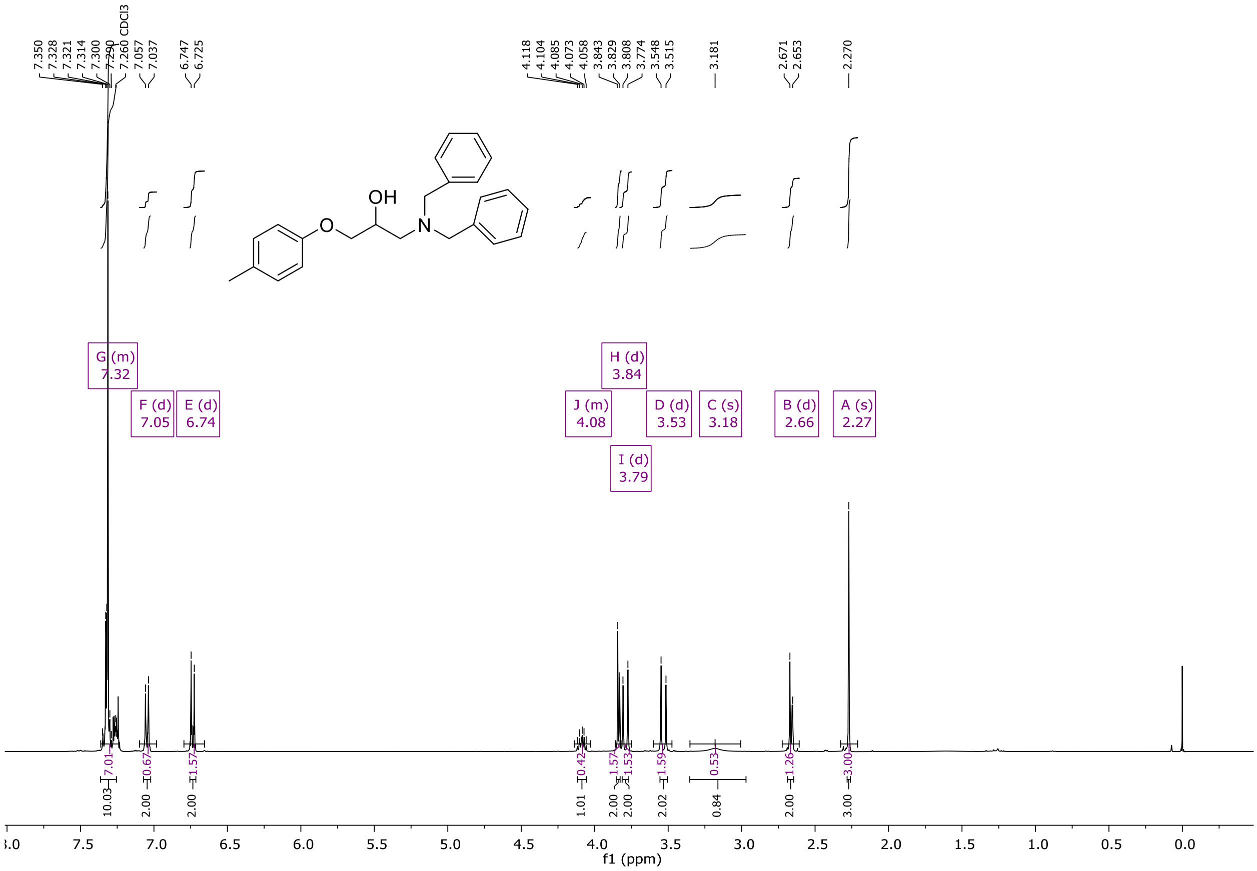

1-(Dibenzylamino)-3-(*p*-tolyloxy)propan-2-ol (8)

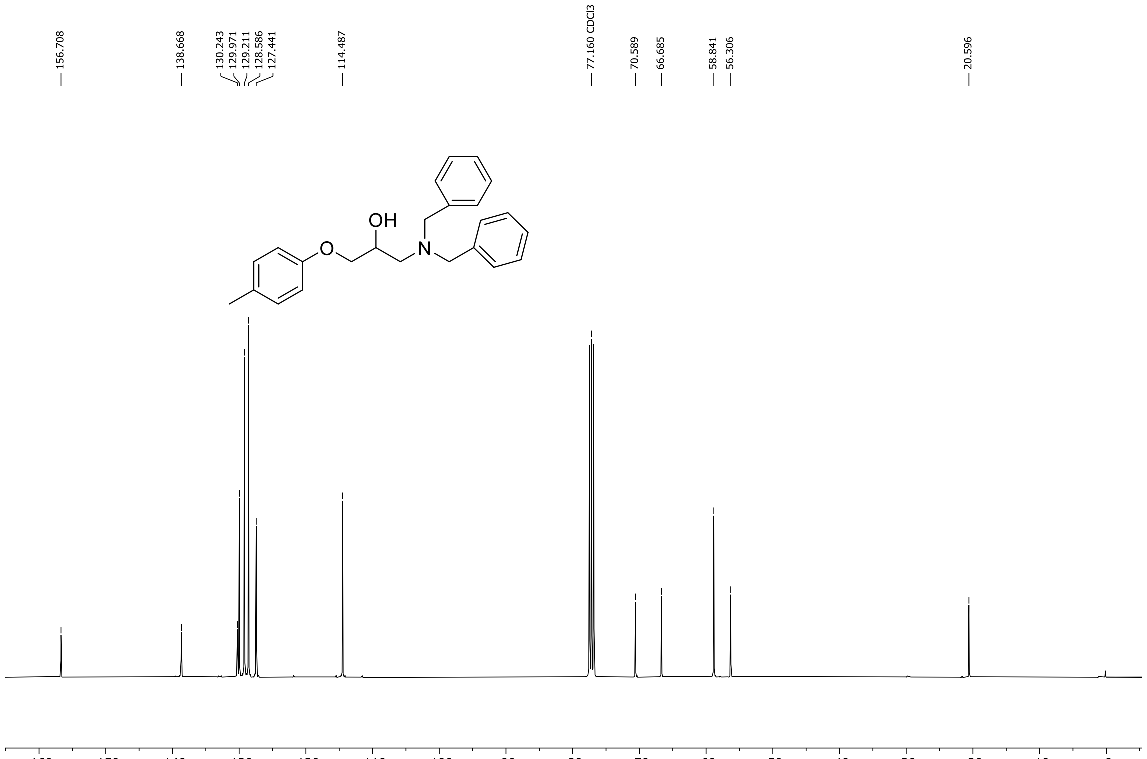

1-(Dibenzylamino)-3-(*p*-tolyloxy)propan-2-ol (8)

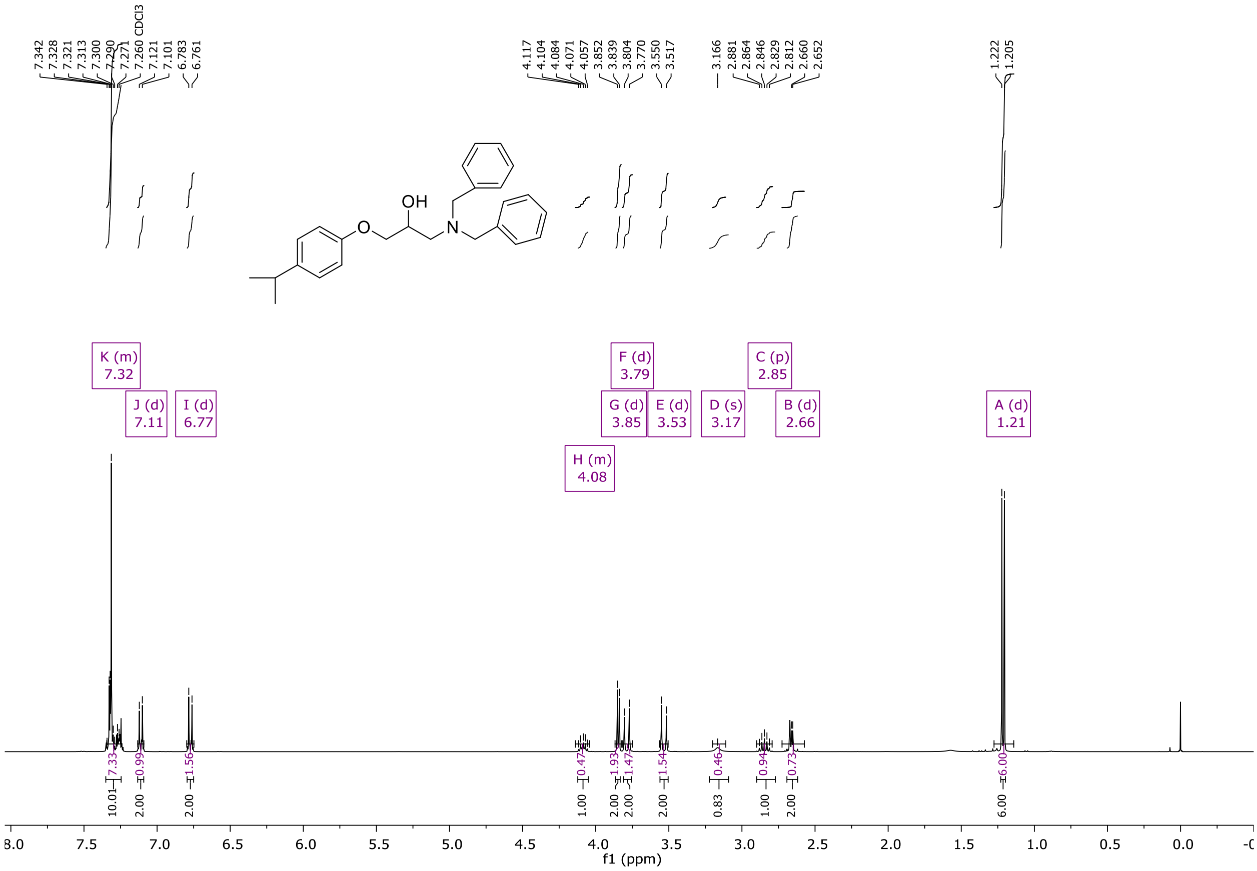

1-(Dibenzylamino)-3-(4-isopropylphenoxy)propan-2-ol (9)

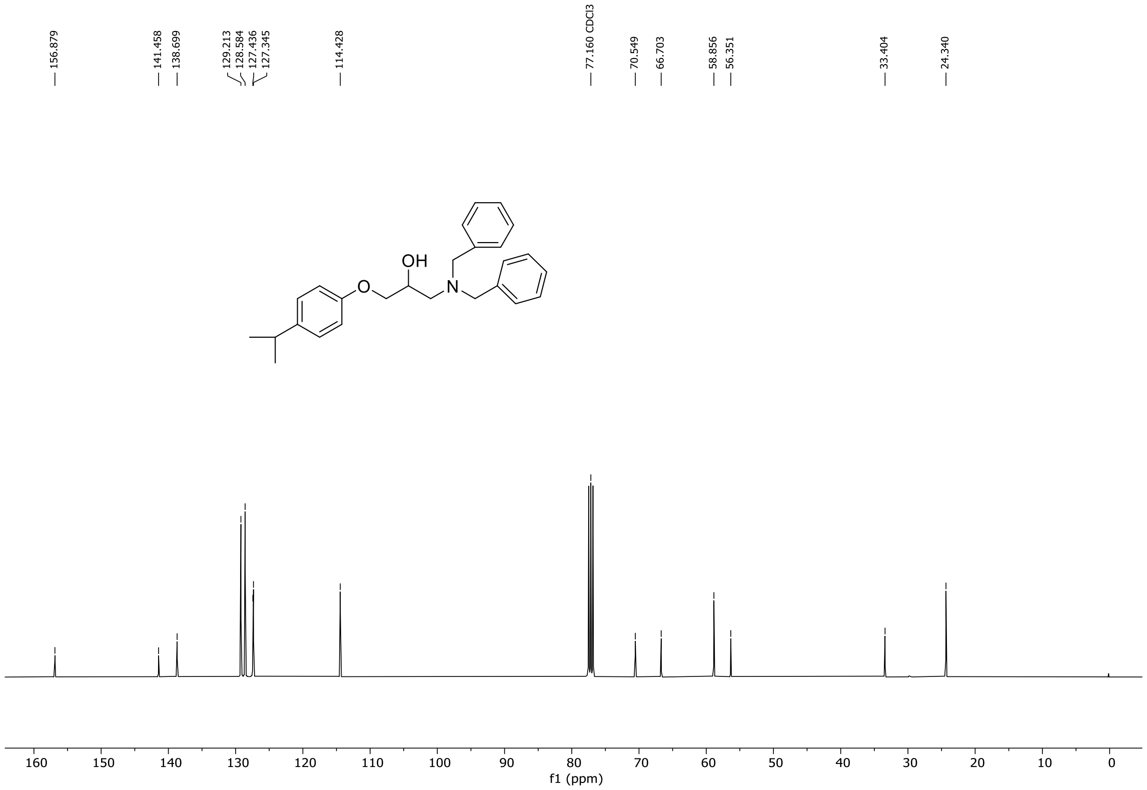

1-(Dibenzylamino)-3-(4-isopropylphenoxy)propan-2-ol (9)

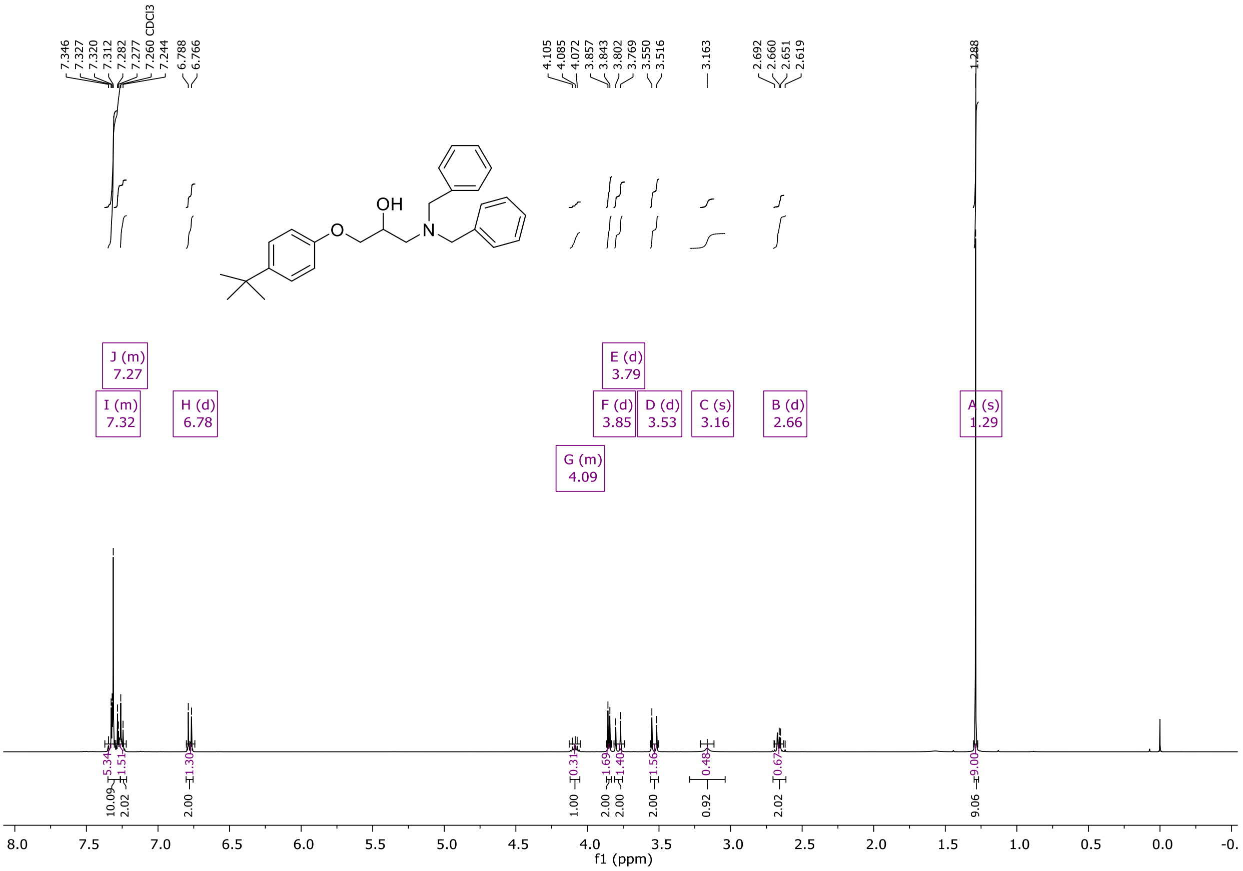

1-[4-(*Tert*-butyl)phenoxy]-3-(dibenzylamino)propan-2-ol (10)

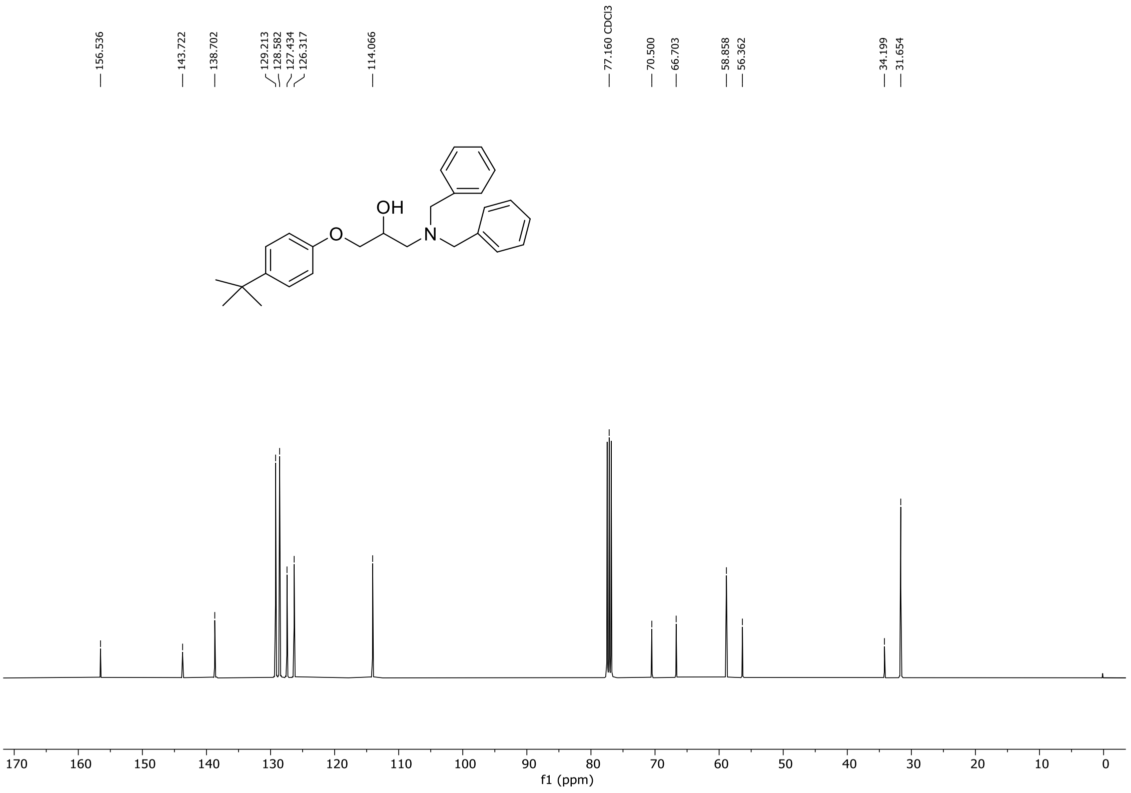

1-[4-(*Tert*-butyl)phenoxy]-3-(dibenzylamino)propan-2-ol (10)

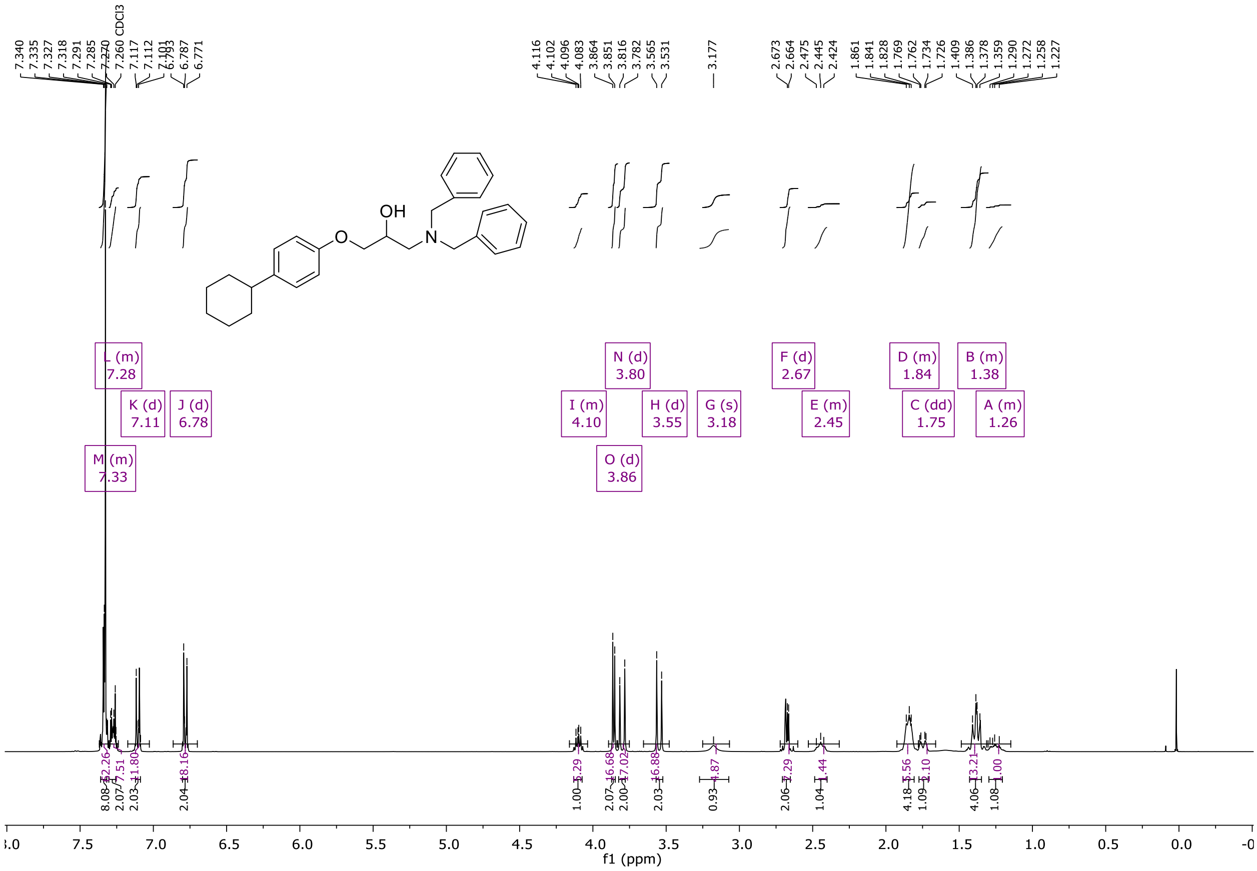

1-(4-Cyclohexylphenoxy)-3-(dibenzylamino)propan-2-ol (11)

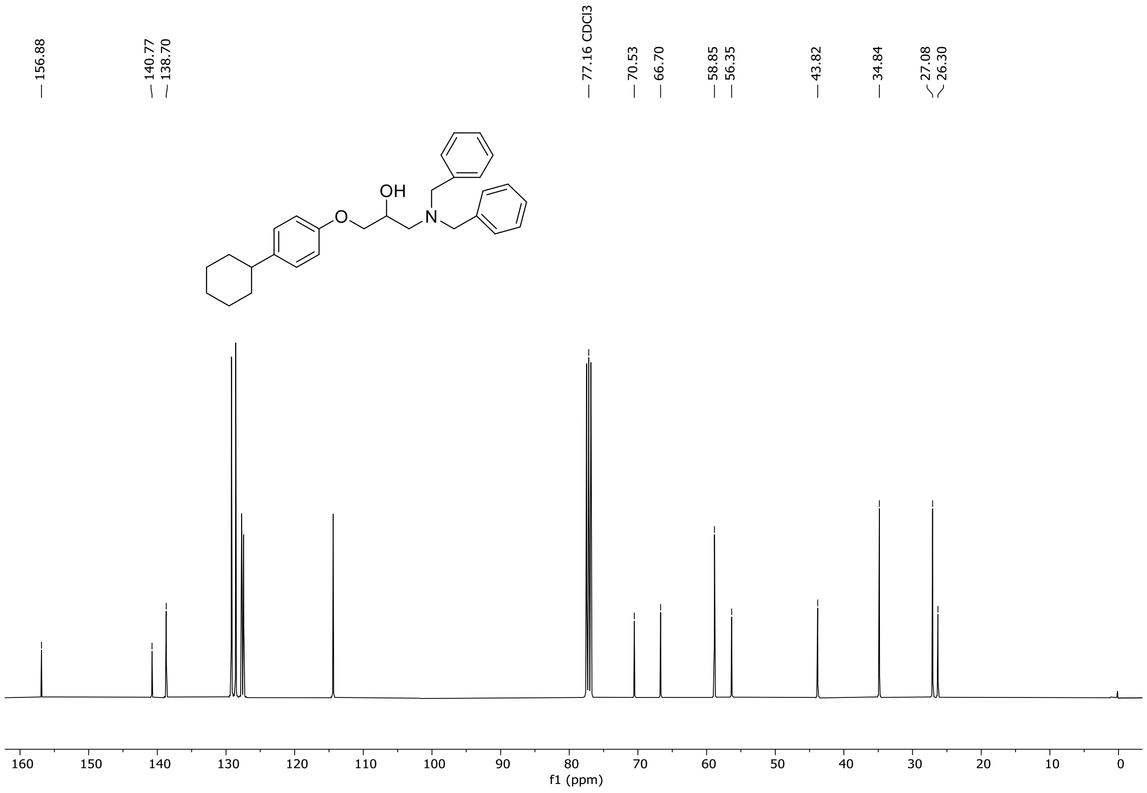

1-(4-Cyclohexylphenoxy)-3-(dibenzylamino)propan-2-ol (11)

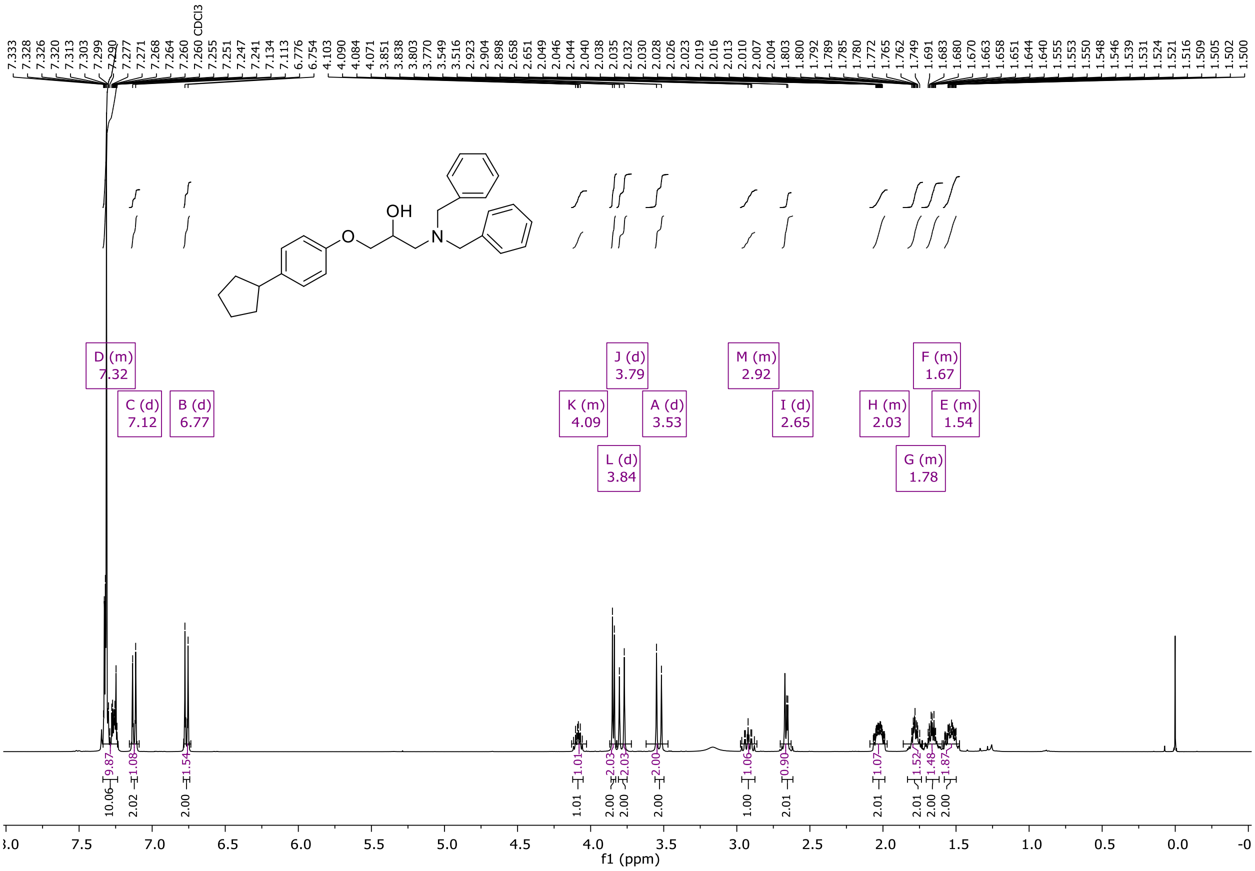

1-(4-Cyclopentylphenoxy)-3-(dibenzylamino)propan-2-ol (12)

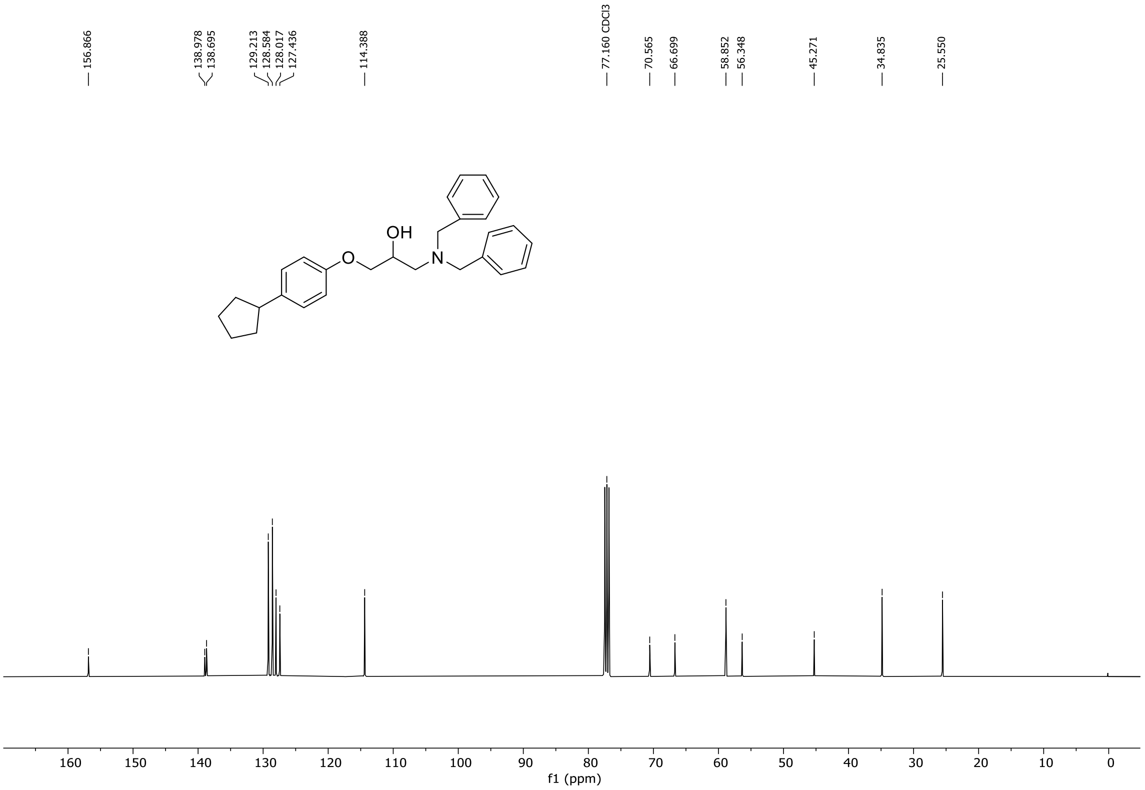

1-(4-Cyclopentylphenoxy)-3-(dibenzylamino)propan-2-ol (12)

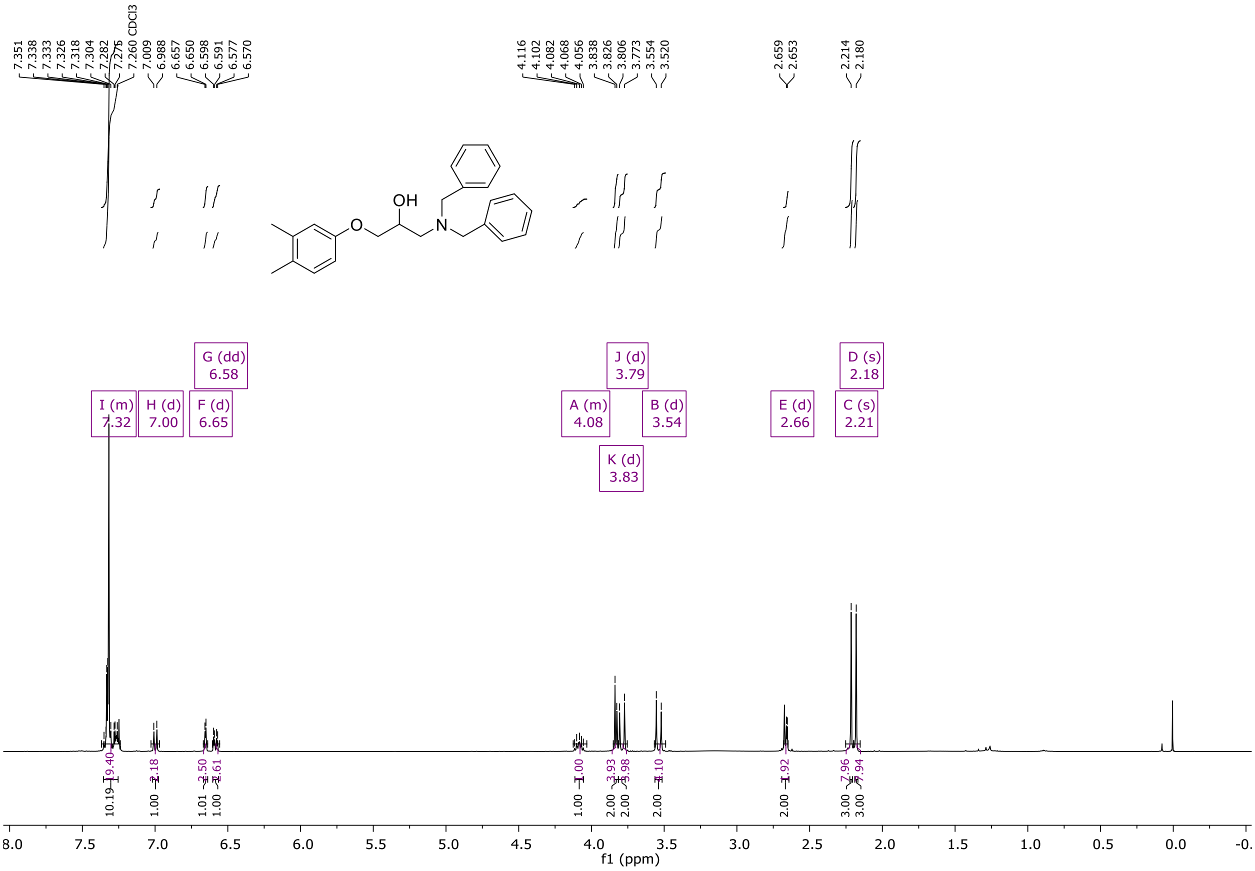

1-(Dibenzylamino)-3-(3,4-dimethylphenoxy)propan-2-ol (13)

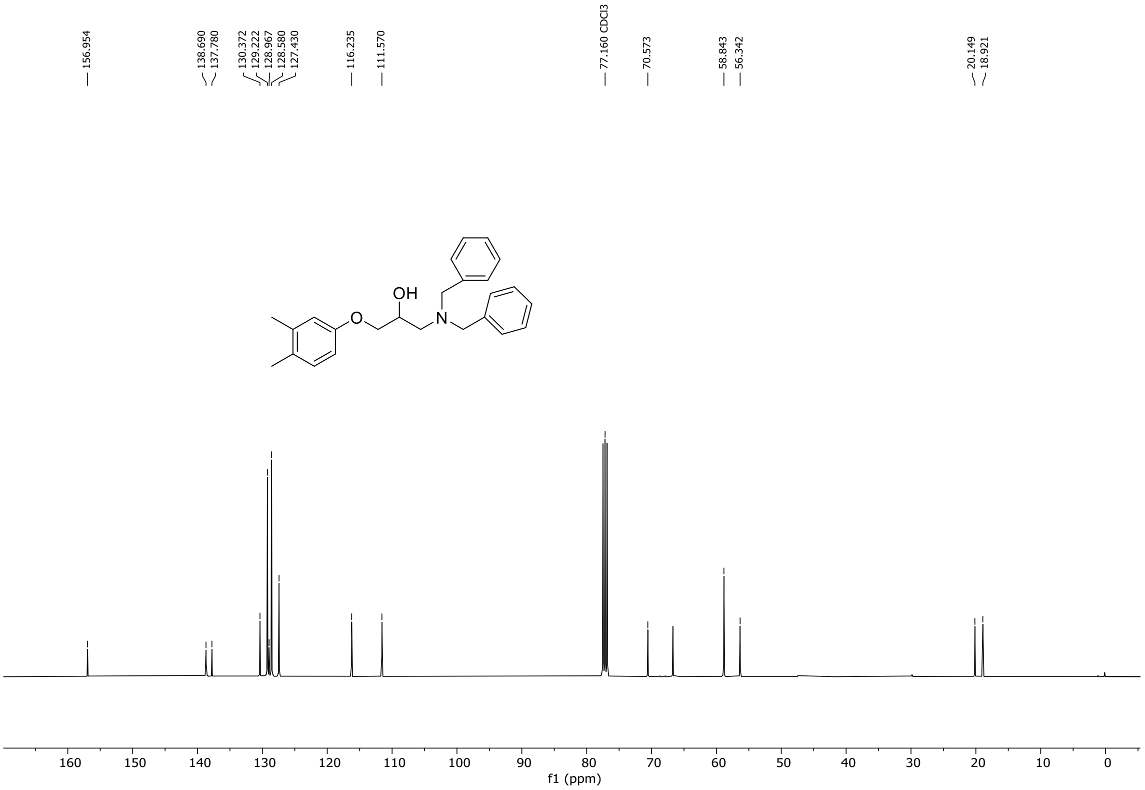

1-(Dibenzylamino)-3-(3,4-dimethylphenoxy)propan-2-ol (13)

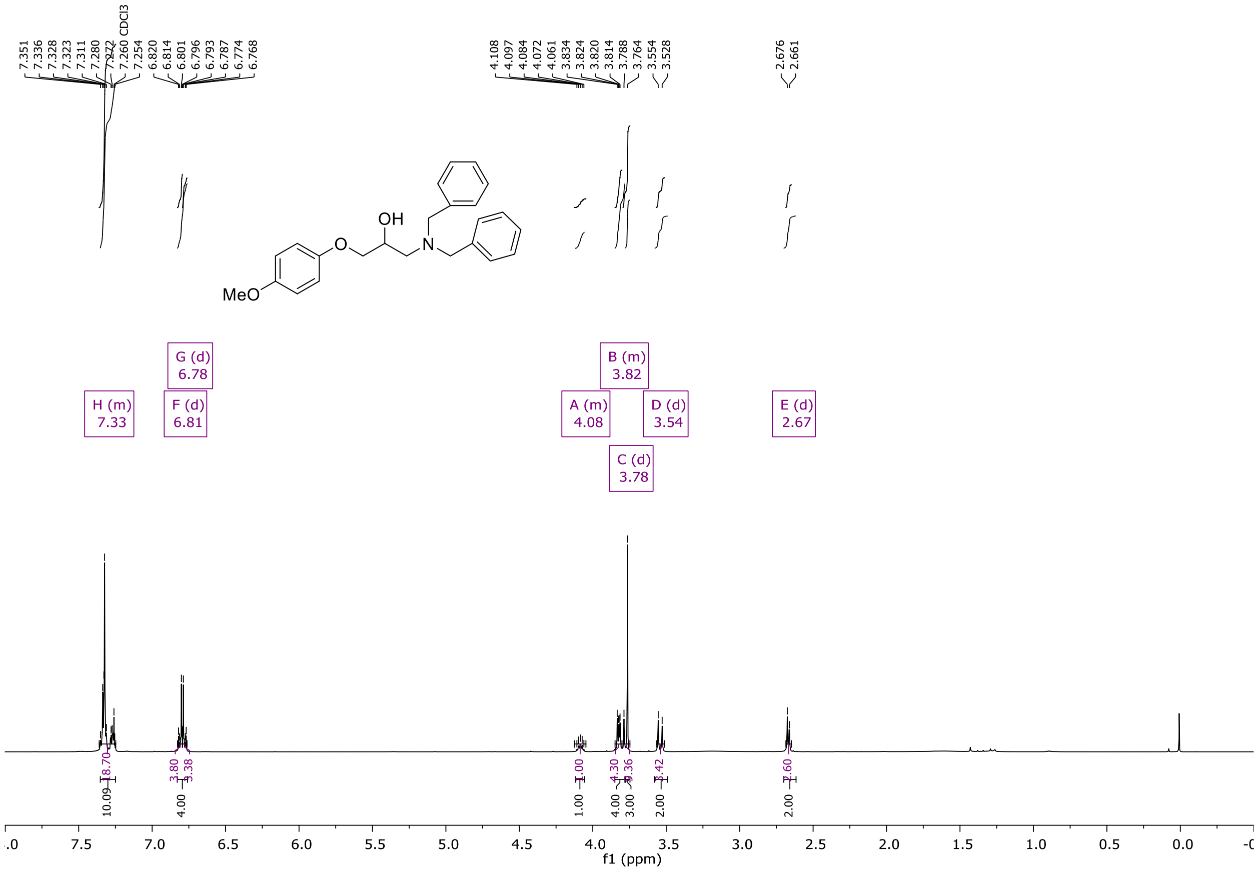

1-(Dibenzylamino)-3-(4-methoxyphenoxy)propan-2-ol (14)

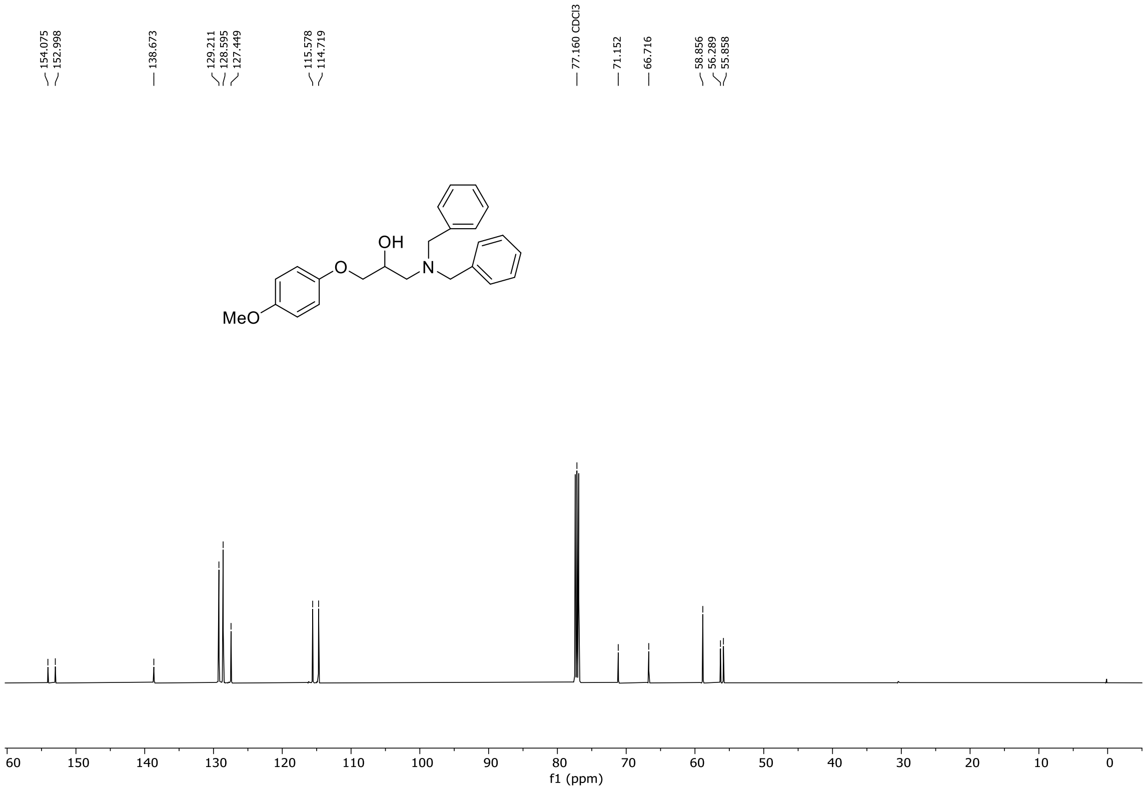

1-(Dibenzylamino)-3-(4-methoxyphenoxy)propan-2-ol (14)

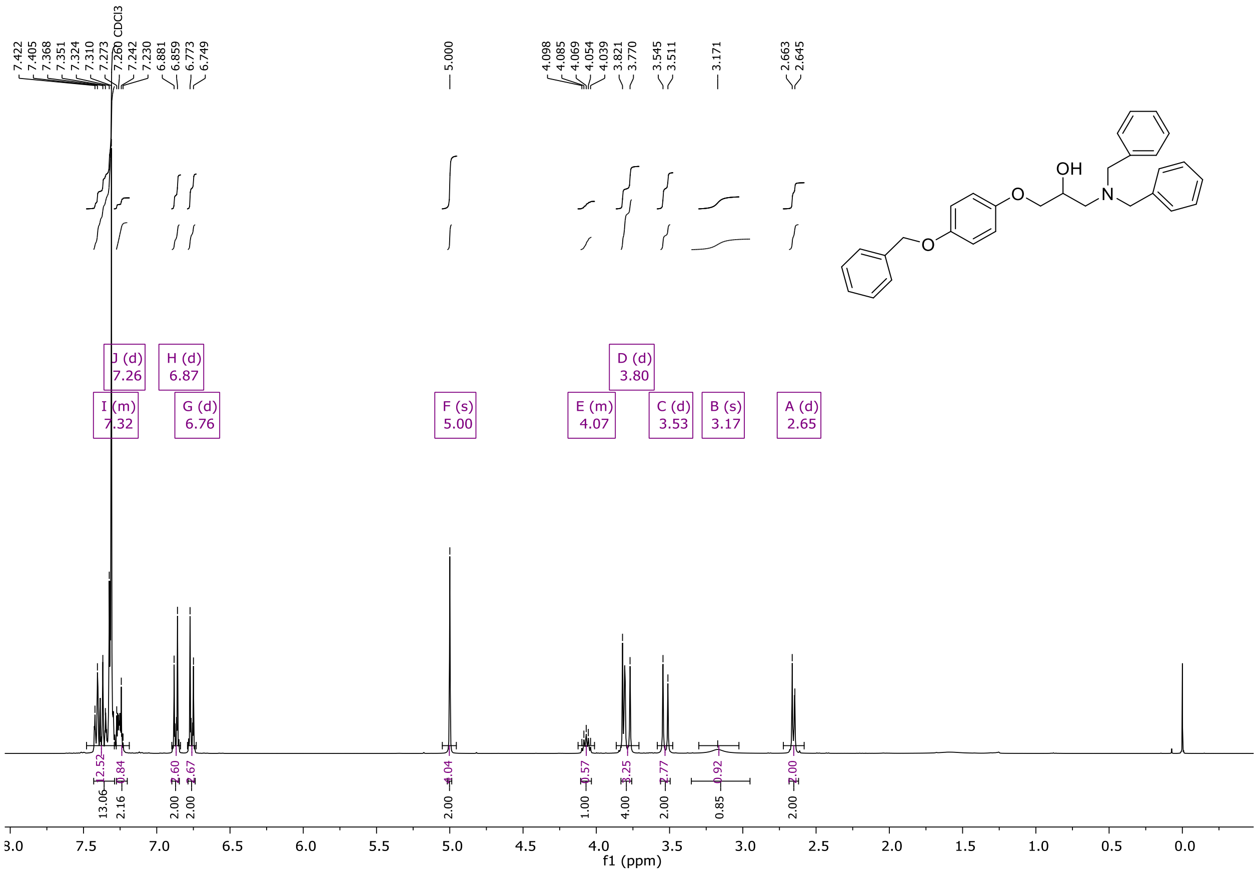

1-[4-(Benzyloxy)phenoxy]-3-(dibenzylamino)propan-2-ol (15)

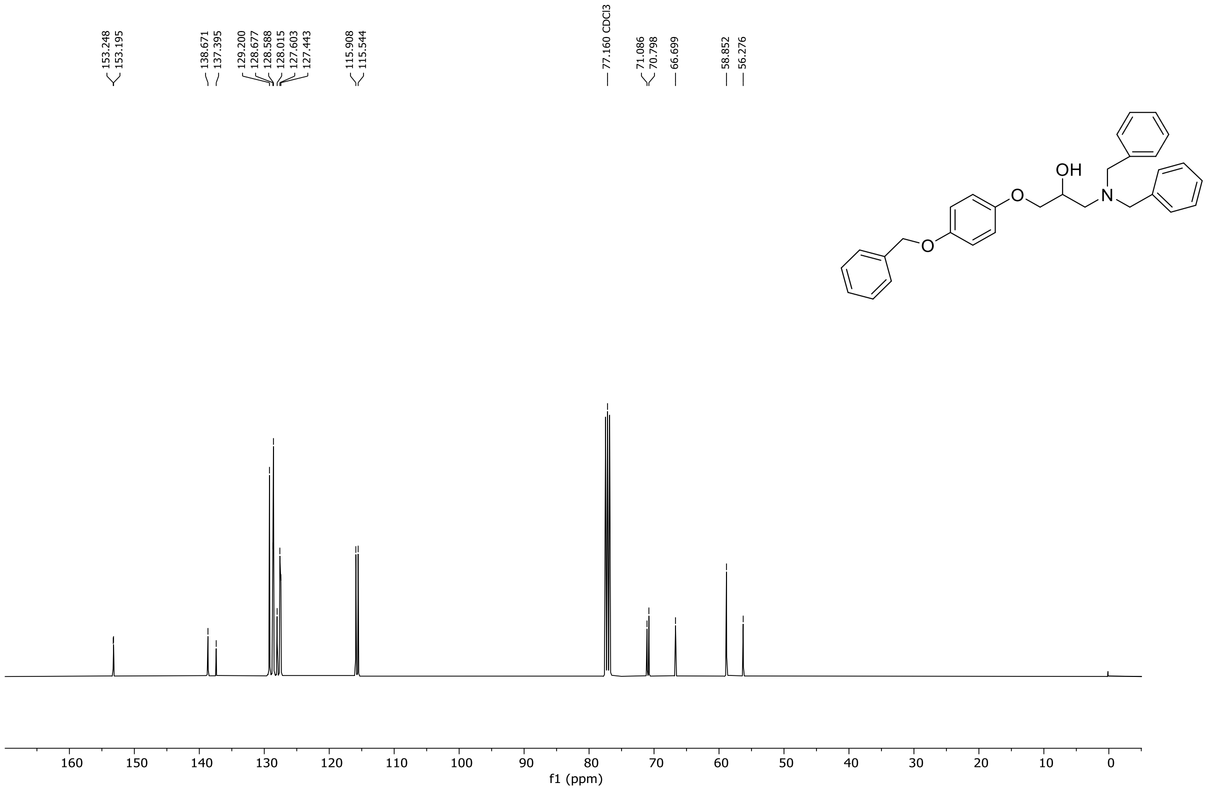

1-(4-(Benzyloxy)phenoxy)-3-(dibenzylamino)propan-2-ol (15)

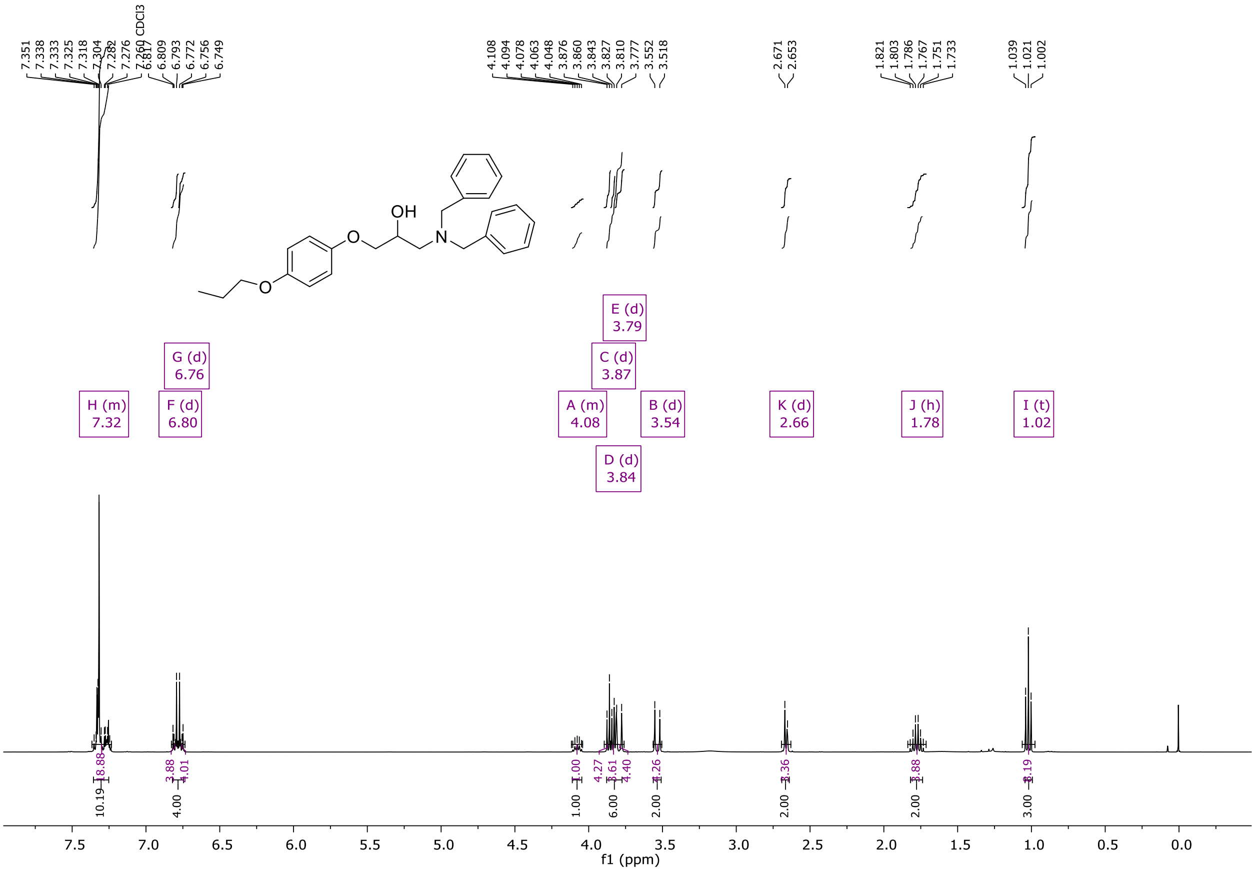

1-(Dibenzylamino)-3-(4-propoxyphenoxy)propan-2-ol (16)

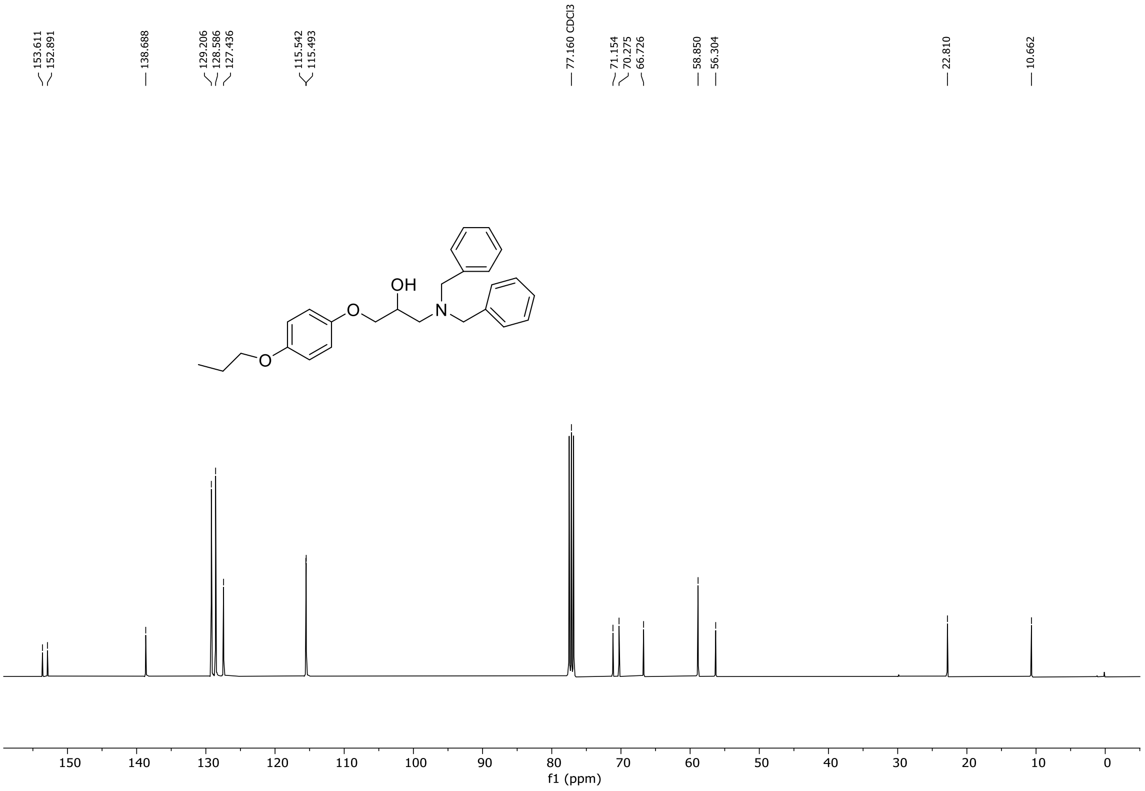

1-(Dibenzylamino)-3-(4-propoxyphenoxy)propan-2-ol (16)

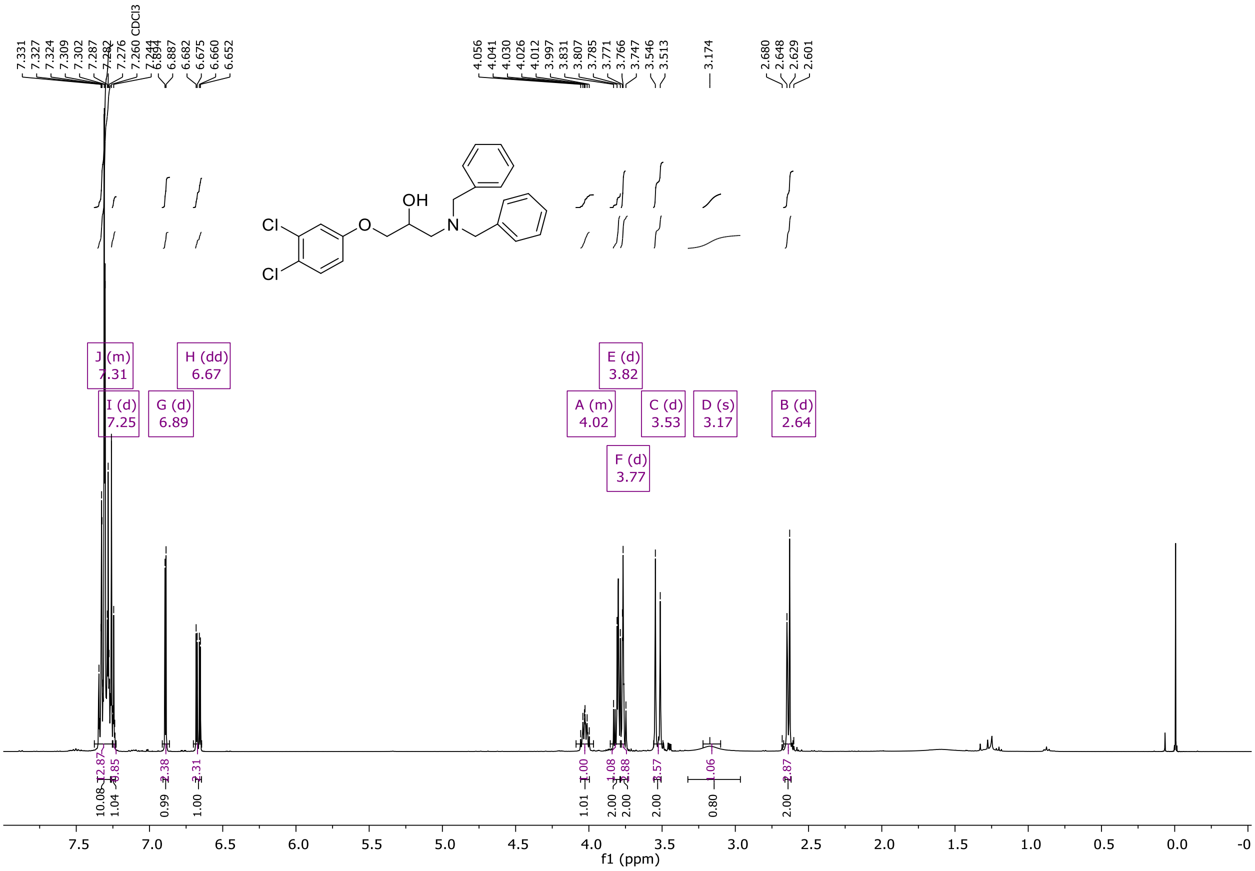

1-(Dibenzylamino)-3-(3,4-dichlorophenoxy)propan-2-ol (17)

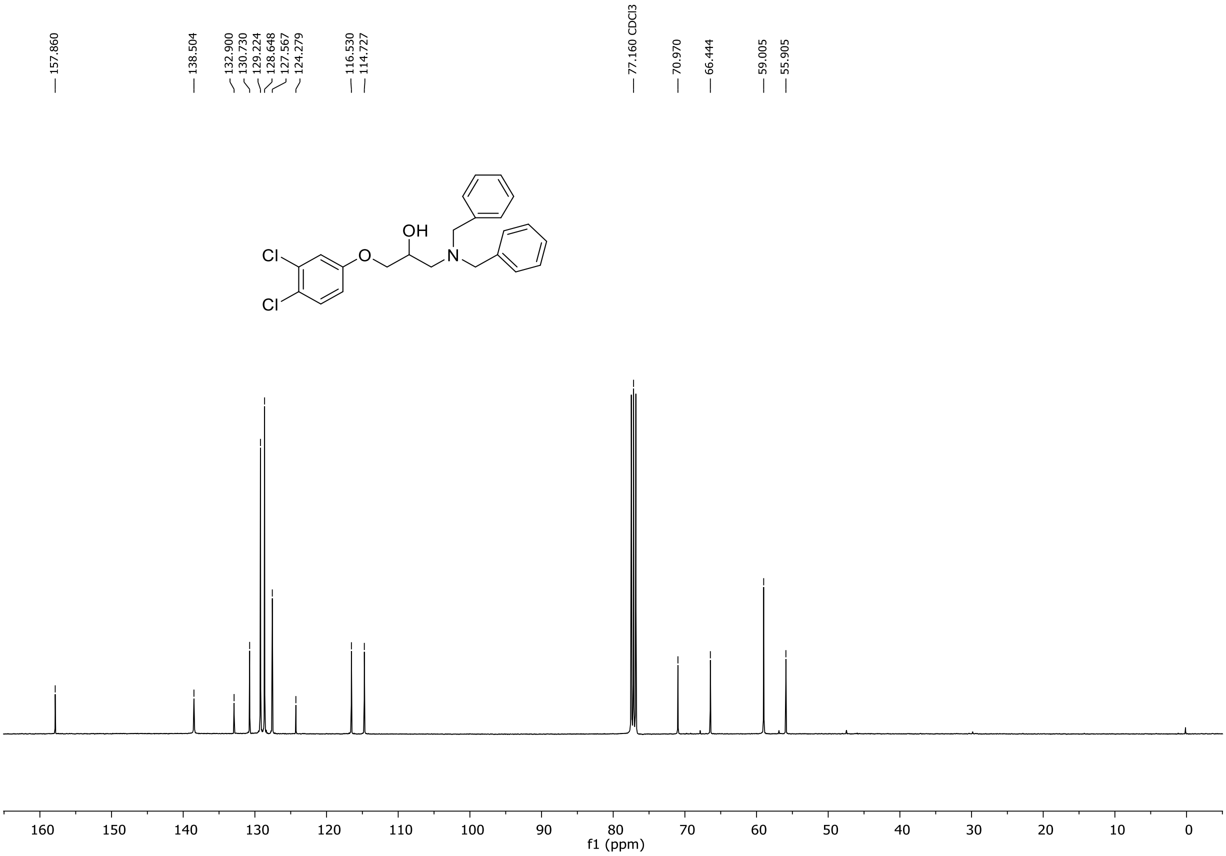

1-(Dibenzylamino)-3-(3,4-dichlorophenoxy)propan-2-ol (17)

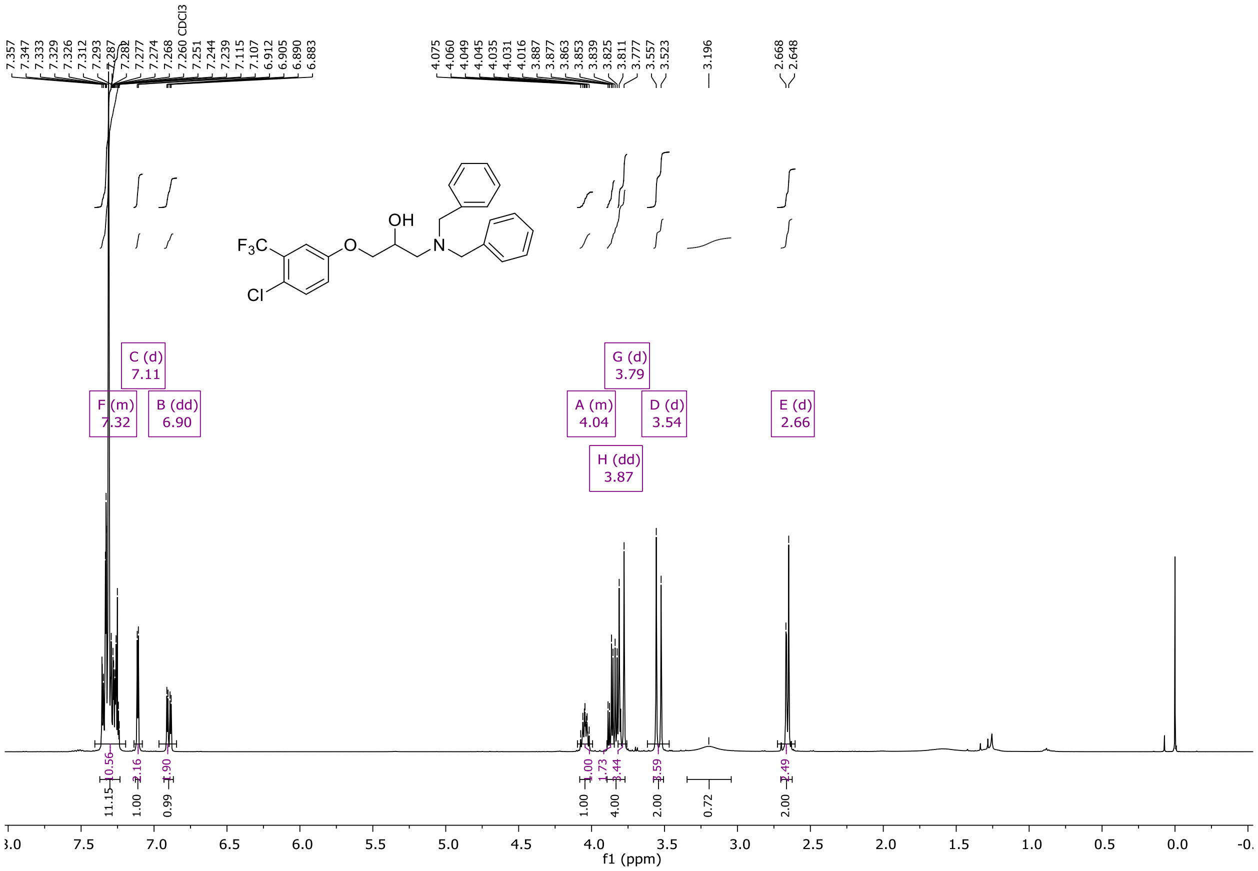

1-[4-Chloro-3-(trifluoromethyl)phenoxy]-3-(dibenzylamino)propan-2-ol (18)

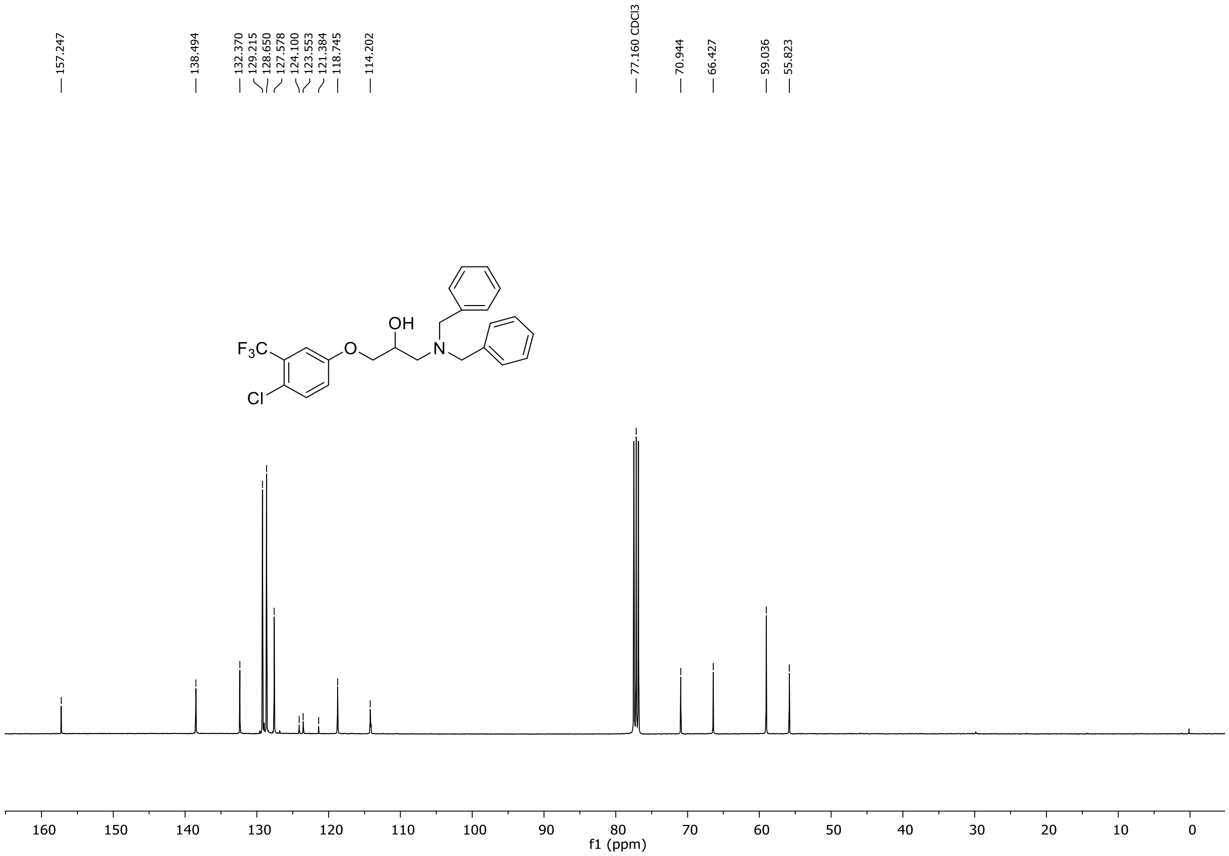

1-[4-Chloro-3-(trifluoromethyl)phenoxy]-3-(dibenzylamino)propan-2-ol (18)

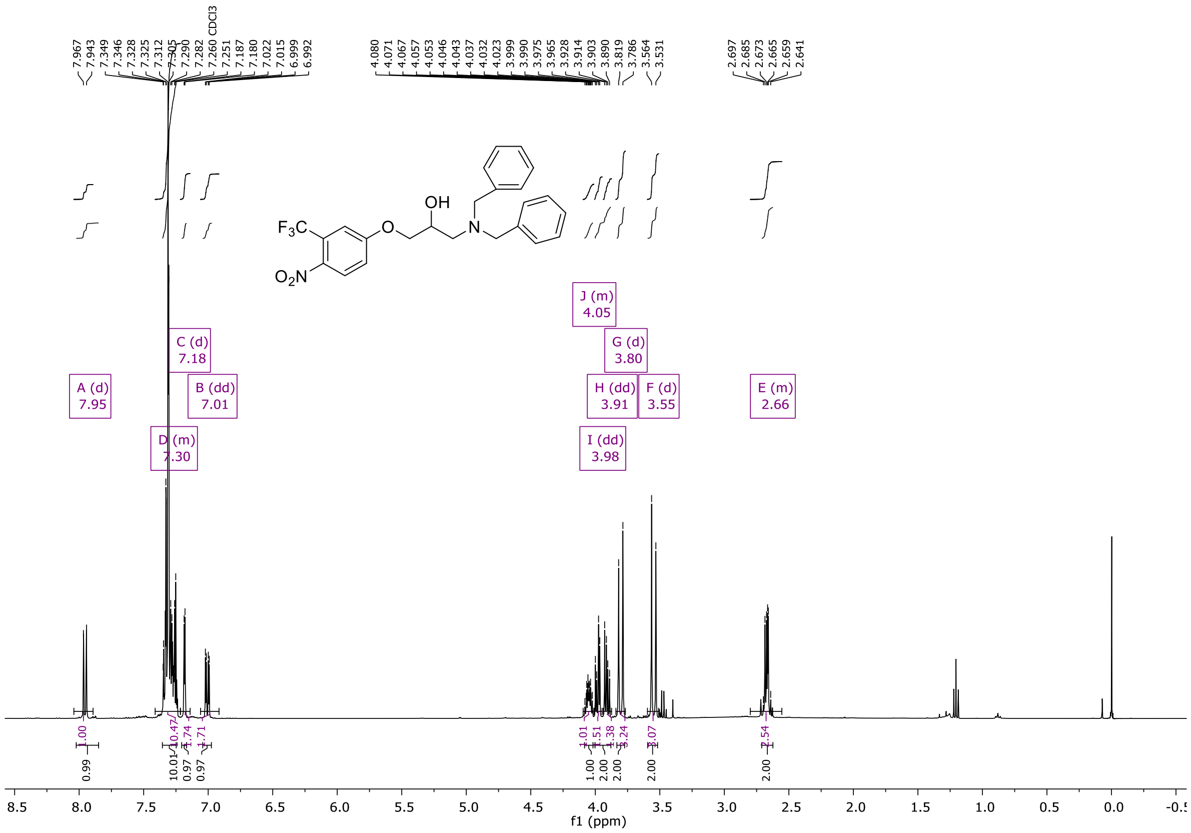

1-(Dibenzylamino)-3-[4-nitro-3-(trifluoromethyl)phenoxy]propan-2-ol (19)

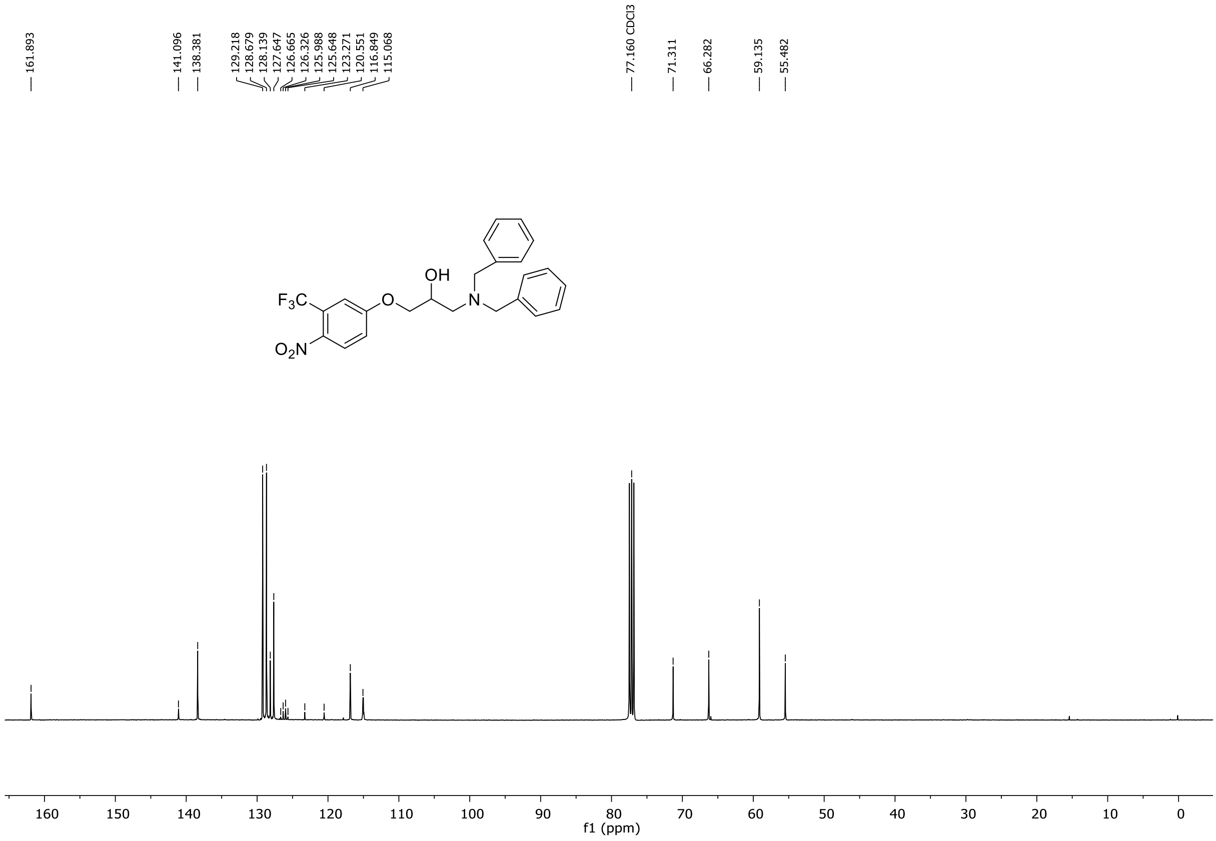

1-(Dibenzylamino)-3-[4-nitro-3-(trifluoromethyl)phenoxy]propan-2-ol (19)

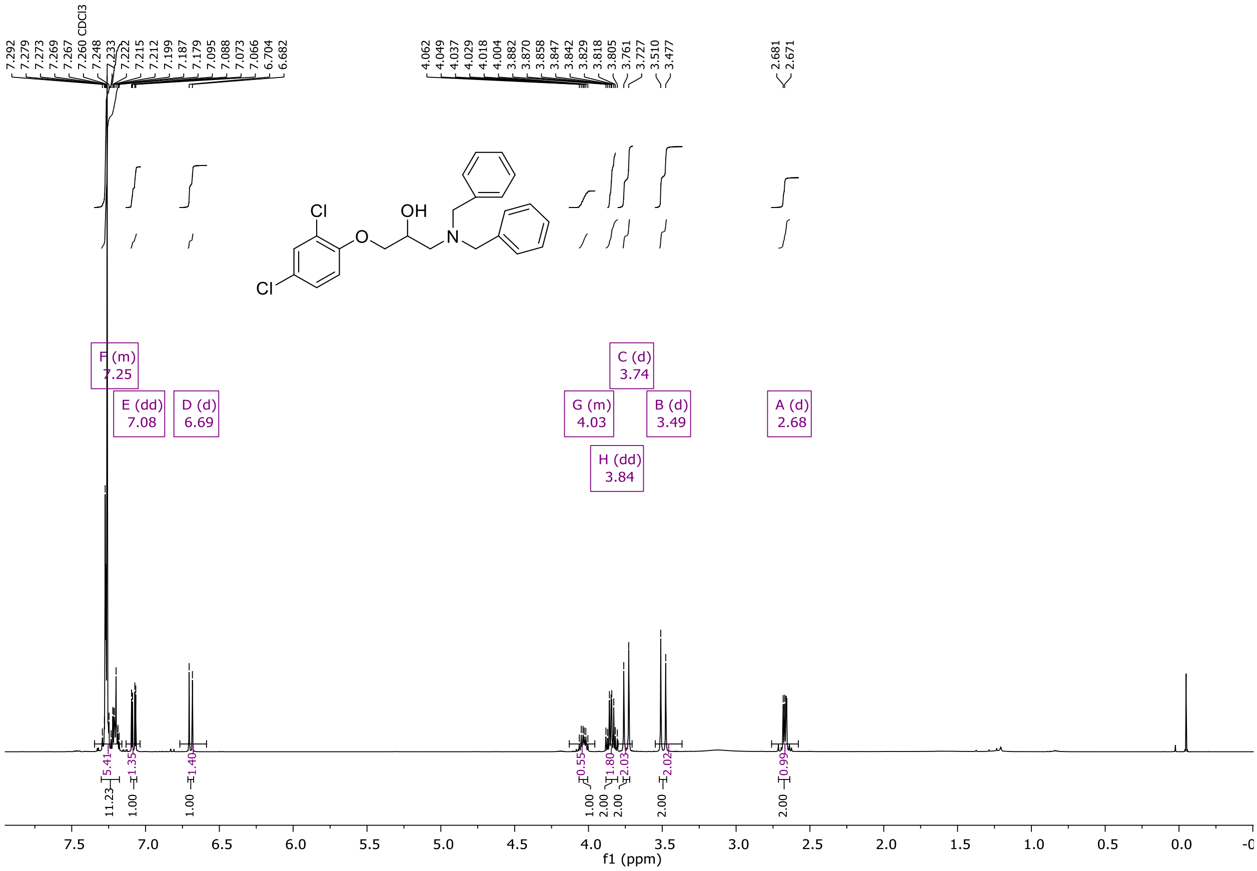

1-(Dibenzylamino)-3-(2,4-dichlorophenoxy)propan-2-ol (20)

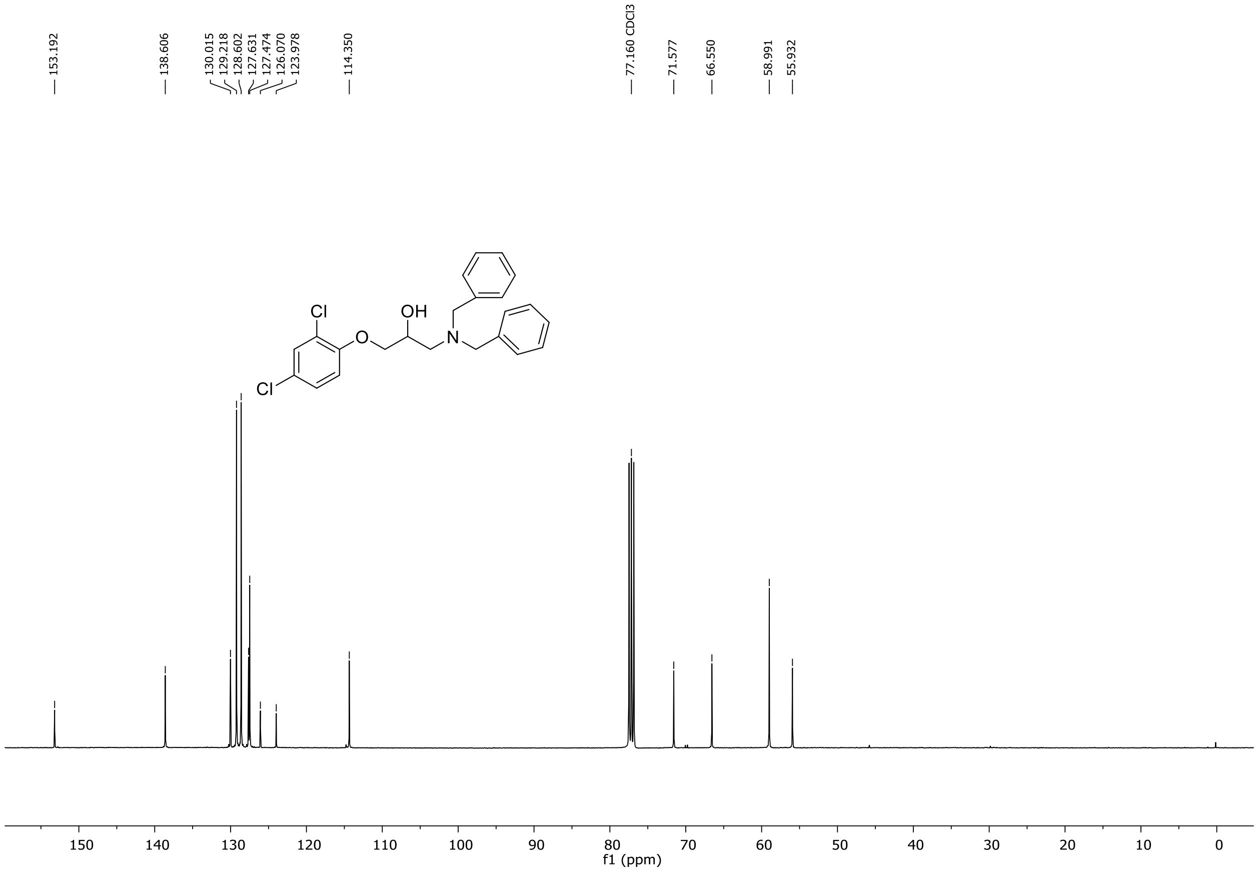

1-(Dibenzylamino)-3-(2,4-dichlorophenoxy)propan-2-ol (20)

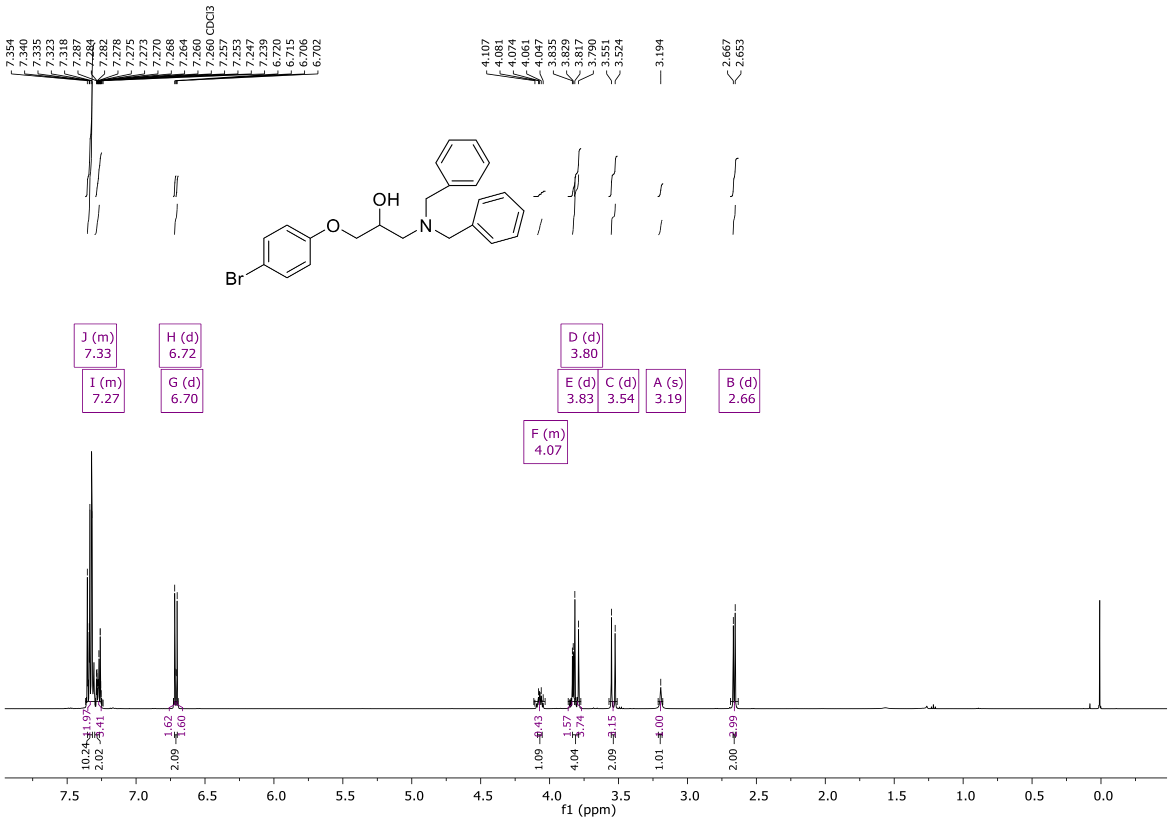

1-(4-Bromophenoxy)-3-(dibenzylamino)propan-2-ol (21)

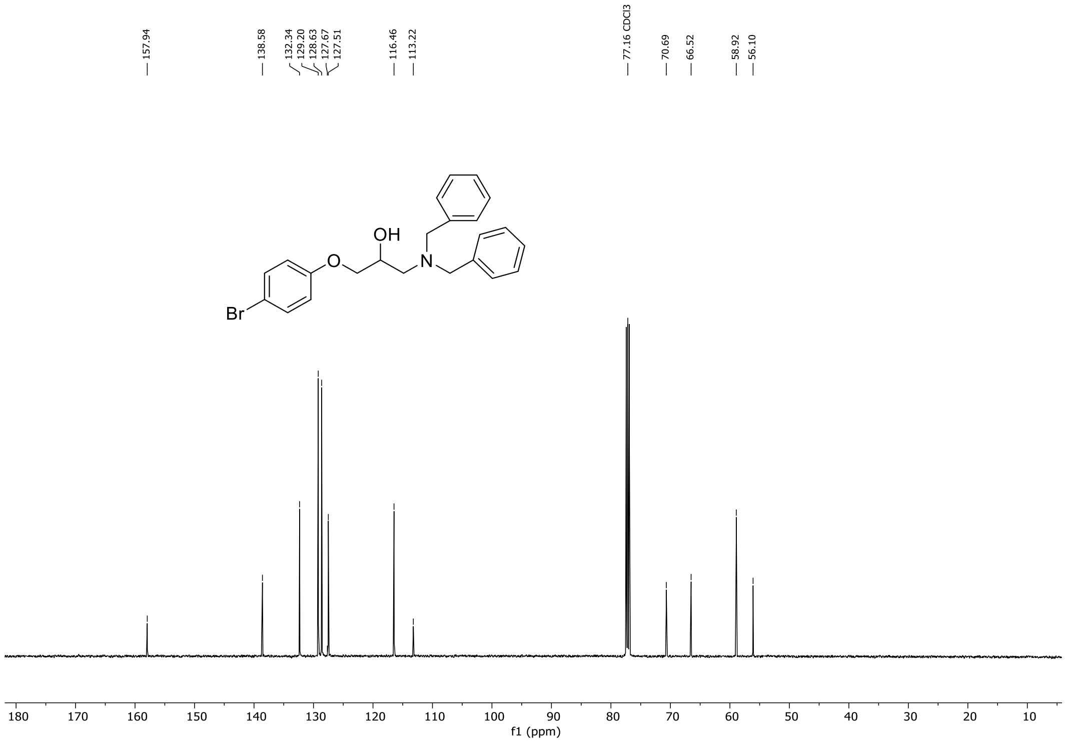

1-(4-Bromophenoxy)-3-(dibenzylamino)propan-2-ol (21)

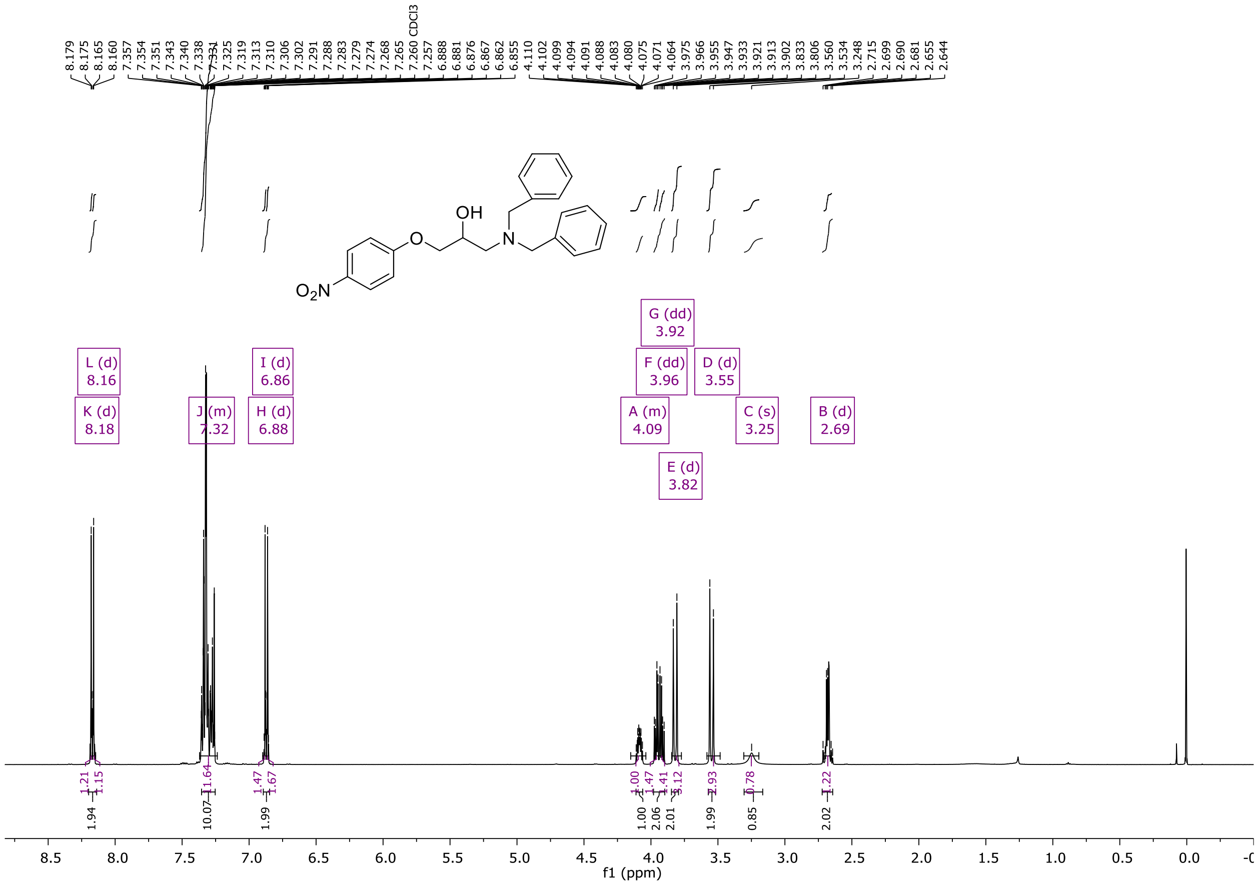

1-(Dibenzylamino)-3-(4-nitrophenoxy)propan-2-ol (22)

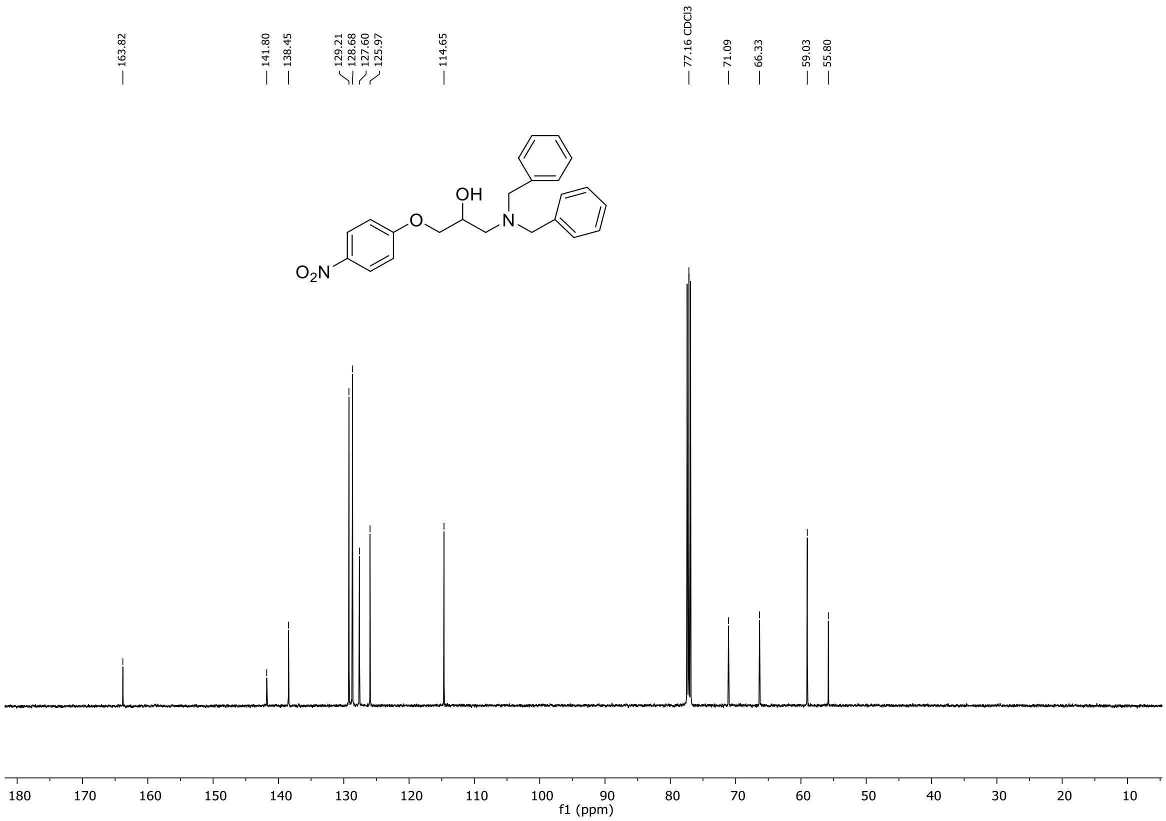

1-(Dibenzylamino)-3-(4-nitrophenoxy)propan-2-ol (22)

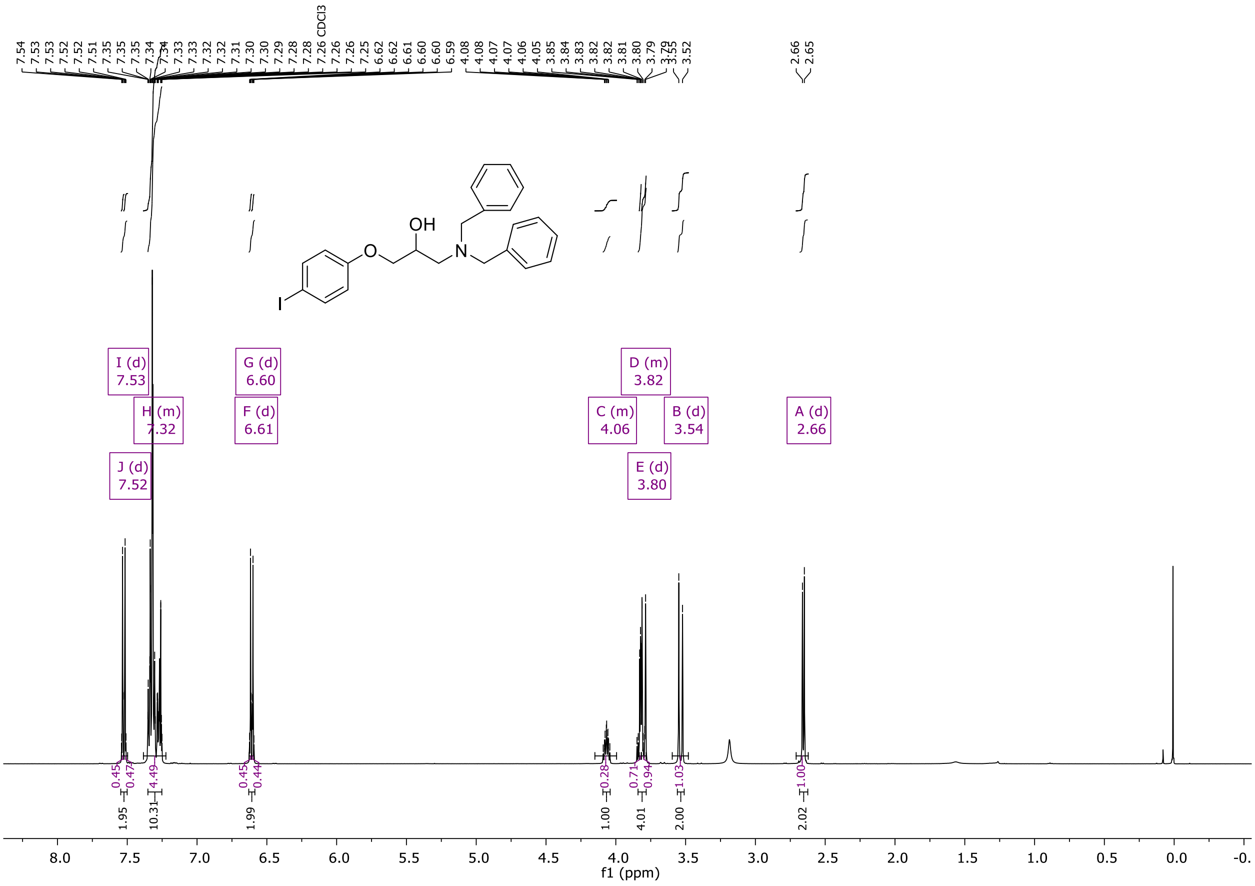

1-(Dibenzylamino)-3-(4-iodophenoxy)propan-2-ol (23)

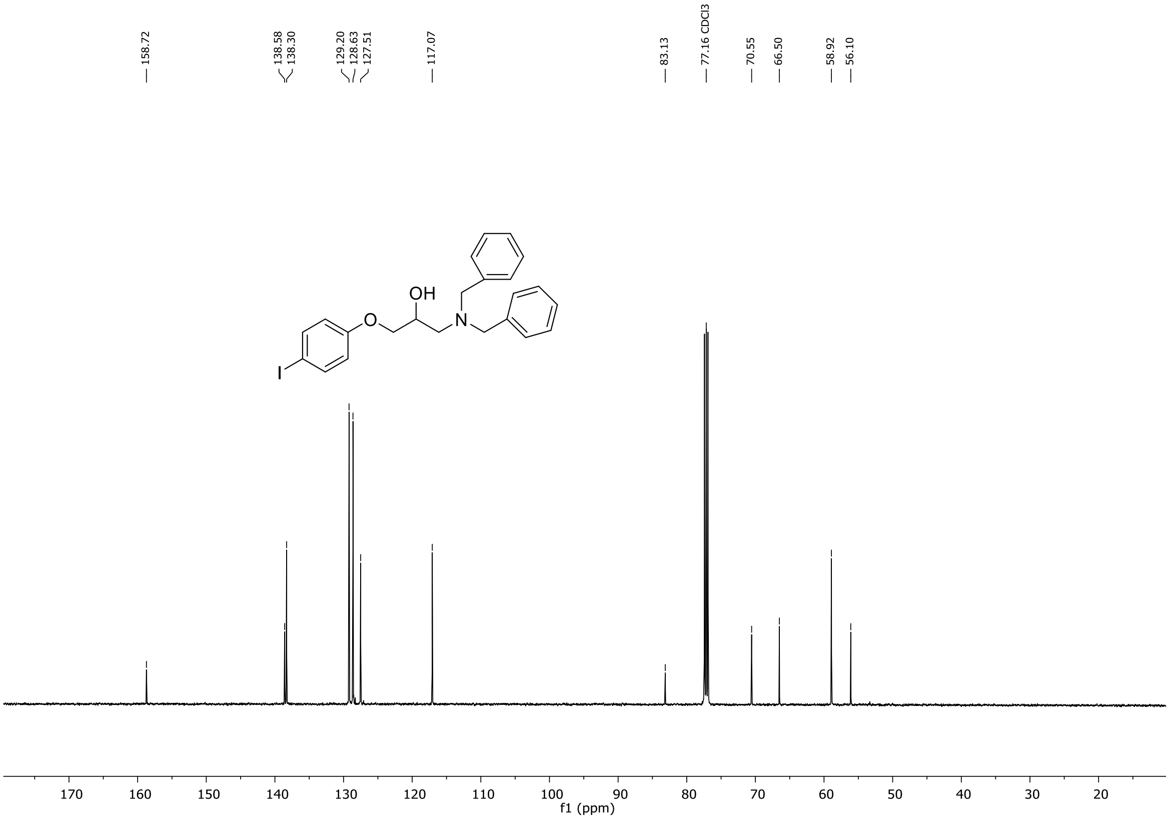

1-(Dibenzylamino)-3-(4-iodophenoxy)propan-2-ol (23)

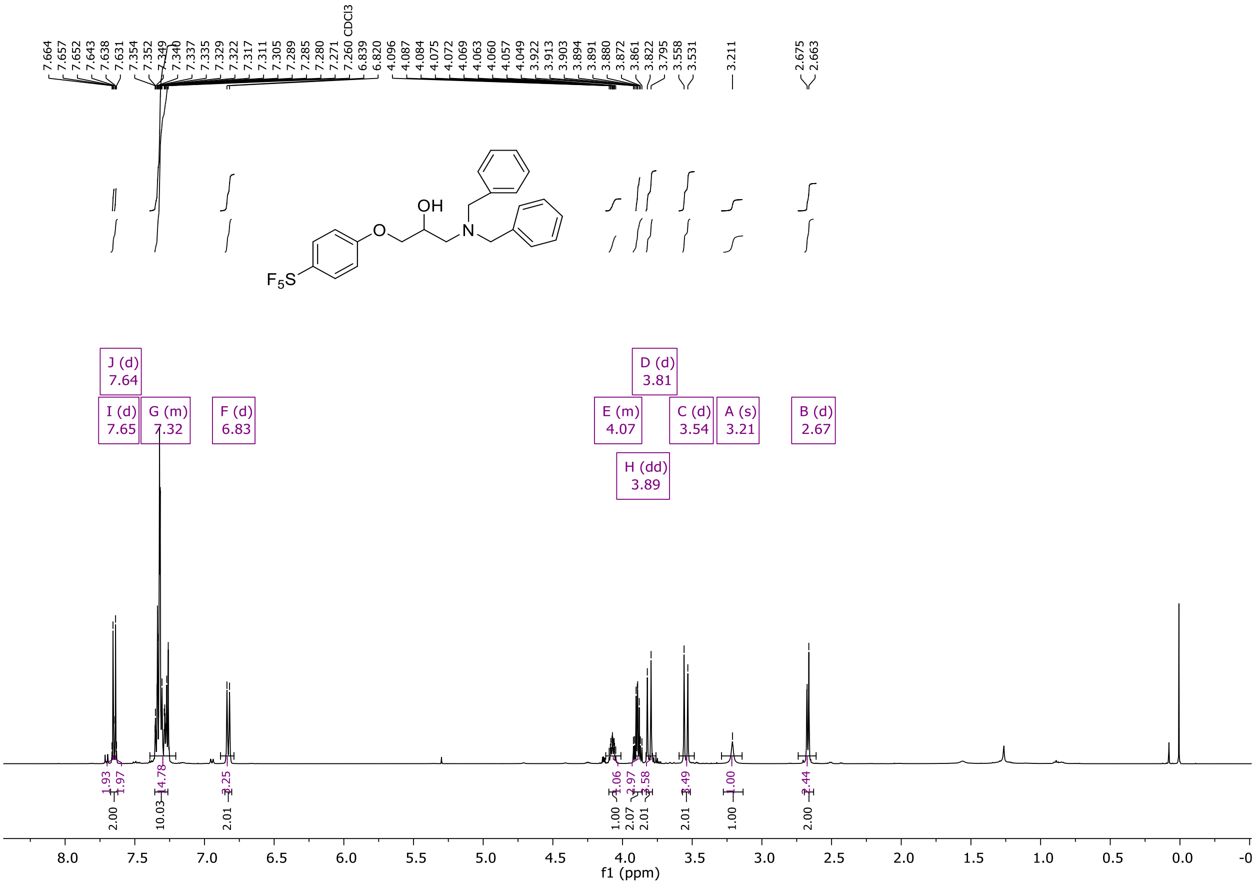

1-(Dibenzylamino)-3-[4-(pentafluoro-λ⁶-sulfaneyl)phenoxy]propan-2-ol (24)

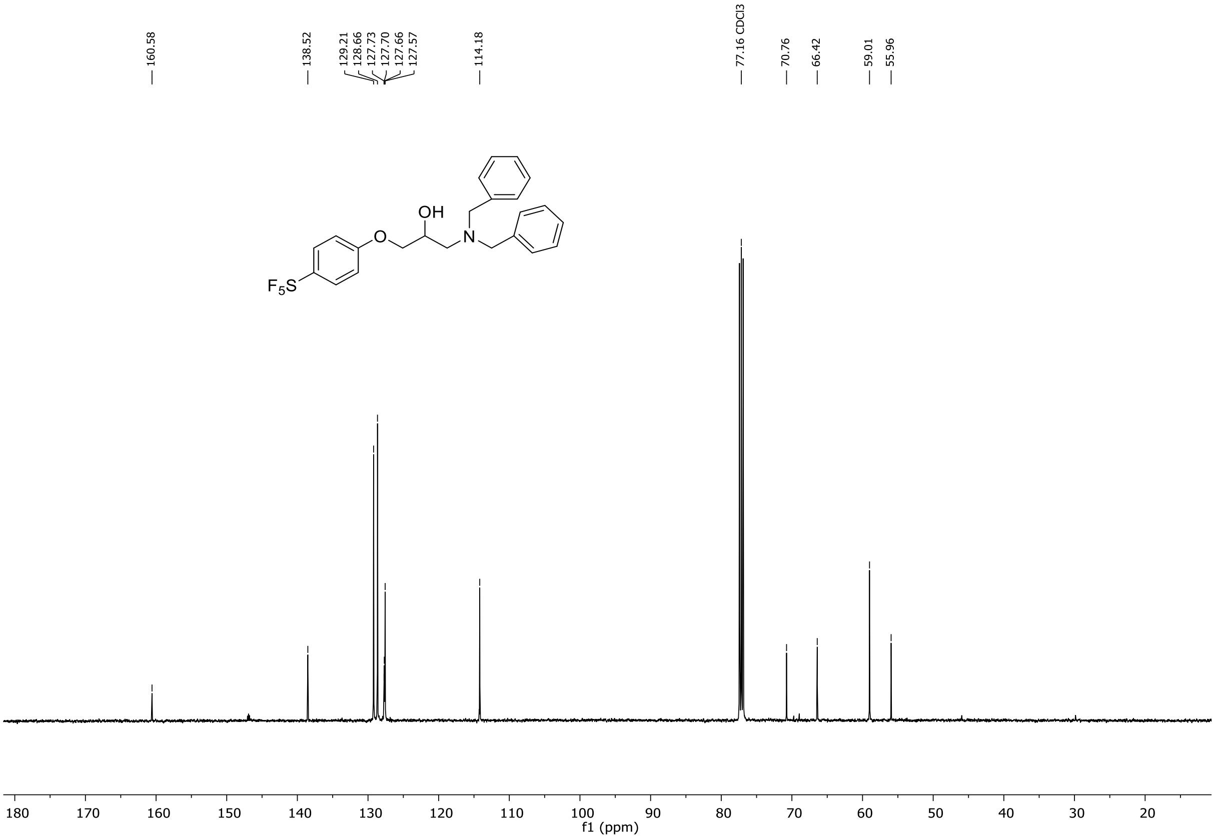

1-(Dibenzylamino)-3-[4-(pentafluoro-λ⁶-sulfaneyl)phenoxy]propan-2-ol (24)

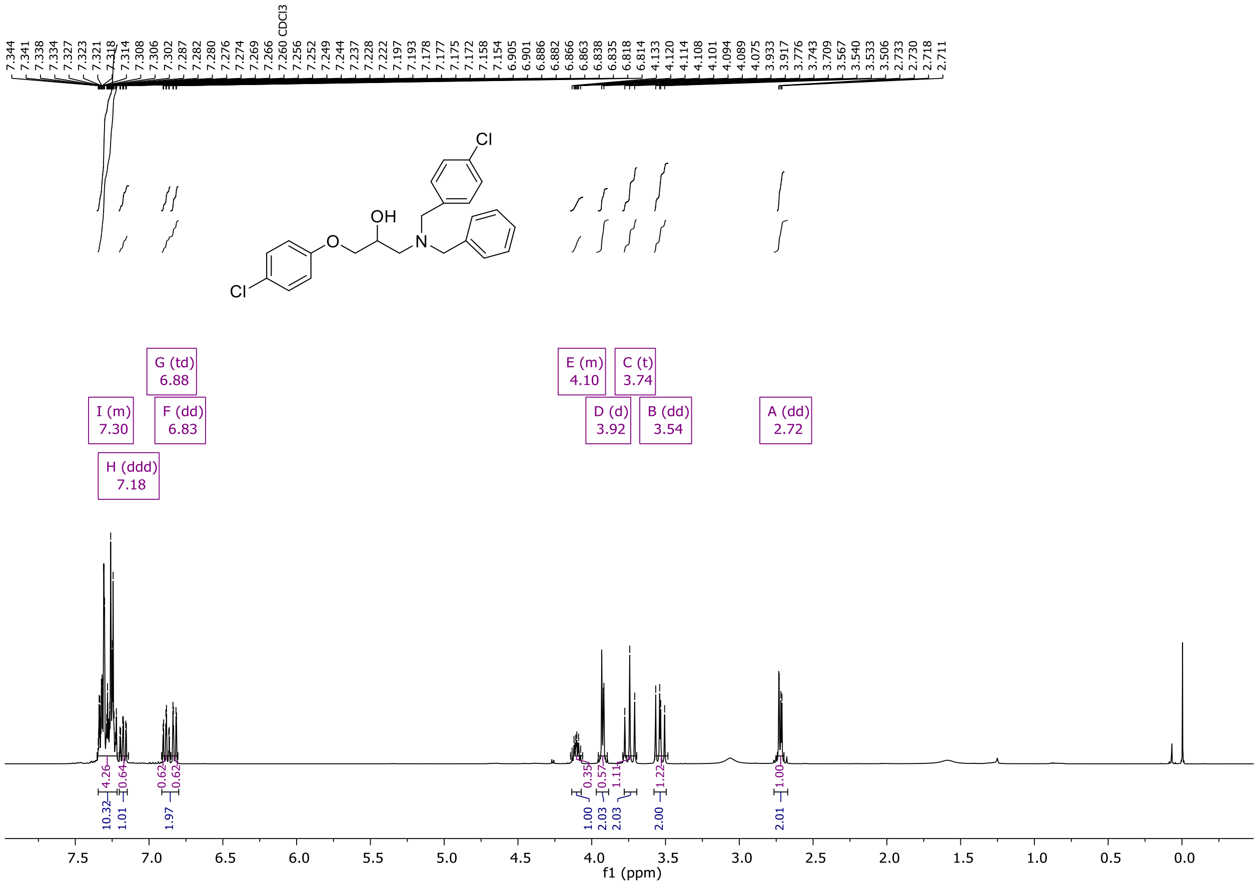

1-[Benzyl(4-chlorobenzyl)amino]-3-(4-chlorophenoxy)propan-2-ol (25)

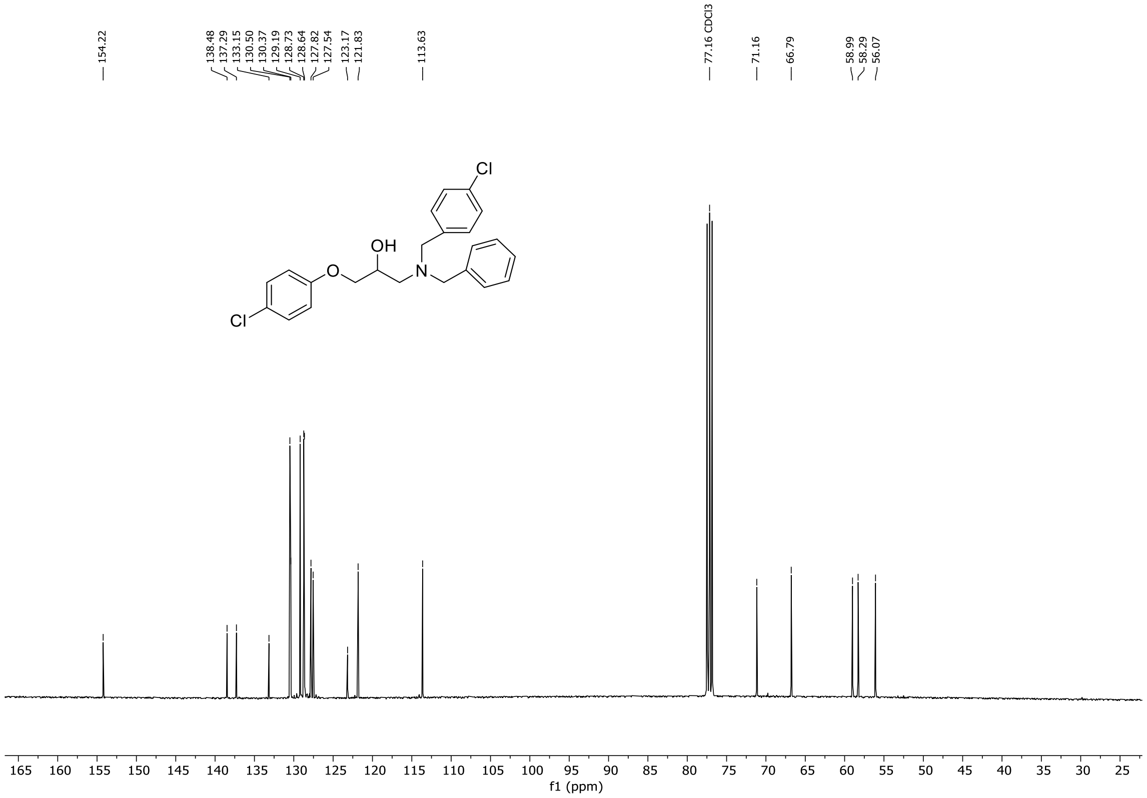

1-[Benzyl(4-chlorobenzyl)amino]-3-(4-chlorophenoxy)propan-2-ol (25)

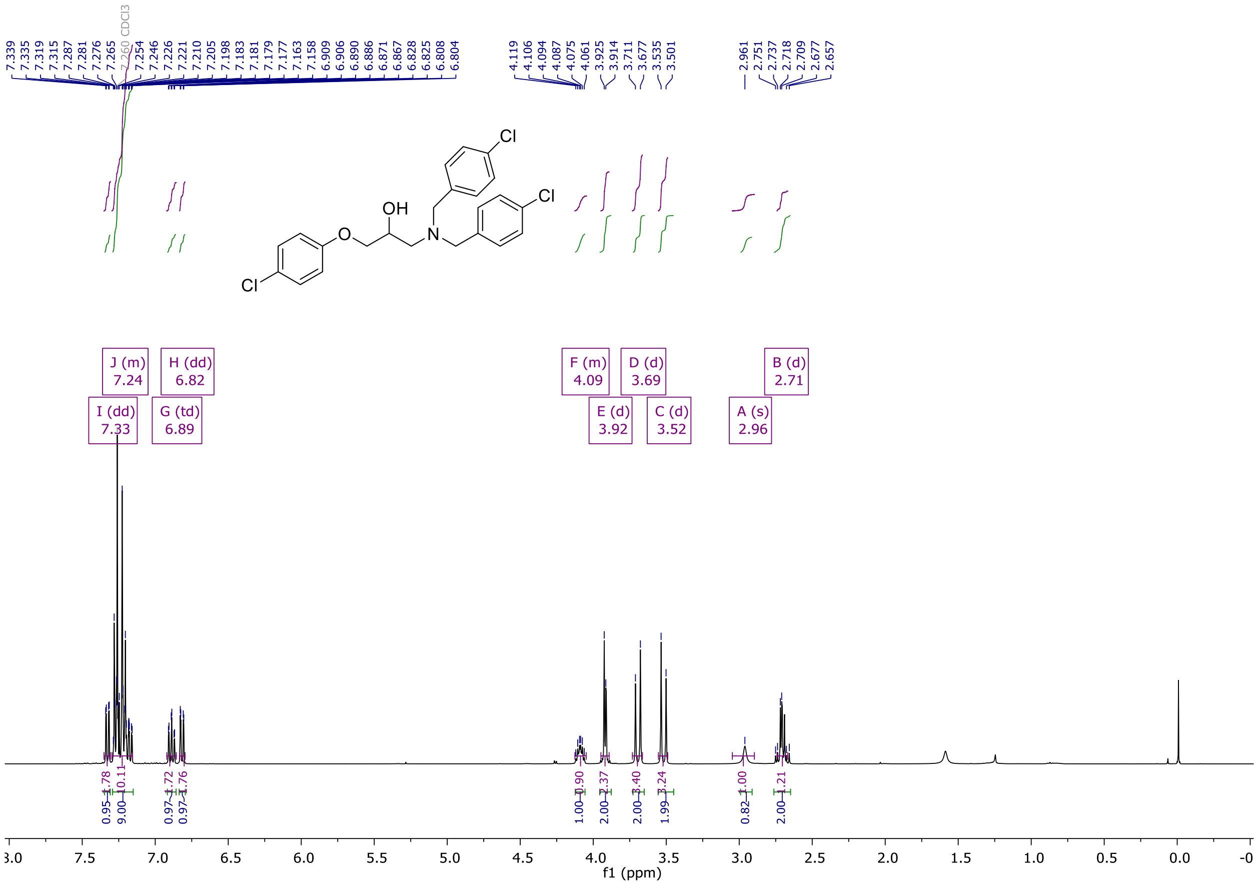

1-[Bis(4-chlorobenzyl)amino]-3-(4-chlorophenoxy)propan-2-ol (26)

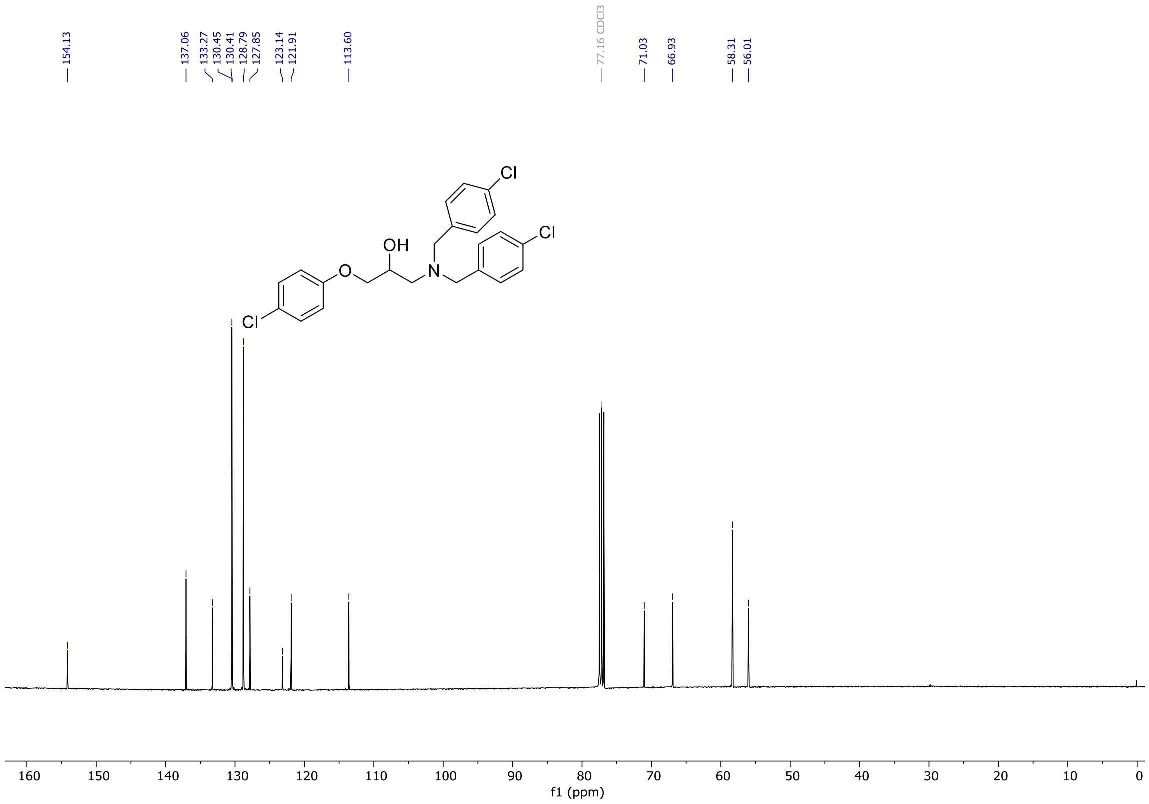

1-[Bis(4-chlorobenzyl)amino]-3-(4-chlorophenoxy)propan-2-ol (26)

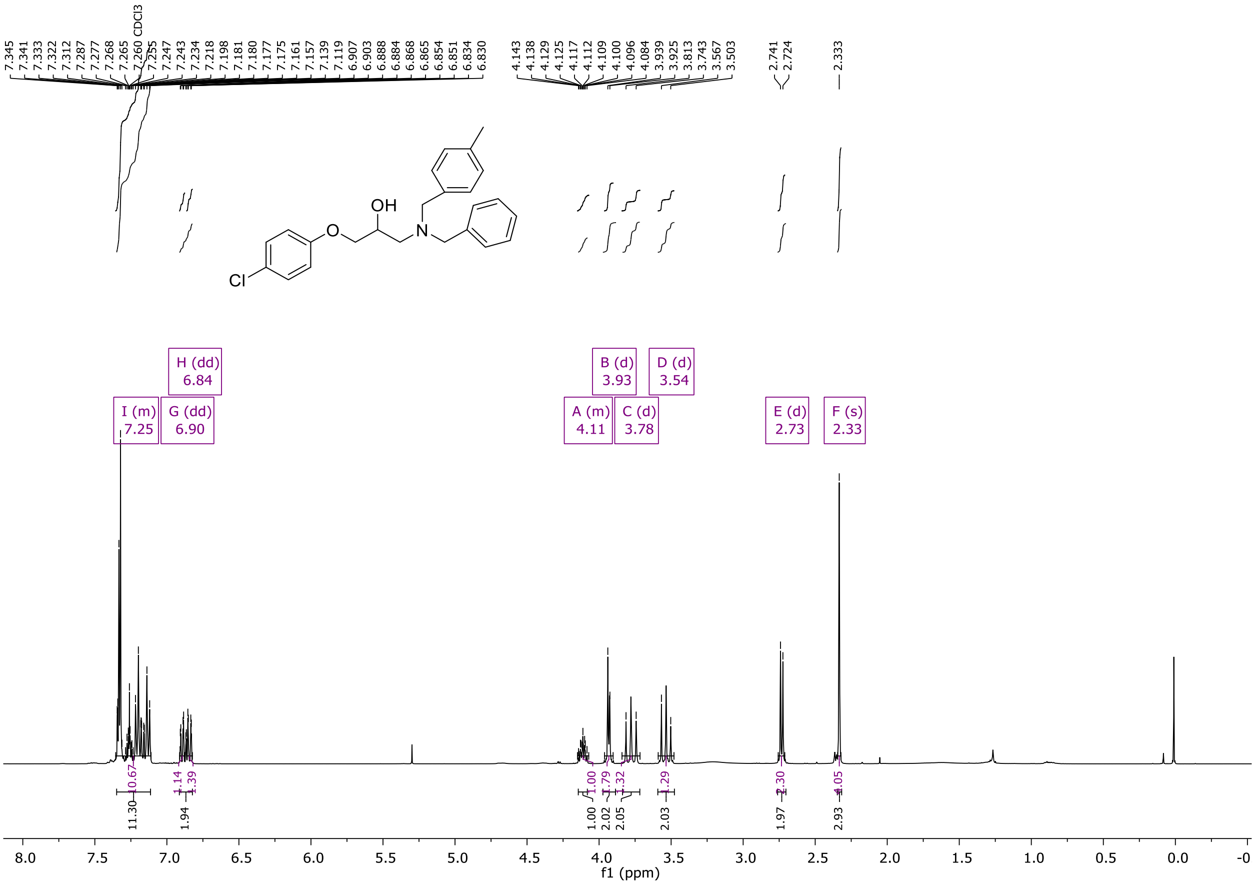

1-[Benzyl(4-methylbenzyl)amino]-3-(4-chlorophenoxy)propan-2-ol (27)

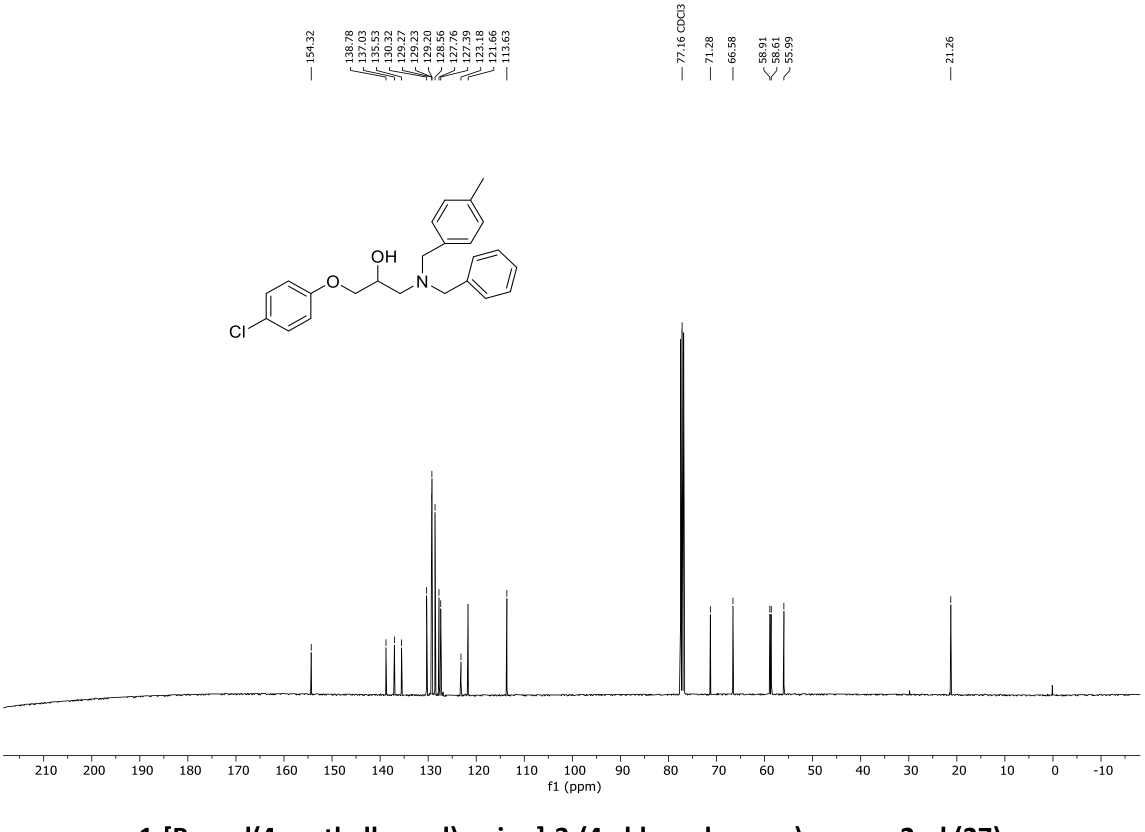

1-[Benzyl(4-methylbenzyl)amino]-3-(4-chlorophenoxy)propan-2-ol (27)

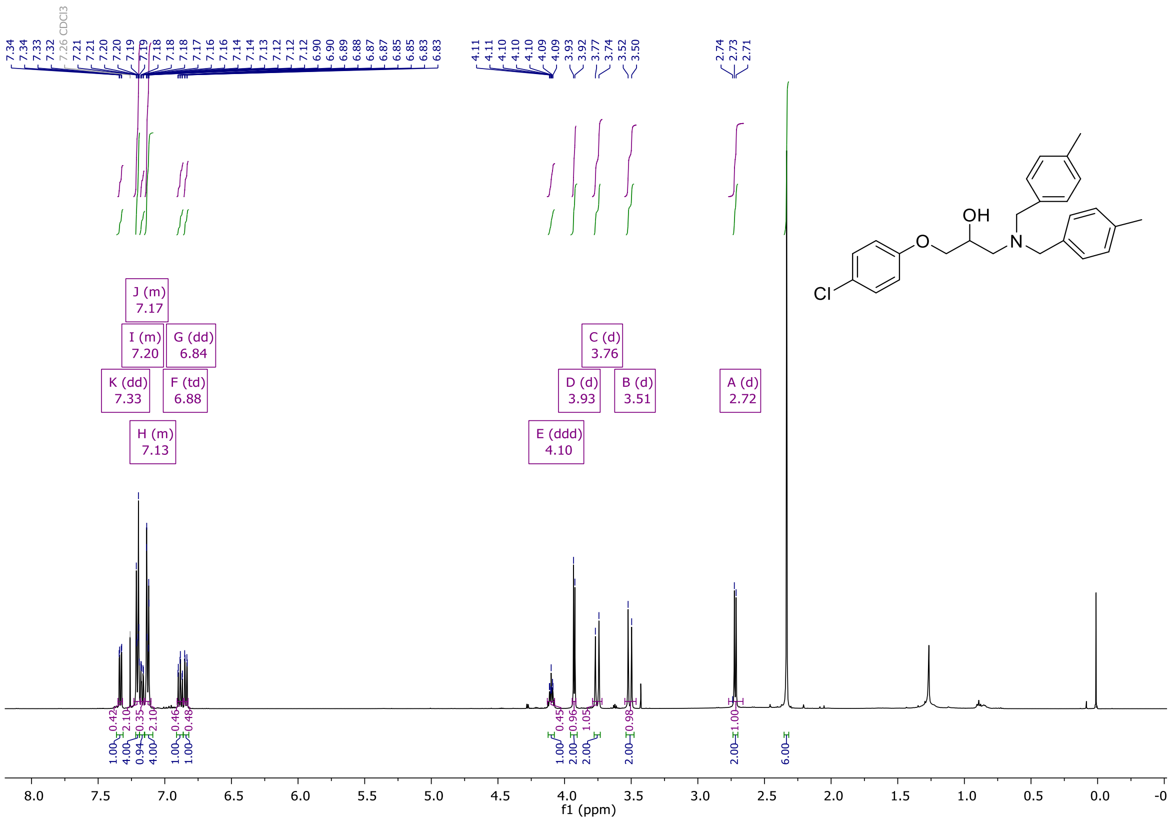

1-[Bis(4-methylbenzyl)amino]-3-(4-chlorophenoxy)propan-2-ol (28)

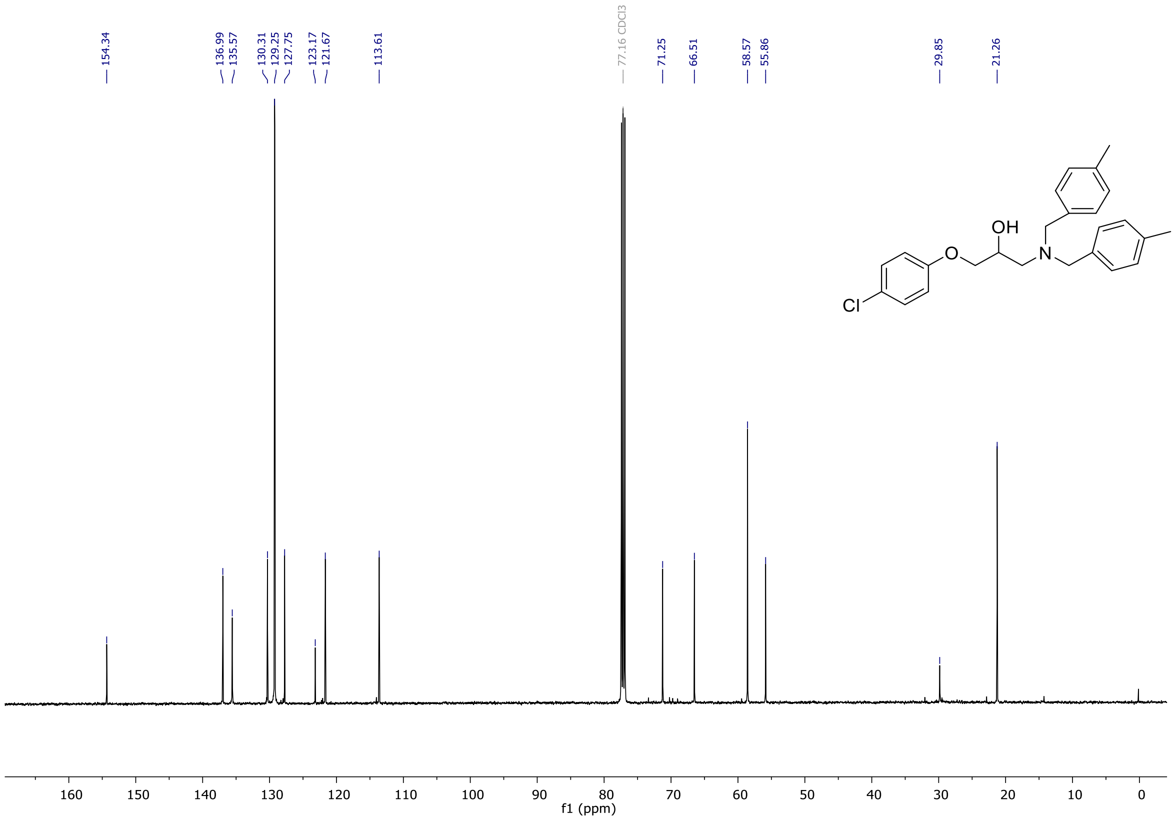

1-[Bis(4-methylbenzyl)amino]-3-(4-chlorophenoxy)propan-2-ol (28)

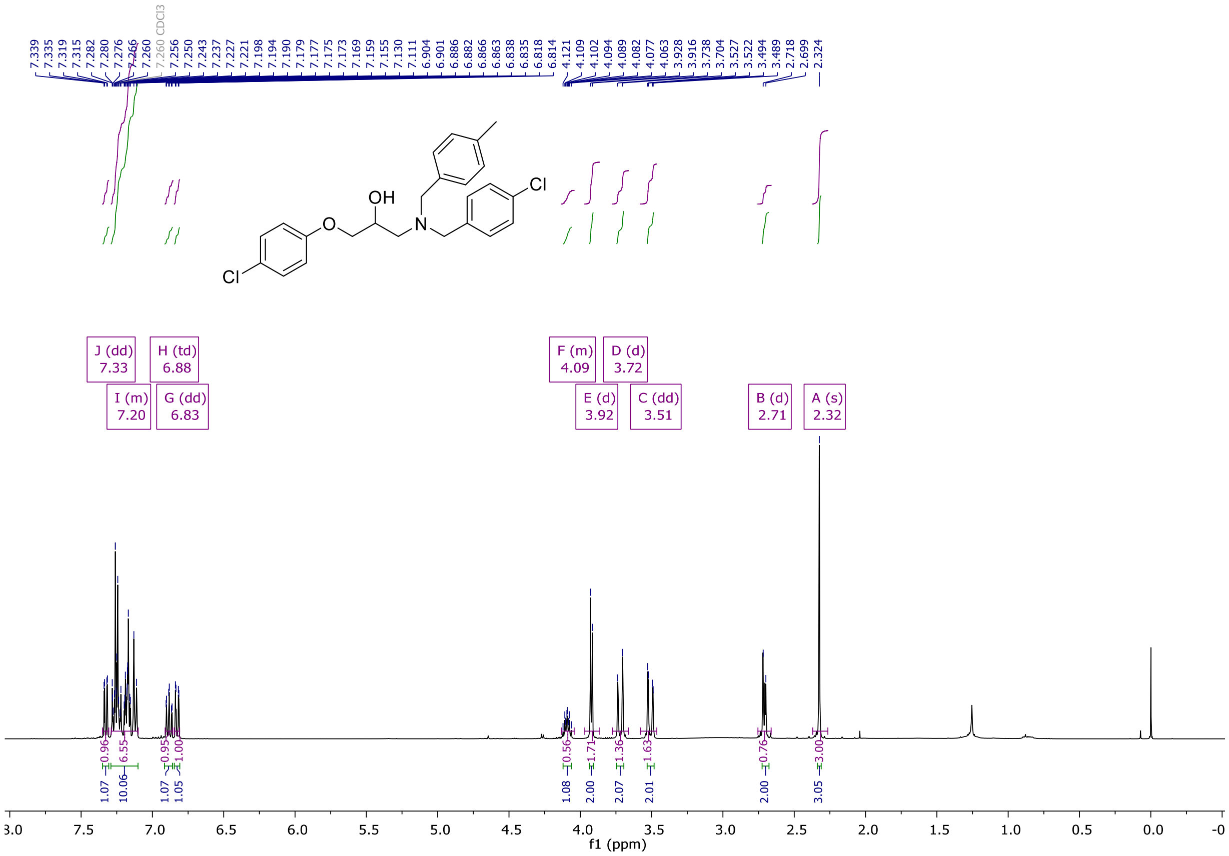

1-[(4-Chlorobenzyl)(4-methylbenzyl)amino]-3-(4-chlorophenoxy)propan-2-ol (29)

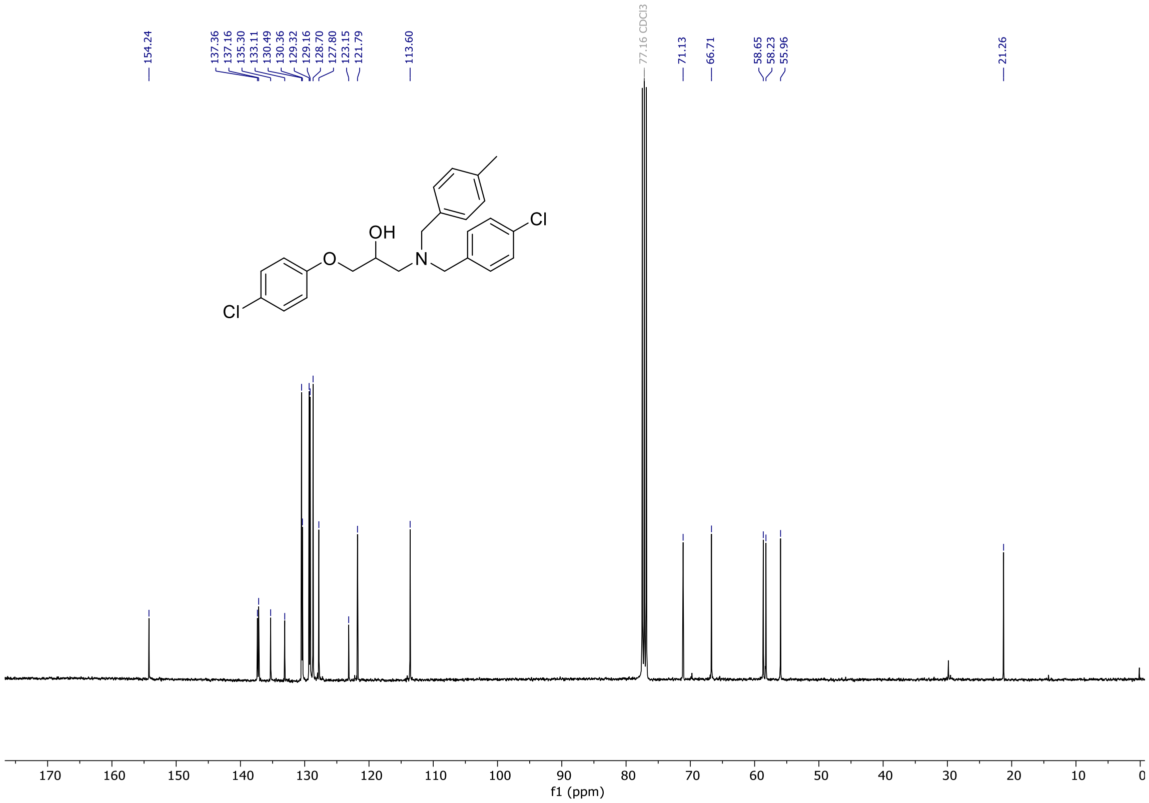

1-[(4-Chlorobenzyl)(4-methylbenzyl)amino]-3-(4-chlorophenoxy)propan-2-ol (29)

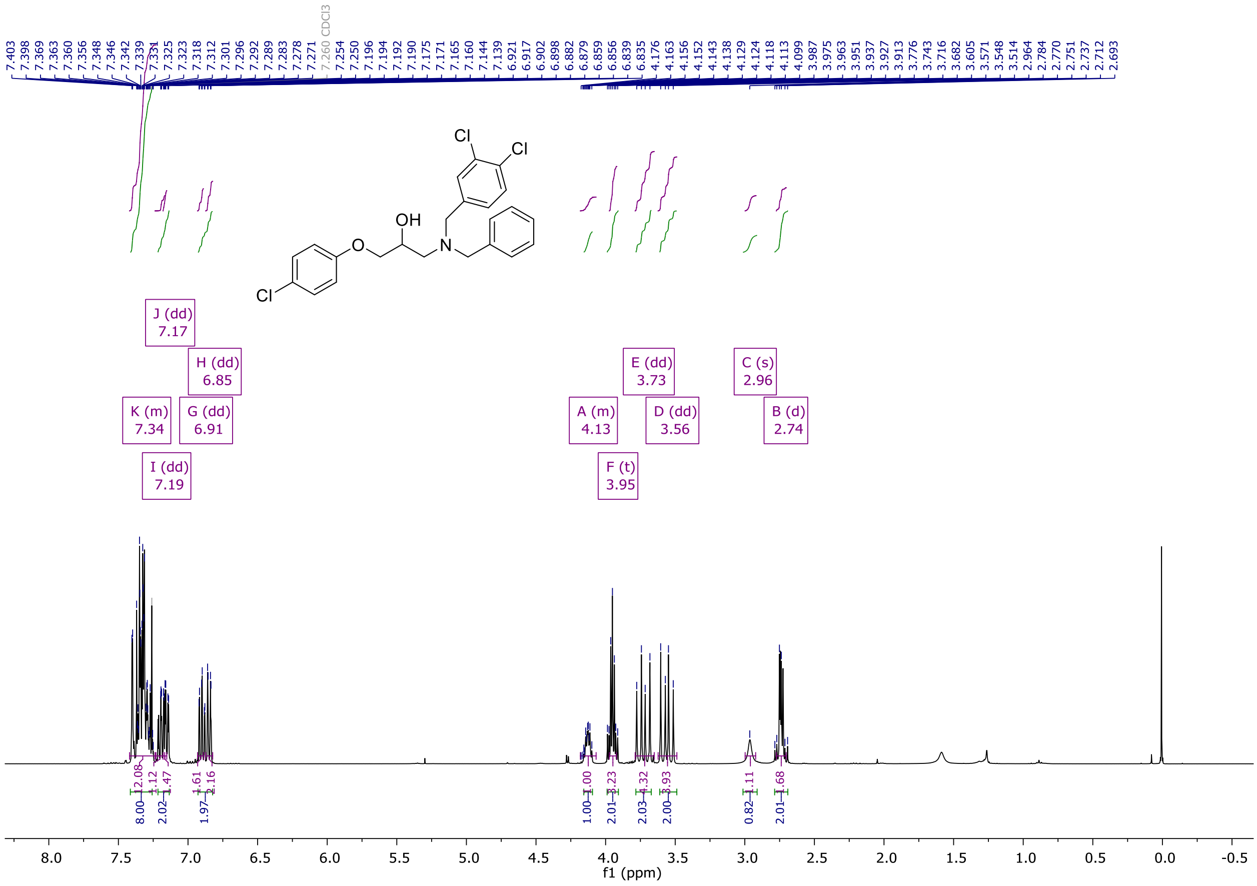

1-[Benzyl(3,4-dichlorobenzyl)amino]-3-(4-chlorophenoxy)propan-2-ol (30)

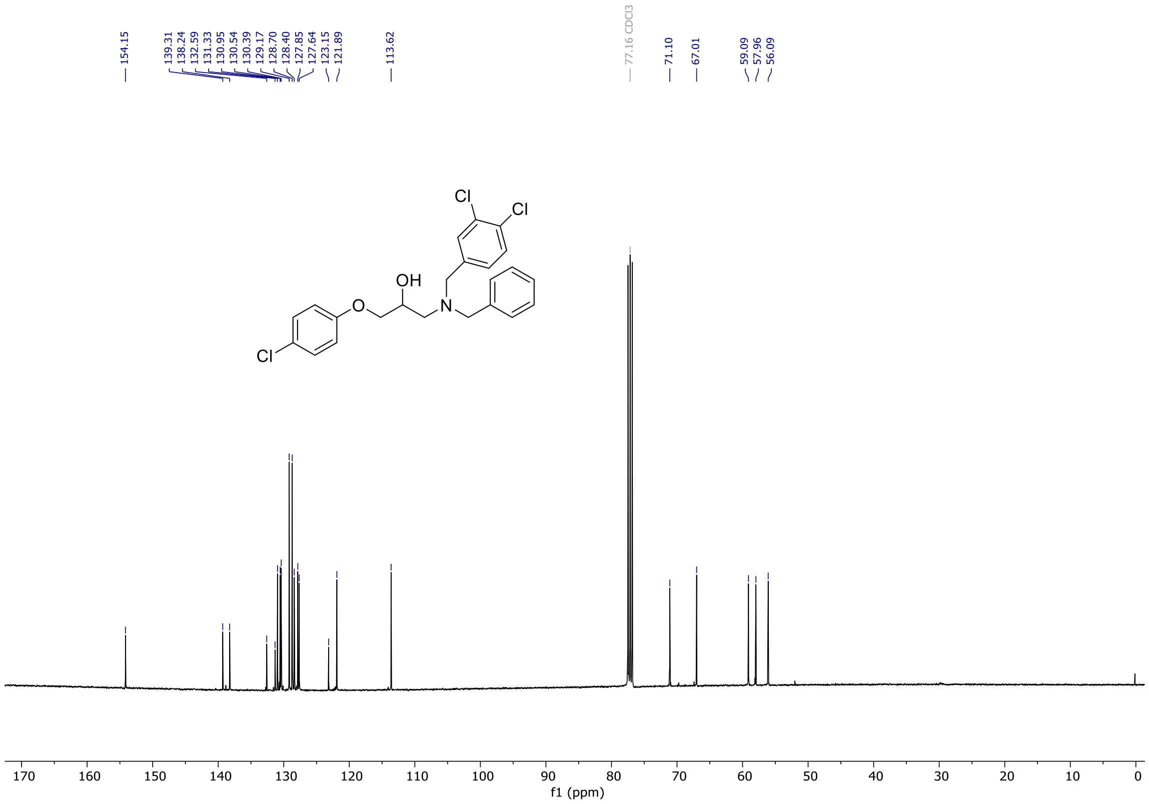

1-[Benzyl(3,4-dichlorobenzyl)amino]-3-(4-chlorophenoxy)propan-2-ol (30)

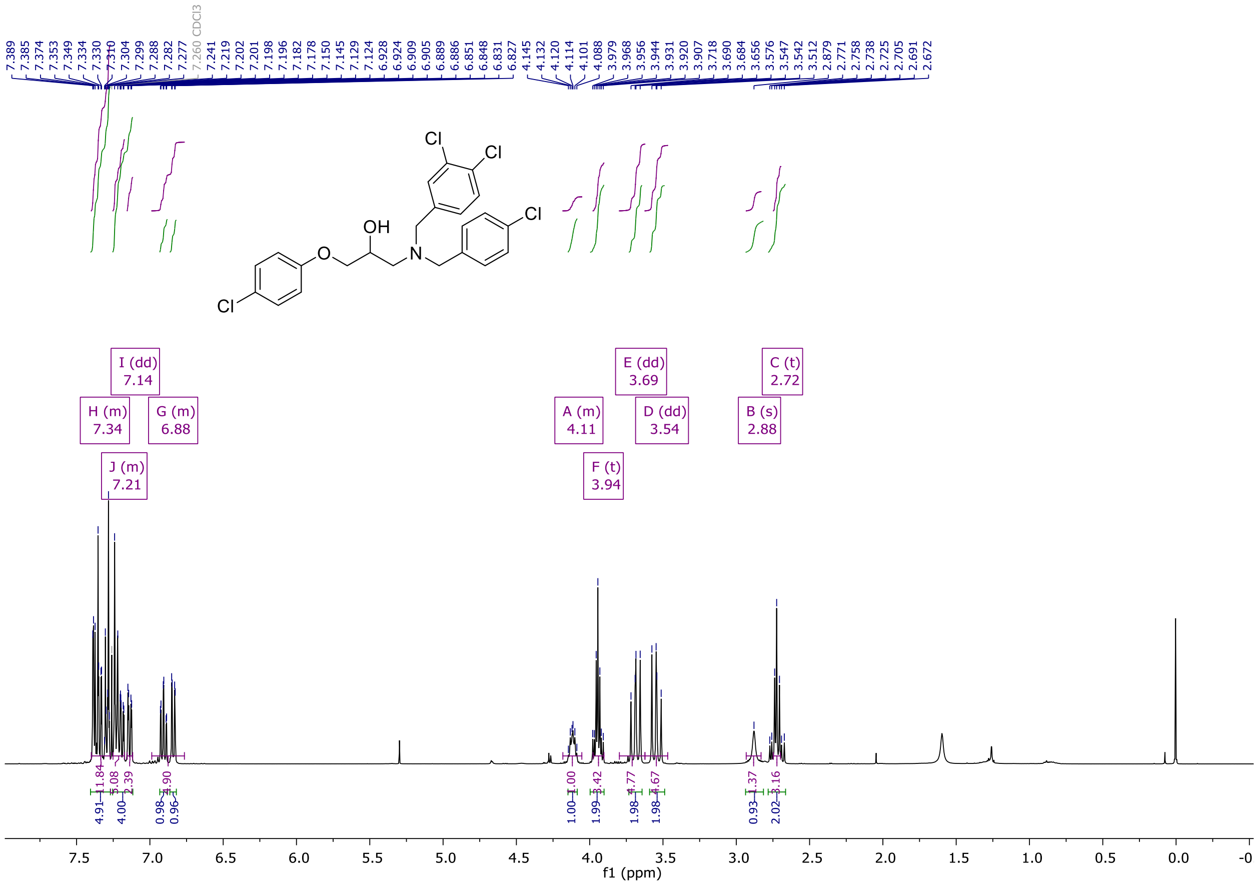

1-[(4-Chlorobenzyl)(3,4-dichlorobenzyl)amino]-3-(4-chlorophenoxy)propan-2-ol (31)

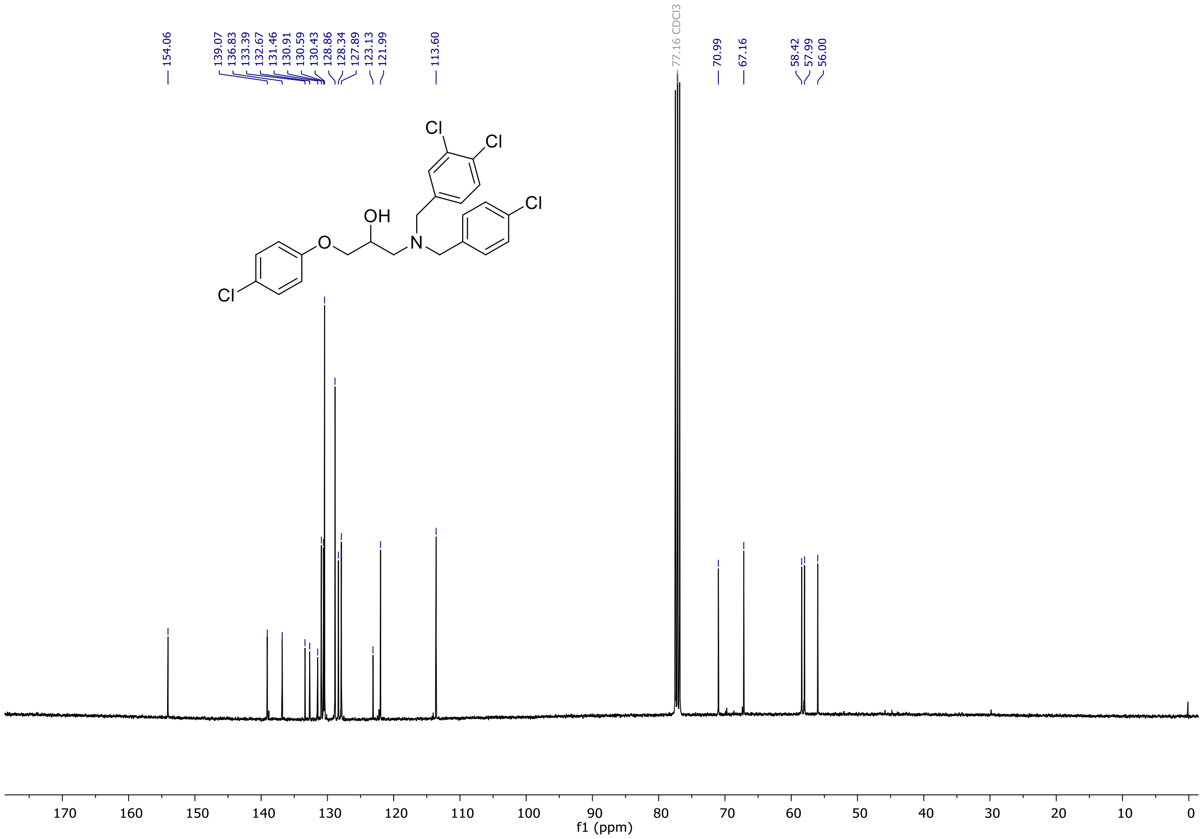

1-[(4-Chlorobenzyl)(3,4-dichlorobenzyl)amino]-3-(4-chlorophenoxy)propan-2-ol (31)

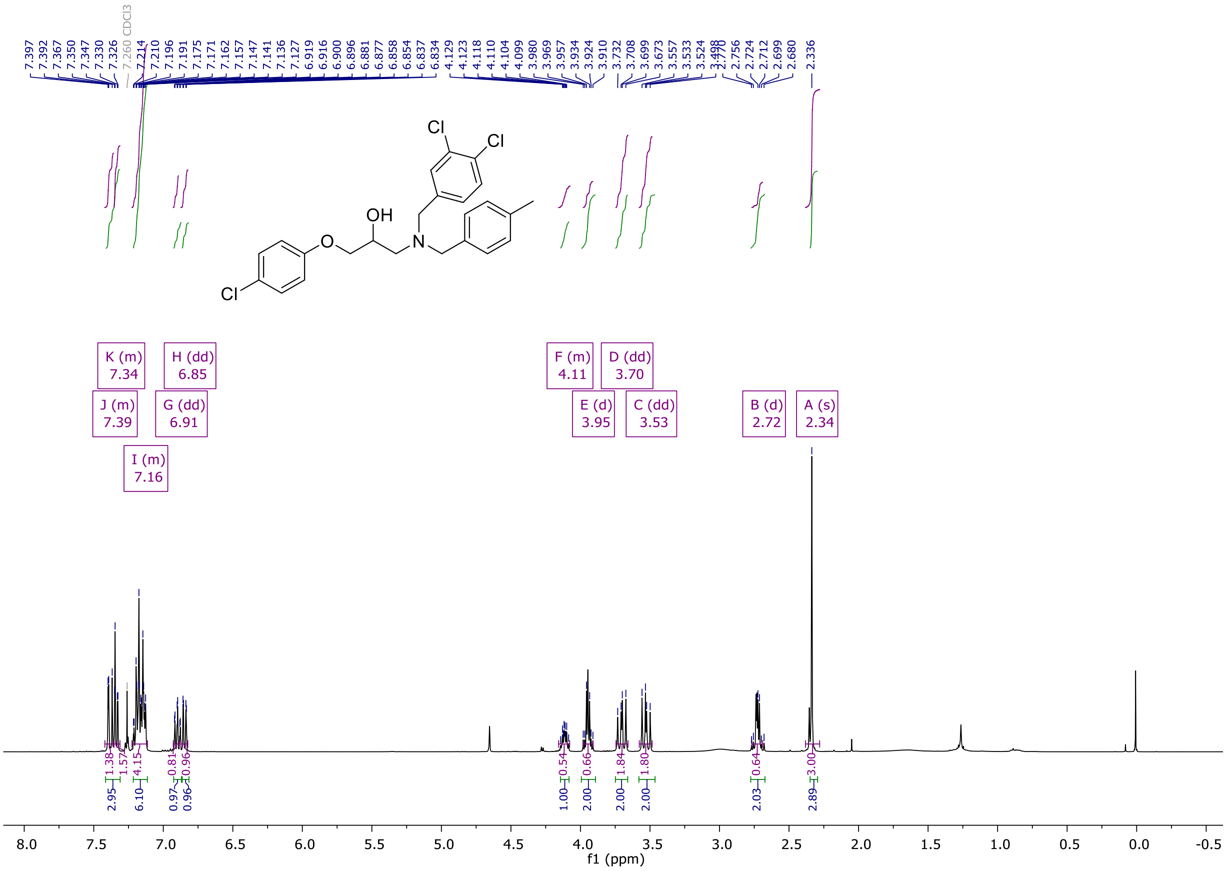

1-(4-Chlorophenoxy)-3-[(3,4-dichlorobenzyl)(4-methylbenzyl)amino]propan-2-ol (32)

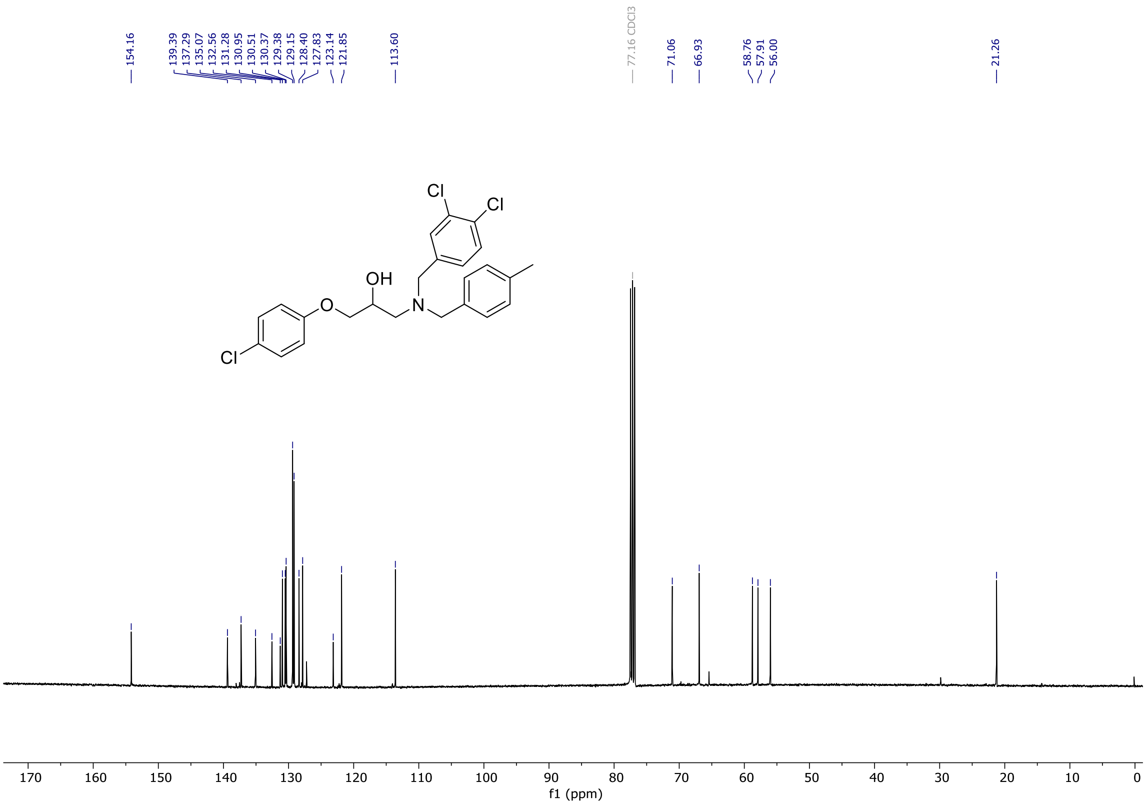

1-(4-Chlorophenoxy)-3-[(3,4-dichlorobenzyl)(4-methylbenzyl)amino]propan-2-ol (32)

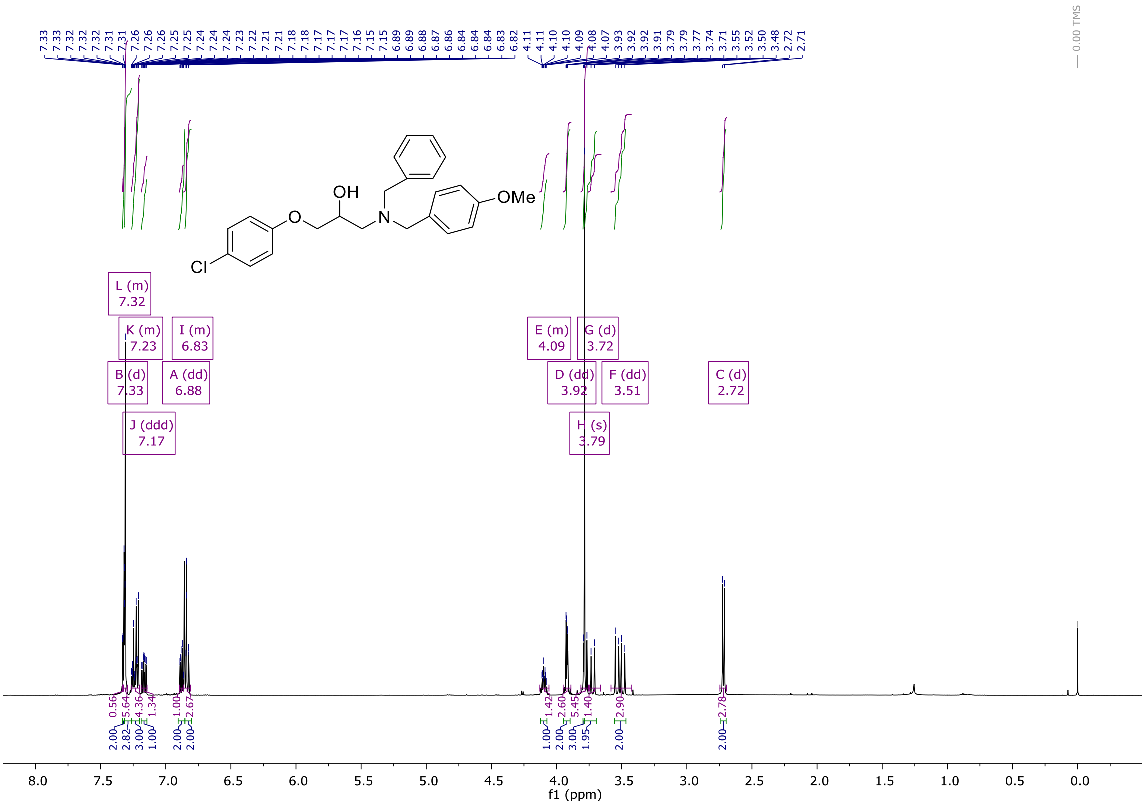

1-[Benzyl(4-methoxybenzyl)amino]-3-(4-chlorophenoxy)propan-2-ol (33)

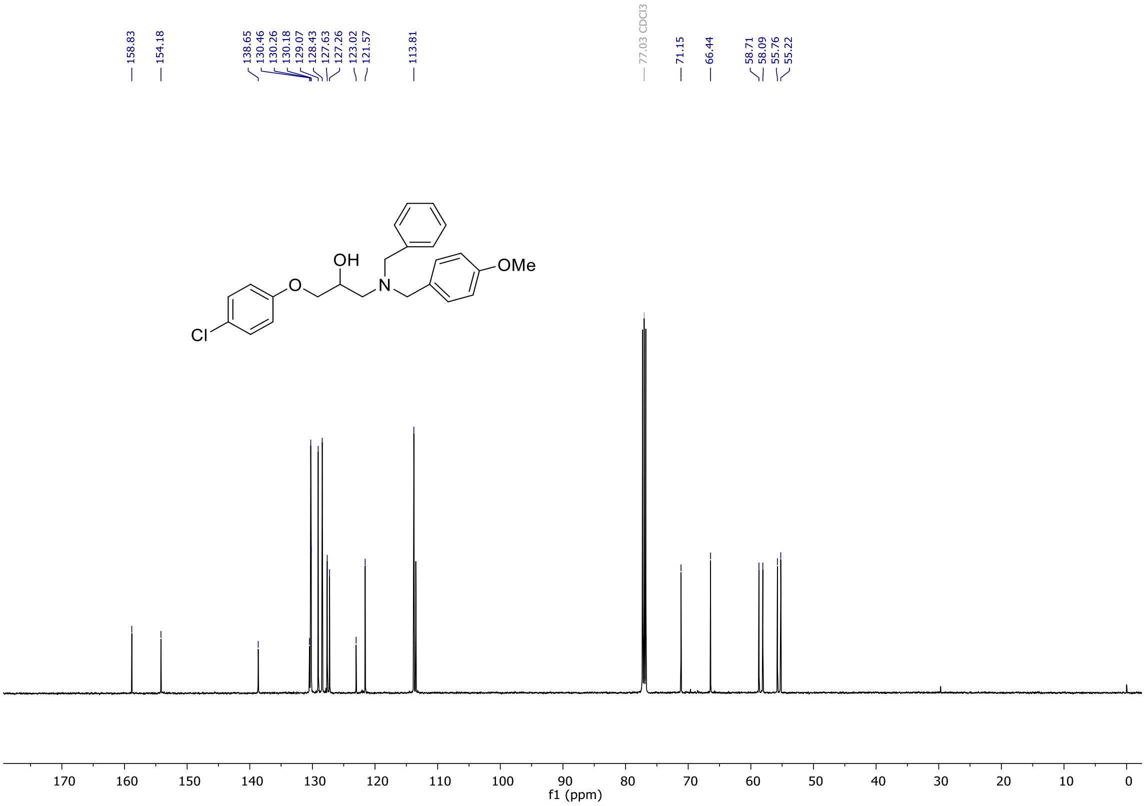

1-[Benzyl(4-methoxybenzyl)amino]-3-(4-chlorophenoxy)propan-2-ol (33)

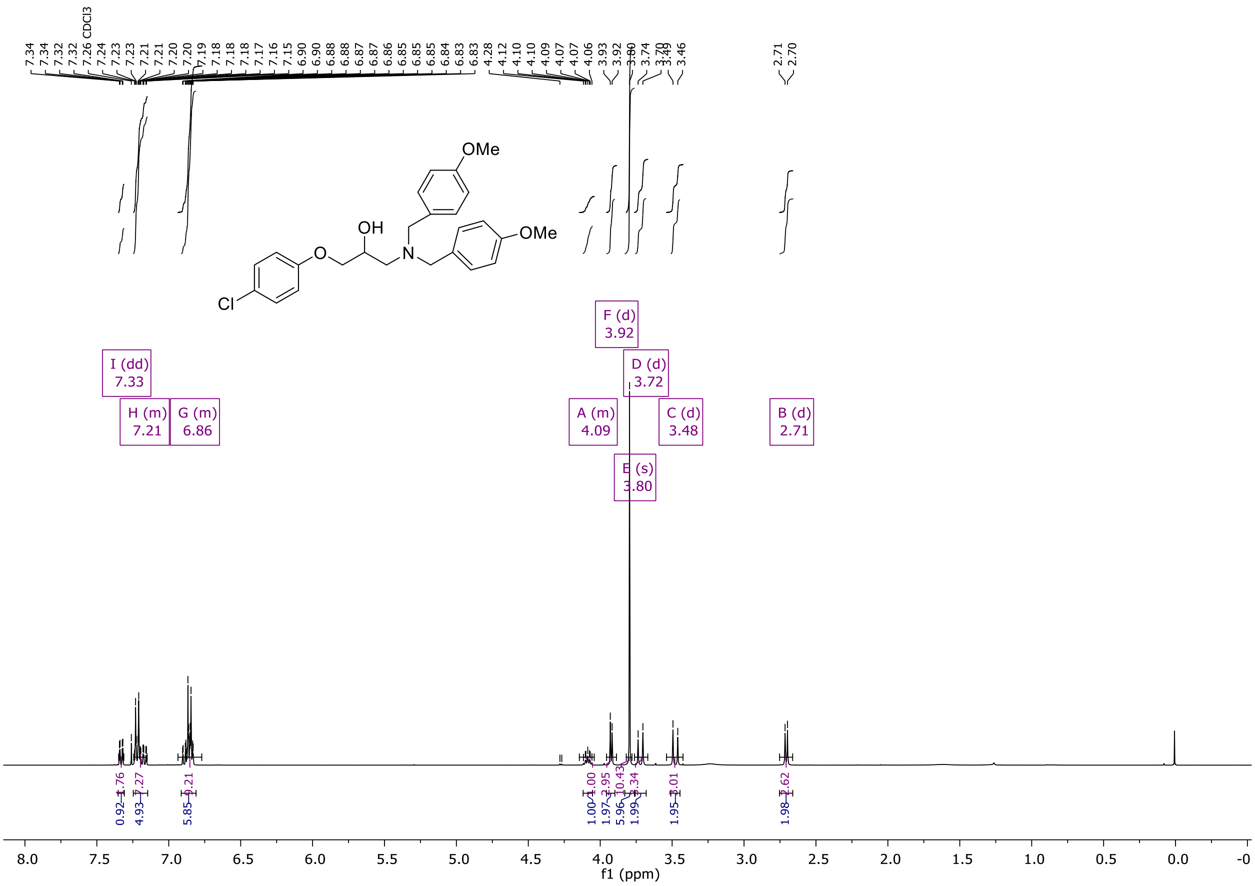

1-[Bis(4-methoxybenzyl)amino]-3-(4-chlorophenoxy)propan-2-ol (34)

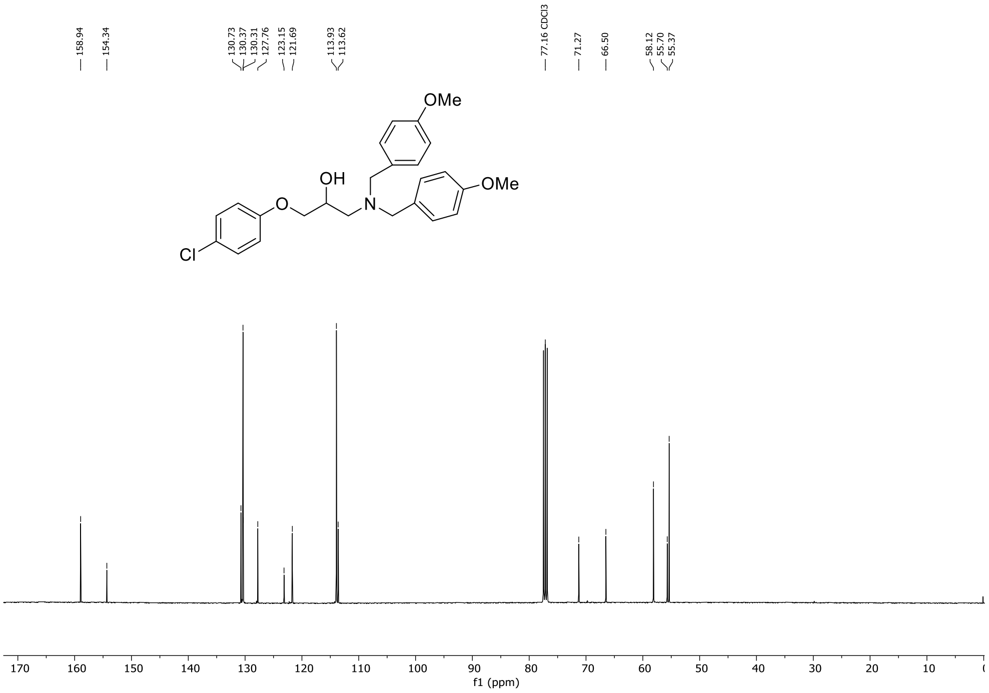

1-[Bis(4-methoxybenzyl)amino]-3-(4-chlorophenoxy)propan-2-ol (34)

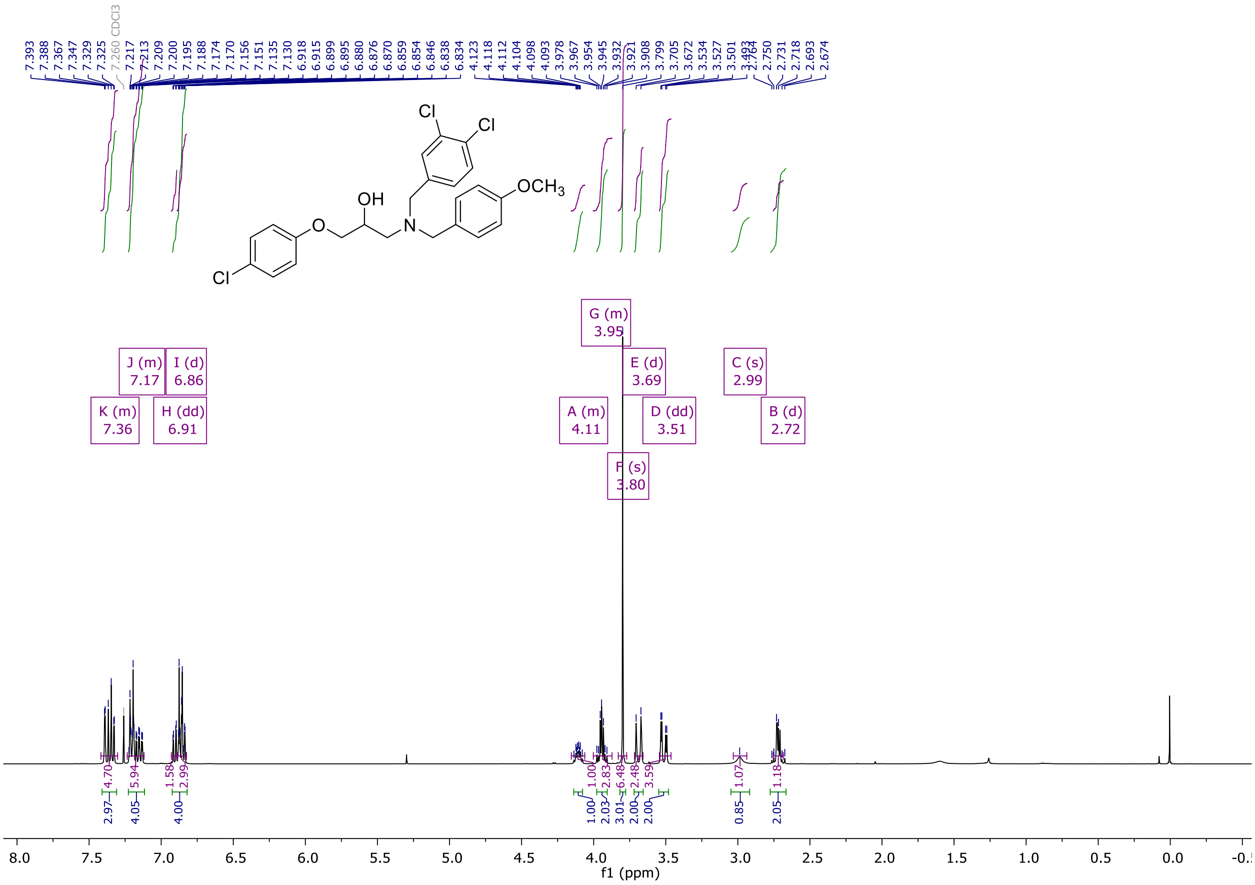

1-(4-Chlorophenoxy)-3-[(3,4-dichlorobenzyl)(4-methoxybenzyl)amino]propan-2-ol (35)

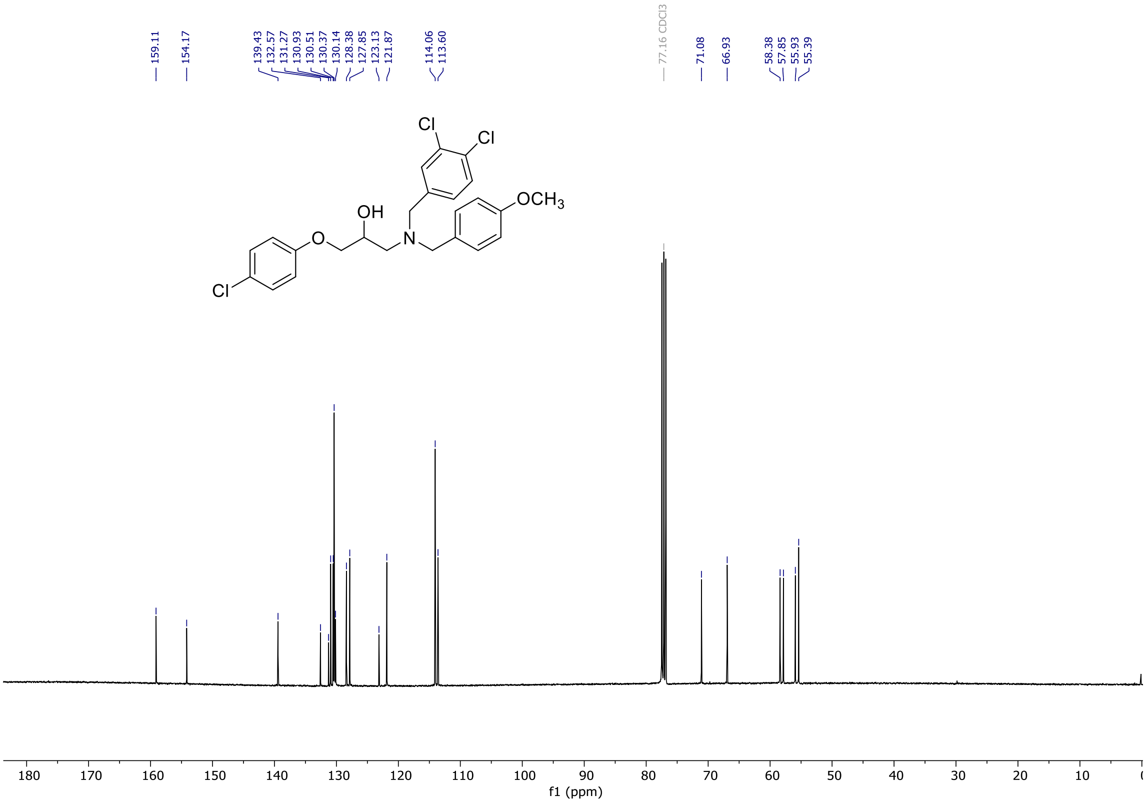

1-(4-Chlorophenoxy)-3-[(3,4-dichlorobenzyl)(4-methoxybenzyl)amino]propan-2-ol (35)

HPLC/MS analysis of BC1618, 1-35

1-(4-Chlorophenoxy)-3-(dibenzylamino)propan-2-ol

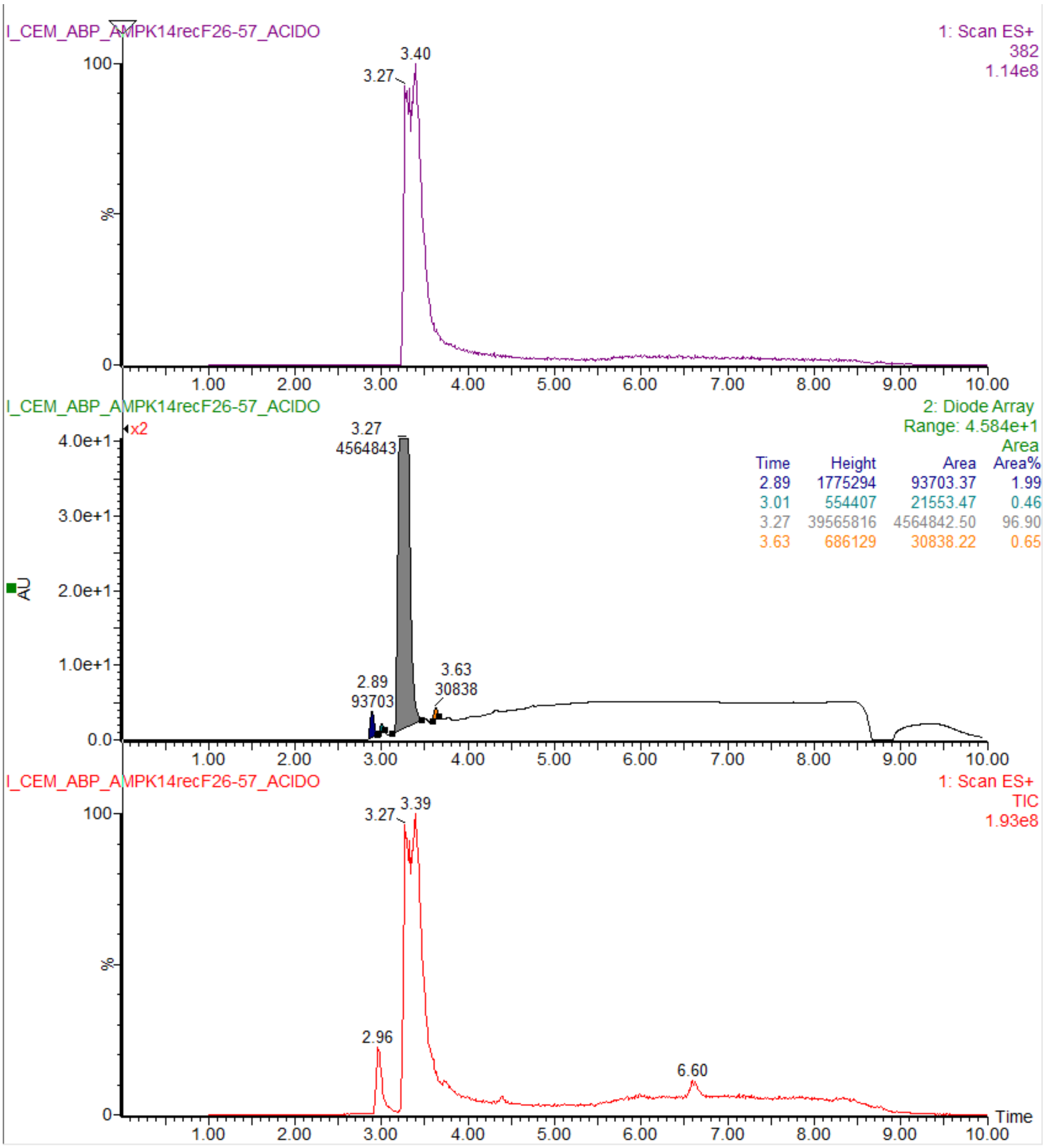

1-(Dibenzylamino)-3-(*p*-tolyloxy)propan-2-ol

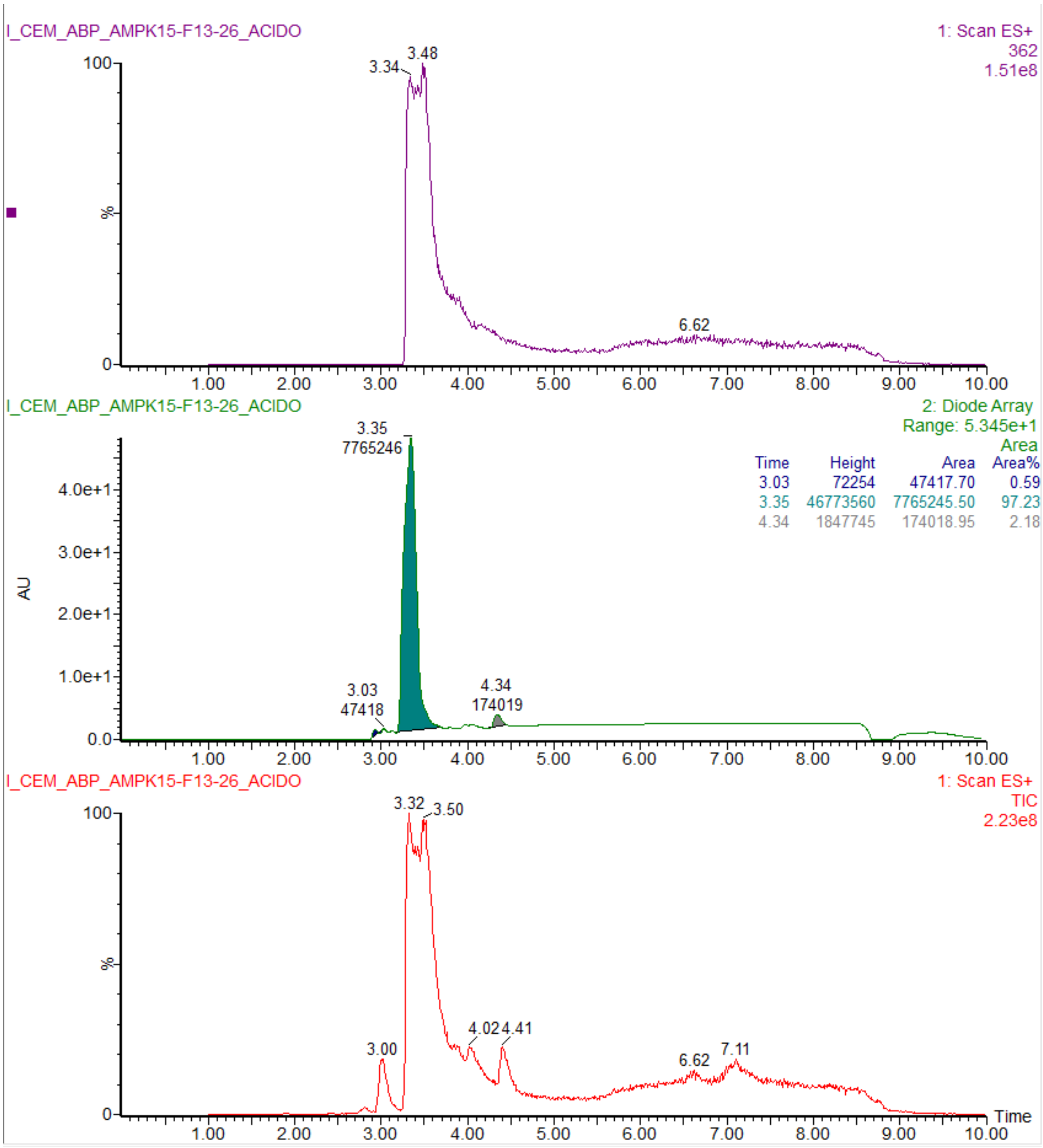

1-(4-Cyclohexylphenoxy)-3-(dibenzylamino)propan-2-ol

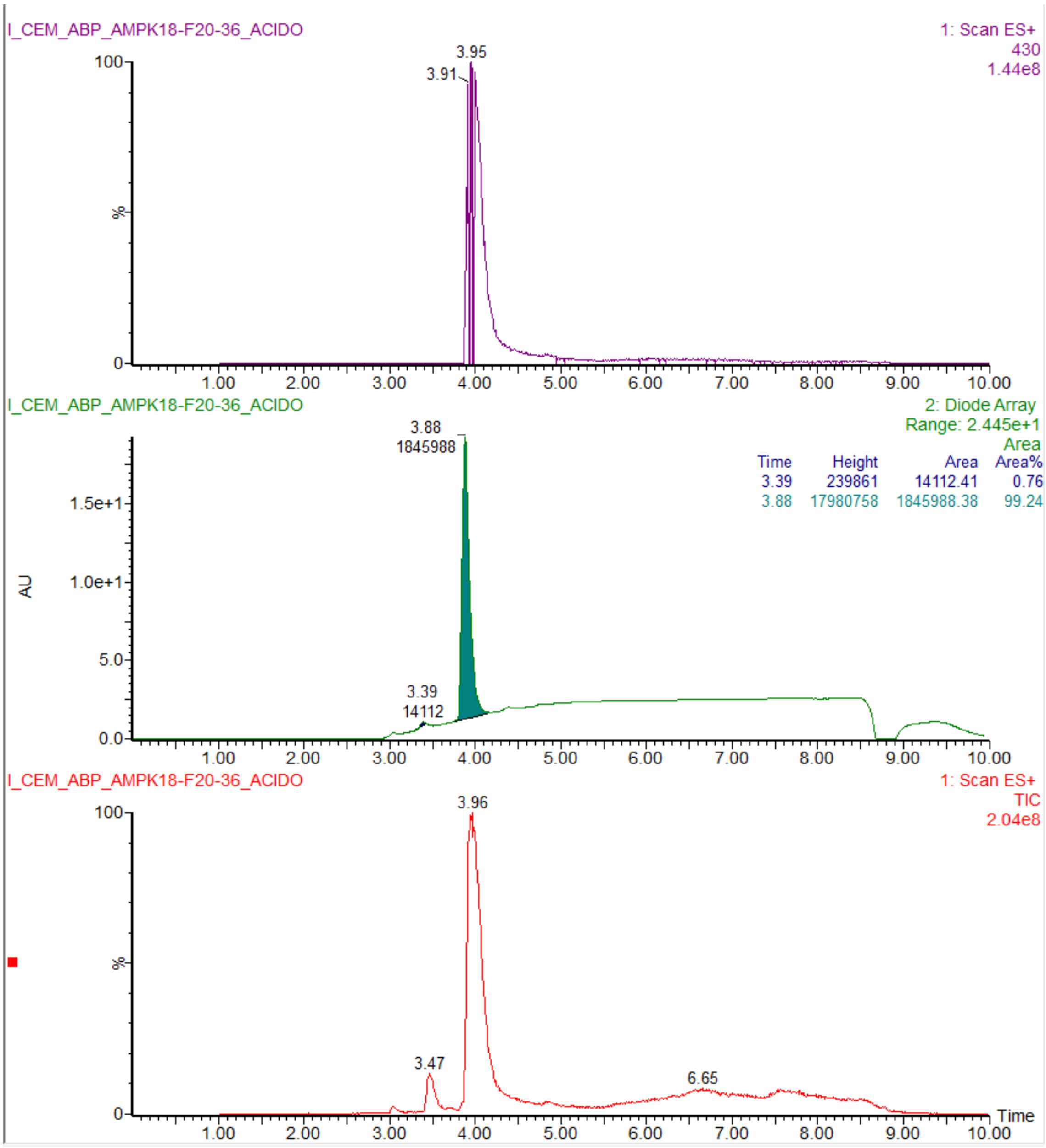

1-[4-(*Tert*-butyl)phenoxy]-3-(dibenzylamino)propan-2-ol

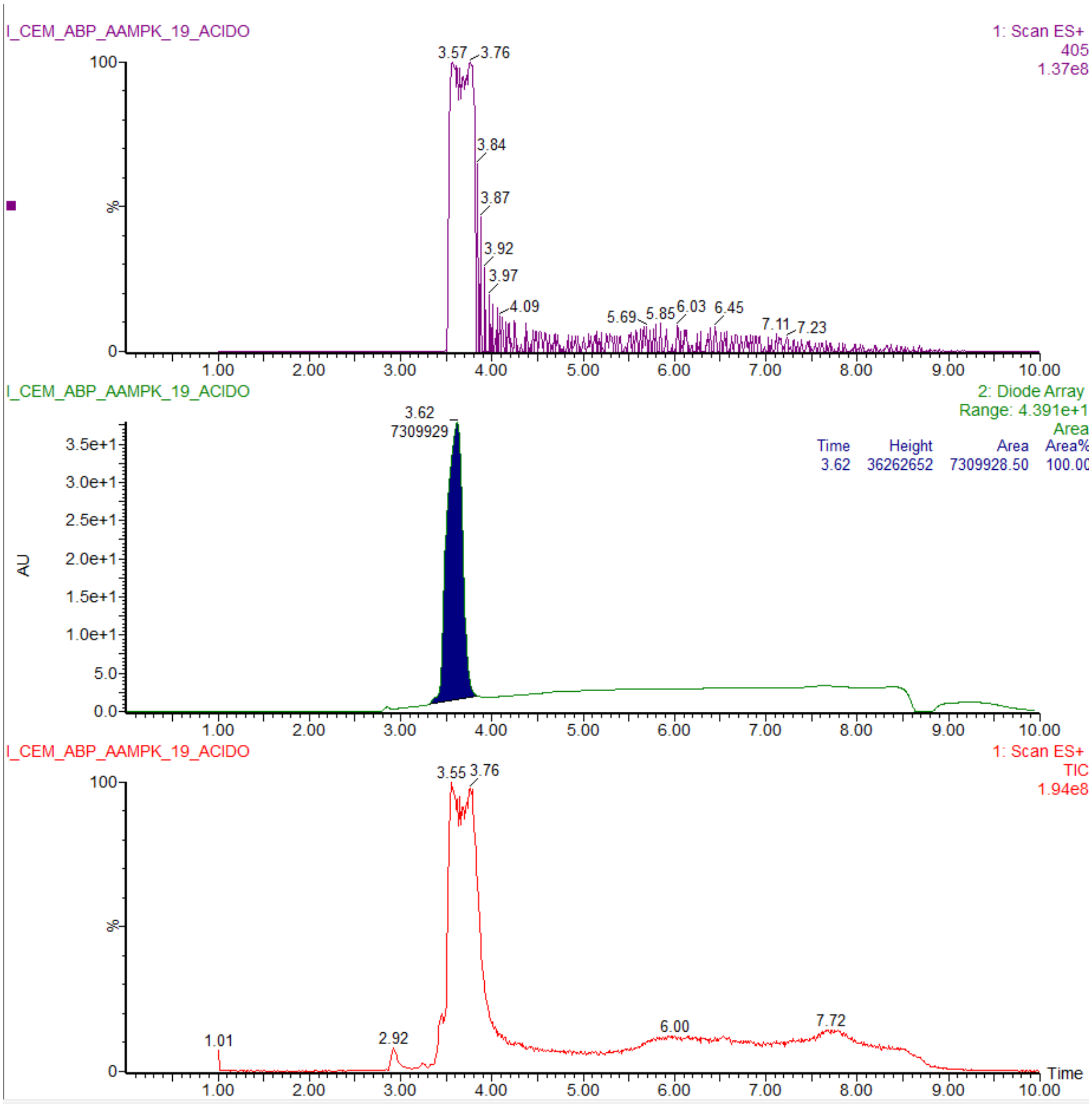

1-[4-(Benzyloxy)phenoxy]-3-(dibenzylamino)propan-2-ol

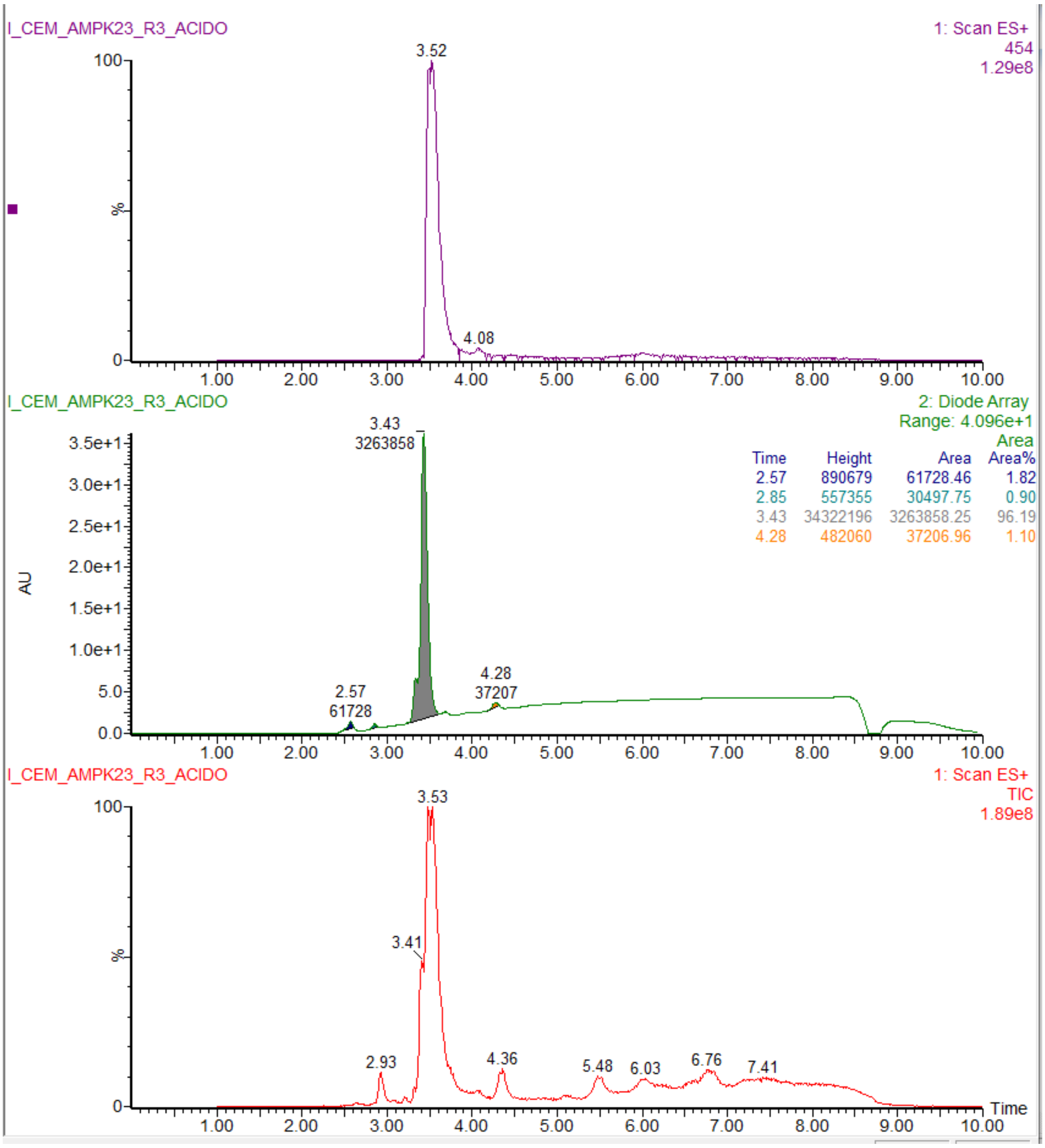

1-(Dibenzylamino)-3-(2,4-dichlorophenoxy)propan-2-ol

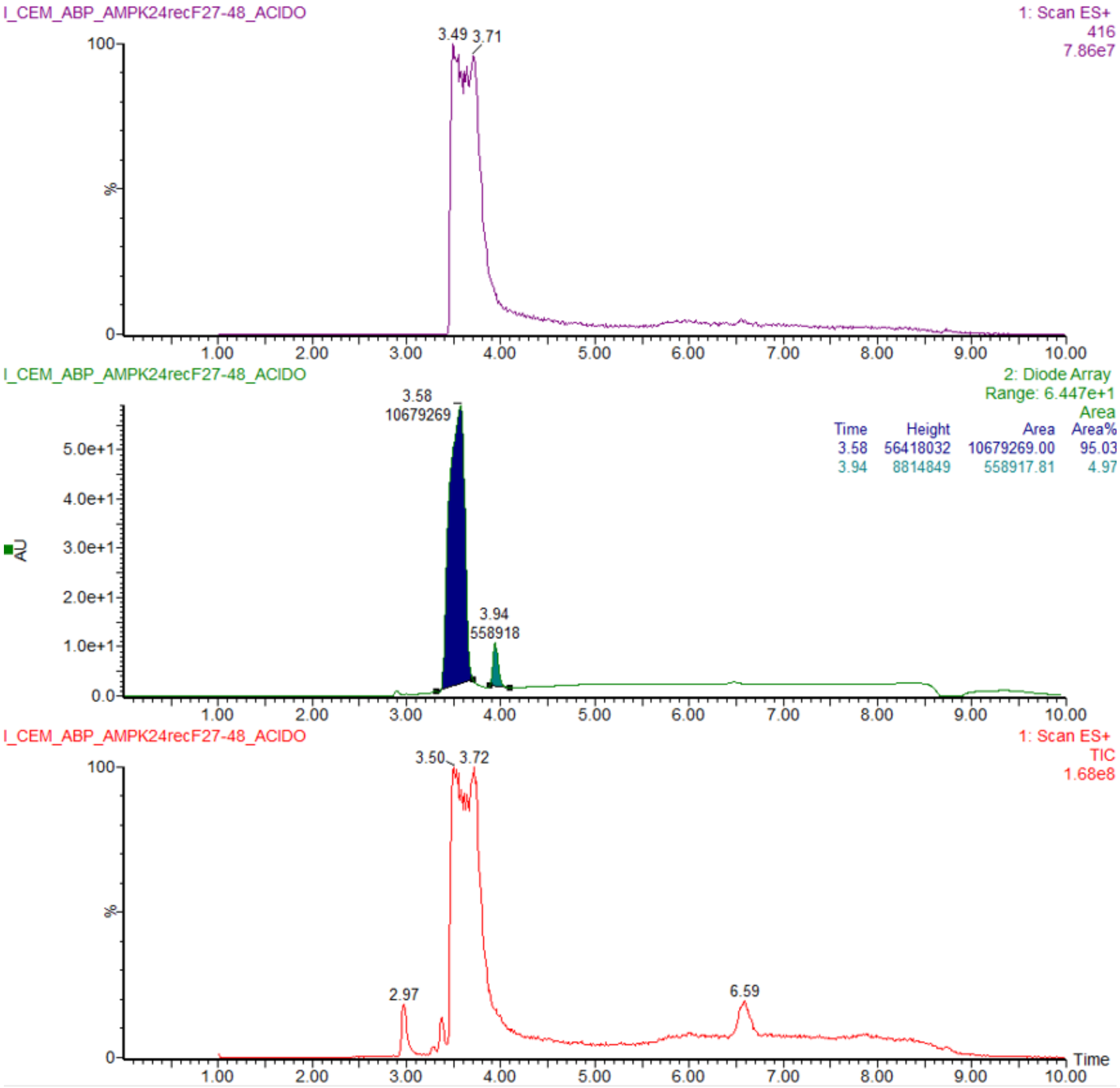

1-(4-Cyclopentylphenoxy)-3-(dibenzylamino)propan-2-ol

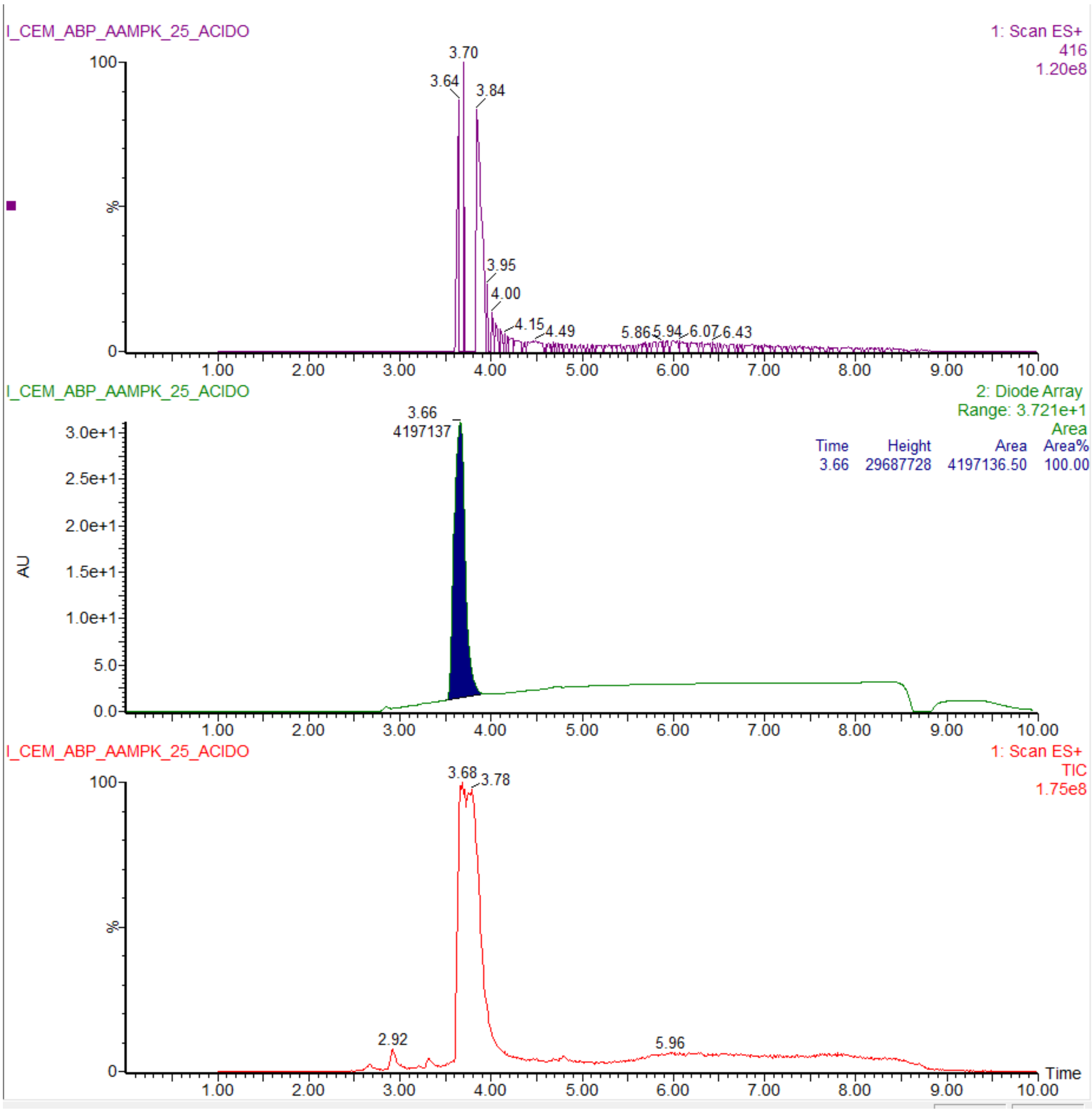

1-(Dibenzylamino)-3-(4-isopropylphenoxy)propan-2-ol

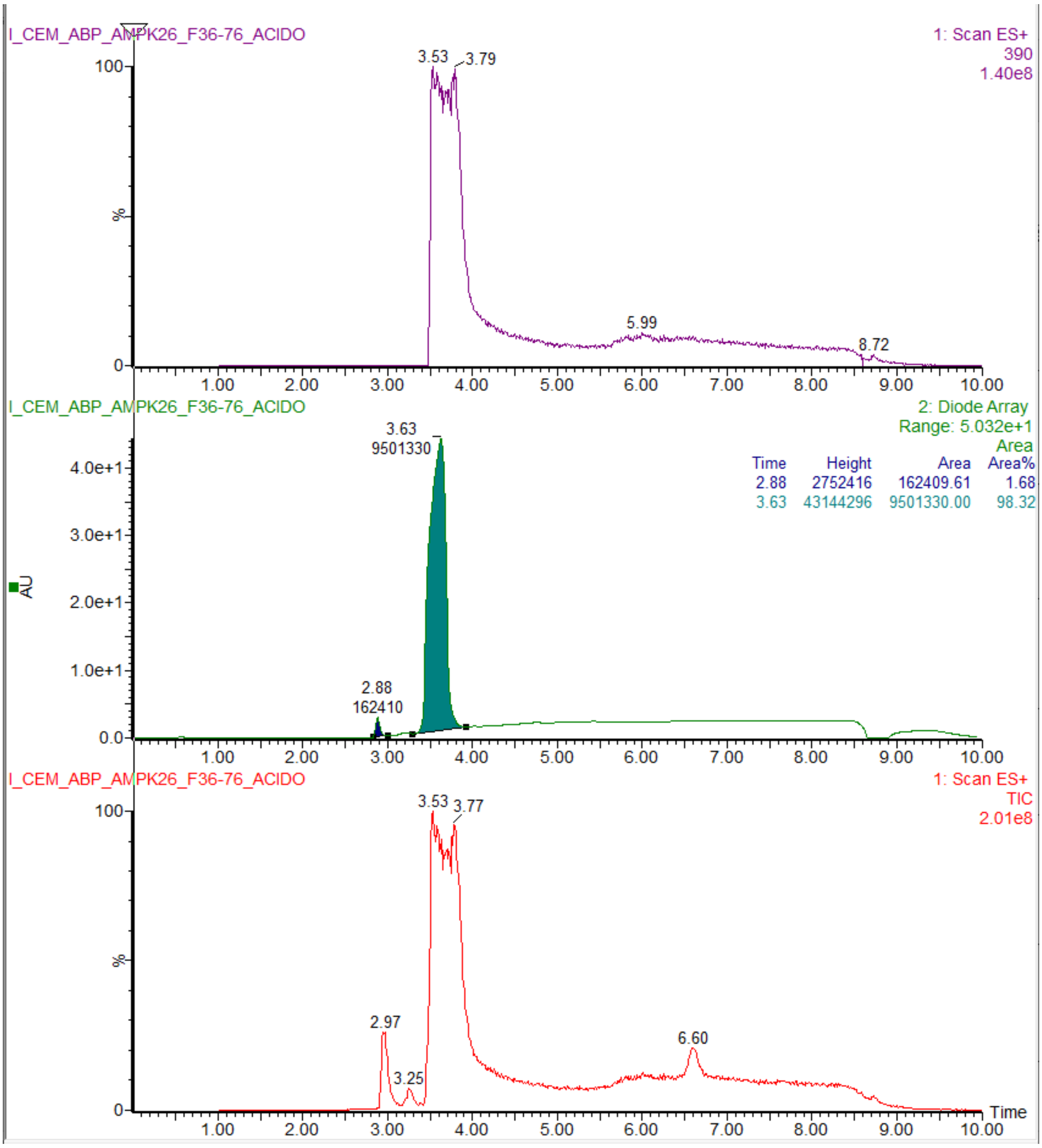

1-(Dibenzylamino)-3-phenoxypropan-2-ol

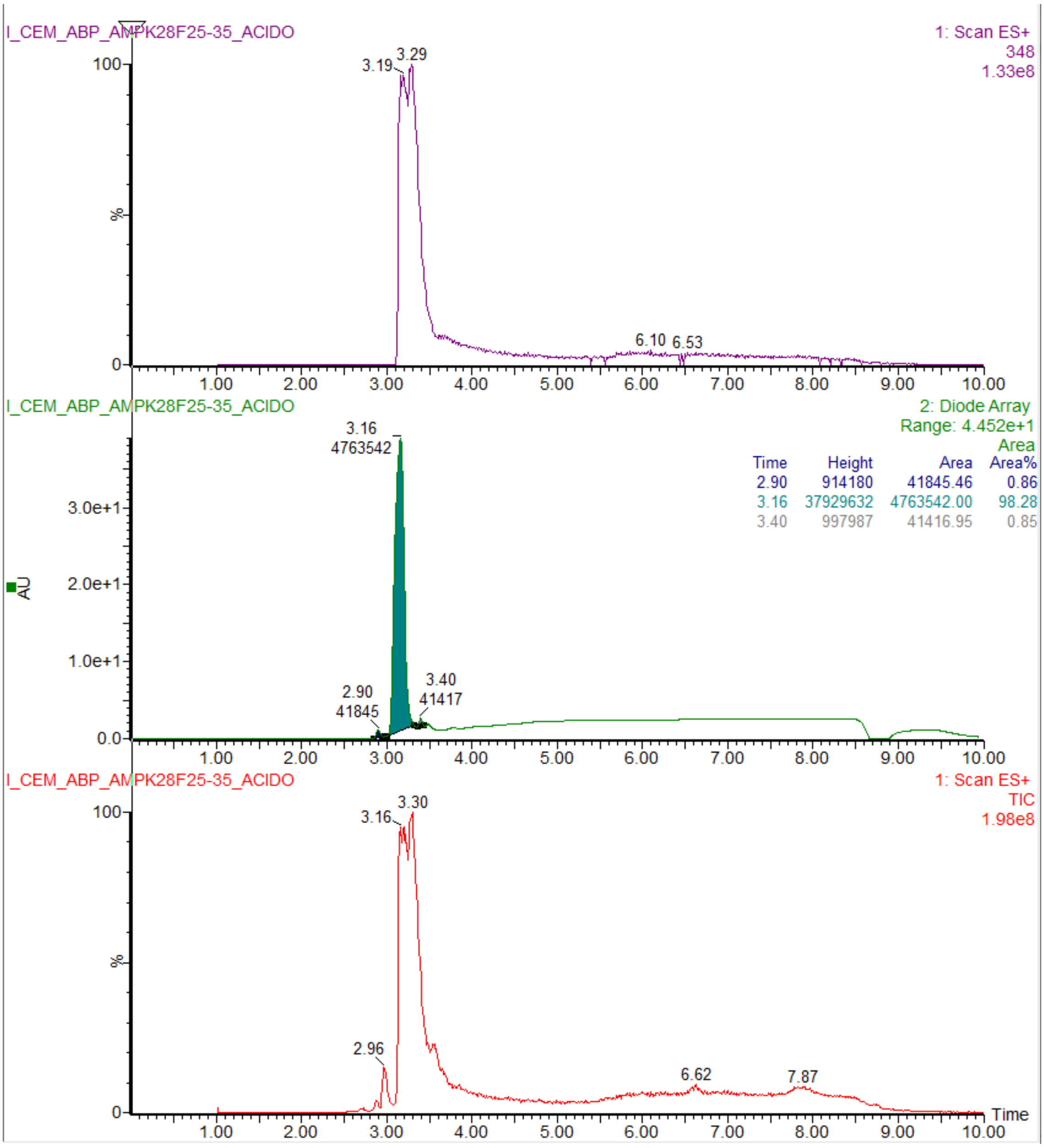

1-(Dibenzylamino)-3-(3,4-dimethylphenoxy)propan-2-ol

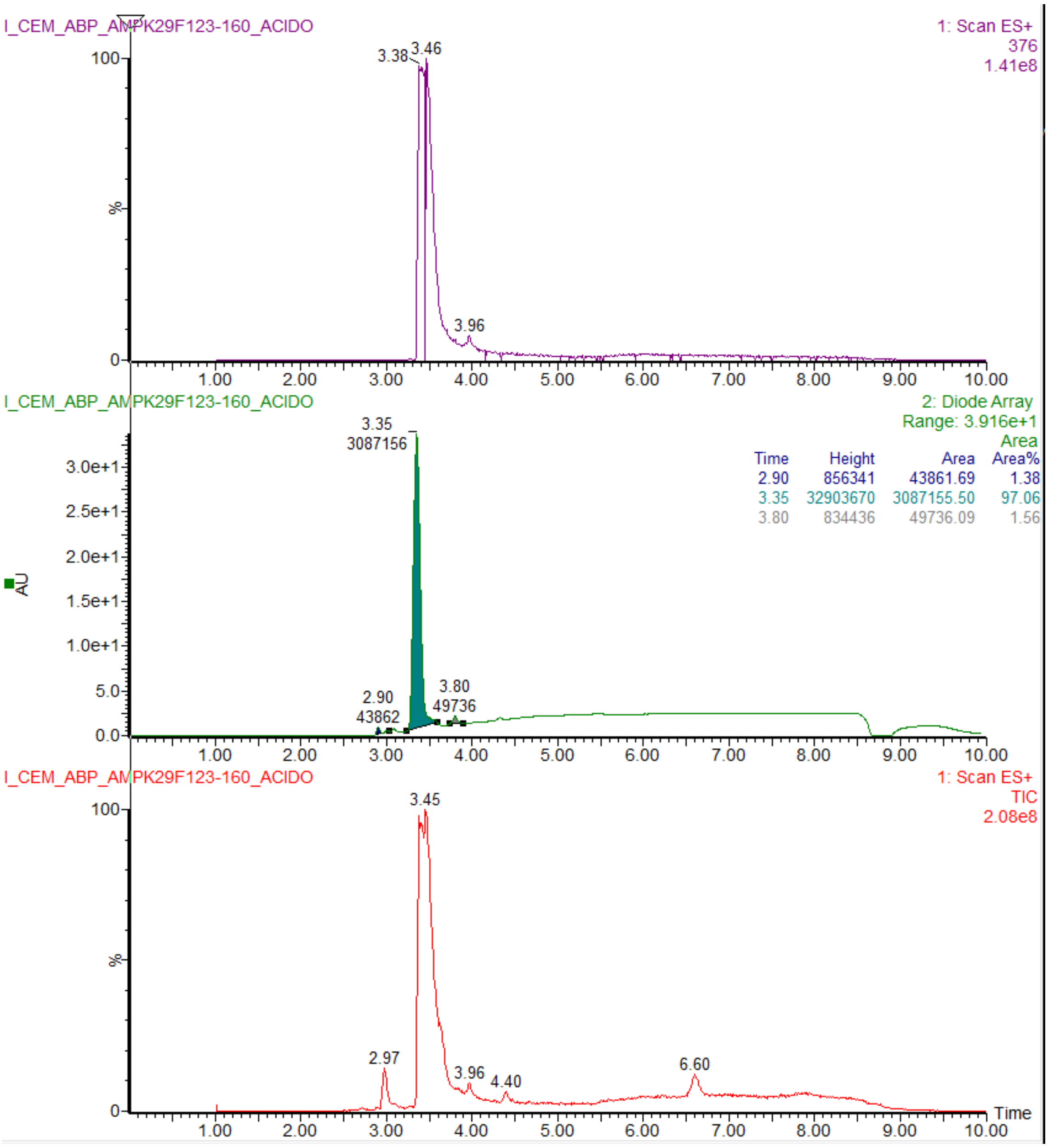

Time	Height	Area	Area Area%
2.90	856341	43861.69	1.38
3.35	32903670	3087155.50	97.06
3.80	834436	49736.09	1.56

1-(Dibenzylamino)-3-(4-methoxyphenoxy)propan-2-ol

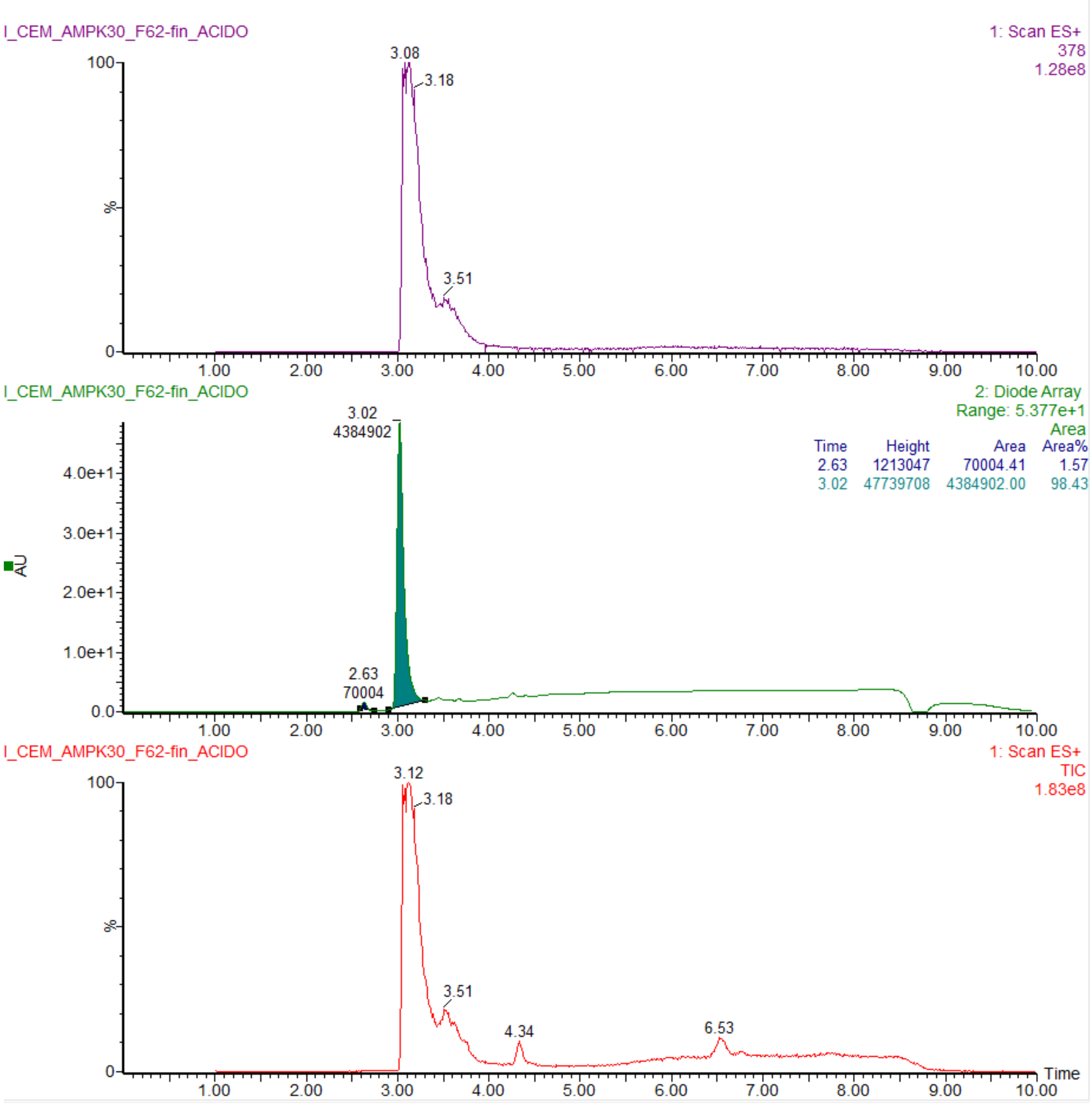

1-(Dibenzylamino)-3-(4-propoxyphenoxy)propan-2-ol

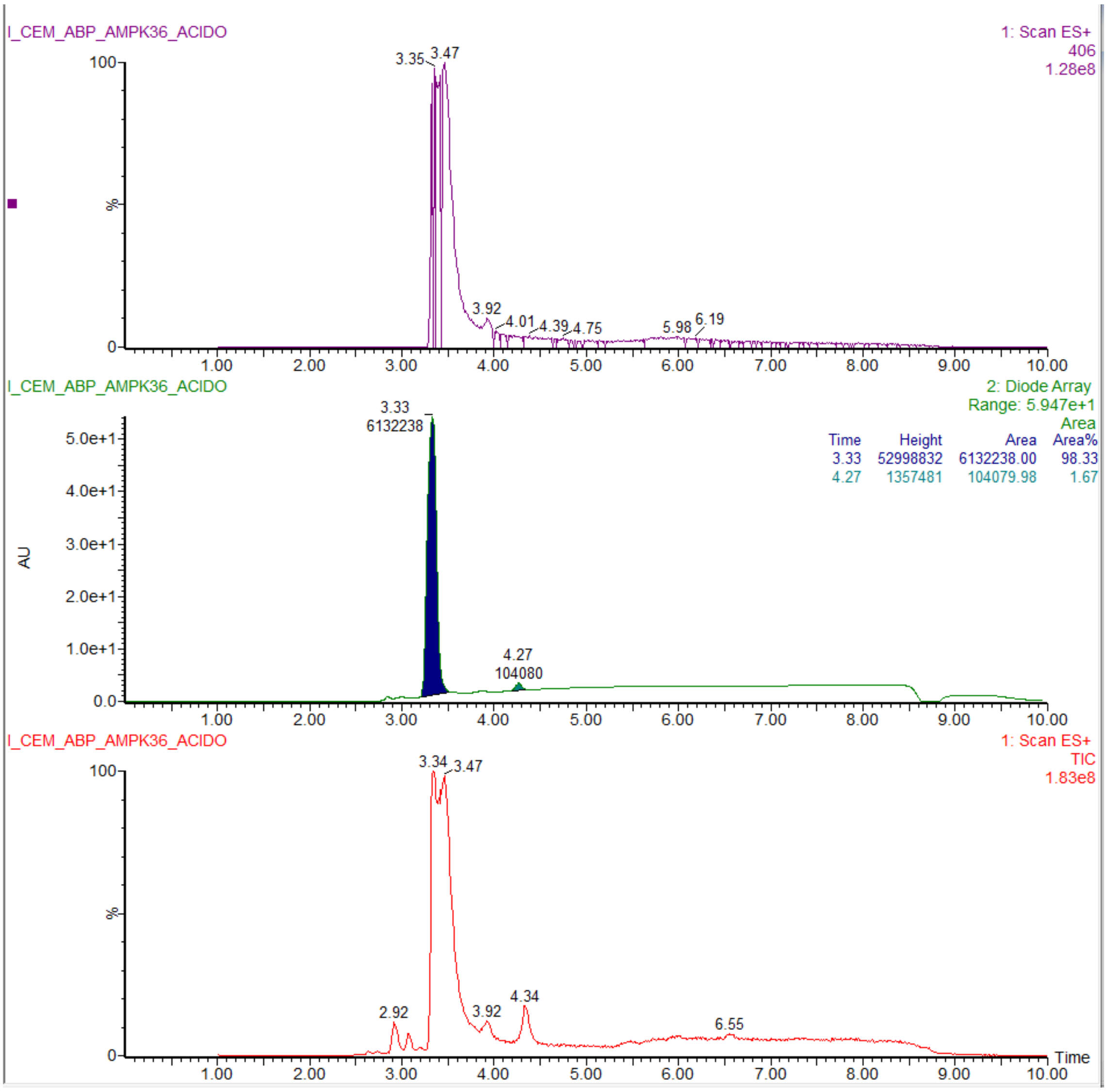

1-(Dibenzylamino)-3-(3,4-dichlorophenoxy)propan-2-ol

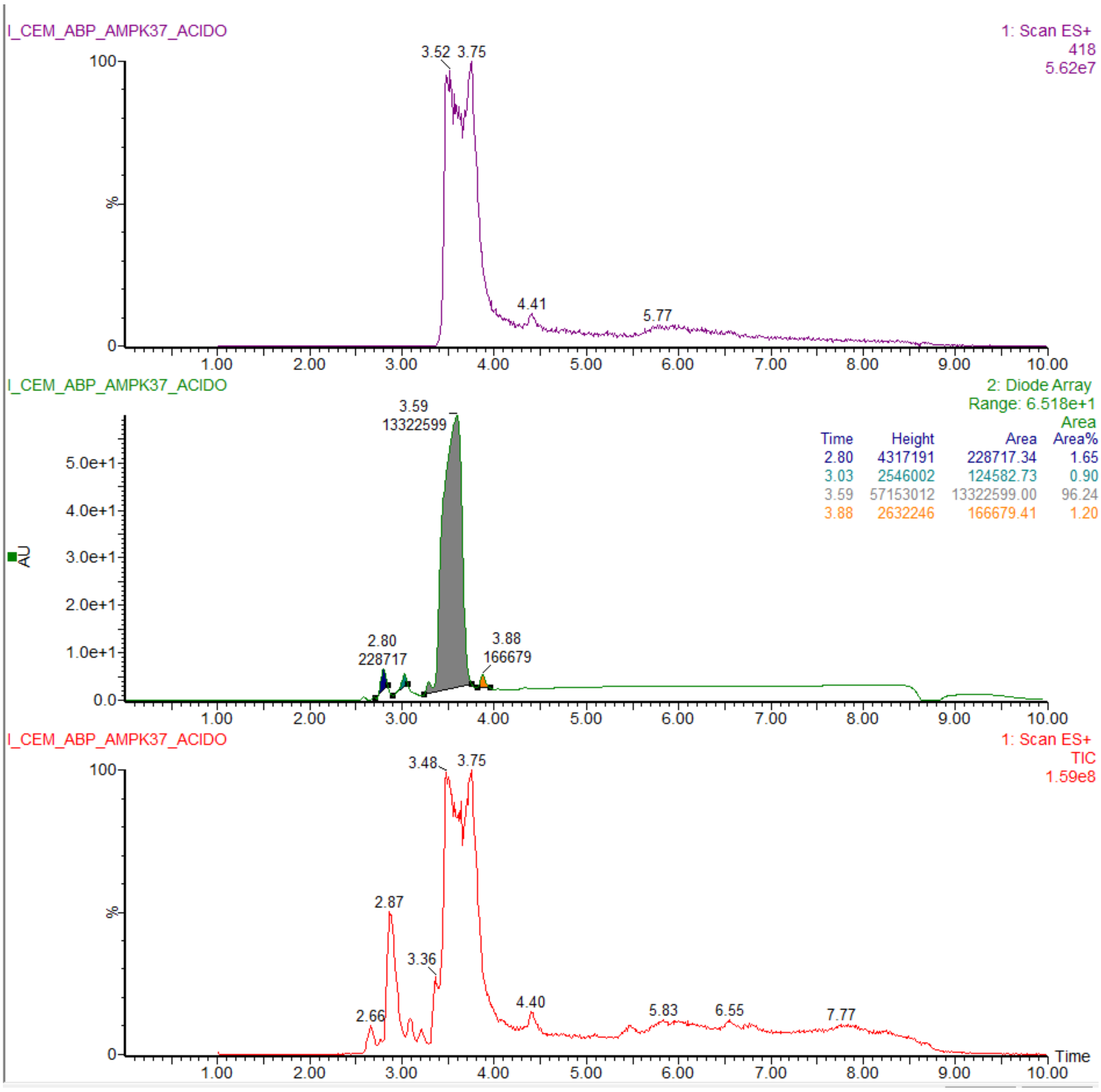

1-[4-Chloro-3-(trifluoromethyl)phenoxy]-3-(dibenzylamino)propan-2-ol

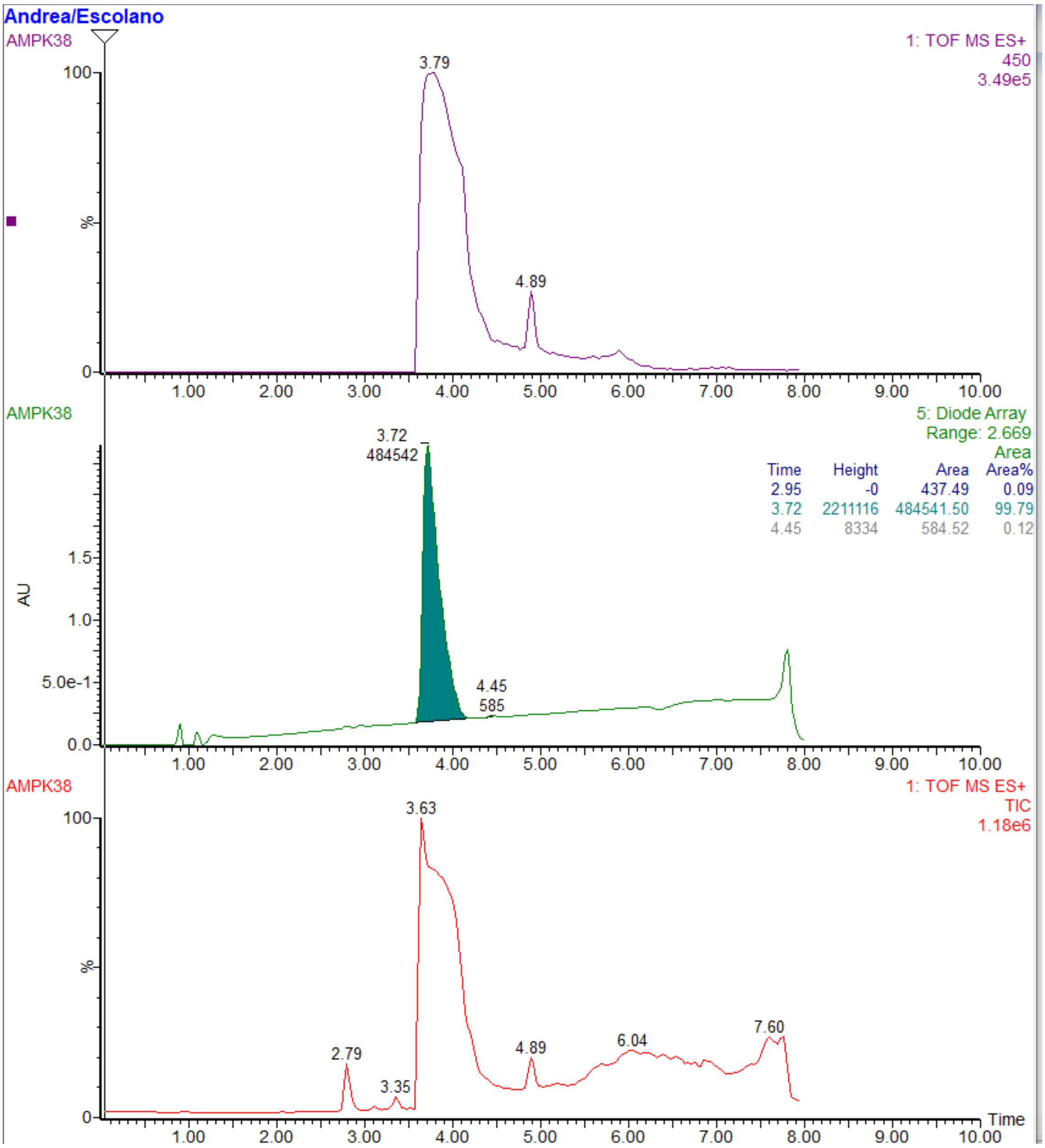

1-(Dibenzylamino)-3-[4-nitro-3-(trifluoromethyl)phenoxy]propan-2-ol

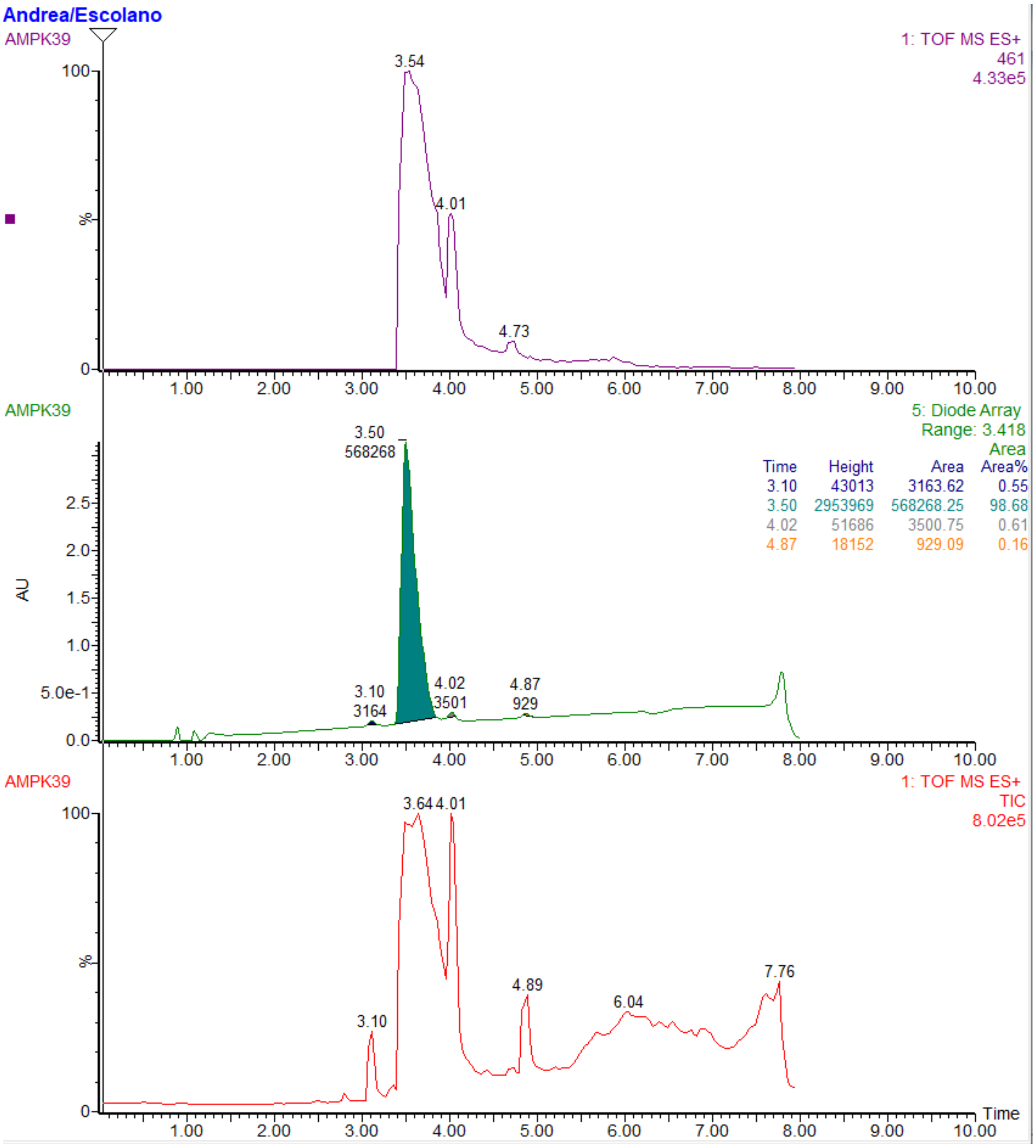

1-[Benzyl(phenyl)amino]-3-[4-(trifluoromethyl)phenoxy]propan-2-ol

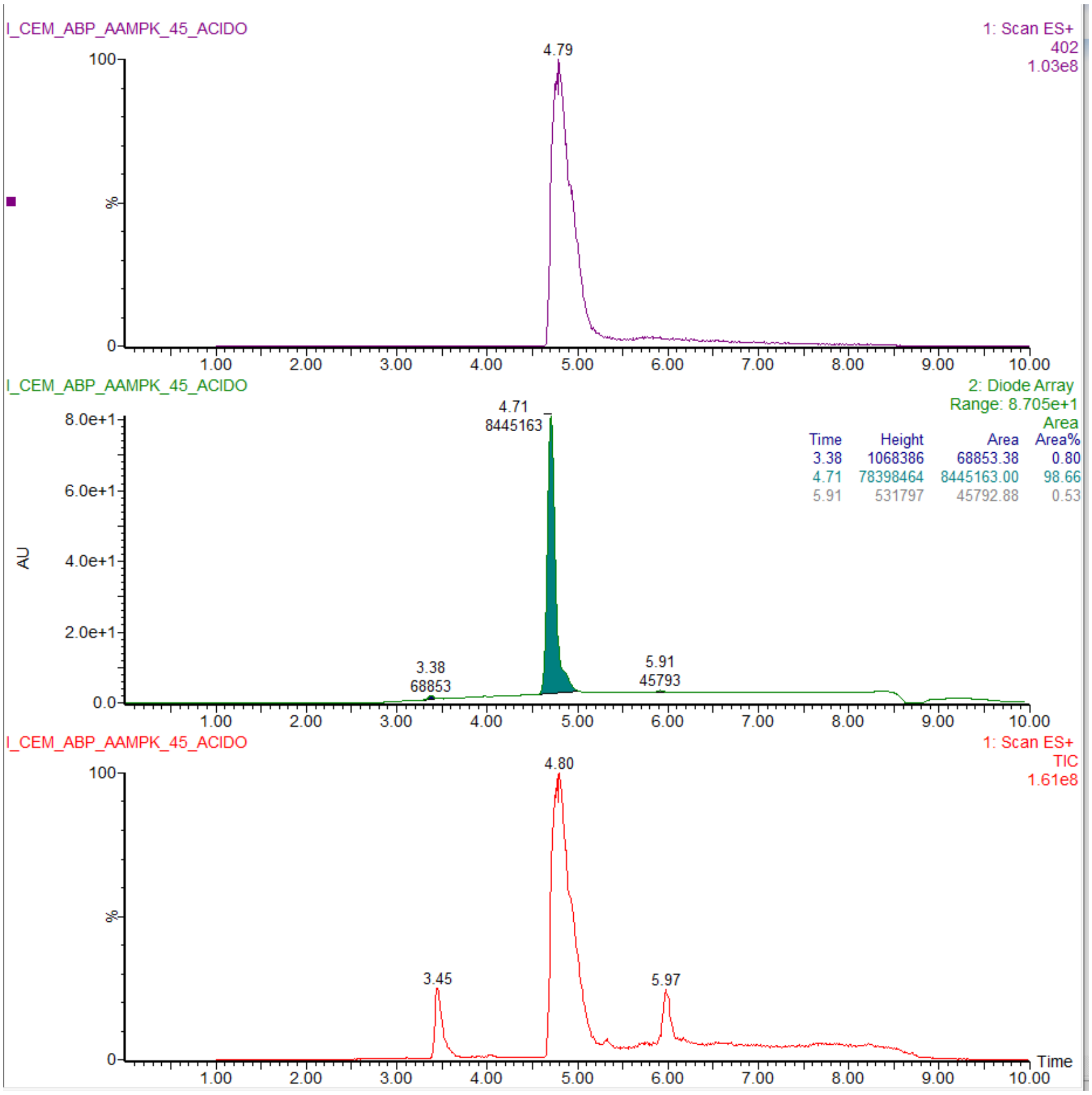

1-(Benzylamino)-3-[4-(trifluoromethyl)phenoxy]propan-2-ol

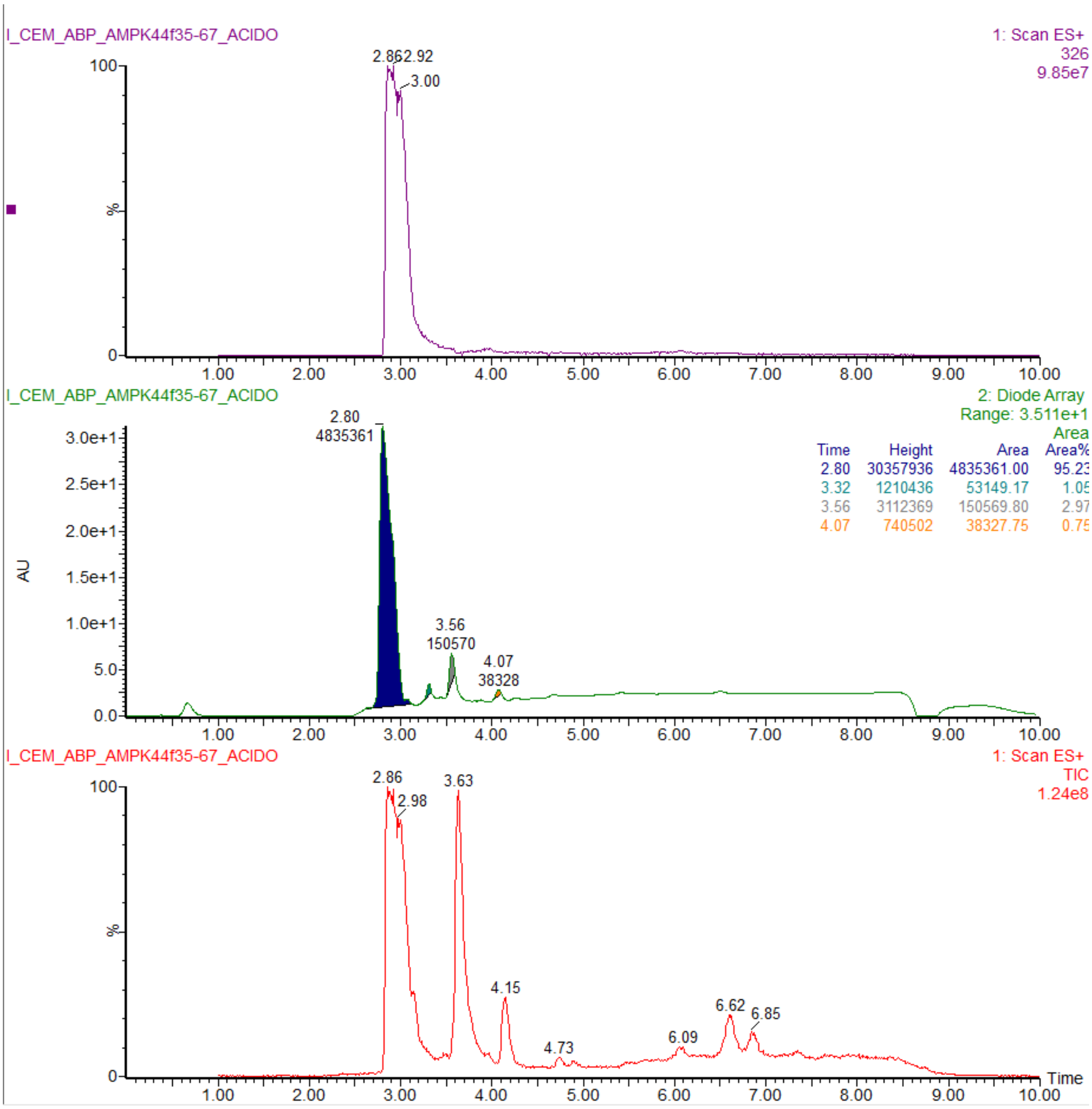

1-[Methyl(phenyl)amino]-3-[4-(trifluoromethyl)phenoxy]propan-2-ol

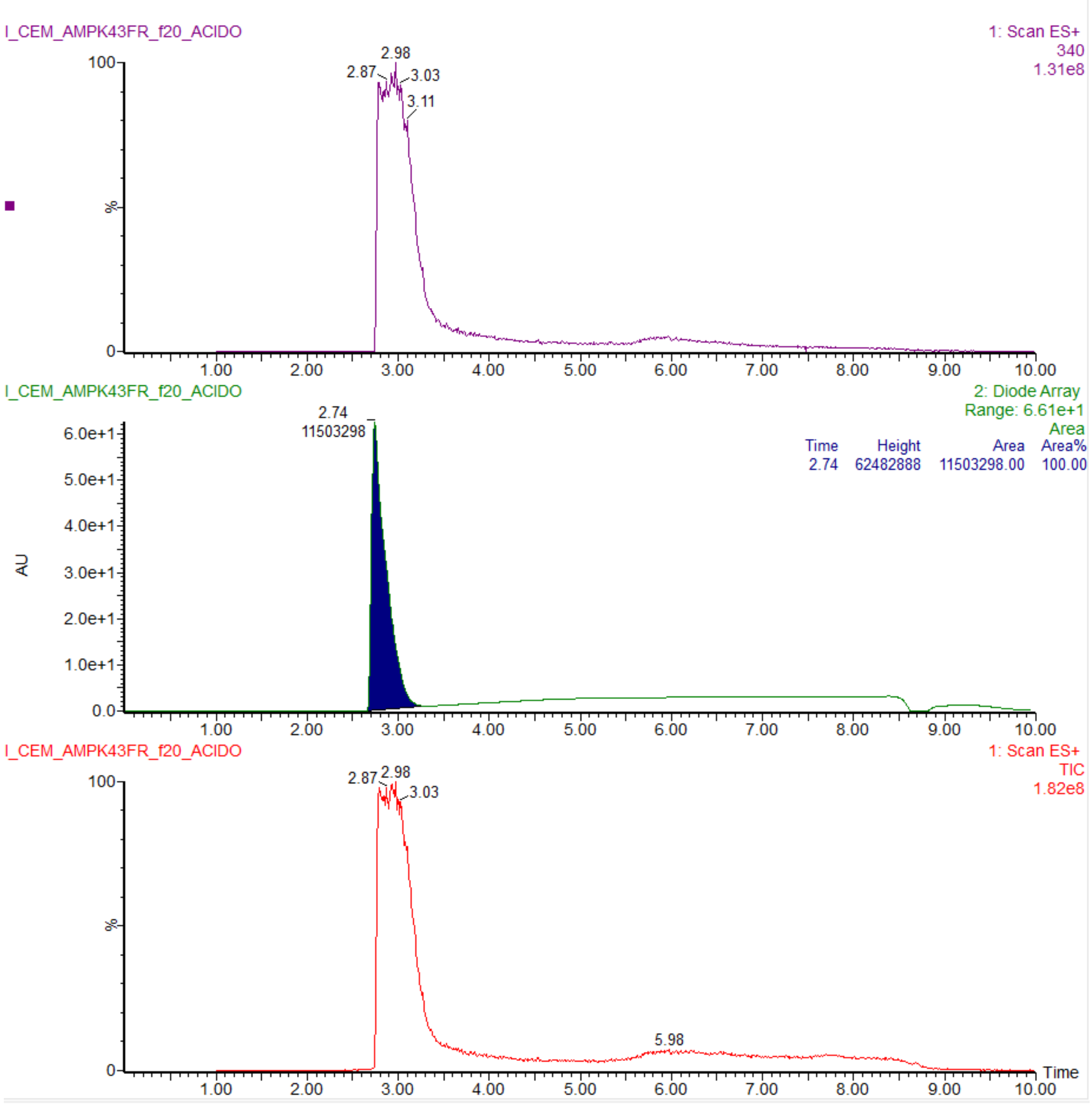

1-(Dimethylamino)-3-[4-(trifluoromethyl)phenoxy]propan-2-ol

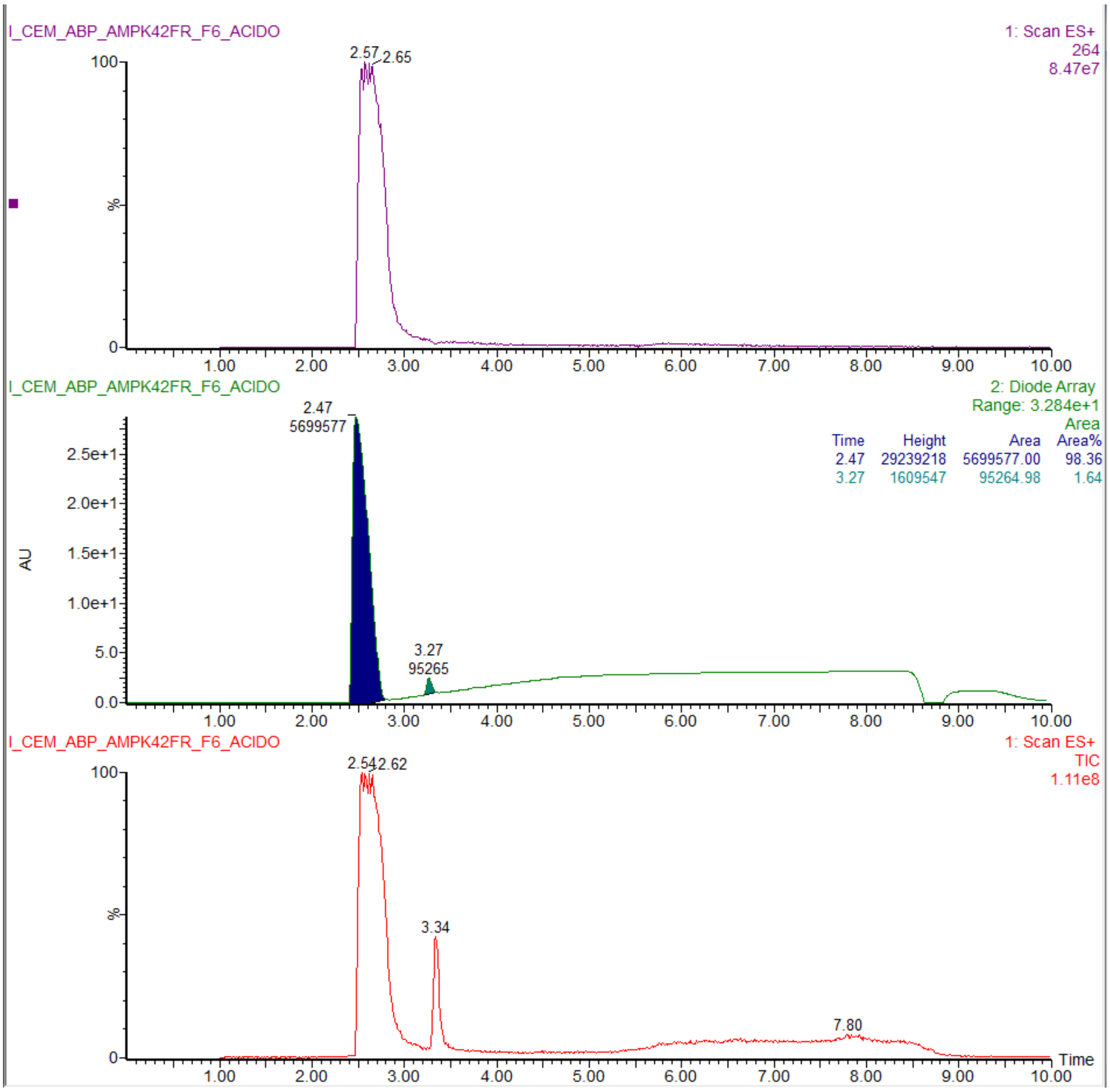

Molecular Formula Strings (Smiles)

Compound	SMILE
BC1618	OC(COC1=CC=C(C(F)(F)F)C=C1)CN(CC2=CC=CC=C2)CC3=CC=CC=C3
1	OC(COC1=CC=C(C(F)(F)F)C=C1)CNCC2=CC=CC=C2
2	OC(COC1=CC=C(C(F)(F)F)C=C1)CN(C)CC2=CC=CC=C2
3	CN(C)CC(O)COC1=CC=C(C(F)(F)F)C=C1
4	OC(COC1=CC=C(C(F)(F)F)C=C1)CN(CC2=CC=CC=C2)C3=CC=CC=C3
5	OC(COC1=CC=C(C(F)(F)F)C=C1)CN(CC2=CC=CC=C2)CC3=CC=CN=C3
6	OC(COC1=CC=CC=C1)CN(CC2=CC=CC=C2)CC3=CC=CC=C3
7	ClC1=CC=C(OCC(O)CN(CC2=CC=CC=C2)CC3=CC=CC=C3)C=C1
8	CC1=CC=C(OCC(O)CN(CC2=CC=CC=C2)CC3=CC=CC=C3)C=C1
9	OC(COC1=CC=C(C(C)C)C=C1)CN(CC2=CC=CC=C2)CC3=CC=CC=C3
10	OC(COC1=CC=C(C(C)(C)C)C=C1)CN(CC2=CC=CC=C2)CC3=CC=CC=C3
11	OC(COC1=CC=C(C2CCCCC2)C=C1)CN(CC3=CC=CC=C3)CC4=CC=CC=C4
12	OC(COC1=CC=C(C2CCCC2)C=C1)CN(CC3=CC=CC=C3)CC4=CC=CC=C4
13	CC1=C(C)C=C(OCC(O)CN(CC2=CC=CC=C2)CC3=CC=CC=C3)C=C1
14	OC(COC1=CC=C(OC)C=C1)CN(CC2=CC=CC=C2)CC3=CC=CC=C3
15	OC(COC1=CC=C(OCC2=CC=CC=C2)C=C1)CN(CC3=CC=CC=C3)CC4=CC=CC=C4
16	OC(COC1=CC=C(OCCC)C=C1)CN(CC2=CC=CC=C2)CC3=CC=CC=C3
17	ClC1=C(Cl)C=C(OCC(O)CN(CC2=CC=CC=C2)CC3=CC=CC=C3)C=C1
18	ClC1=C(C(F)(F)F)C=C(OCC(O)CN(CC2=CC=CC=C2)CC3=CC=CC=C3)C=C1
19	OC(COC1=CC(C(F)(F)F)=C([N+]([O-])=O)C=C1)CN(CC2=CC=CC=C2)CC3=CC=CC=C3
20	ClC1=CC(Cl)=C(OCC(O)CN(CC2=CC=CC=C2)CC3=CC=CC=C3)C=C1
21	BrC1=CC=C(OCC(O)CN(CC2=CC=CC=C2)CC3=CC=CC=C3)C=C1
22	OC(COC1=CC=C([N+]([O-])=O)C=C1)CN(CC2=CC=CC=C2)CC3=CC=CC=C3
23	IC1=CC=C(OCC(O)CN(CC2=CC=CC=C2)CC3=CC=CC=C3)C=C1
24	OC(COC1=CC=C(S(F)(F)(F)(F)F)C=C1)CN(CC2=CC=CC=C2)CC3=CC=CC=C3
25	ClC1=CC=C(OCC(O)CN(CC2=CC=C(Cl)C=C2)CC3=CC=CC=C3)C=C1
26	OC(COC1=CC=C(Cl)C=C1)CN(CC2=CC=C(Cl)C=C2)CC3=CC=C(Cl)C=C3
27	ClC1=CC=C(OCC(O)CN(CC2=CC=C(C)C=C2)CC3=CC=CC=C3)C=C1
28	ClC1=CC=C(OCC(O)CN(CC2=CC=C(C)C=C2)CC3=CC=C(C)C=C3)C=C1
29	ClC1=CC=C(OCC(O)CN(CC2=CC=C(C)C=C2)CC3=CC=CC=C3)C=C1
30	OC(COC1=CC=C(Cl)C=C1)CN(CC2=CC=C(Cl)C(Cl)=C2)CC3=CC=CC=C3
31	OC(COC1=CC=C(Cl)C=C1)CN(CC2=CC=C(Cl)C(Cl)=C2)CC3=CC=C(Cl)C=C3
32	OC(COC1=CC=C(Cl)C=C1)CN(CC2=CC=C(Cl)C(Cl)=C2)CC3=CC=C(C)C=C3
33	ClC1=CC=C(OCC(O)CN(CC2=CC=CC=C2)CC3=CC=C(OC)C=C3)C=C1
34	ClC1=CC=C(OCC(O)CN(CC2=CC=C(OC)C=C2)CC3=CC=C(OC)C=C3)C=C1
35	OC(COC1=CC=C(Cl)C=C1)CN(CC2=CC=C(Cl)C(Cl)=C2)CC3=CC=C(OC)C=C3

1-(4-Chlorophenoxy)-3-(dibenzylamino)propan-2-ol

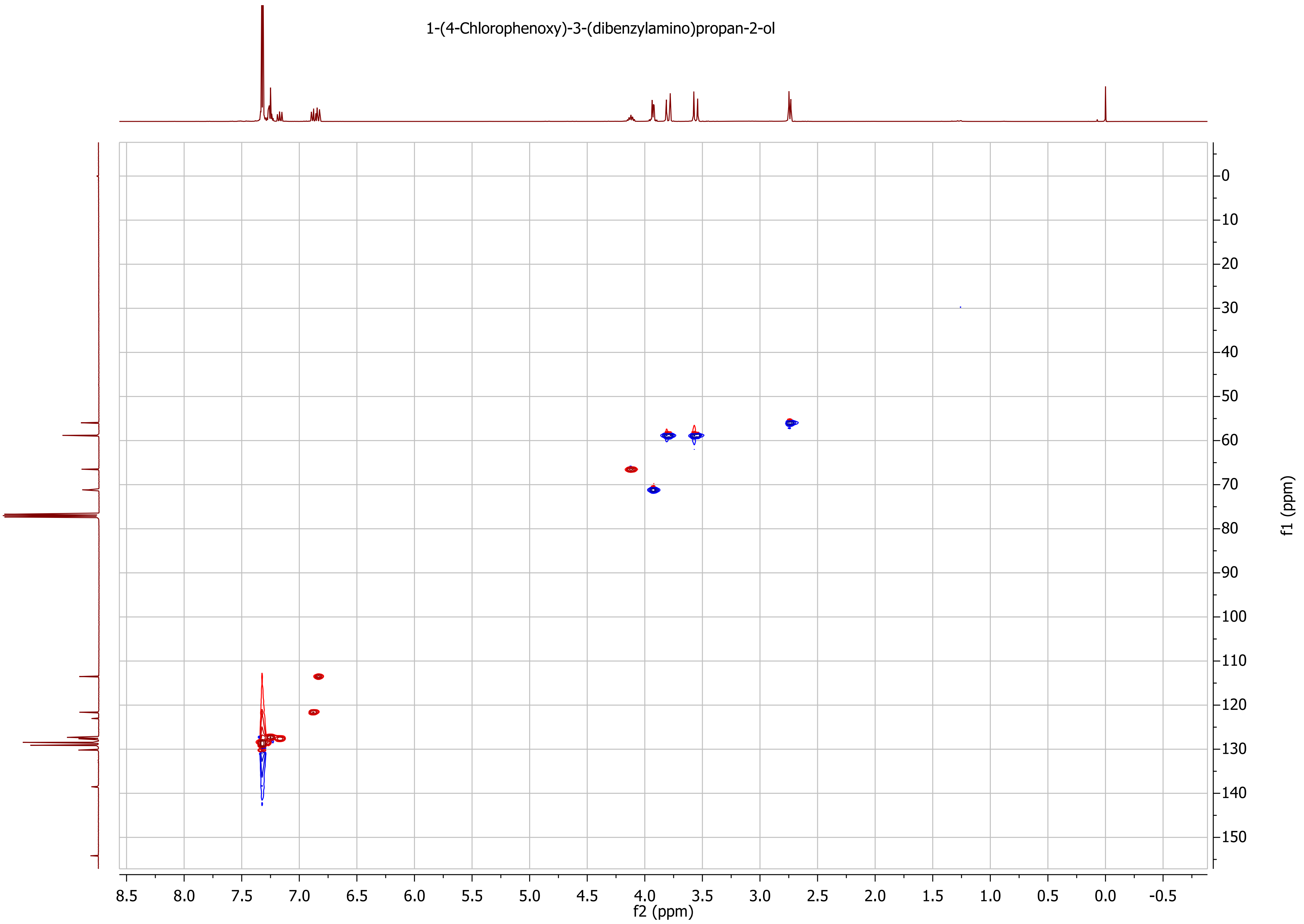

1-(Dibenzylamino)-3-(p-tolyloxy)propan-2-ol

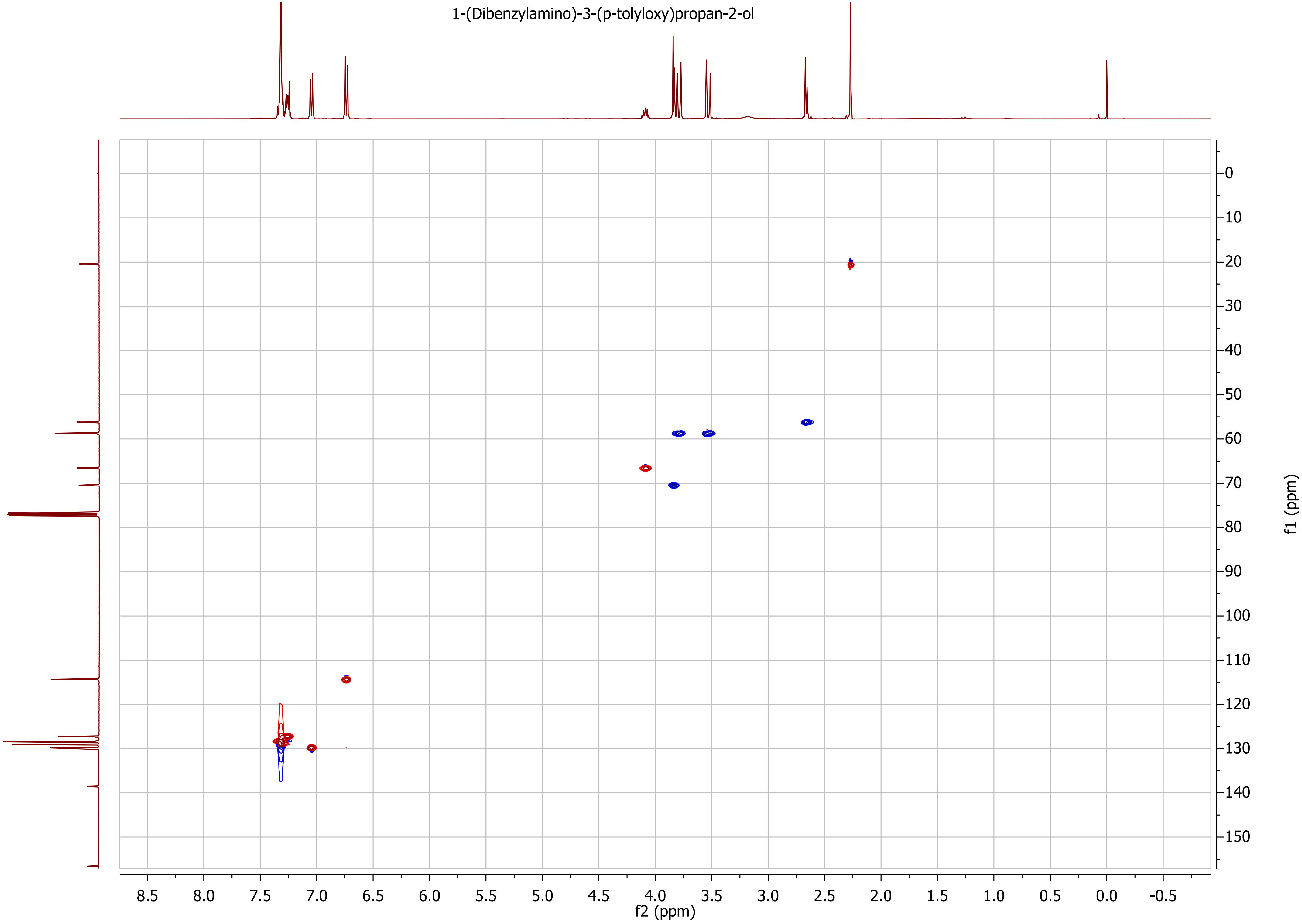

1-(4-Cyclohexylphenoxy)-3-(dibenzylamino)propan-2-ol

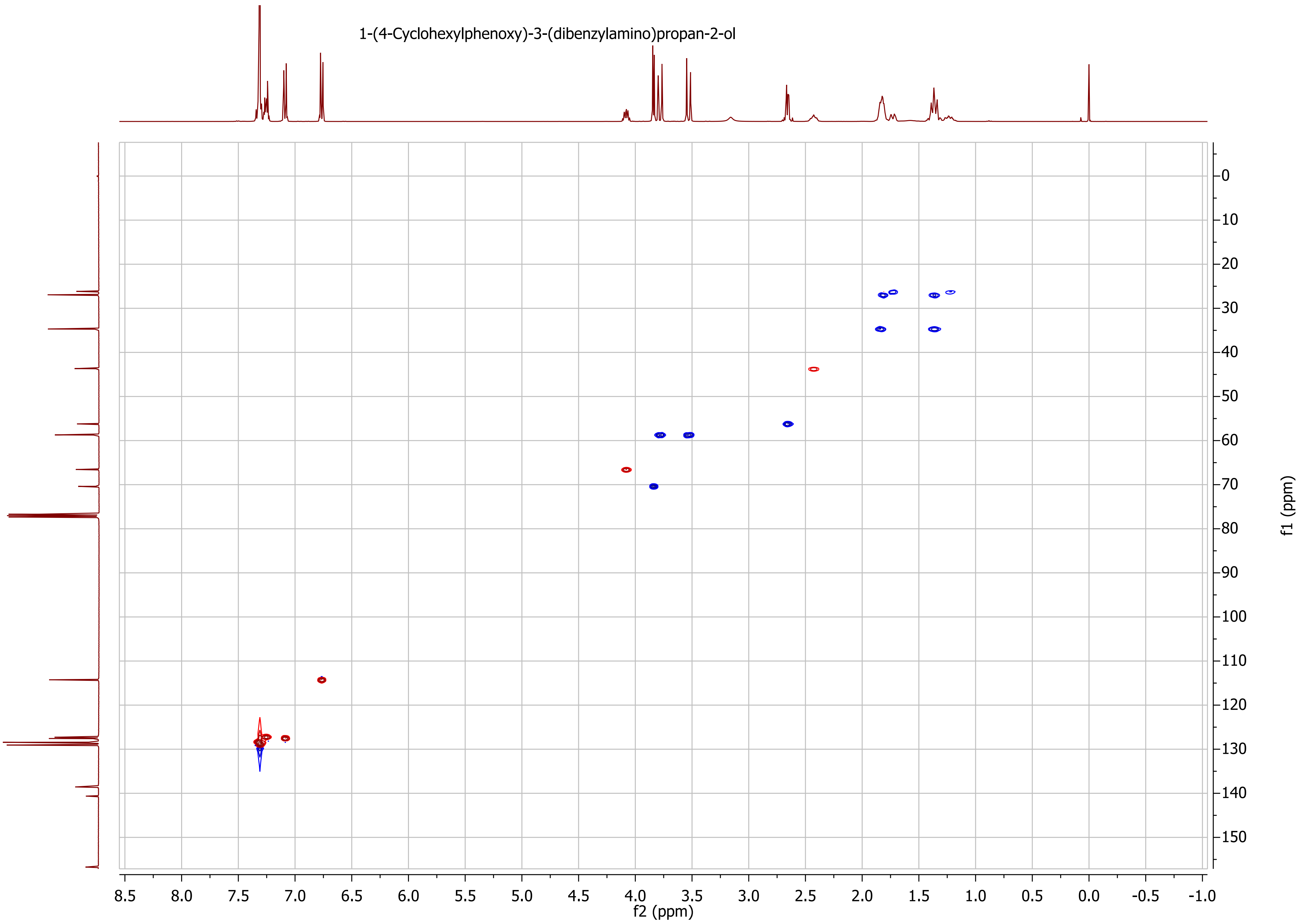

1-[4-(Tert-butyl)phenoxy]-3-(dibenzylamino)propan-2-ol

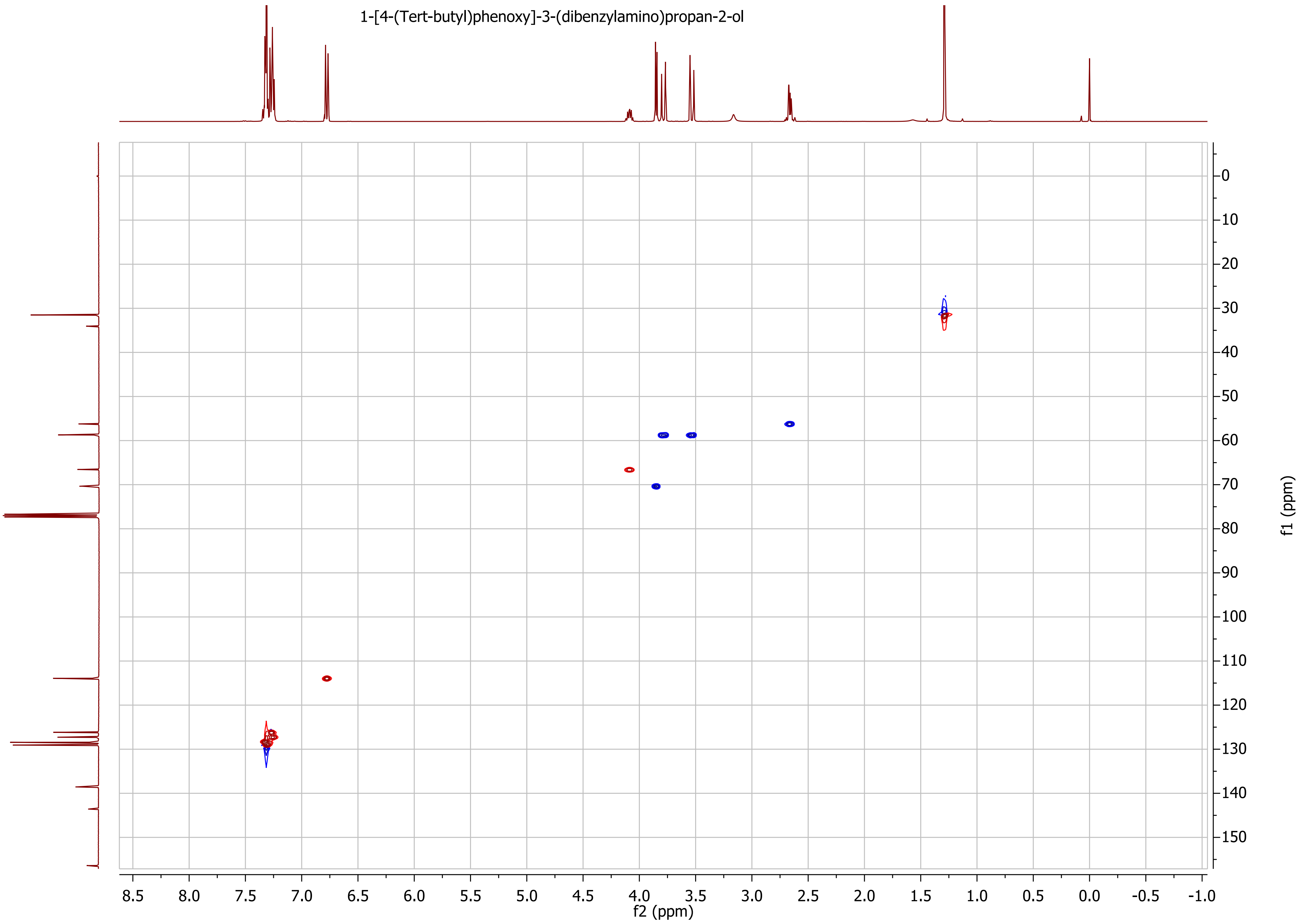

1-[4-(Benzyloxy)phenoxy]-3-(dibenzylamino)propan-2-ol

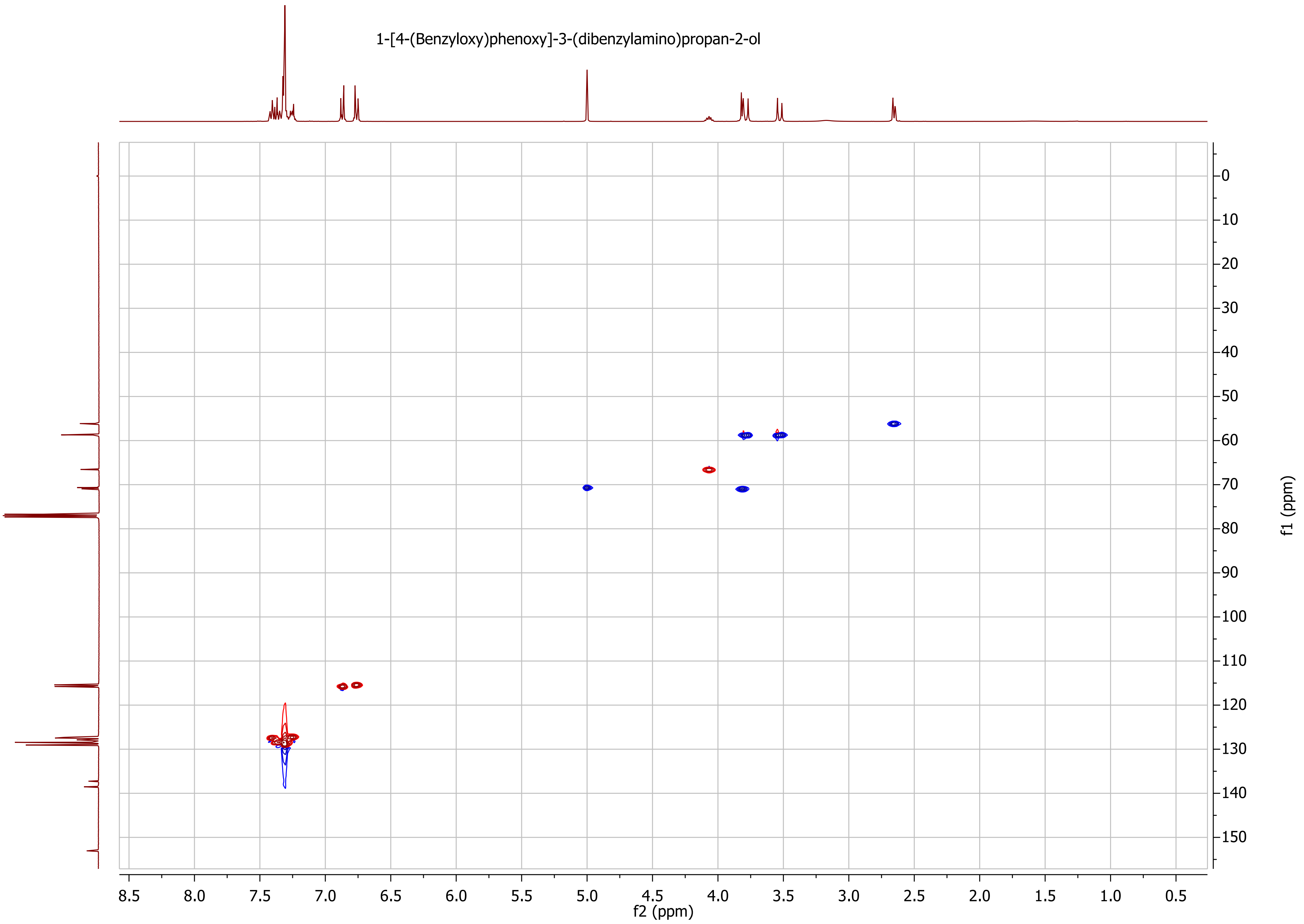

1-(Dibenzylamino)-3-(2,4-dichlorophenoxy)propan-2-ol

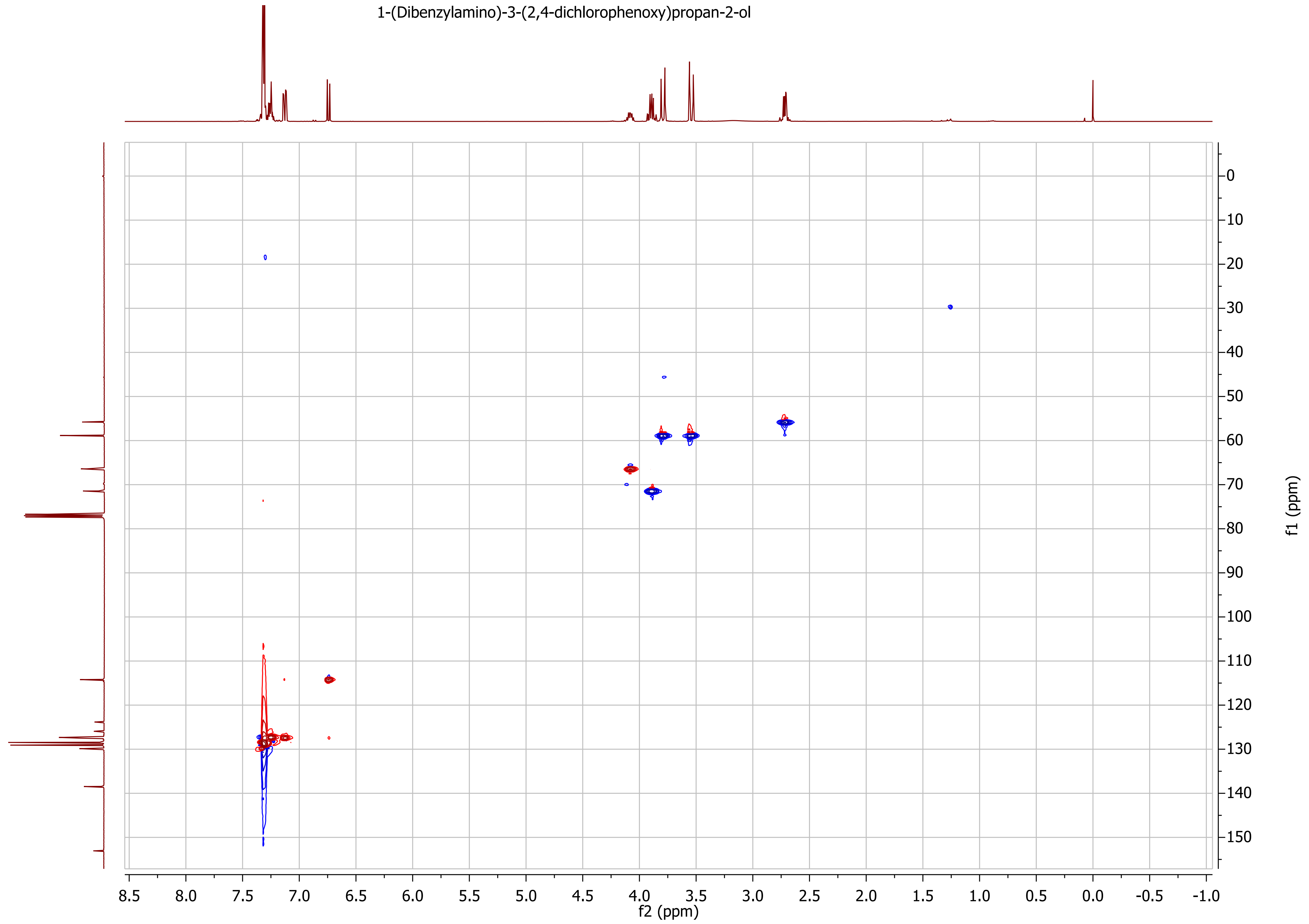

1-(4-Cyclopentylphenoxy)-3-(dibenzylamino)propan-2-ol

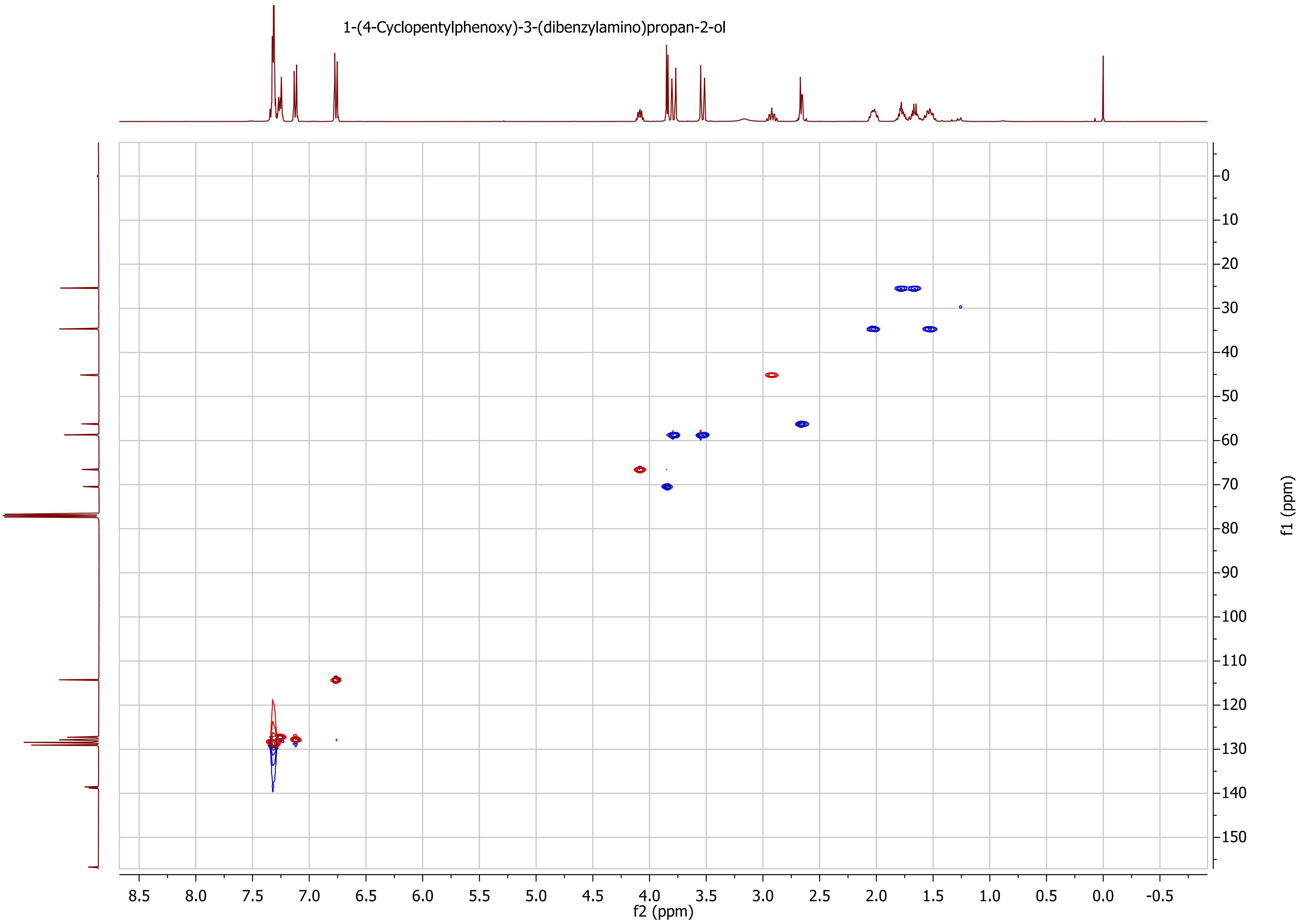

1-(Dibenzylamino)-3-(4-isopropylphenoxy)propan-2-ol

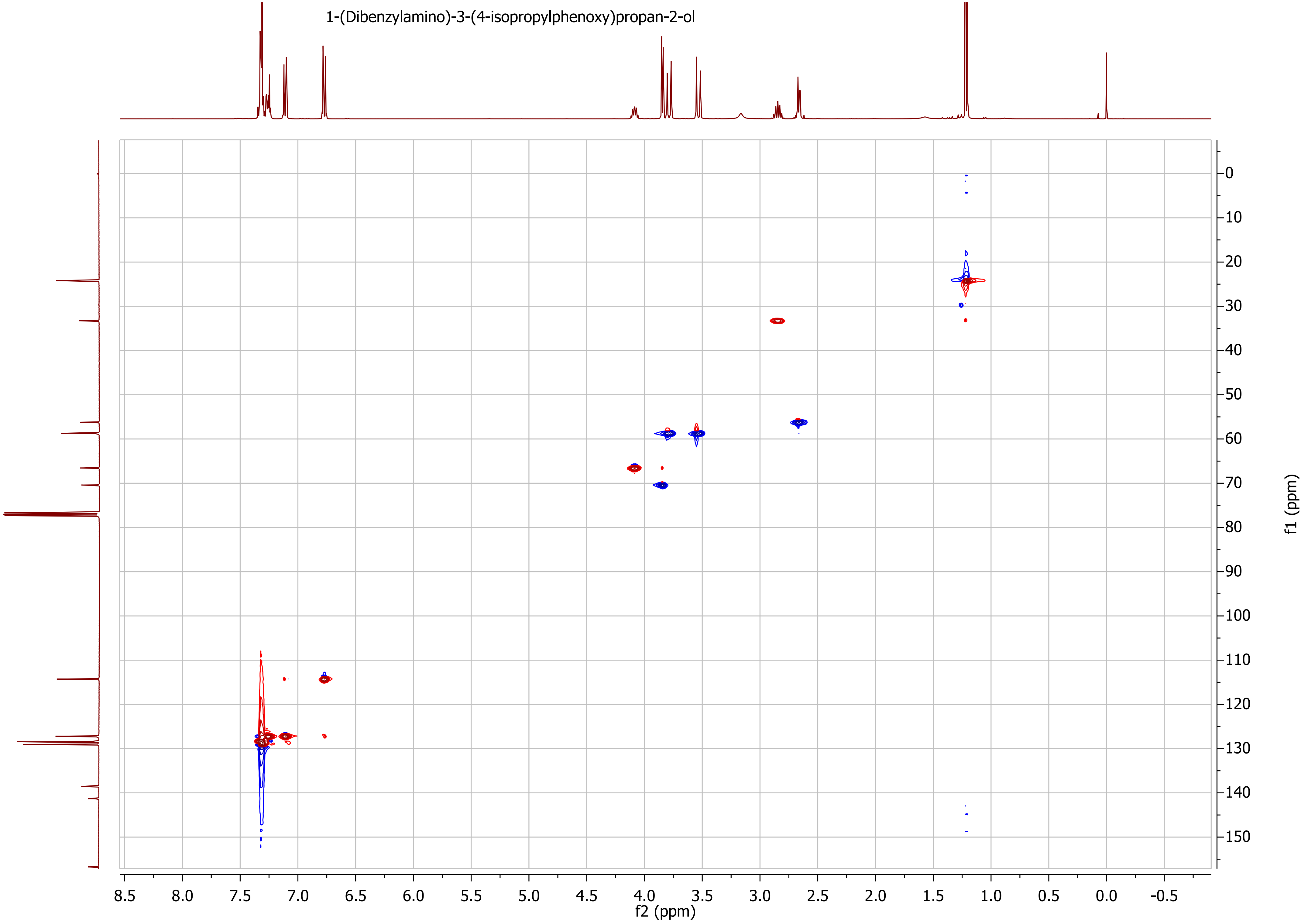

1-(Dibenzylamino)-3-phenoxypropan-2-ol

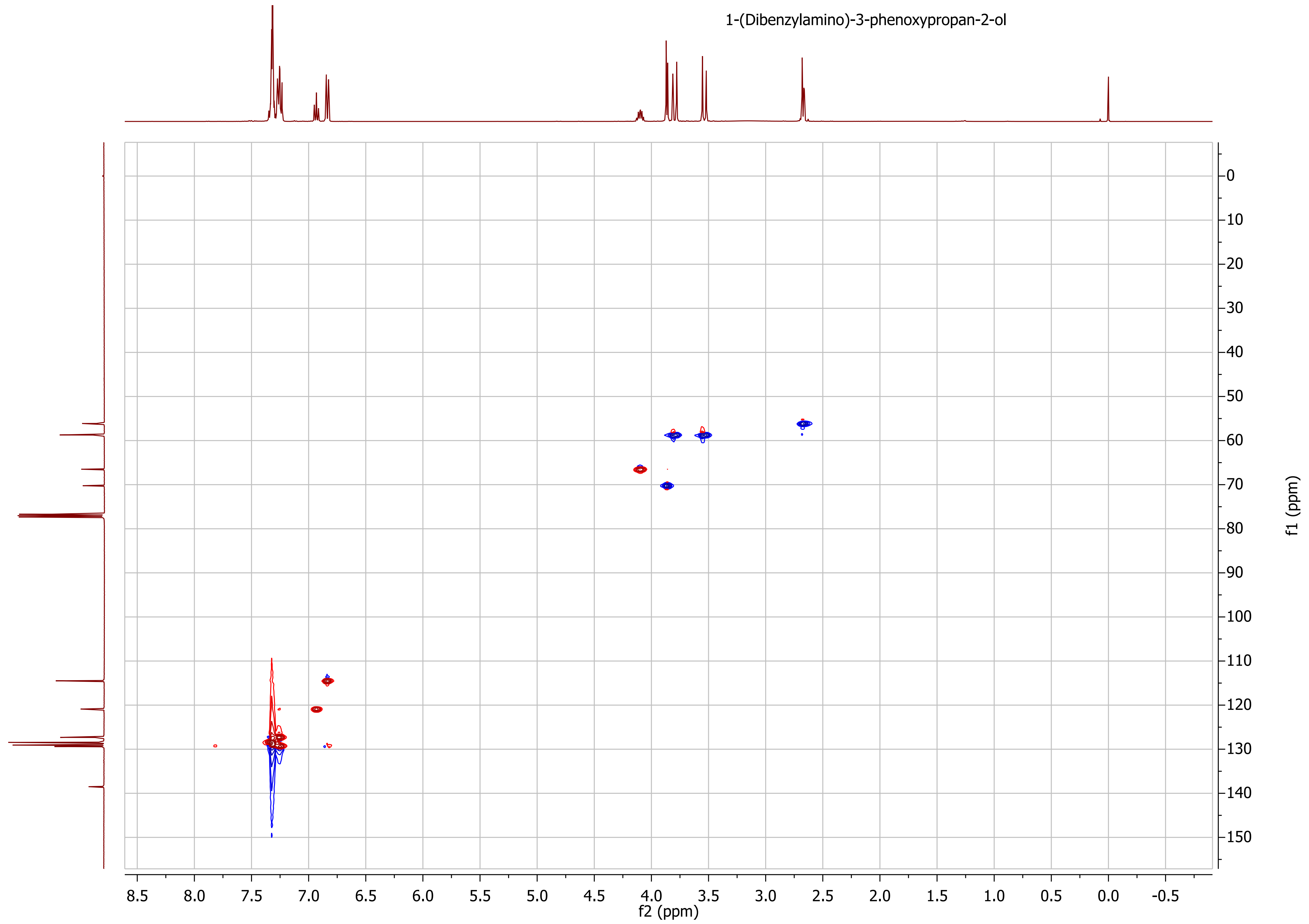

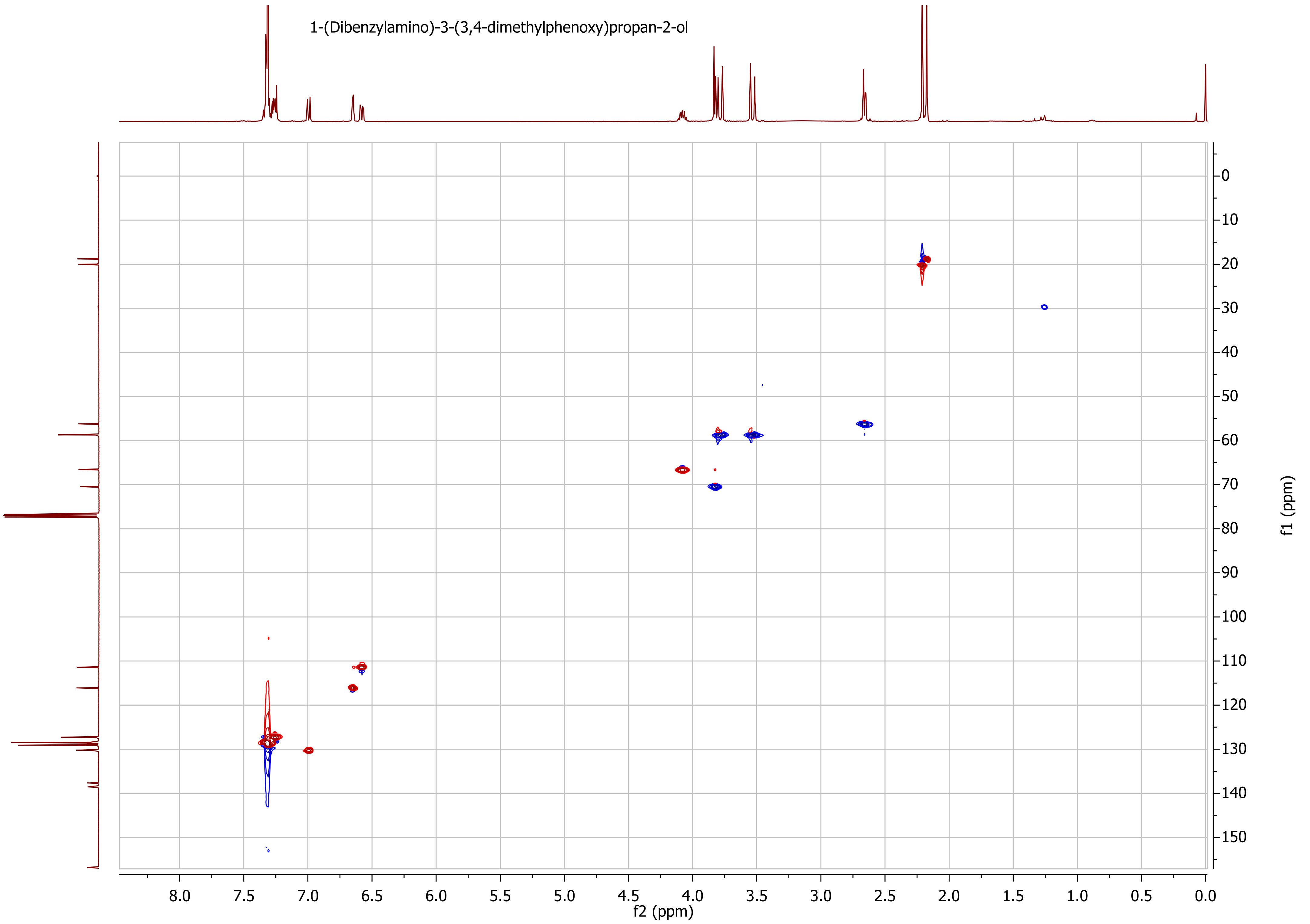

1-(Dibenzylamino)-3-(3,4-dimethylphenoxy)propan-2-ol
f2 (ppm)
f1 (ppm)
8.0
7.5
7.0
6.5
6.0
5.5
5.0
4.5
4.0
3.5
3.0
2.5
2.0
1.5
1.0
0.5
0.0
0
10
20
30
40
50
60
70
80
90
100
110
120
130
140
150

1-(Dibenzylamino)-3-(4-methoxyphenoxy)propan-2-ol

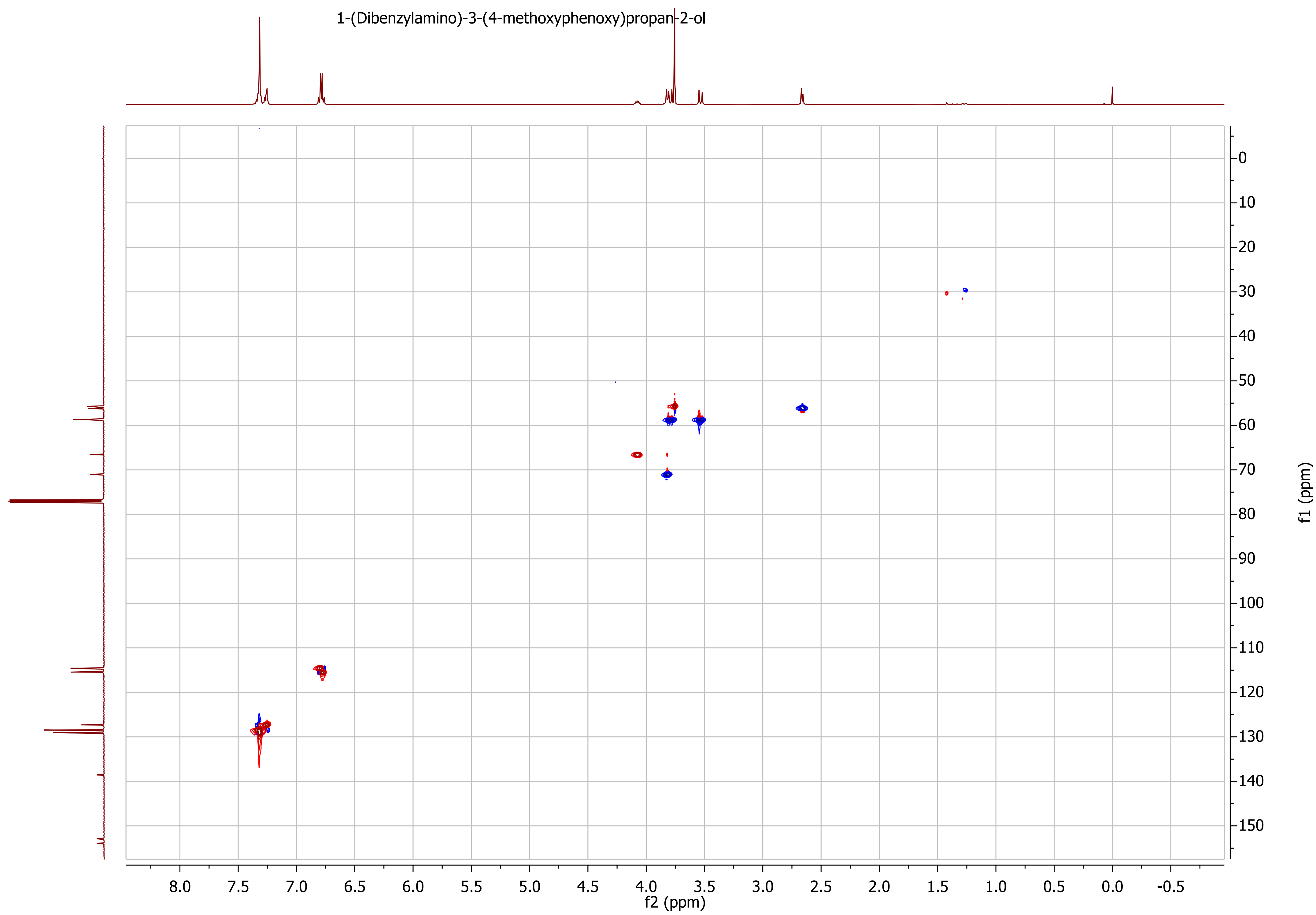

1-(Dibenzylamino)-3-(4-propoxyphenoxy)propan-2-ol

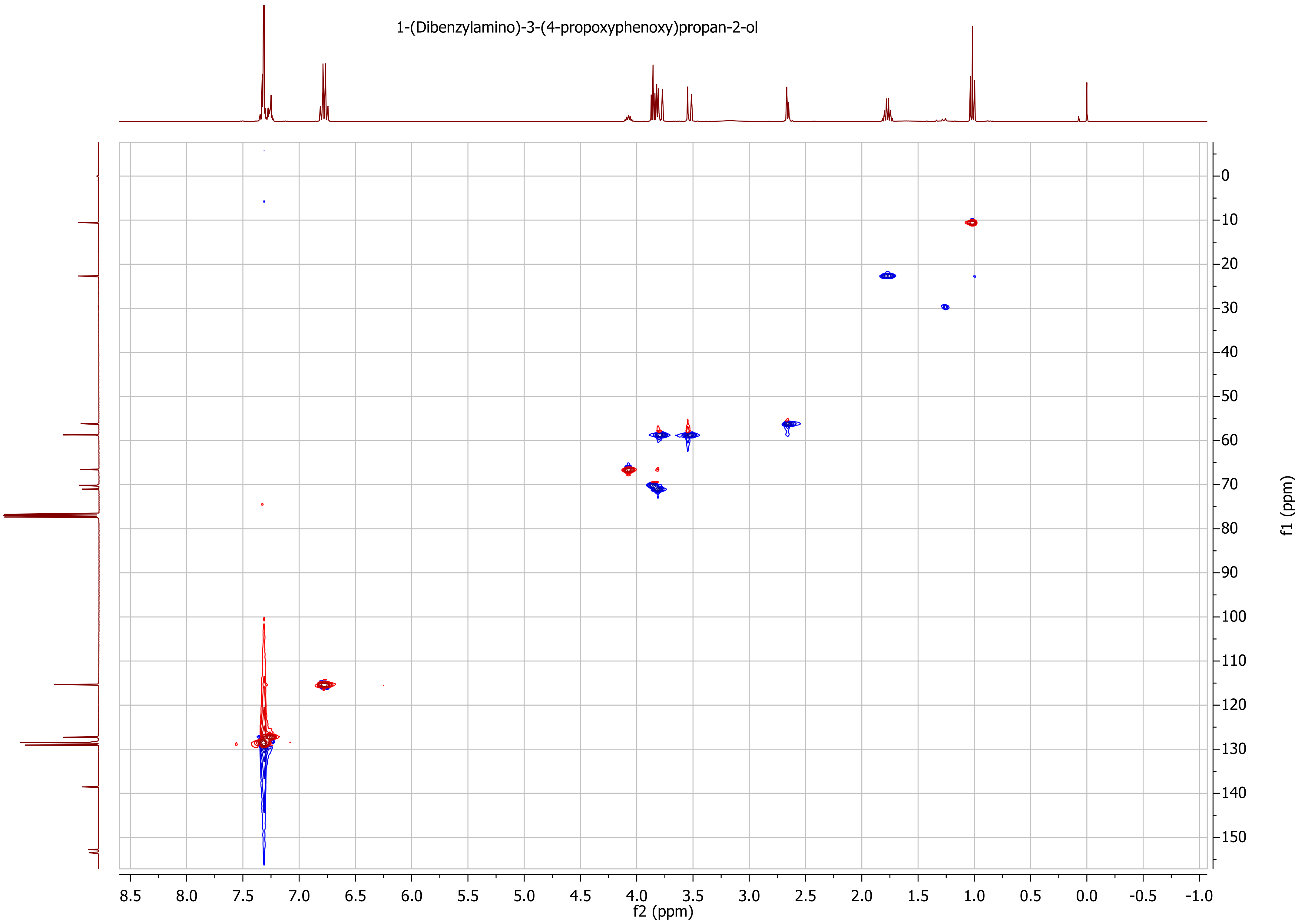

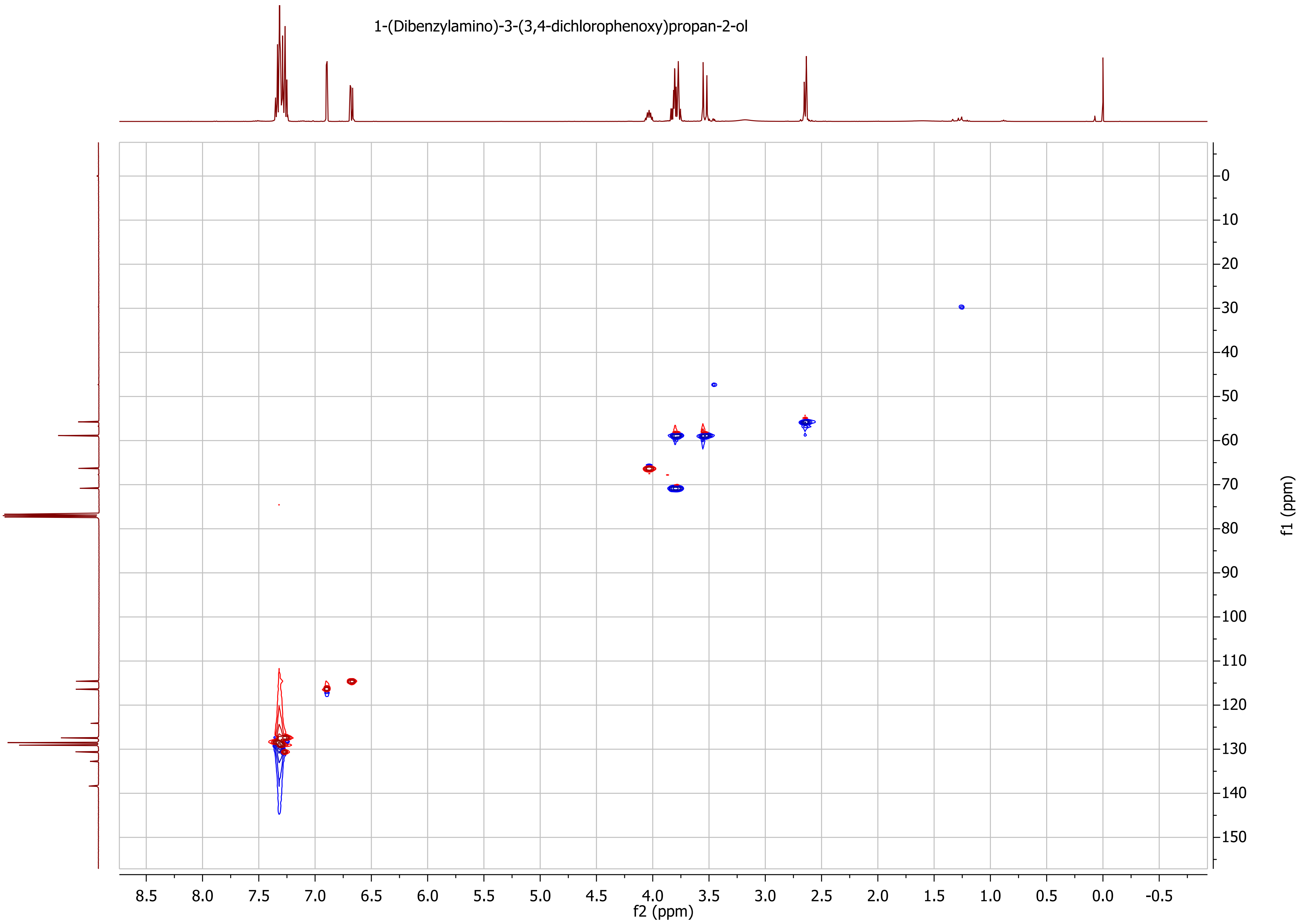

1-(Dibenzylamino)-3-(3,4-dichlorophenoxy)propan-2-ol
f2 (ppm)
f1 (ppm)

1-[4-Chloro-3-(trifluoromethyl)phenoxy]-3-(dibenzylamino)propan-2-ol

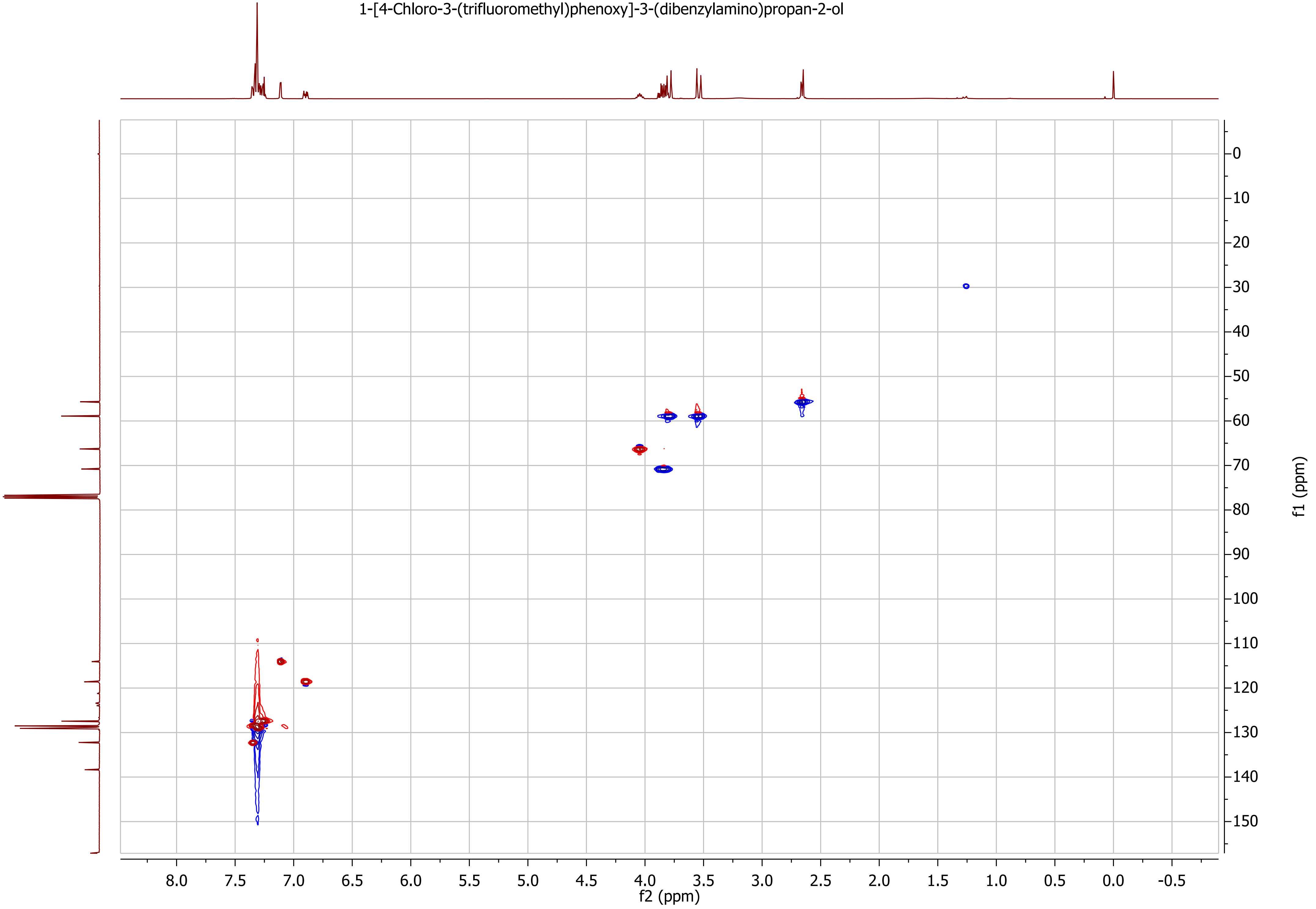

1-(Dibenzylamino)-3-[4-nitro-3-(trifluoromethyl)phenoxy]propan-2-ol

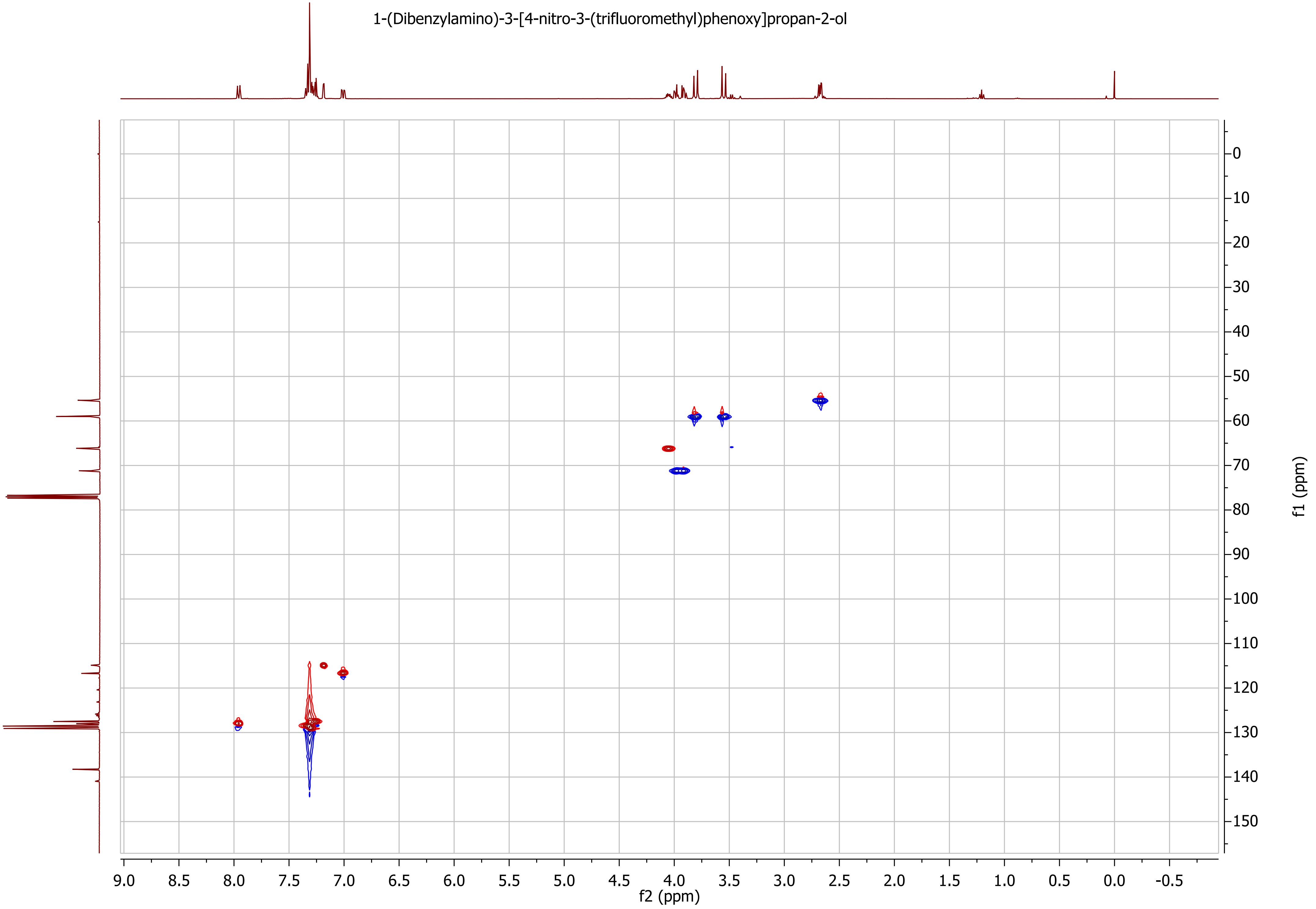

1-[Benzyl(pyridin-3-ylmethyl)amino]-3-[4-(trifluoromethyl)phenoxy]propan-2-ol

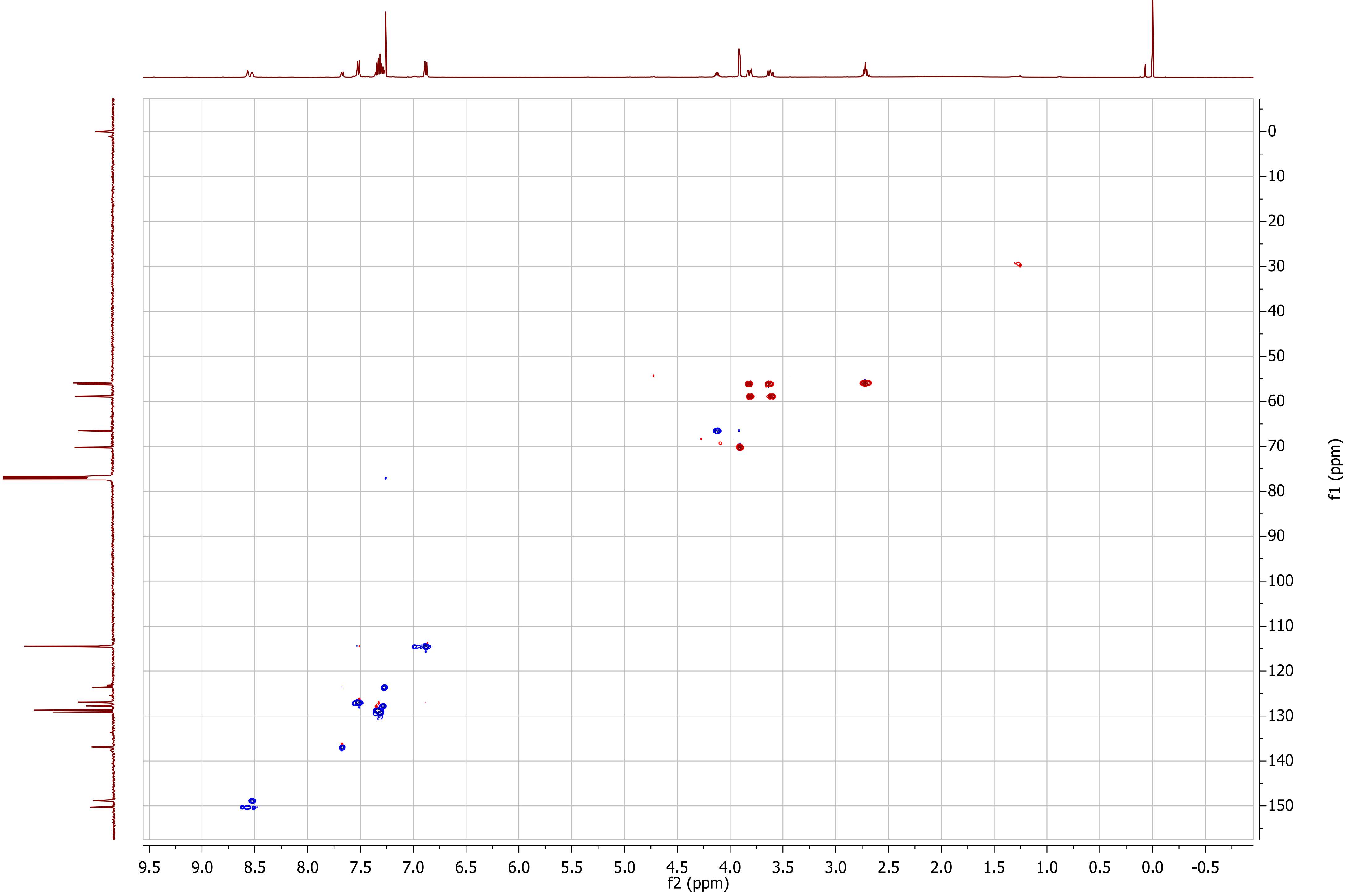

1-[Benzyl(phenyl)amino]-3-[4-(trifluoromethyl)phenoxy]propan-2-ol

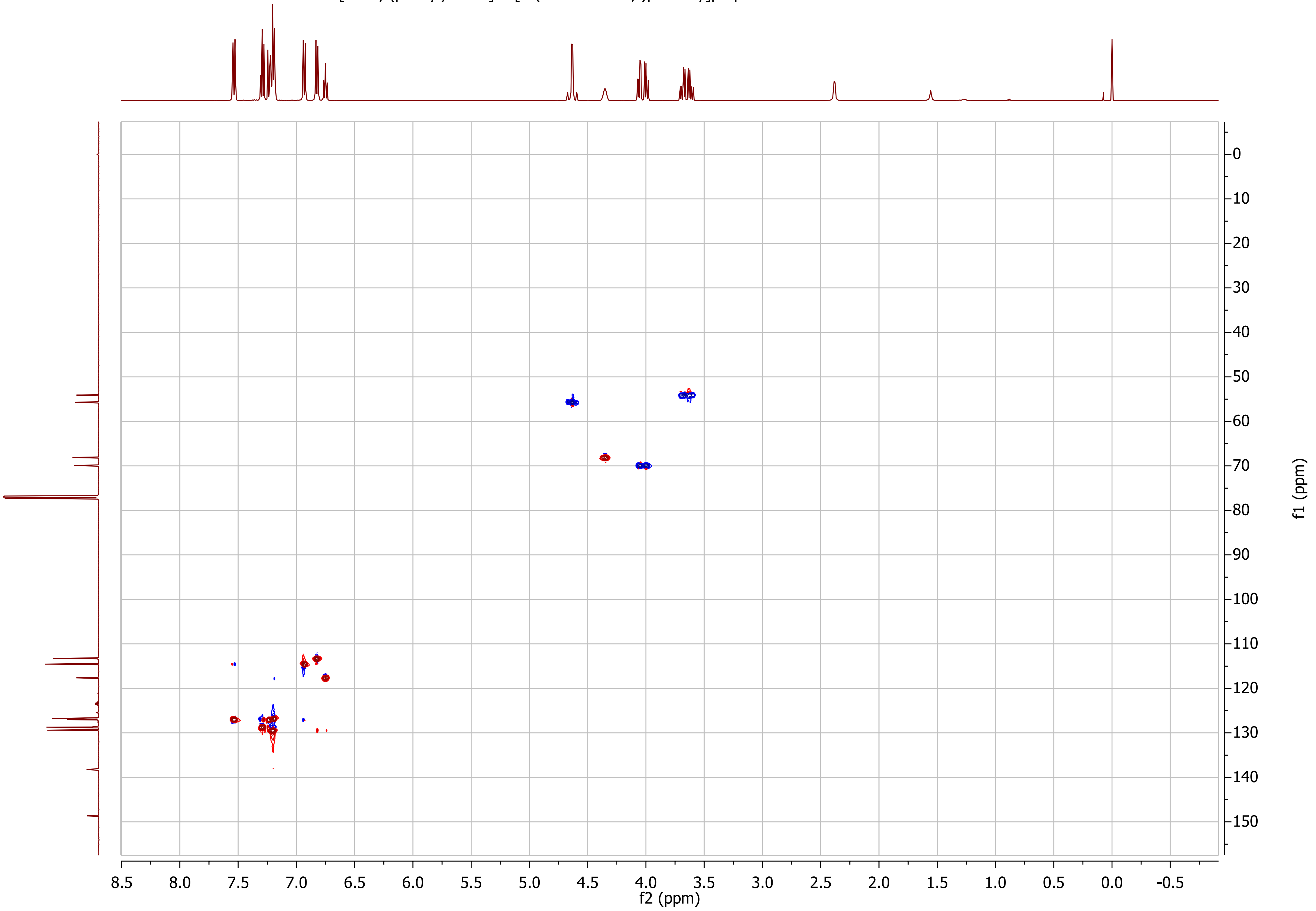

1-(Benzylamino)-3-[4-(trifluoromethyl)phenoxy]propan-2-ol

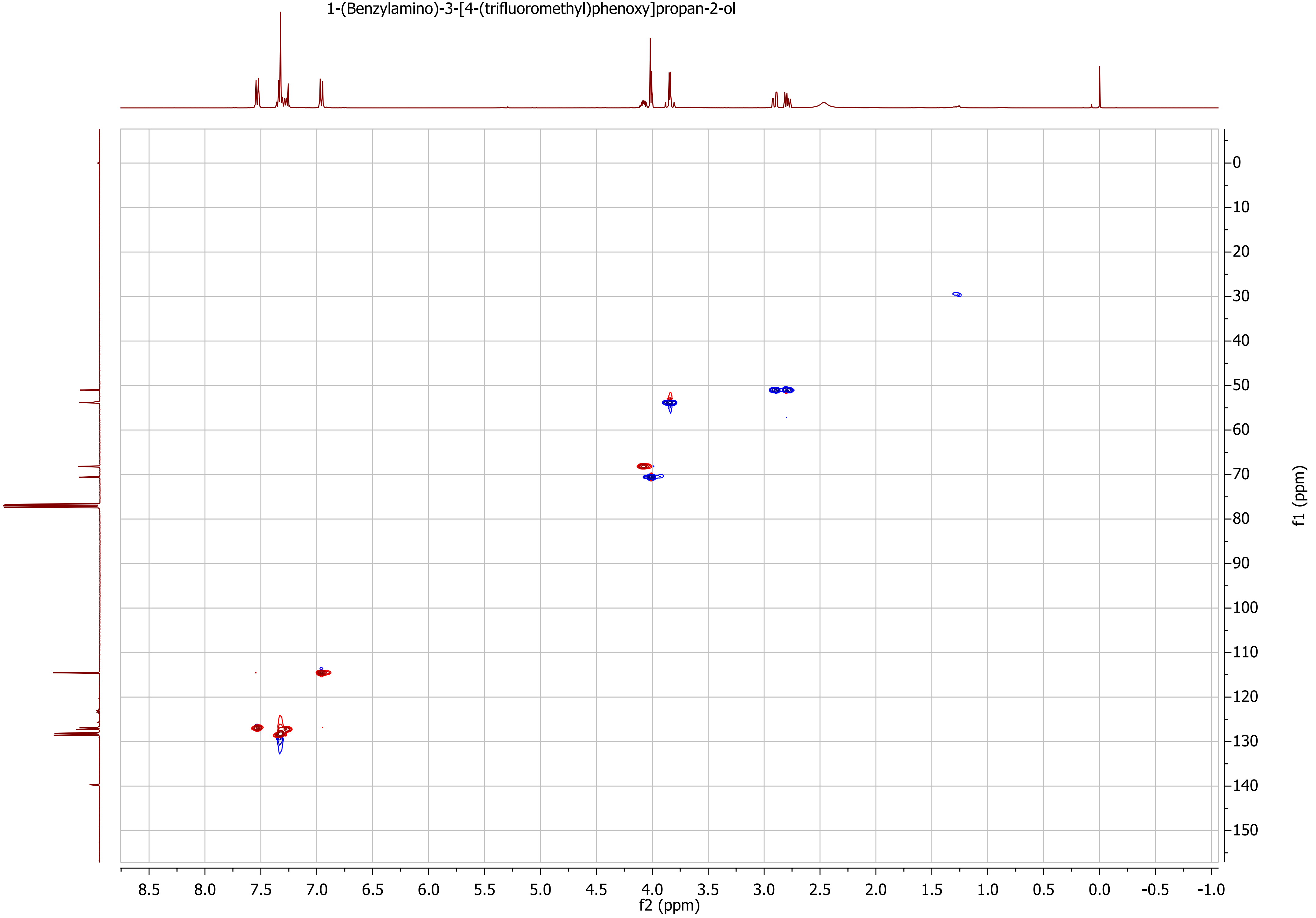

1-[Methyl(phenyl)amino]-3-[4-(trifluoromethyl)phenoxy]propan-2-ol

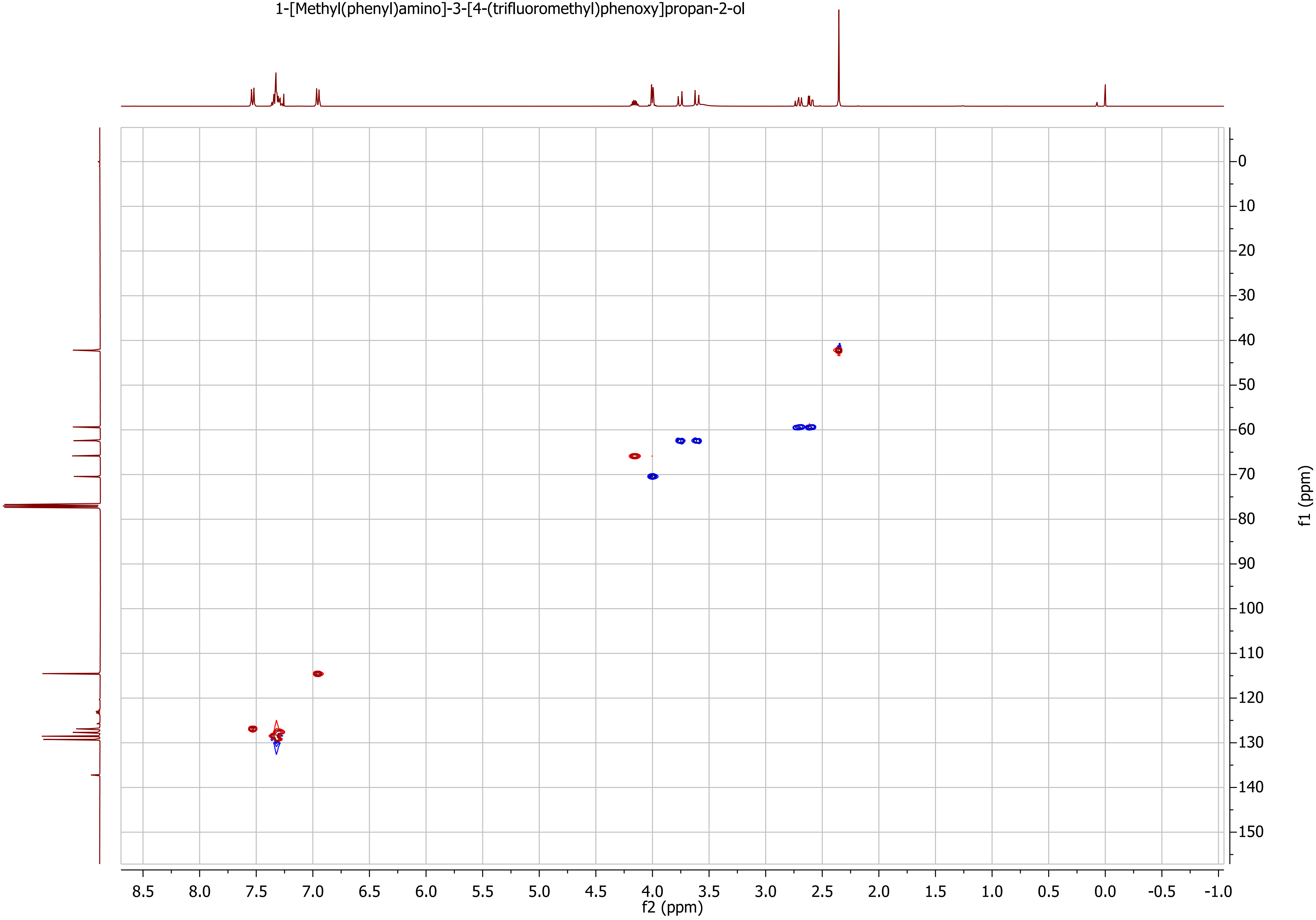

1-(Dimethylamino)-3-[4-(trifluoromethyl)phenoxy]propan-2-ol

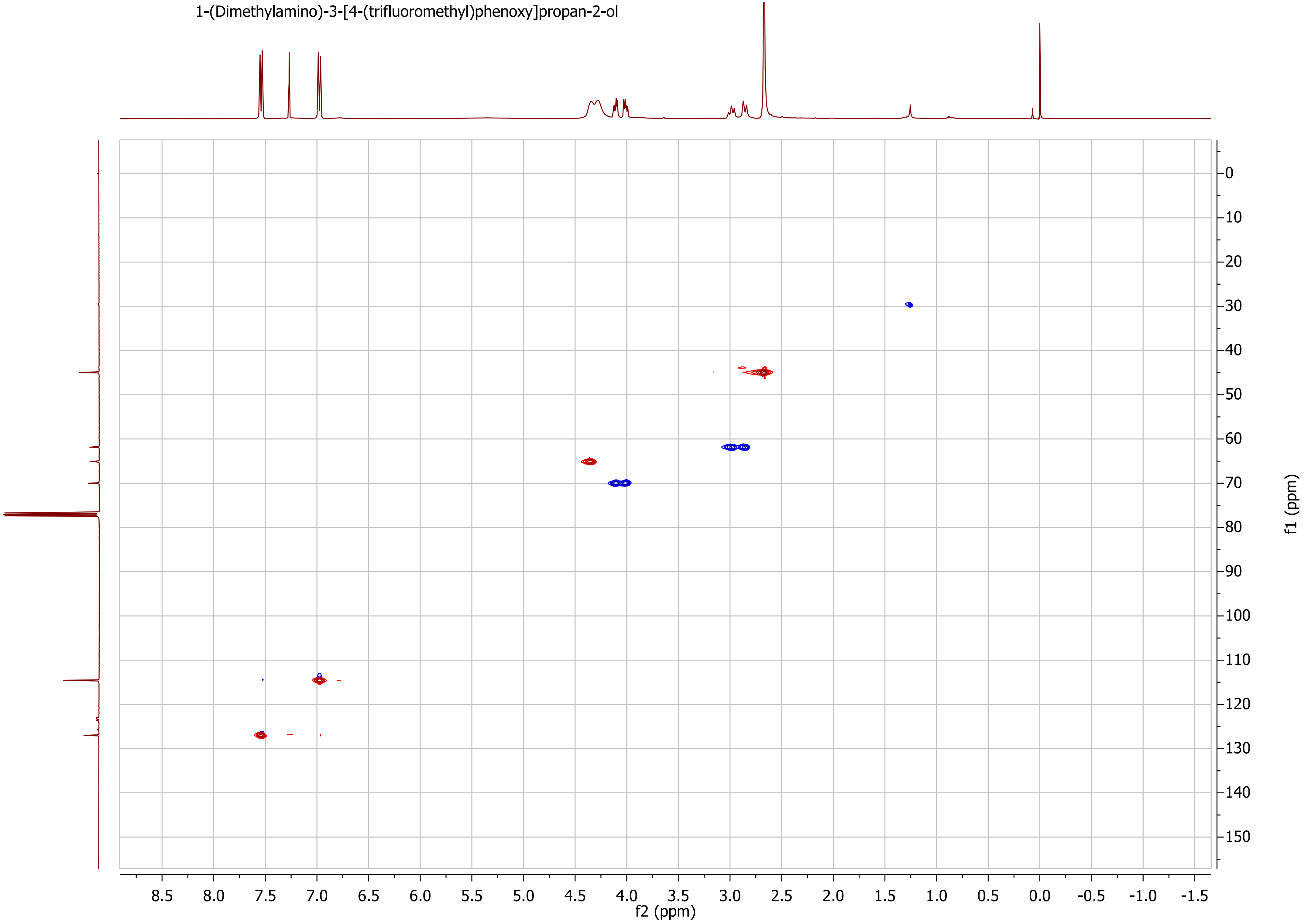

1-(4-Bromophenoxy)-3-(dibenzylamino)propan-2-ol

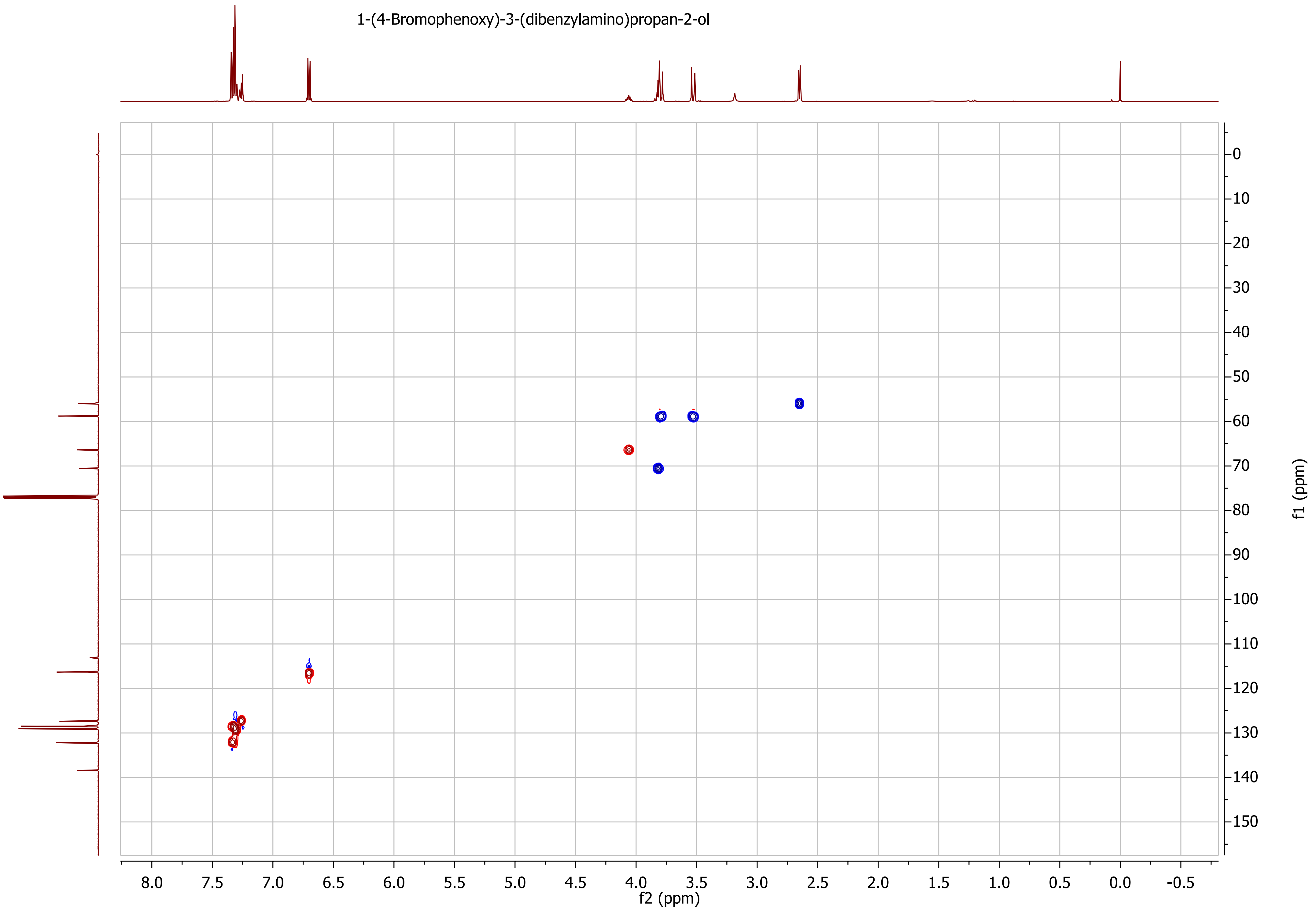

1-(Dibenzylamino)-3-(4-nitrophenoxy)propan-2-ol

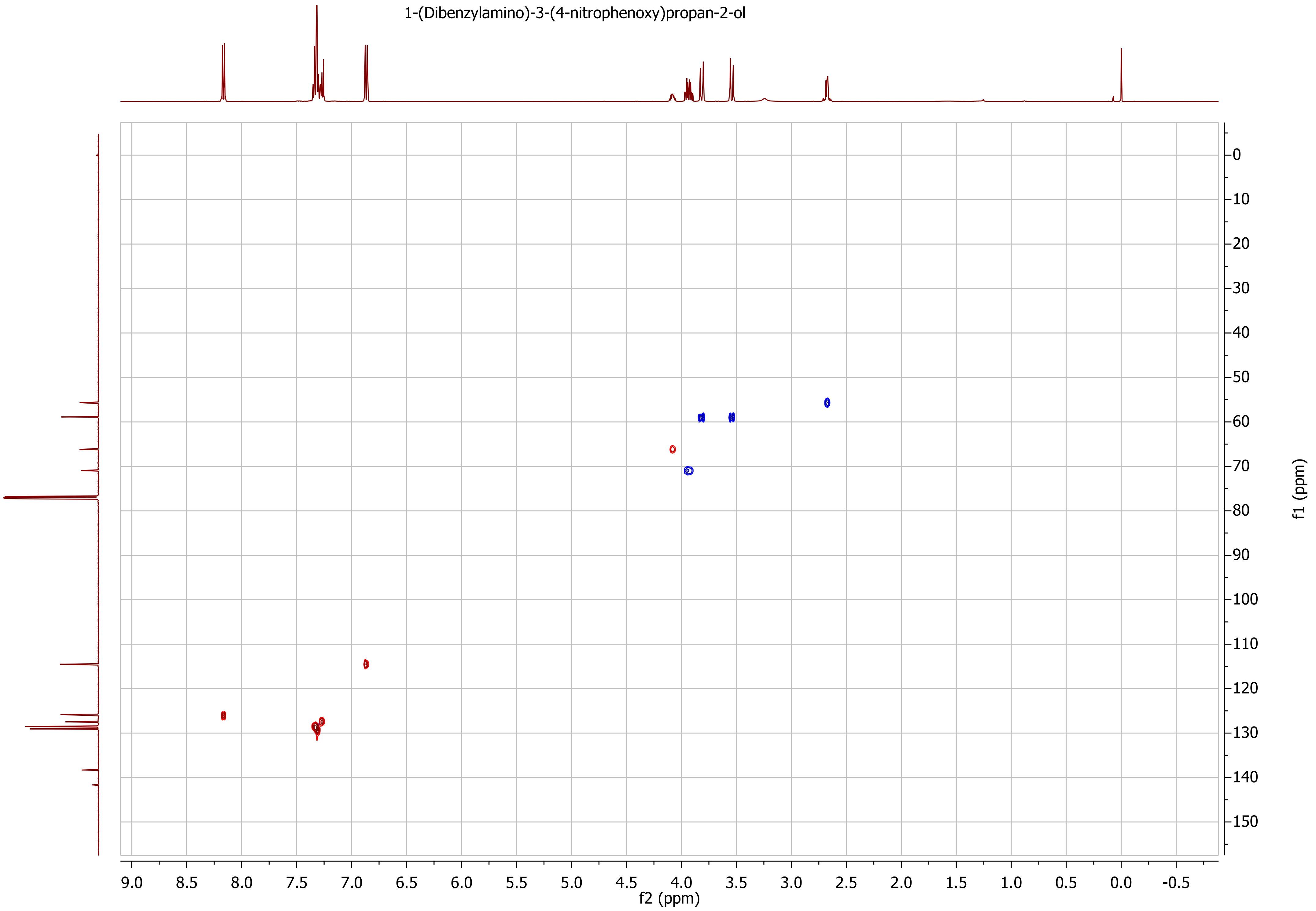

1-(Dibenzylamino)-3-(4-iodophenoxy)propan-2-ol

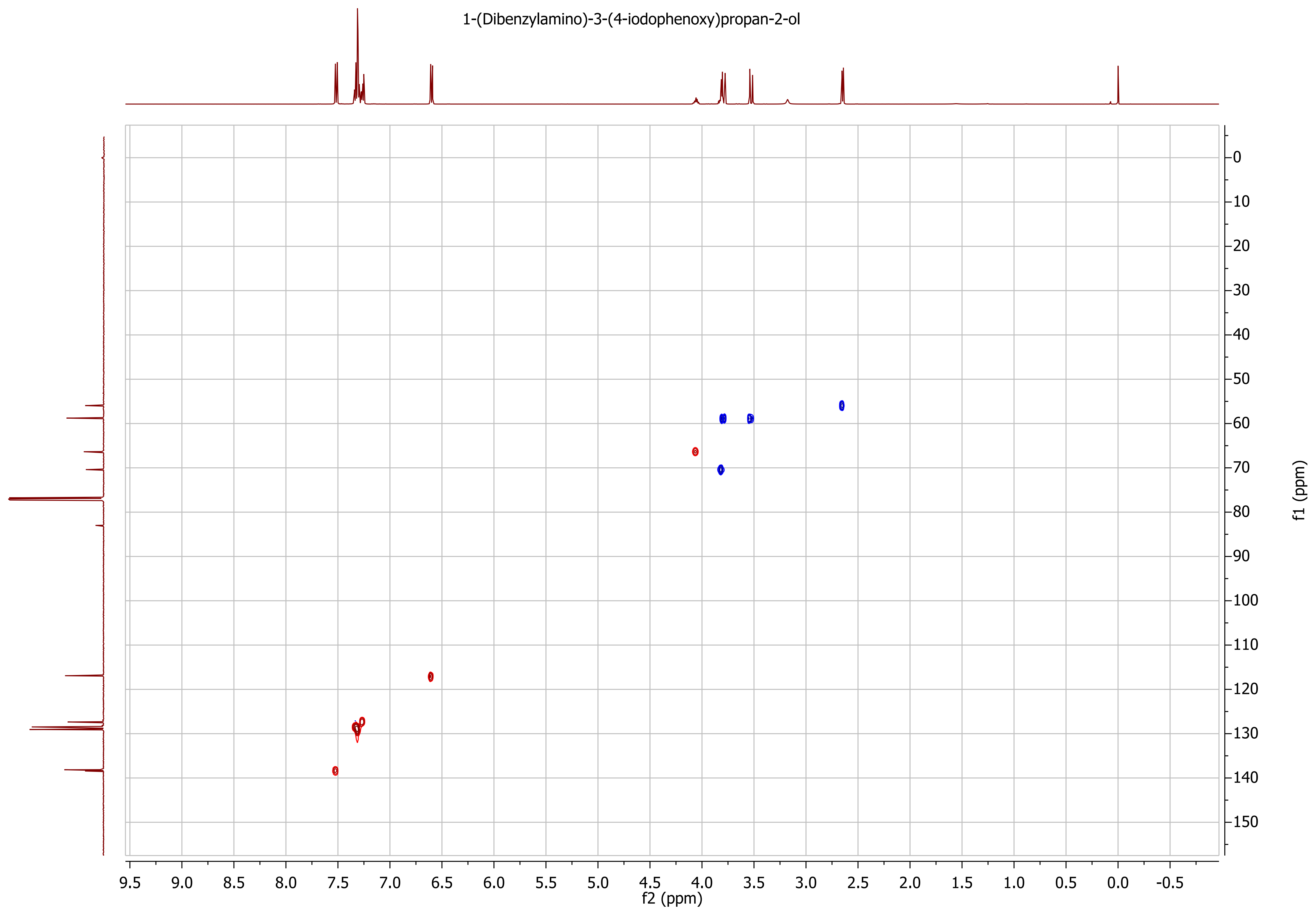

1-(Dibenzylamino)-3-[4-(pentafluoro-λ6-sulfaneyl)phenoxy]propan-2-ol

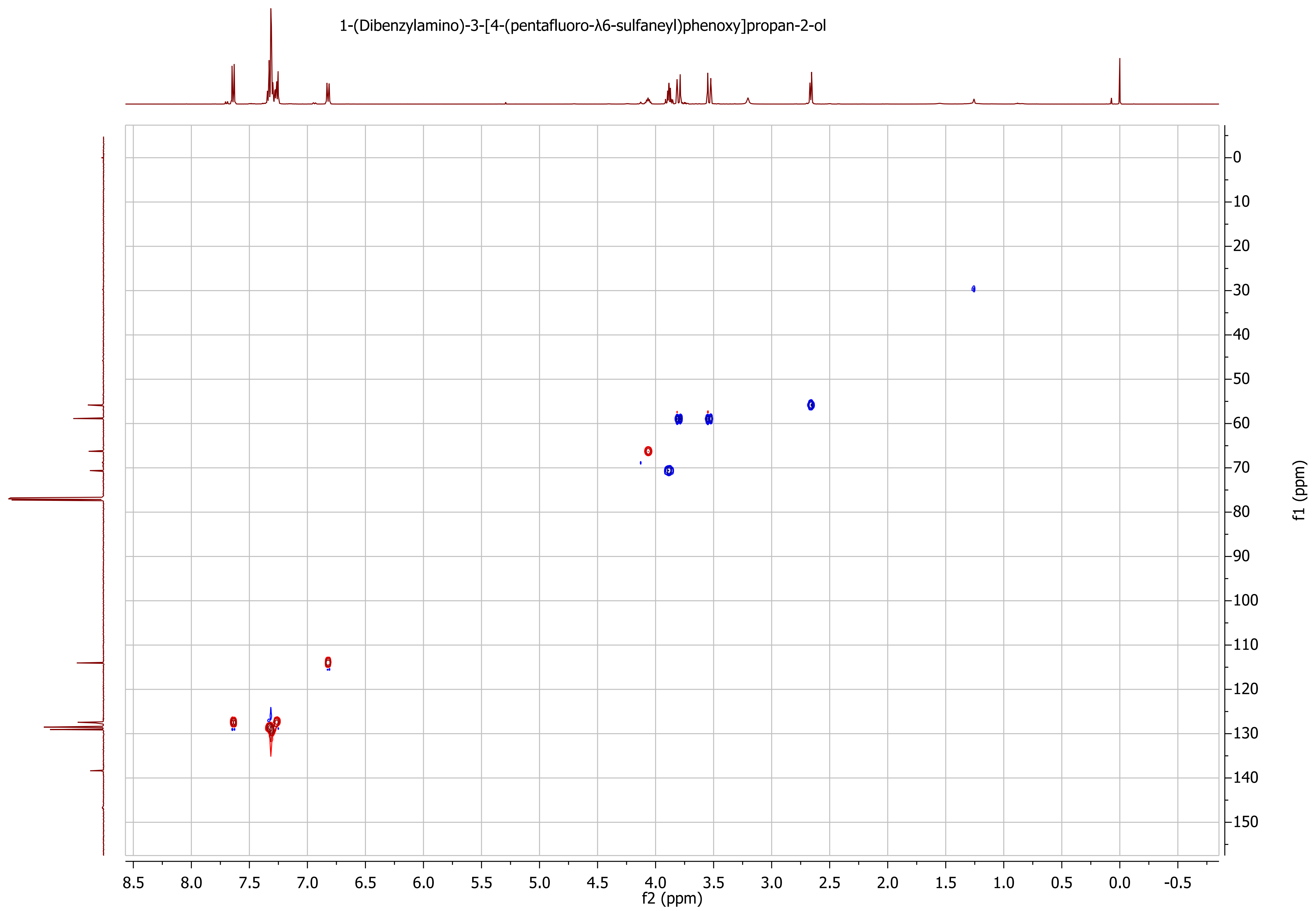

1-[Benzyl(4-chlorobenzyl)amino]-3-(4-chlorophenoxy)propan-2-ol

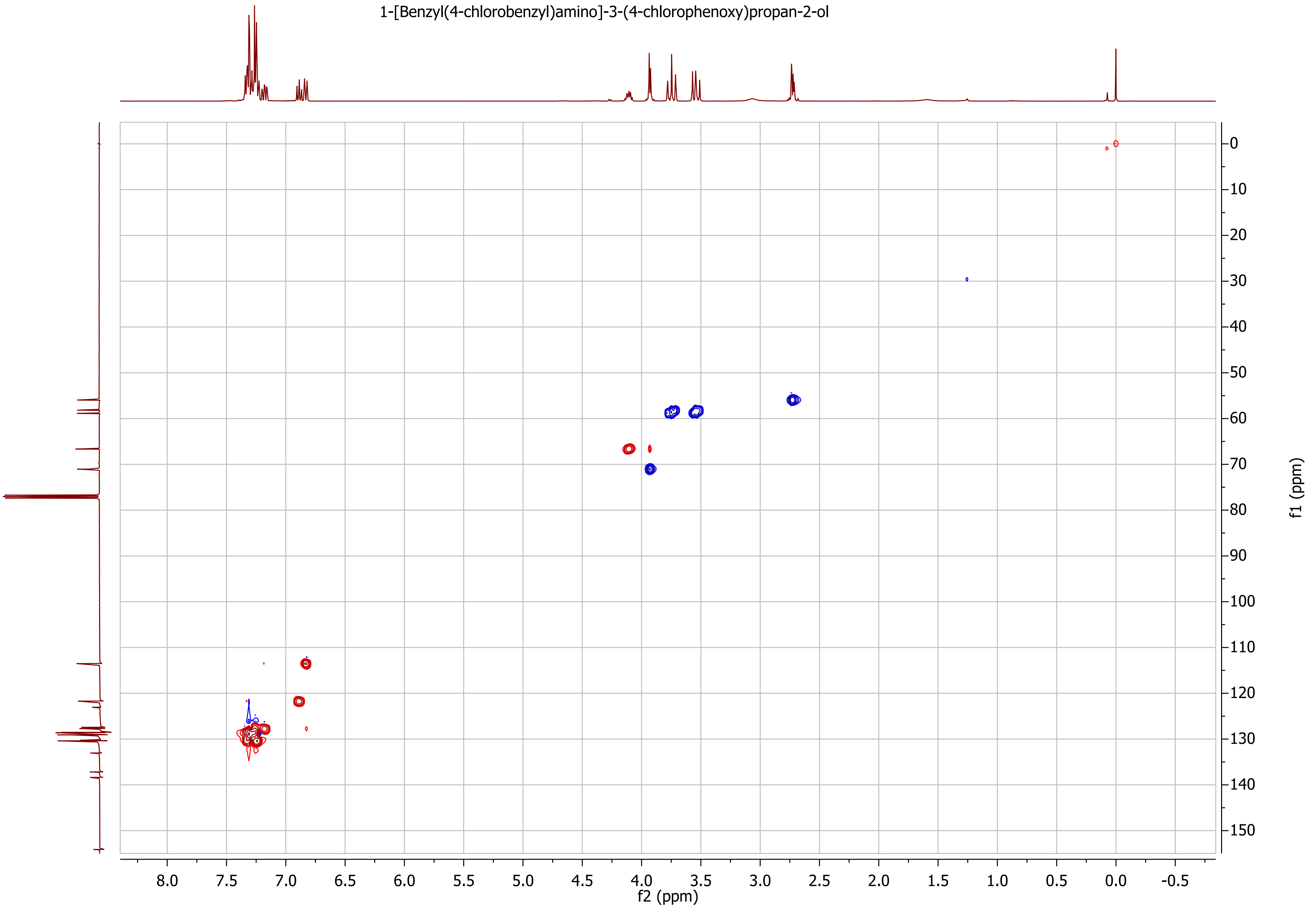

1-[Benzyl(4-methylbenzyl)amino]-3-(4-chlorophenoxy)propan-2-ol

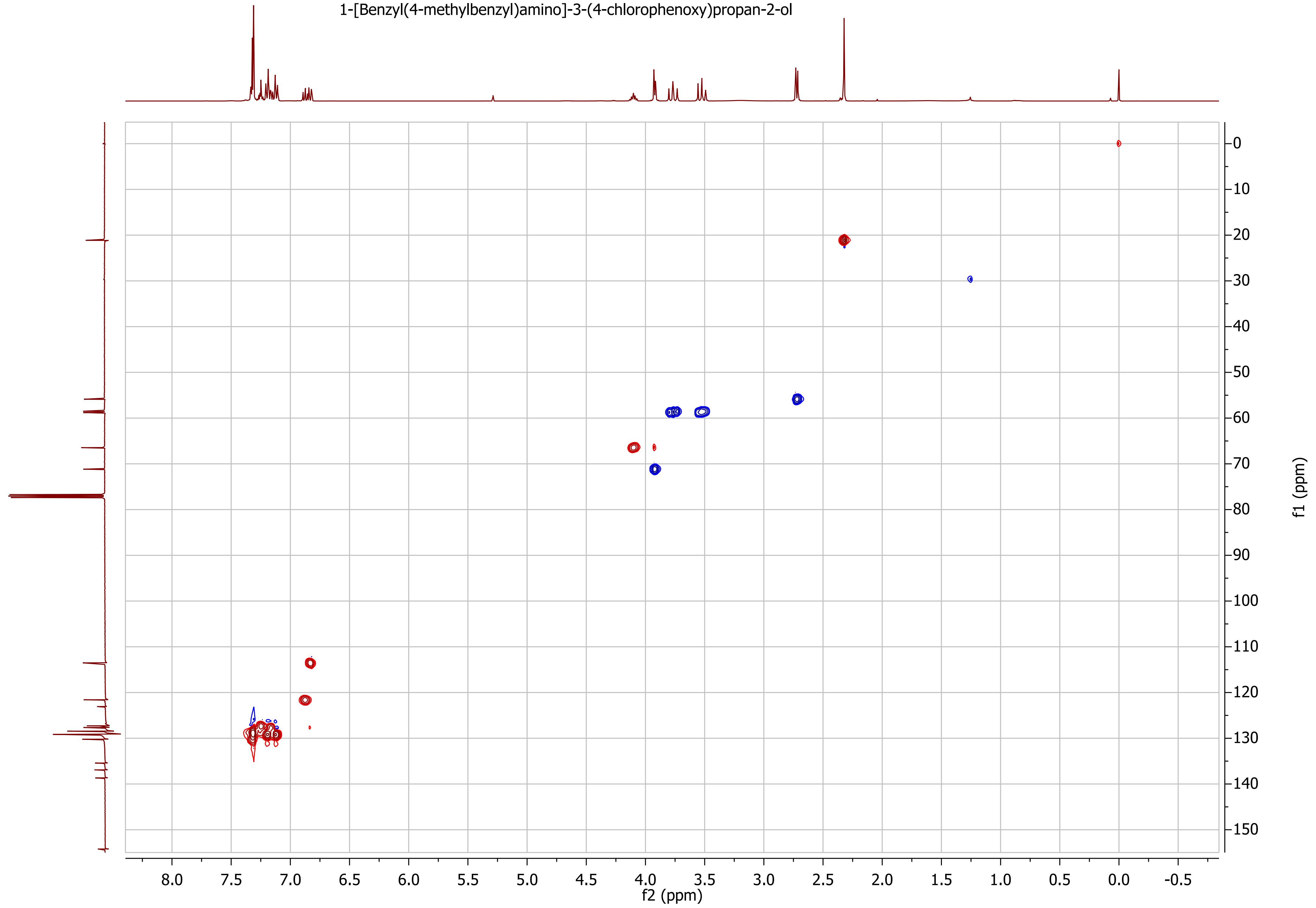

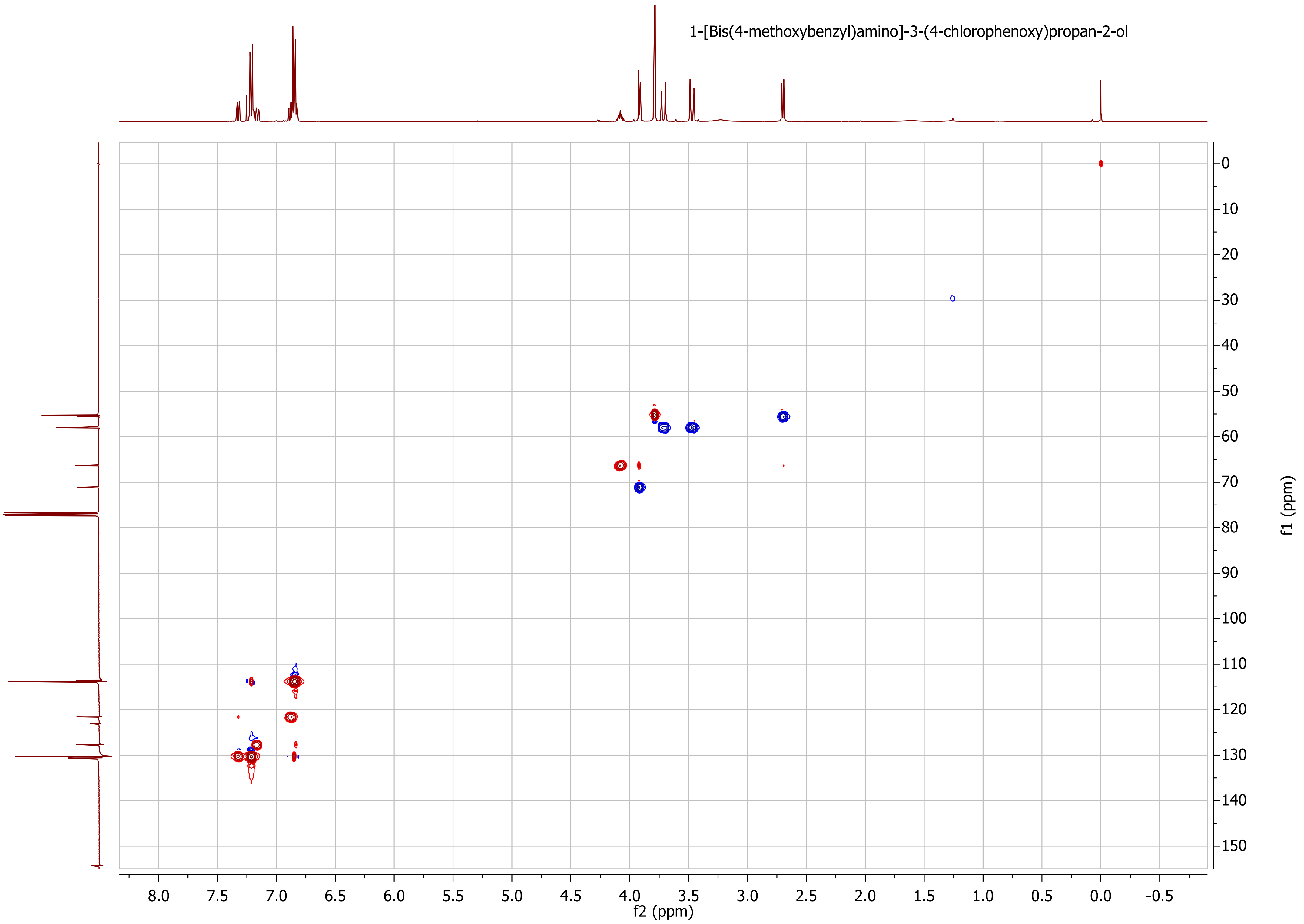
1-[Bis(4-methoxybenzyl)amino]-3-(4-chlorophenoxy)propan-2-ol
f2 (ppm)
f1 (ppm)

1-[Benzyl(3,4-dichlorobenzyl)amino]-3-(4-chlorophenoxy)propan-2-ol

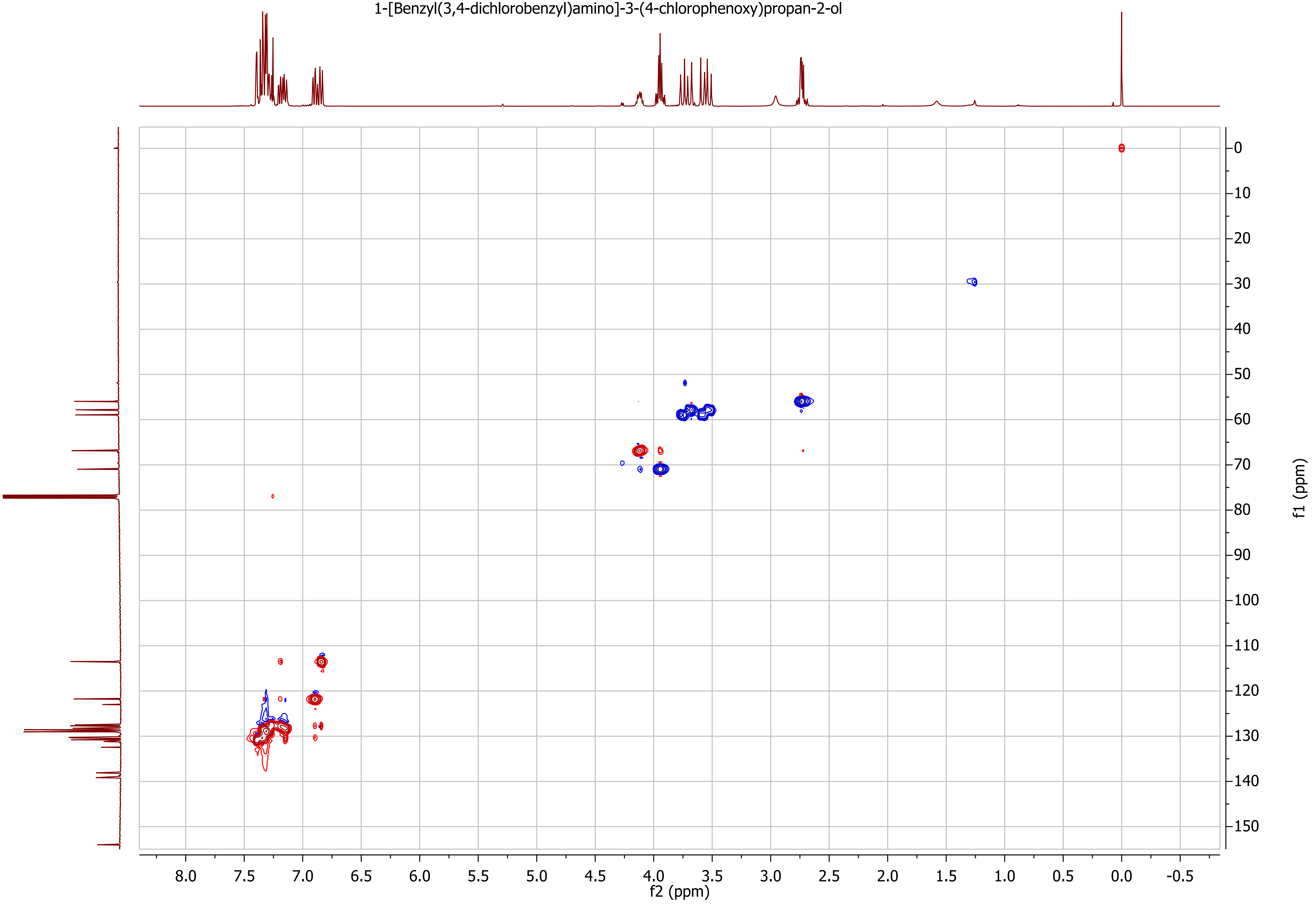

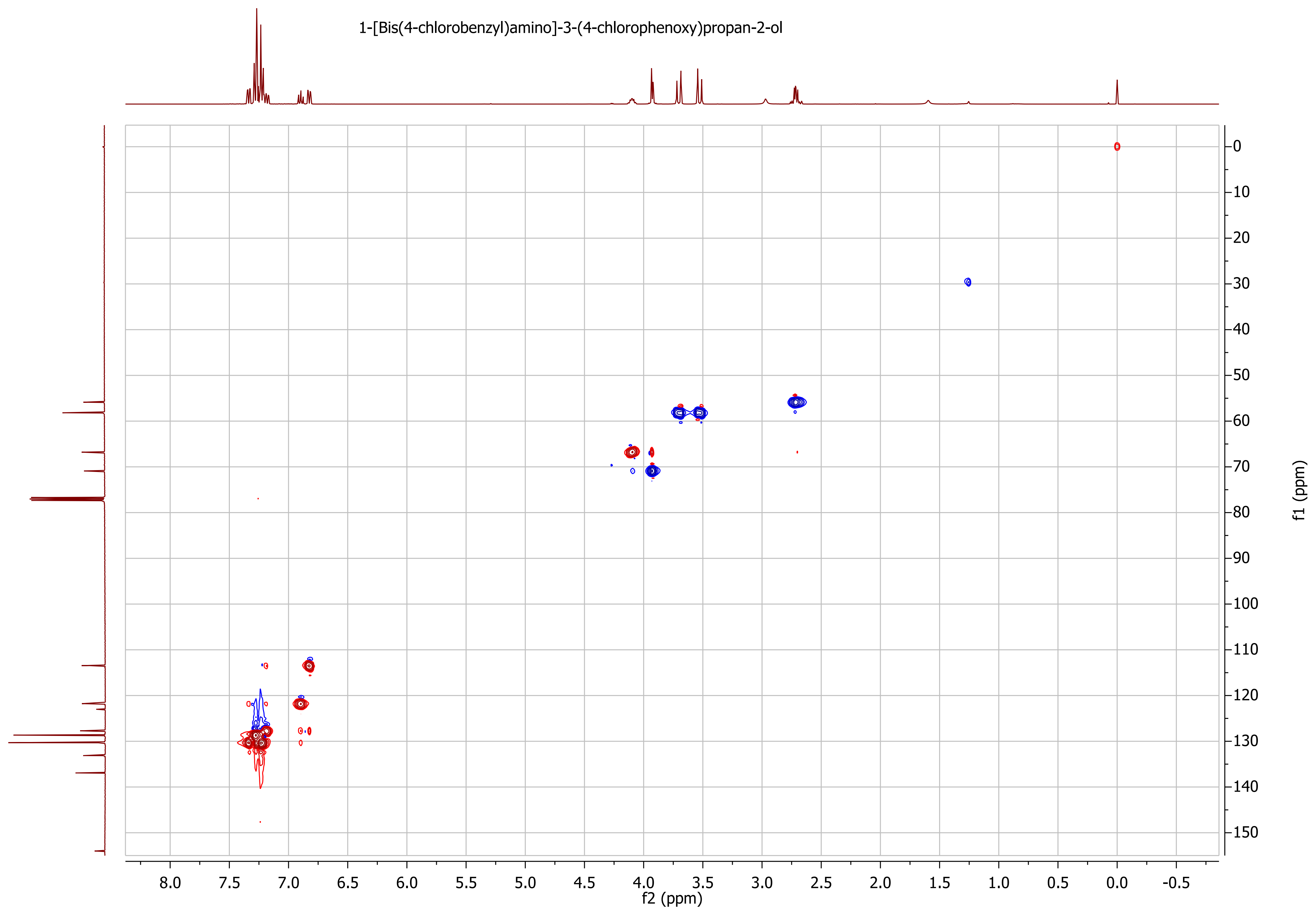
1-[Bis(4-chlorobenzyl)amino]-3-(4-chlorophenoxy)propan-2-ol
f2 (ppm)
f1 (ppm)

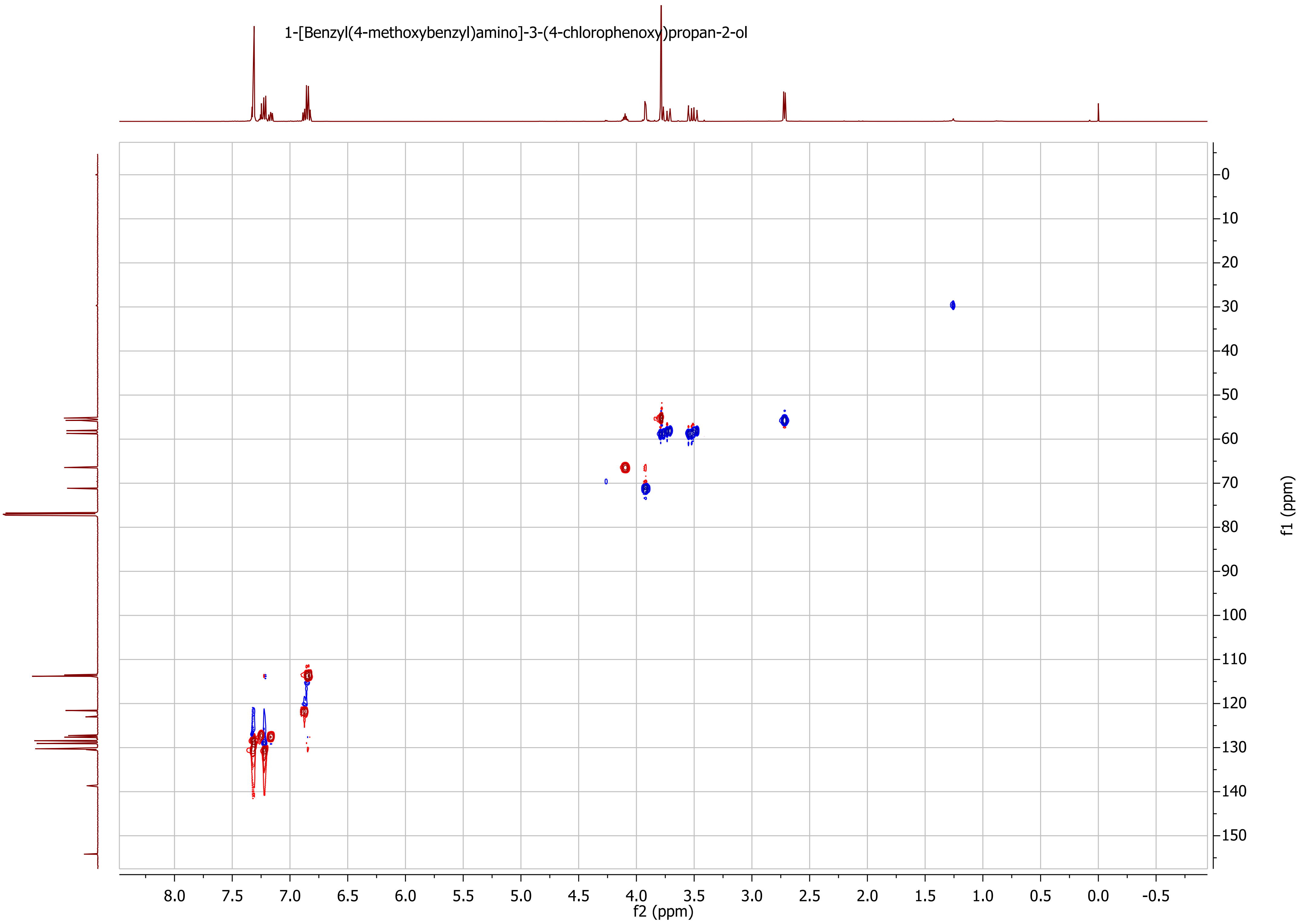
1-[Benzyl(4-methoxybenzyl)amino]-3-(4-chlorophenoxy)propan-2-ol
f2 (ppm)
f1 (ppm)

1-[(4-Chlorobenzyl)(3,4-dichlorobenzyl)amino]-3-(4-chlorophenoxy)propan-2-ol

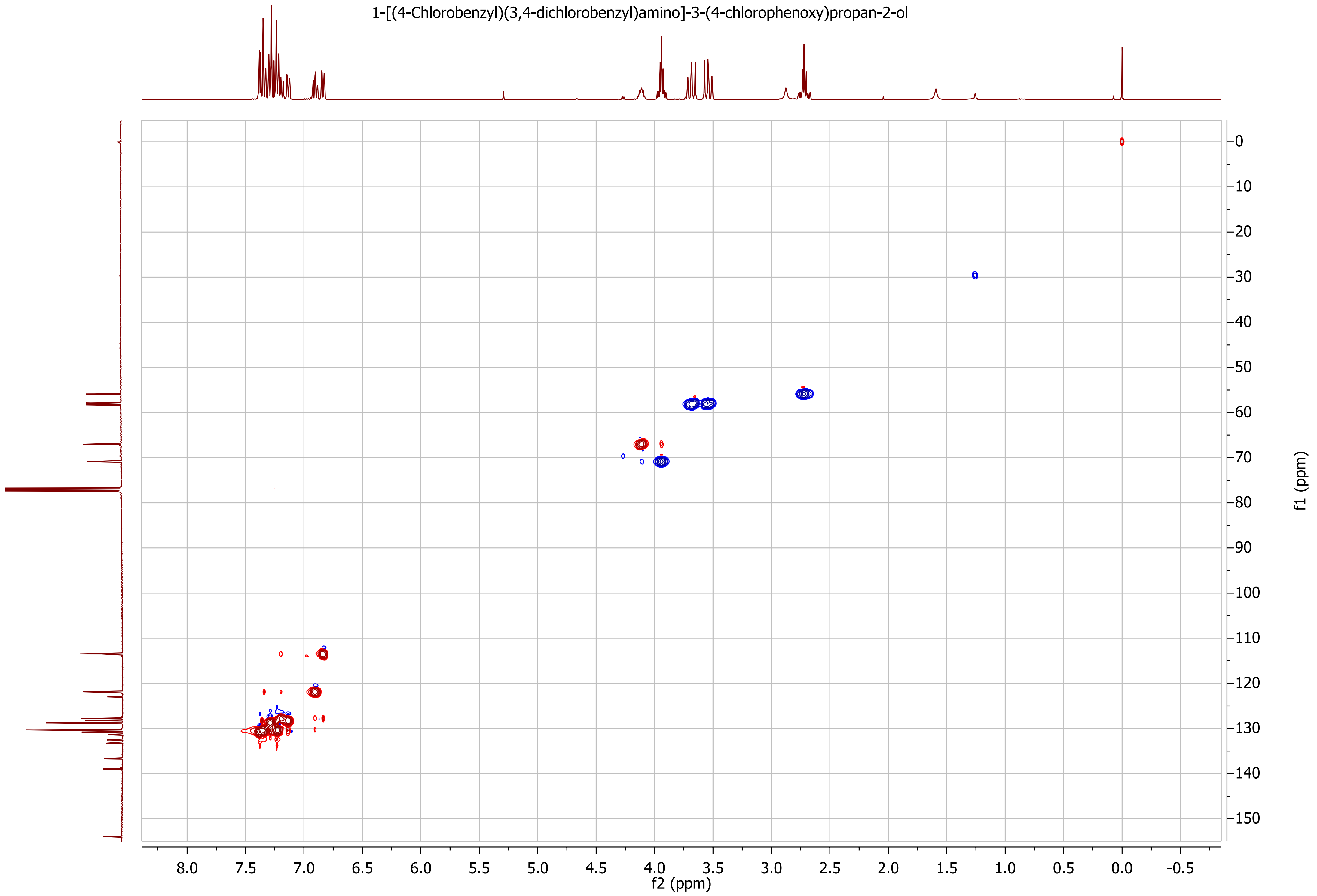

Supplement: Supplementary file 1 [file molecules-28-05468-s001.zip › molecules-2472650-supplementary.pdf]
